# ﻿But wait, there’s more! Descriptions of new species and undescribed sexes of flattie spiders (Araneae, Selenopidae, *Karaops*) from Australia

**DOI:** 10.3897/zookeys.1150.93760

**Published:** 2023-02-27

**Authors:** Sarah C. Crews

**Affiliations:** 1 California Academy of Sciences, Department of Entomology, 55 Music Concourse Drive, San Francisco, CA, 94118, USA California Academy of Sciences, Department of Entomology San Francisco United States of America

**Keywords:** Kimberley, New South Wales, Northern Territory, Pilbara, Queensland, South Australia, taxonomy, Western Australia

## Abstract

Nineteen new species of *Karaops* are described: *K.durrantorum***sp. nov.** (♂), *K.morganoconnelli***sp. nov.** (♀♂), *K.joehaeneri***sp. nov.** (♀), *K.dalmanyi***sp. nov.** (♀♂), *K.garyodwyeri***sp. nov.** (♂), *K.dejongi***sp. nov.** (♀♂), *K.malumbu***sp. nov.** (♀♂), *K.conilurus***sp. nov.** (♂), *K.yumbubaarnji***sp. nov.** (♀♂), *K.markharveyi***sp. nov.** (♀♂), *K.nitmiluk***sp. nov.** (♀), *K.kennerleyorum***sp. nov.** (♂), *K.jawayway***sp. nov.** (♀), *K.mparntwe***sp. nov.** (♀), *K.larapinta***sp. nov.** (♀), *K.kwartatuma***sp. nov.** (♂), *K.madhawundu***sp. nov.** (♀), and *K.mareeba***sp. nov.** (♀). The male of *K.umiida* Crews, 2013 was found to be misidentified and is now *K.conilurus***sp. nov.***Karaopsyindjibarndi***syn. nov.** is a new synonym of *K.nyiyaparli*. *Selenopsaustraliensis* L. Koch, 1875 is considered a nomen dubium because the holotype is an immature male, and the species previously referred to as *K.australiensis* (L. Koch, 1875) is here described as *K.strayamate***sp. nov.** (♀♂). The males of *K.marrayagong* Crews & Harvey, 2011 and *K.banyjima* Crews, 2013 are described for the first time. To manage the growing diversity of the genus, most of the species have been placed in species groups, which are diagnosed. They are the Central Desert group, the *strayamate* group, the *raveni* group, the *dawara* group, the *francesae* group, the Kimberley group, and the Pilbara-Gascoyne group. New keys are provided to accommodate the new species, and new distribution maps and new records are provided for all species. Diagnoses and descriptions are emended where required. Images of live spiders, many not previously seen alive, and natural history information are also provided.

## ﻿Introduction

The genus *Karaops* Crews & Harvey, 2011 was recently described (Crews and Harvey 2011) to accommodate the selenopids, a type of flattie spider, found in Australia. Until 2011, only a single species had been described, *Selenopsaustraliensis* L. Koch 1875 (now *K.strayamate* sp. nov.). Crews and Harvey (2011) described 23 new species of the genus, and in 2013, Crews (2013) described 13 additional species. Here, 19 more species are described, with one synonym, bringing the total number of *Karaops* to 54. Molecular data (Suppl. material 1) indicate that there are myriad species that remain undescribed due to a lack of adult specimens, and there are some adults that are awaiting description (unpubl. data). Given the number of new species found by searching previously unsearched areas during 15,000 km of targeted fieldwork, there are assuredly many more as yet unknown *Karaops*.

Due to this diversity, species groups have been erected into which most species are placed. Diagnoses are provided below for: The Central Desert species group, the *strayamate* group, the *raveni* group, the *dawara* group, the *francesae* group, the Kimberley group, and the Pilbara-Gascoyne group.

Selenopids are known to have the fastest turning strike of any terrestrial animal (Zeng and Crews 2018). They are also extremely fast in general, nocturnal, cryptic, and of course, flat. Thus, many people have difficulty finding them, and if they do, they have difficulty capturing them. This is likely why they can be very common locally but are often poorly represented in museum collections. Twenty-four species of *Karaops* are known from a single sex (with little chance of any single males and females being paired). Only six of the 54 species are represented by both sexes and with more than ten adult specimens.

Because the distributions are poorly known, it is difficult to comment on geographical patterns. However, there appears to be widespread species with pockets of different species that may or may not be closely related to the widespread taxon. Additionally, there are two species groups (*raveni* and *strayamate*) in which the sister taxa are found on the east coast and the west coast of the continent (Map 9, Suppl. material 1). A distributional pattern in which closely related taxa are seen on both the east and west side of the continent with no close relatives in between also occurs in the trapdoor spider genera *Cataxia* Rainbow, 1914 and *Euoplos* Rainbow, 1914 (Rix et al. 2017: figs 288, 321), pholcid spiders (Huber 2001), and plants such as Eucalyptus L’Héritier de Brutelle, 1789 subgenus Eucalyptus (Ladiges et al. 2010: fig. 2), and others.

Many previously described *Karaops* species were collected in pitfall traps left for a month to more than a year, and they are in poor condition. Additionally, dead spiders look nothing like live ones, and live ones in situ do not look the same as they do ex situ. The purpose of the aforementioned fieldtrip was to visit places where the animals had been collected in an attempt to find additional specimens that would be in better condition than those currently available, to try to find missing sexes, to survey areas for new species, and to use the spiders in biomechanical studies. To survey as many areas as possible with limited funds and time, searching in one locality was only possible for a maximum of a few hours. The spiders were fed as the trip proceeded during the course of two months. With the accumulation of nearly 200 specimens, it became somewhat tedious, and many lived for > 1 year after returning with them to the United States. Although not all of the previously collected species were found, two previously undescribed sexes were found, multiple previously described adults in decent to excellent condition representing eight species that were only known from a few adult specimens were collected, an additional 13 new species described here were found (some of the newly described species were not collected on this trip), and of course thousands of places remain unsurveyed.

Rearing the spiders proved to be extremely important for obtaining adults. Of the new species/sexes, nearly all of them were able to be described from maturing in captivity. Ten of them wouldn’t be described at all, four would only be described from a single sex, and rearing increased the number of adult specimens for four described species. Many of the specimens have been photographed alive, and these photos can be compared with the images of the species in situ as well as to dead specimens. The fieldwork and rearing have also provided information on best practices for how and where to find the spiders in a timely manner, how to keep them alive, and a better idea of the ranges of some of the species. This is important for determining whether a species warrants short range endemic (SRE) status (Harvey 2002). An SRE is an animal with a restricted distribution, usually less than 10,000 km^2^. The live specimens were also used to obtain prey capture data for biomechanical studies. Finally, the new specimens were used for a biogeographical study (unpubl. data). The 2.5-month field trip has generated a considerable amount of data, underlining the importance fieldwork.

Suppl. material 2: table S1 in the supplementary material contains information about when adults are more likely to be found to improve targeted collecting efforts. These data are included for those reared in captivity, and it is possible that these data may not reflect what occurs in nature, something completely unknown. However, there are data for multiple species that indicate the timing of maturity is the same in nature as it is captivity.

In some cases, new illustrations were made of previously described species for convenience or because better-preserved specimens were found. Diagnoses and/or descriptions are emended where required. Not all of the types were able to be reviewed, so it is possible that further emendations will be necessary.

## ﻿Materials and methods

### ﻿Bioregionalization in Australia

Australia has a long history of bioregionalization studies (discussed in Ebach 2012). The Interim Biogeographic Regionalisation of Australia (IBRA) was proposed in 1993–94 and published in 1995 by the Australian Department of Environment and Heritage (Thackway and Cresswell 1995). The primary goal was to identify areas that should be prioritized for conservation purposes to determine how funds are best distributed. There are currently 89 regions and 419 subregions recognized. It has been argued that the way the areas are classified is problematic because they are not based on actual biodiversity of an area (Ebach 2012). Here, the IBRA terminology for regions and subregions is used as these data can be useful to examine the validity of particular classifications. Additionally, more detailed data of the abiotic environment and vegetation communities from the immediate collection area can be found via the IBRA.

### ﻿A note on characters used in the descriptions

In previous descriptions of *Karaops* species, characters were described that are static within the genus and so these are not reported for each species and only will be reported when they are unique or species-specific. *Karaops* are diagnosed by a combination of characters (Crews and Harvey 2011), such as no teeth on the tarsal claws and no scopulae, in addition to characters also found in most other selenopids (e.g., fovea are all shallow depressions, the sterna have a posterior indentation, the endites have a conspicuous apical setal tuft). Although the leg formula is obvious when the measurements for each leg are provided, both are reported for convenience. The utility of using leg formulae as a character has been discussed previously, so these are provided out of convention (Crews and Gillespie 2010; Crews 2013). Selenopid legs have a nearly ~ 90° supination at the trochanter joint. Thus, the prolateral part of the leg is effectively dorsal, the dorsal part is effectively retrolateral, etc. When leg terminology indicates dorsal, ventral, pro- or retrolateral, it is referring to the actual surface rather than the effective surface. When the term inner is used, it means toward the midline. For the teeth, 1-0-1 indicates there is a large space between the two teeth.

### ﻿Selenopid genitalia terminology

Using the same terminology for the same characters across taxa is a best practice that is only sometimes followed, even by the same author, including the author of this paper. It can be difficult to do this if homology is unclear and the terminology changes as more data are obtained. However, having lots of data and working within a single genus or family can make this easier. In addition to using the same terminology within closely related taxa, it is also more prudent to try to use common terms and not conjure new, unnecessary terms if there are functional ones already in use.

To try to maintain uniformity of genitalia terminology within the family, the terms that are used are primarily from the Spider Anatomy Ontology on BioPortal (Ramírez and Michalik 2014, 2019). In the past, different terms have been used to refer the same structure, and terms have been used incorrectly. Although some of these terms have synonyms, the ones used here will hopefully be used for future studies of *Karaops* and selenopids in general, if applicable. There are known to be other structures in both males and females of some members of the family that are not mentioned here as they are not present in this genus, and thus, not used in this paper.

On the epigyne, the following terms are used: copulatory openings, epigynal plate, epigynal pockets, epigynal windows, lateral lobes, median field, posterior excavation. Most are straightforward and used across many spider families. Selenopids typically have two copulatory openings, although in some cases, these can be located in a single atrium, as illustrated in the Kimberley species group of *Karaops* (i.e., Figs 21C, D, 23A, B, 28F, G; Crews 2011: figs 119, 120). The term epigynal pocket has been applied across a variety of families, and the structures are clearly not homologous across these families. In Salticidae Blackwall, 1841 and Thomisidae Sundevall, 1833, there can be one or two pockets, and it/they is/are referred to as the “epigynal coupling pocket” because of interaction with the RTA of the male (Edwards 2015, 2017; Maddison et al. 2020). Occasionally, something is called a pocket that actually leads to an opening of sorts. In selenopids, an epigynal pocket resembles an actual empty pocket – that is, there is one way in and out, and there is nothing inside. It is unclear whether any part of the male palp interacts with these pockets because it has not been observed during mating. Epigynal pockets are not present in all selenopid taxa, and they appear to be the most common in *Hovops* Benoit, 1968 and *Selenops* Simon, 1897. It is unclear if they are homologous across genera or even within genera (Corronca and Rodríguez 2011: fig. 2; Crews 2011: fig. 1; Rodríguez and Corronca 2014: fig. 3). They typically are present at the inner edges of the lateral lobes; however, they may also be located along the posterior margin or in the middle of the epigyne. In some species, they are separated and in others, they are fused into a single pocket. Epigynal window is a term that appears most commonly within the Salticidae. It is found across the family but may not be homologous. Here, the term is applied for the epigynes of species from the *dawara* species group (Figs 12A, C, 13C, 14C, E). As in salticids, they are weakly sclerotized and translucent, resembling windows. The copulatory openings are located in the windows. Median field and lateral lobes are terms commonly used across spiders, including Selenopidae. Median field refers to the area in the center of the epigynal plate with ambiguous boundaries. It is not a term to demarcate a structure, but to indicate an area where a specific structure may be located (e.g., a lobe, a depression, an atrium, etc.). Lateral lobes may or may not be conspicuous across selenopid taxa. In some species they are prominent and easily demarcated and in others, there are only remnants, such as small sutures or folds, and finally, sometimes they are not demarcated at all (e.g., Fig. 41C, D vs. Figs 12A, C, 13C, 14E; Crews 2011: figs 29, 30 vs. Crews et al. 2020: fig. 6a–d). In species from the Kimberley species group, there is a median depression which the lateral lobes partially cover that is referred to as an atrium; atrium is also used when discussing this structure dorsally as part of the endogyne (Figs 21B, 23B, 28G, 30C). A posterior excavation refers to the posterior epigynal margin and whether it is straight (e.g., 3A) or indented (Crews and Harvey 2011: fig. 9).

The terms used for structures of the endogyne are copulatory ducts, accessory bulbs, fertilization ducts, posterodorsal fold, spermathecae, and uterus externus. The copulatory openings lead to the copulatory ducts. Copulatory ducts connect the copulatory openings to the sperm storage sites or accessory bulbs (Figs 3D, 21B, 51D). Sometimes the ducts are long and winding (Figs 10E, 12B, D, 13D, 14D, 51D) or are very short (Figs 21B, D, 23B, 44D, F). The spermathecae are where the fertilization ducts arise. Fertilization ducts connect the spermathecae to the uterus externus. The uterus externus can be torn away during dissection, or sometimes requires removal to see other structures, so it is not always present in preserved specimens. Posterodorsal fold indicates an infolding of the cuticle. The author does not know of any other groups where this term has been applied or if other spiders have such a structure. The posterodorsal fold is highly sclerotized unlike the uterus externus. The uterus externus is found between the two folds if they are separated laterally, corresponding to separation of the lateral lobes, or are dorsal to this structure. Posterodorsal folds can be very large, covering nearly all of the other internal structures (Crews 2011: figs 24, 26), medium (Crews 2011: fig. 14), small (Fig. 10E), very small (Fig. 51D), separated (Fig. 55E), and non-existent (Fig. 42E). Because this is an infolding of the epigynal plate, it is assumed that this is homologous across taxa within the family.

For the male palp, the following terminology is used: cymbial chemosensory patch, conductor, conductor sheath, embolus, medial part of the conductor, median apophysis, retrobasal cymbial process, retrolateral tibial apophysis, spermophor, spinules, subtegulum, tegular lobe, tegular sheath, tegulum. The cymbial chemosensory patch in selenopids is straightforward. It is common in *Anyphops* Benoit, 1968 and is also known in *Karaops*, where it occurs as small or larger (Figs 36A, B, 44A, B, 46A, B, E). Conductors vary broadly across Selenopidae (Corronca 2005: fig. 1E; Corronca and Rodríguez 2011: fig. 2H; Crews 2011: figs 7, 79, 169; Crews and Harvey 2011: figs 5, 73, 83), from being fairly simple (Figs 31D, 32A), to extremely elaborate (Figs 36A, 50C, 58A, 60A, 63C, 69A). In *Karaops*, many of the conductors have a sheath that the embolus lies in referred to as a conductor sheath (Fig. 65A). In the Kimberley group, the conductor is usually somewhat crescent shaped, but also has a protrusion that is close to the middle of the bulb, and this is referred to as the medial part of the conductor (Fig. 22A). In *Karaops*, the outer edge of the tegulum is usually formed into a large sheath that houses most of the embolus or the conductor and is sometimes connected to the conductor (Fig. 65A). The emboli of Selenopidae can be long, short, narrow, or wide. In *Karaops*, most of them are fairly long and narrow. There is ambiguity in some selenopids, particularly in *Karaops*, where the tegulum ends and the embolus begins (Figs 4A, 9E). The tegulum extends somewhat ventrally and basally beyond the cymbium. This is referred as the tegular lobe (Figs 4A, 9E). It is unknown whether this term is used or if there is another term in use that indicates the same thing. The origin of the embolus is demarcated where the tegular lobe narrows. The median apophysis in Selenopidae is fairly similar across the family, except in some species of *Anyphops* where it can be extremely large and elaborate (Corronca 2005: fig. 1E), in *Godumops* Crews & Harvey, 2011 where it is non-existent (Crews and Harvey 2011: fig. 5), or it is very small and attached to the tegular lobe in some *Karaops* (Crews and Harvey 2011: figs 7, 13). Some selenopids, *Karaops* included, have a sort of knob retrobasally on the cymbium (Fig. 69A). It is not quite the same as a paracymbium, despite its name being the definition of a paracymbium. Here, it is referred to as the retrobasal cymbial process. The retrolateral tibial apophysis, spermophor, subtegulum, and tegulum are not ambiguous. There is one further term used here, spinules, that has been used before in other taxa for small cuticular extensions. They are not in a socket like spines, and it is unclear whether they articulate. In selenopids, they have been found in *Selenops* (Crews 2018: fig. 2) and *Karaops* (Figs 4A, 9D, 63C) on the median apophyses. They likely occur in more species than documented but because they are difficult to see they may have been overlooked in the past.

### ﻿Images and illustrations

Images of dead specimens were taken with a Leica dissecting microscope and stacked using AutoMontage. Images of live specimens were taken with a Nikon D810 or a Canon 7D. Outlines of illustrations were made by putting a piece of paper on a photograph displayed on a tablet and tracing with a pencil to obtain correct proportions. The illustration was then shaded with a pencil. It was then scanned and put in Adobe Photoshop for additional shading and so that details could be added. Maps were made using QGIS v. 3.26.3 (QGIS.org 2021) with additional details added in Adobe Illustrator.

The figures in this work are with resolution suitable for printing, which is reduced in comparison with the originals, but if you want to see them in full-resolution files, you could contact the author.

### ﻿Locality data and description format

Because all species mentioned in this work are from Australia, the word “Australia” has been eliminated from the locality data and instead replaced with the state, and the state is not repeated for each entry, e.g.:

***Holotype***: Queensland • ♀; base of Jim Crow Mountain; 23°13'S, 150°38'E; Jul. 1982; A. Rozefelds leg.; QMS 61054. Paratype: ♂; Johansen’s Cave, 23°09'S, 150°28'E; 100 m; 29 May 2000; G.B. Monteith leg.; fogging trees with pyrethrum; vine scrub; QMS 57515. Other material examined: 4♀; Bowen, Murray’s Bay Road, Rose Bay Walking Trail; -19.9863, 148.2608; ~ 26 m; 3 Jun. 2019; S. Crews, M. Brandley leg.; under rocks; sel_1438–1441; SCC19_012 • 1♂; Brandy Creek; 20°21'S, 148°43'E; 15 May 1975; R. Monroe, J. Covacevich, P. Filewood leg.; (QMS 47115).

Because there are images of what some of the spiders looked like in life, the colors, patterns, and setal coverage of both live and preserved species are indicated if they differ from one another. They are separated by a slash, e.g.: “Color (in life/preserved): Abdomen: dorsally golden brown, darker medially, with a chevron medially, sides of chevron are angled anteriorly, darker posteriorly, laterally spotted/golden parts yellowish, darker parts orange-red, pattern inconspicuous, dark, thick stubby setae, giving the abdomen a spotted appearance from a distance, ventrally yellowish white.” Thus, the description of a character before the slash indicates how the spider appears in life, and the description of a character after the slash refers to the preserved specimen.

If material examined or new records indicate “reared in captivity”, detailed information can be found in the Supplementary files. Juveniles that have been assigned to species have been determined using molecular data (unpubl. data) or were collected at the exact same time and place as adults.

### ﻿Anatomical structures

**AB** accessory bulb

**ALE** anterior lateral eye

**AME** anterior median eye

**At** atrium

**C** conductor

**CD** copulatory duct

**CO** copulatory opening

**CS** conductor sheath

**Cx** coxa

**Cy** cymbium

**d** dorsal

**E** embolus

**FD** fertilization duct

**Fm** femur

**LL** lateral lobe

**MA** median apophysis

**MF** median field

**mpc** medial part of the conductor

**Mt** metatarsus

**pdf** posterodorsal fold

**PLE** posterior lateral eye

**PME** posterior median eye

**pr** prolateral

**Pt** patella

**rbcp** retrobasal cymbial process

**rl** retrolateral

**RTA** retrolateral tibial apophysis

**S** spermatheca

**Sp** spinules

**St** subtegulum

**Ta** tarsus

**Ti** tibia

**TL** tegular lobe

**Tr** trochanter

**TS** tegular sheath

**UE** uterus externus

**v** ventral

### ﻿Repositories

**AM**Australian Museum (Graham Milledge, Helen Smith)

**MAGNT**Museum and Art Gallery of the Northern Territory (Gavin Dally)

**QM**Queensland Museum (Michael Rix, Owen Seeman)

**SAM**South Australia Museum (Leslie Chisholm)

**WAM**Western Australian Museum (Mark S. Harvey, Julianne Waldock)

**ZMT** Zoological Museum Turku (Varpu Vahtera)

**ZSMH** Zoologisches Museum, Hamburg (Danilo Harms, Nadine Dupérré)

## ﻿Taxonomy

### ﻿Family Selenopidae Simon, 1897

#### ﻿ Selenops

Taxon classificationAnimaliaAraneaeSelenopidae

Latreille, 1819

723C5652-4753-5100-9D57-3D5FDF9F9E3B

##### Type species.

*Selenopsradiatus* Latreille, 1819.

#### ﻿Genus Karaops

Taxon classificationAnimaliaAraneaeSelenopidae

Crews & Harvey, 2011

6041A88E-280C-5573-BB59-72F4DF46EDAA

##### Type species.

*Karaopsellenae* Crews & Harvey, 2011.

##### Diagnosis.

*Karaops* do not have scopulae or teeth on the tarsal claws. In almost all species (except *K.yumbu* Crews, 2013), the embolus arises from a tegular lobe.

##### Nomen dubium, here designated.

*Selenopsaustraliensis* L. Koch 1875: 615, pl. 48, fig. 6; L. Koch 1876: 832, pl. 71, fig. 3.

##### Discussion.

In Crews and Harvey (2011) it was reported that the holotype of *Karaopsaustraliensis* was a juvenile. The types in ZSMH were not examined at that time. A re-examination of L. Koch (1875) lists the figure from page 615, pl. 48, fig. 6 as “Femina”, although the illustration appears to be an immature male. The material at the ZSMH was photographed, and ZSMH-A0000791 is the holotype juvenile male from Bowen. The other specimen, ZSMH-A0000792, is the female, presumably, from L. Koch (1876), denoted as the syntype. The locality data for the female denoted as the syntype says Sydney. After closer examination of the holotype male, it is believed that this is actually an immature of *K.raveni* Crews & Harvey, 2011 or a member of the *raveni* species group based on the leg spination and the curvature of the eye rows; thus, it could also have been found in Sydney as *K.raveni* and *K.marrayagong* Crews & Harvey, 2011 of the *raveni* group both occur there. Adult specimens previously determined as *Selenopsaustraliensis*, and that are here described as members of the new species *Karaopsstrayamate* sp. nov., occur far from New South Wales. Because of the uncertainty and inability to determine to which specimen Koch was referring to, *K.australiensis* is considered a nomen dubium.

##### Distribution.

Australia excluding Tasmania.

##### Composition.

With the new species described here, there are now a total of 54 *Karaops* species. Several others for which adults have not been collected are known via molecular and locality data.

### ﻿Key to *Karaops* species, species groups, and unplaced species

An attempt has been made to use characters that remain after preservation and that are not variable, but there are so few specimens of some species, the key will likely need to be emended when additional specimens are collected (males of *Karaopsmadhawundu* sp. nov. and *K.mareeba* sp. nov. are unknown, and the female of *K.kennerleyorum* sp. nov. is unknown).

**Table:** 

1	Females	**2**
–	Males	**12**
2(1)	With epigynal windows (Figs 10D, F, 12A, C, 13C, 14C, E)	***dawara* species group**
–	Without epigynal windows (Figs 3A, 21A, 33B, 41C, 42C, 44C, 49C)	**3**
3(2)	Copulatory openings located within a central atrium, copulatory ducts indistinct from one another at their origins (Figs 21A–D, 23A, B, 28F, G, 30B, C; Crews 2013: figs 1, 2)	**Kimberley species group**
–	Copulatory openings not located within a central atrium, copulatory ducts distinct at their origins (Figs 3A, 33B, 41C, 42C, 44C, 49C)	**4**
4(3)	Copulatory ducts with several tight coils (Crews and Harvey 2011: figs 10, 12, 16, 76 [erroneously referred to as SD = sperm duct])	***strayamate* species group**
–	Copulatory ducts otherwise (Figs 3A, 33B, 41C, 42C, 44C, 49C)	**5**
5(4)	With unsclerotized median lobe, copulatory ducts short, with only one turn or coil (Fig. 3A–F; Crews and Harvey 2011: figs 47, 48, 53, 54, 57, 58, 59, 60, 63, 64)	**Central Desert species group**
–	Without unsclerotized median lobe, or if one is present, copulatory ducts long with several turns (Figs 33A, B, 41C, D, 42C, E, 44C–F, 49C, D; Crews 2013: figs 13, 14)	**6**
6(5)	Leg I and II Mt and Ta spination 5, 4	**7**
–	Leg I and II Mt and Ta spination otherwise	**8**
7(6)	Lateral lobes indistinct and copulatory openings difficult to discern (Fig. 41 C, D)	***K.madhawundu* sp. nov.**
–	Lateral lobes more distinct and copulatory openings easily discernable (Figs 49C–F, 51C–F, 53B, C, 55B–E, 59B, C, 60C, D; Crews and Harvey 2011: figs 33, 34, 37, 38, 41, 42, 43–46; Crews 2013: figs 5–10, 13–16, 25, 26, 31–34)	**Pilbara-Gascoyne species group**
8(6)	Copulatory openings located beneath m-shaped hoods (Fig. 33B, C)	***K.yumbubaarnji* sp. nov.**
–	Copulatory openings not located beneath m-shaped hoods	**9**
9(8)	Large, round accessory bulbs with no coiling of copulatory ducts (Figs 42C, E, 44C–F, 48D, E)	**10**
–	Accessory bulbs and copulatory ducts otherwise (Crews and Harvey 2011: figs 25–28, 31, 32, 67–70)	**11**
10(9)	Copulatory openings beneath small, sclerotized lobe that does not reach epigynal plate margin (Fig. 42C)	***K.mareeba* sp. nov.**
–	Copulatory openings in small depression of median field (Figs 44C, E, 47D)	***K.markharveyi* sp. nov.**
11(9)	Long, diamond-shaped to triangular median field and no epigynal pockets (Crews and Harvey 2011: figs 25, 27, 31)	***raveni* species group**
–	Without well-defined median field, with epigynal pockets (Crews and Harvey 2011: figs 67, 69)	***francesae* species group**
12(1)	With unpaired spines ventrally on Ti, Mt I and II	***raveni* species group**
–	With paired spines ventrally on Ti, Mt I and II	**13**
13(12)	Median apophysis with two conspicuous branches or a branch and a lobe (Fig. 4C; Crews and Harvey 2011: figs 49, 51, 55, 61, 65, 71)	**14**
–	Median apophysis with single branch or second branch very difficult to see (Figs 19A, 36C, 39A, 46A, 50C, 65A; Crews and Harvey 2011: figs 7, 13; Crews 2013: figs 11, 17, 19, 21, 35)	**15**
14(13)	Cheliceral promargin with more than 3 teeth, retromargin with more than 2 teeth	***francesae* species group**
–	Promargin with 3 teeth, retromargin with 2 teeth	**Central Desert species group**
15(13)	With prominent cymbial chemosensory patch (Figs 36B, 46B, E)	**16**
–	Without prominent cymbial chemosensory patch	**17**
16(15)	Median apophysis shaped like a bird’s head distally (Fig. 46A)	***K.markharveyi* sp. nov.**
–	Median apophysis not shaped like a bird’s head distally (Fig. 36A, C)	***K.yumbubaarnji* sp. nov.**
17(15)	Long palpal tibia, ratio of cymbium length to palpal tibia length > 0.60 (Figs 18D, 19A, B, 20C, 22A–C, 25B, D–F, 26B, 27B, E, F, 29A–C, 31B, D, E, 32A–C)	**Kimberley species group**
–	Ratio of cymbium length to palpal tibia length < 0.65	**18**
18(17)	Median apophysis not distinctly separate from tegular lobe (Crews and Harvey 2011: figs 7, 13, 73)	***strayamate* species group**
–	Median apophysis distinctly separate from tegular lobe	**19**
19(18)	Cymbium round and rather flat dorsoventrally, with median apophysis at tip of large, unsclerotized retromedial extension of tegulum (Crews 2013: figs 35, 36)	***K.yumbu***
–	Cymbium and median apophysis otherwise	**20**
20(19)	Conductor sheath curved to a point with terminus directed laterally across middle of bulb (Fig. 39A)	***K.kennerleyorum* sp. nov.**
–	Conductor large, extended, twisted, curved, or arched, but terminus not directed laterally across middle of bulb (Figs 50C, 56A, 58A, 60A, 63C, 65A; Crews 2013: figs 11, 17, 19, 21, 23)	**Pilbara-Gascoyne species group**

#### The Central Desert species group

**Diagnosis.** The epigynes of the Central Desert species group have fleshy median lobes and large, round accessory bulbs that are connected to the spermathecae by a short duct that is coiled once or twice (Fig. 3C, D). The median apophysis of this species group has two conspicuous branches or a lobe, one is sclerotized and the other is not (Fig. 4A; Crews and Harvey 2011: figs 49, 51, 55, 61).

**Composition.** At least eight species make up the Central Desert species group: *Karaopsmanaayn* Crews & Harvey, 2011 (♀♂), *K.vadlaadambara* Crews & Harvey, 2011 (♀♂), *K.pilkingtoni* Crews & Harvey, 2011 (♀♂), *K.deserticola* Crews & Harvey, 2011 (♀), *K.ngarutjaranya* Crews & Harvey, 2011 (♀♂), *K.larapinta* sp. nov. (♀), *K.mparntwe* sp. nov. (♀), and *K.kwartatuma* sp. nov. (♂). Other undescribed species are known from this group, and the region is poorly explored regarding these animals.

**Map 1.**
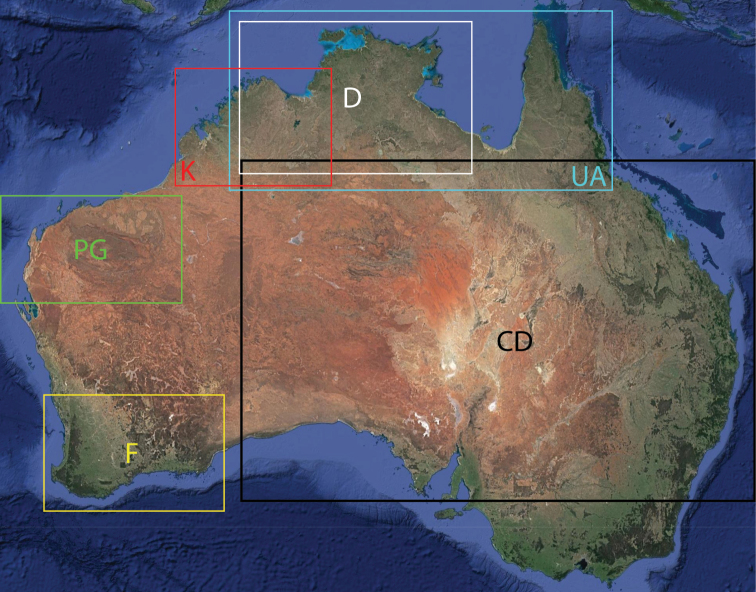
Map of Australia showing areas that are expanded in subsequent figures. CD = Central Desert group, D = *dawara* group, F = *francesae* group, K = Kimberley group, UA = species that are not assigned to a group, PG = Pilbara-Gascoyne group.

**Map 2.**
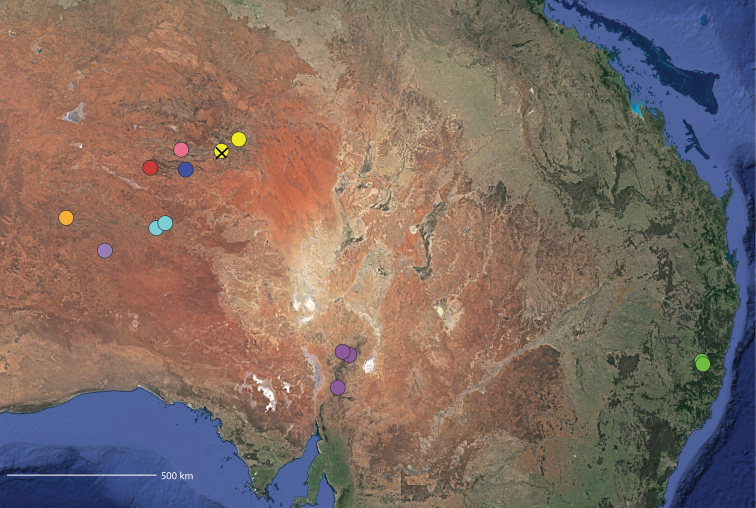
Species of the Central Desert species group; orange = juvenile from Morgan Range, light purple = *Karaopsdeserticola*, teal = *Karaopsngarutjaranya*, red = juveniles from Watarrka, blue = *Karaopslarapinta* sp. nov., pink = *Karaopskwartatuma* sp. nov., yellow = *Karaopspilkingtoni*, x = *Karaopsmparntwe* sp. nov., purple = *Karaopsvadlaadambara*, green = *Karaopsmanaayn*. Juveniles are treated as distinct species based on molecular data.

**Distribution.** Primarily found in the Central Desert region of the southern Northern Territory and northern South Australia; however, *Karaopsvadlaadambara* is from further east in the Gammon and Flinders Ranges, South Australia, and *K.manaayn* is known from northern New South Wales, near the Macleay River (Maps 1, 2).

### ﻿Key to the species of the Central Desert species group

**Table:** 

1	Females	**2**
–	Males	**8**
2(1)	Round accessory bulbs connected by a short duct to smaller spermathecae (Fig. 3B; Crews and Harvey 2011: figs 48, 54, 58, 60, 64)	**3**
–	Accessory bulbs more oval than round, nearly the same size as spermathecae (Fig. 3D, F)	***K.mparntwe* sp. nov.**
3(2)	Accessory bulbs separated by less than one accessory bulb diameter (Fig. 3B; Crews and Harvey 2011: fig. 58)	**4**
–	Accessory bulbs separated by at least one accessory bulb diameter (Crews and Harvey 2011: figs 54, 60, 64)	**5**
4(3)	Accessory bulbs much larger than spermathecae, abutting edge of epigynal plate laterally (Crews and Harvey 2011: fig. 58)	***K.pilkingtoni***
–	Accessory bulbs only slightly larger than spermathecae, not abutting edge of epigynal plate (Fig. 3B)	***K.larapinta* sp. nov.**
5(3)	Accessory bulbs and spermathecae nearly same distance from midline (Crews and Harvey 2011: figs 54, 60, 64)	**6**
–	Accessory bulbs closer to midline than spermathecae, known from only northern New South Wales (Crews and Harvey 2011: figs 47, 48)	***K.manaayn***
6(5)	Median lobe wider anteriorly than posteriorly (Crews and Harvey 2011: figs 59, 63)	**7**
–	Median lobe nearly the same width throughout its length (Crews and Harvey 2011: fig. 53)	***K.vadlaadambara***
7(6)	Median lobe extremely narrow, median field resembles a keyhole (Crews and Harvey 2011: fig. 63)	***K.ngarutjaranya***
–	Median lobe gradually narrows anteriorly to posteriorly (Crews and Harvey 2011: fig. 59)	***K.deserticola***
8(1)	One arm of median apophysis a stubby lobe (Fig. 4A, C; Crews and Harvey 2011: fig. 55)	**9**
–	Both arms of median apophysis long, well-defined (Crews and Harvey 2011 figs 51, 61)	**10**
9(8)	Conductor narrow, nearly straight retrolaterally, slightly curving to a point apically (Crews and Harvey 2011: fig. 55)	***K.pilkingtoni***
–	Conductor more pyramid shaped (Fig. 4A, C)	***K.kwartatuma* sp. nov.**
10(8)	Conductor sclerotized throughout (Crews and Harvey 2011: figs 51, 61)	**11**
–	Conductor mostly unsclerotized except for a pointed distal process; known from only northern New South Wales (Crews and Harvey 2011: fig. 49)	***K.manaayn***
11(10)	Conductor with a basal retrolateral lobe nearly covering distal arm of median apophysis (Crews and Harvey 2011: fig. 51)	***K.vadlaadambara***
–	Conductor and distal arm of median apophysis separated (Crews and Harvey 2011: fig. 61)	***K.ngarutjaranya***

#### The *strayamate* species group

**Diagnosis.** Females of the *strayamate* species group have copulatory ducts coiled multiple times into spirals. Males have small tegular lobes and no clear distinction between the tegular lobe and the lightly sclerotized median apophysis, and the embolus is very long and thin, following the perimeter of the cymbium.

**Composition.** This group comprises four species: *Karaopsstrayamate* sp. nov. (♀♂), *K.gangarie* Crews & Harvey, 2011 (♀♂), *K.monteithi* Crews & Harvey, 2011 (♀), and *K.ellenae* Crews & Harvey, 2011 (♀♂).

**Map 3.**
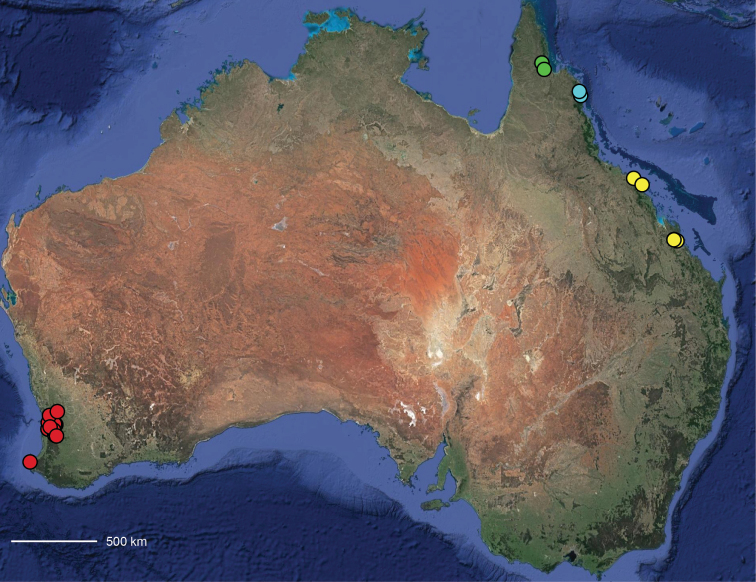
Species of the *Karaopsstrayamate* species group; red = *Karaopsellenae*, green = *Karaopsmonteithi*, light blue = *Karaopsgangarie*, yellow = *Karaopsstrayamate*.

**Distribution.** Northeastern Queensland, except for *Karaopsellenae*, known from southwestern Western Australia (Map 3).

### ﻿Key to the species of the *strayamate* species group

**Table:** 

1	Females	**2**
–	Males	**5**
2(1)	Accessory bulbs do not extend further anteriorly than copulatory ducts (Crews and Harvey 2011: figs 10, 12, 16 [fig. 10 labeled incorrectly: SD and FD are the copulatory ducts, SP is the accessory bulb, the posteriormost part of the illustration is the spermathecae which are connected to the fertilization ducts which are not illustrated here])	**3**
–	Accessory bulbs extend further anteriorly than copulatory ducts (Crews and Harvey 2011: fig. 76)	***K.ellenae***
3(2)	Lateral lobes distinct (Crews and Harvey 2011: figs 11, 15)	**4**
–	Lateral lobes indistinct, large posterior excavation to margin of epigynal plate (Crews and Harvey 2011: fig. 9)	***K.strayamate* sp. nov.**
4(3)	Lateral lobes frame a somewhat diamond-shaped median field (Crews and Harvey 2011: fig. 15)	***K.monteithi***
–	Lateral lobes only conspicuous posteriorly, do not frame the median field (Crews and Harvey 2011: fig. 11)	***K.gangarie***
5(1)	Embolus terminates at ~ 4 o’clock (Crews and Harvey 2011: figs 7, 13)	**6**
–	Embolus terminates at ~ 1 o’clock (Crews and Harvey 2011: fig. 73)	***K.ellenae***
6(5)	dRTA longer than vRTA in lateral view (Crews and Harvey 2011: fig. 8)	***K.strayamate* sp. nov.**
–	dRTA shorter or of equal length to vRTA in lateral view (Crews and Harvey 2011: fig. 14)	***K.gangarie***

#### The *raveni* species group

**Diagnosis.** Species of the *raveni* species group can be separated from other groups by extreme flatness, wide carapace, and the AER are nearly straight with the PER only slightly recurved. Additionally, the males have unpaired spines ventrally on Ta and Mt I and II.

**Composition.** The *raveni* species group comprises three species: *Karaopsraveni* Crews & Harvey, 2011(♀♂), *K.jarrit* Crews & Harvey, 2011 (♀♂), and *K.marrayagong* Crews & Harvey, 2011 (♀♂).

**Map 4.**
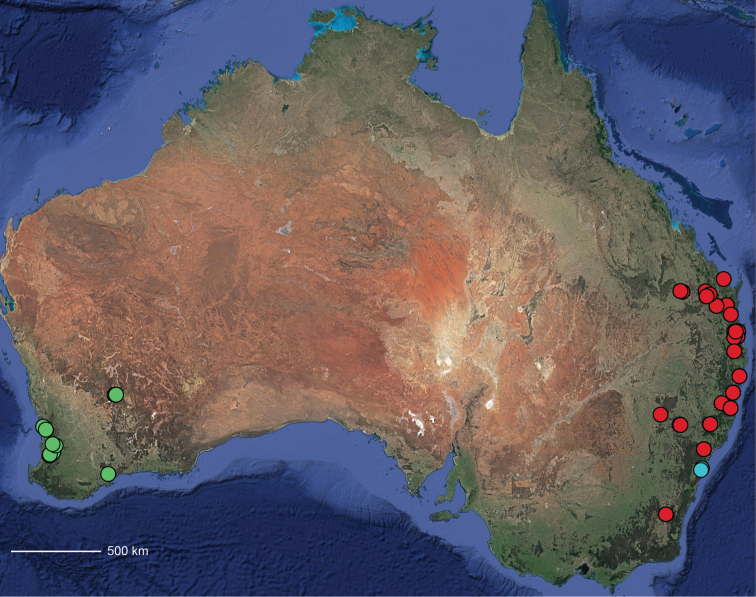
Species of the *Karaopsraveni* species group; green = *Karaopsjarrit*, red = *Karaopsraveni*, pale blue = *Karaopsmarrayagong*.

**Distribution.** New South Wales, north to southeastern Queensland. *Karaopsraveni* is one of the most widespread and well-collected *Karaops* species, occurring across the entire range of the species group (Map 4). *Karaopsmarrayagong* occurs within the distribution of *K.raveni* in the Sydney Basin of New South Wales. *Karaopsjarrit*, however, occurs in far southwestern Western Australia, nearly 3000 km away.

### ﻿Key to the species of the *raveni* species group

**Table:** 

1	Females	**2**
–	Males	**4**
2(1)	Accessory bulbs separated by more than one accessory bulb diameter (Crews and Harvey 2011: fig. 32)	***K.raveni***
–	Accessory bulbs separated by less than one accessory bulb diameter (Crews and Harvey 2011: figs 26, 28)	**3**
3(2)	Four promarginal teeth, known from only the Sydney Basin, New South Wales	***K.marrayagong***
–	Three^*^ promarginal teeth, known from only southwest Western Australia	***K.jarrit***
4(1)	The dRTA is at least two times longer than the vRTA (Crews and Harvey 2011: figs 29, 30)	***K.raveni***
–	The dRTA is not two times longer than vRTA (Figs 9E, F, 10B; Crews and Harvey 2011: figs 23, 24)	**5**
5(4)	The dRTA reaches branch of median apophysis in ventral and lateral views, known only from southwest Western Australia (Crews and Harvey 2011: figs 23, 24)	***K.jarrit***
–	The dRTA does not reach branch of median apophysis in ventral or lateral views, known from only the Sydney Basin, New South Wales (Figs 9E, F, 10B)	***K.marrayagong***

#### The *dawara* species group

**Diagnosis.** Females of the *dawara* species group have epigynal windows, and the copulatory ducts are unsclerotized at their origin and extend laterally from the windows. They are nearly invisible without staining. After coiling once, they narrow, are sclerotized, and tortuous. *Karaopsyumbu* is unique in having the median apophysis arise distally on an unsclerotized, retromedially-oriented extension of the tegulum (Crews 2013: figs 35, 36).

**Composition.** Four species are known from the *dawara* species group: *Karaopsdawara* Crews & Harvey, 2011 (♀), *K.nitmiluk* sp. nov. (♀), *K.jawayway* sp. nov. (♀), and *K.yumbu* Crews, 2013 (♂). *Karaopsyumbu* is the only known male of this group and is therefore not included in the key. Results of a molecular analysis indicate its placement in this group (Suppl. material 1).

**Map 5.**
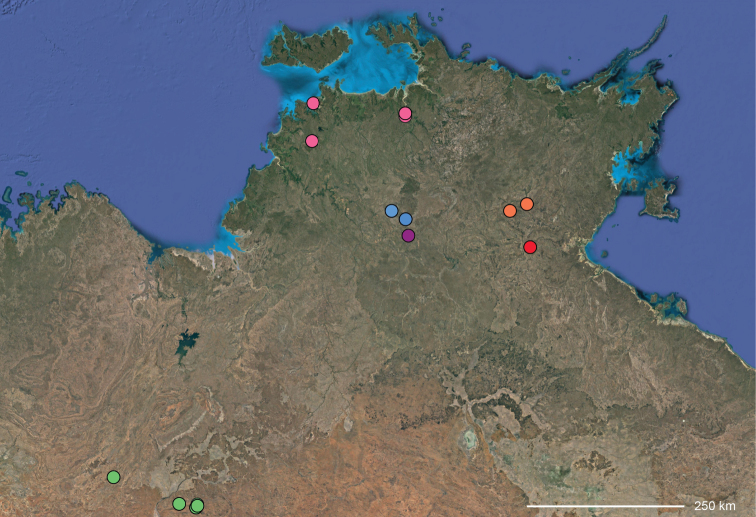
Species of the *Karaopsdawara* species group; green = *Karaopsyumbu*, pink = *Karaopsdawara*, blue = *Karaopsnitmiluk* sp. nov., purple = juvenile from Cutta Cutta Cave, orange = juvenile from Wongalara Wildlife Sanctuary, red = *Karaopsjawayway* sp. nov. Juveniles are treated as distinct species based on molecular data.

**Distribution.** The northern part of the Northern Territory, east to the Gulf of Carpentaria (Maps 1, 5).

**Discussion.** The females have somewhat odd genitalia in that the copulatory openings have not been located. The copulatory ducts connect directly to the windows (Figs 10F, G, 12A–D, 13C, D, 14C–F; Crews and Harvey 2011: figs 79, 80), but there is no visible opening. If there is an opening, it is very small and unable to be seen with a dissecting microscope in either stained or unstained specimens. Another place that seems likely for the copulatory openings is to the sides of the diamond-shaped or triangular part in the median field since there is a recess to the sides (Fig. 14C); however, this area has no openings either, unless they are so tiny that they cannot be seen. If there is an opening here, the sperm would be deposited into an open area surrounded by an unsclerotized, diamond-shaped structure that is connected to the anteromedial, lightly sclerotized area in between the windows, rather than connected to the windows or to the copulatory ducts. Perhaps the male punctures the epigynal windows to make contact with the copulatory ducts. *Karaopsyumbu*, which is the only know male from the group and is not paired with any of the females, does have a very different palp than any of the other *Karaops* species. In *K.dawara*, the accessory bulbs are at the end of the sclerotized part of the copulatory ducts and are easily seen in the two specimens that are known (Crews and Harvey 2011: fig. 80). In *K.nitmiluk* sp. nov. and *K.jawayway* sp. nov., the probable accessory bulbs are tiny and difficult to see from the dorsal or ventral views. The hypothesized accessory bulbs are indicated on Figs 10E and 14D. To confirm, the specimens would need further dissection as well as SEM imaging, and while there are four specimens of *K.nitmiluk* sp. nov., the genitalia all differ from each other, and there is only a single specimen of *K.jawayway* sp. nov., thus, due to rarity, no further actions have been made.

### ﻿Key to the females of the *dawara* species group

**Table:** 

1	Copulatory ducts wide, medial area where lateral lobes and windows abut diamond shaped (Figs 10D–G, 12A–D, 13C, D, 14C–F)	**2**
–	Copulatory ducts narrow, anterior margin of medial area where lateral lobes and windows abut shaped like an inverted triangle (Crews and Harvey 2011: figs 79, 80)	***K.dawara***
2(1)	Posteriorly, sclerotized part of copulatory ducts is closer to the lateral edges of epigynal plate than anteriorly (Fig. 14C–F)	***K.jawayway* sp. nov.**
–	Posteriorly, sclerotized part of copulatory ducts is the same distance from or further from lateral edges of epigynal plate than anteriorly (Figs 10D–G, 12A–D, 13C, D)	***K.nitmiluk* sp. nov.**

#### The *francesae* species group

**Diagnosis.** Species of the *francesae* group have more than three promarginal teeth and six pairs of ventral spines on Ti I and II and four pairs of ventral spines on Mt I and II. Females have epigynal pockets. Males have a two-armed median apophysis: the dorsal arm is sclerotized, the ventral arm is unsclerotized, and the dRTA is longer than the vRTA.

**Composition.***Karaopsfrancesae* Crews & Harvey, 2011 (♀♂) and *K.toolbrunup* Crews & Harvey, 2011 (♀♂).

**Map 6.**
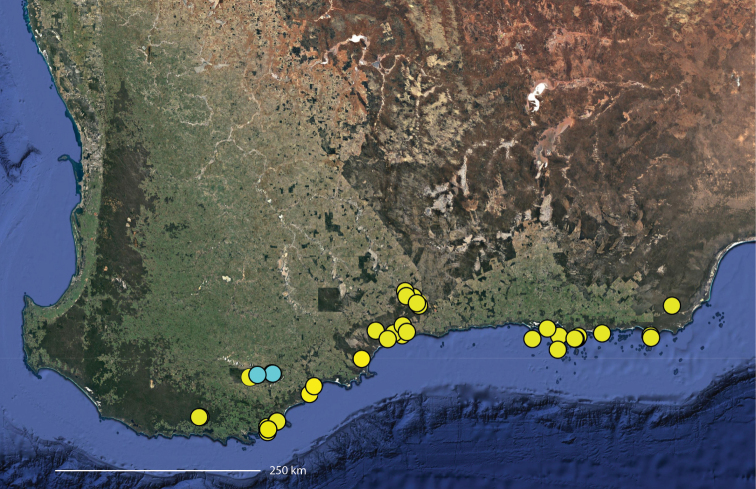
Species of the *Karaopsfrancesae* species group; yellow = *Karaopsfrancesae*, teal = *Karaopstoolbrunup*.

**Distribution.** Southwestern Western Australia. *Karaopstoolbrunup* occurs within the range of *K.francesae*, and molecular data indicate that there may be introgression; however, the two species can be reliably separated morphologically (Suppl. material 1, Maps 1, 6).

### ﻿Key to the species of the *francesae* species group

**Table:** 

1	Females	**2**
–	Males	**3**
2(1)	Copulatory ducts long and accessory bulbs clearly distinct, internal structures generally occupy more than 1/2 of epigynal plate length (Crews and Harvey 2011: figs 67, 68)	***K.francesae***
–	Copulatory ducts very short, internal structures occupy less than 1/2 of epigynal plate length (Crews and Harvey 2011: figs 69, 70)	***K.toolbrunup***
3(2)	Embolus follows perimeter of bulb (Crews and Harvey 2011: fig. 65)	***K.francesae***
–	Embolus does not reach the edge of bulb (Crews and Harvey 2011: fig. 71)	***K.toolbrunup***

#### The Kimberley species group

**Diagnosis.** Females of the Kimberley species group all have an atrium in the median field where the copulatory openings are located (Fig. 21A, B). They connect to two large copulatory ducts leading to the accessory bulb and spermathecae (Fig. 21B). The lateral lobes are separated posteriorly. Males of the Kimberley species group have a cymbium length to palpal tibial length ratio of > 0.60. The conductor is generally crescent shaped, with differences between species manifesting in how far the apical part of the conductor extends retrolaterally (or the size of the indentation between the apical and middle parts) and the shape of the middle part (Fig. 22A). In general, all the species in the group look very much alike, and it was surprising to find there are so many different species supported by both morphological and molecular data (Suppl. material 1). They have all been collected under rocks except for a single specimen on a tree (Figs 18H, 24E, 27C). Although many are known by a single sex, molecular data do not indicate that any of the single males and females are conspecific (Suppl. material 1). There are undescribed species known (Figs 17A–D, 18A, 19C, E, 20B, D, E, 26D), and given the proximity of different species, there are likely many more on the islands and in the carved-out landscape of gorges and ranges in the Kimberley (Figs 18B, 19F, 24C, 27D).

**Composition.** Ten species: *Karaopsjenniferae* Crews & Harvey, 2011 (♀), *K.alanlongbottomi* Crews & Harvey, 2011 (♂), *K.conilurus* sp. nov. (♂), *K.larryoo* Crews & Harvey, 2011 (♂), *K.dalmanyi* sp. nov. (♀♂), *K.keithlongbottomi* Crews & Harvey, 2011 (♂), *K.garyodwyeri* sp. nov. (♂), *K.dejongi* sp. nov. (♀♂), *K.malumbu* sp. nov. (♀♂), *K.umiida* Crews, 2013 (♀).

**Map 7.**
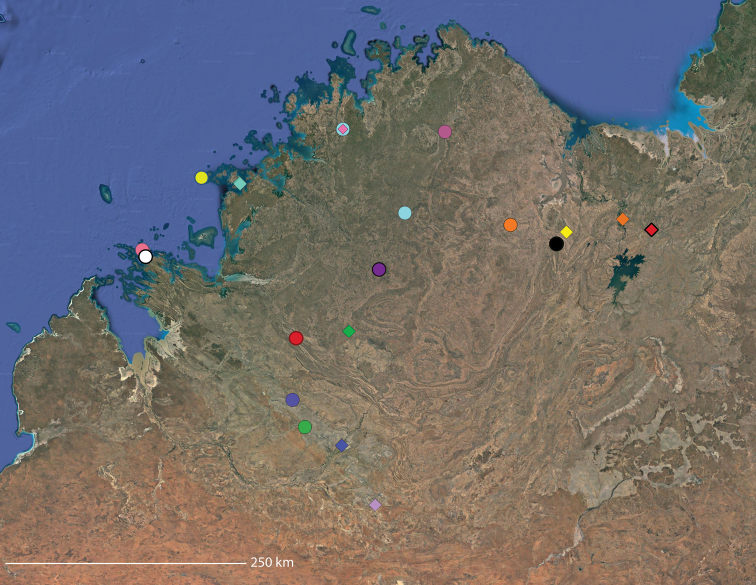
Species of the Kimberley species group. Diamonds (juveniles): light purple = Mimbi Caves; blue = Geike Gorge, green = Adcock Gorge, teal = Augustus Island, pink = Little Mertens Falls, yellow = Emma Gorge, orange = Kelly’s Knob, red = Keep River National Park; Circles: green = *Karaopsdejongi* sp. nov., blue = *Karaopsjenniferae*, red = *Karaopsdalmanyi* sp. nov., purple = *Karaops* sp. adult male (will be described in subsequent publication), white = *Karaopsconilurus* sp. nov., pink = *Karaopsumiida*, yellow = *Karaopsalanlongbottomi*, teal = *Karaopskeithlongbottomi*, mauve = *Karaopslarryoo*, orange = *Karaopsgaryodwyeri* sp. nov., black = *Karaopsmalumbu* sp. nov. Juveniles are treated as distinct species based on molecular data.

**Distribution.** Throughout the Kimberley region, Western Australia, including the islands (Maps 1, 7).

### ﻿Key to the species of the Kimberley species group

**Table:** 

1	Females	**2**
–	Males	**6**
2(1)	Accessory bulbs posterior to or almost even with anterior part of atrium (Figs 21A–D, 23A, B, 30B, C; Crews 2013: figs 1, 2)	**3**
–	Accessory bulbs conspicuously anterior to anterior part of atrium (Fig. 28F, G)	***K.jenniferae***
3(2)	Sclerotized part of lateral lobes around atrium not u-shaped (Figs 21A, C, 23A, 30B)	**4**
–	Sclerotized part of lateral lobes around atrium u-shaped (Crews 2013: figs 1, 2)	***K.umiida***
4(3)	Sclerotized part of lateral lobes around atrium not forming a shape like a pendant droplet (Figs 21A, C, 23A)	**5**
–	Sclerotized part of lateral lobes around atrium shaped like a pendant droplet (Fig. 30C)	***K.malumbu* sp. nov.**
5(4)	Atrium leading to copulatory ducts more or less parallel sided (Fig. 21B, D)	***K.dejongi* sp. nov.**
–	Atrium leading to copulatory ducts narrowed then widened toward the posterior (Fig. 23B)	***K.dalmanyi* sp. nov.**
6(1)	Apical portion of conductor retrolaterally extends beyond medial part of the conductor (Figs 19A, 26B, 27E; Crews and Harvey 2011: fig. 17)	**7**
–	Apical portion of conductor does not retrolaterally extend beyond medial part of the conductor (Figs 22A, C, 31D, 32A; Crews and Harvey 2011: fig. 21; Crews 2013: fig. 3)	**9**
7(6)	Apical portion of conductor retrolaterally extends to edge of bulb or beyond (Figs 26B, 27E; Crews and Harvey 2011: fig. 17)	**8**
–	Apical portion of conductor retrolaterally does not extend to edge of bulb, large *Karaops* (Figs 18I, 19A)	***K.keithlongbottomi***
8(7)	Tegular lobe tiny, narrowed to embolus at ~ 5 o’clock, embolus follows cymbium perimeter (Crews and Harvey 2011: fig. 17)	***K.alanlongbottomi***
–	Tegular lobe not tiny, narrowed to embolus at ~ 6 o’clock, embolus closer to center of bulb, palpal tibia exceptionally long (Fig. 27E, F)	***K.dalmanyi* sp. nov.**
9(6)	C-shaped indentation between apical and medial part of conductor (Figs 22A, C, 31D, 32A; Crews 2013: fig. 3)	**10**
–	No indentation between apical and medial part of conductor (Crews and Harvey 2011: fig. 21)	***K.larryoo***
10)	Medial part of conductor curved on retrolateral side (Figs 31D, 32A; Crews 2013: fig. 3)	**11**
–	Medial part of conductor straight on retrolateral side, squarish (Figs 22A, C)	***K.dejongi* sp. nov.**
11(10)	Conductor curved along top (Fig. 31D; Crews 2013: fig. 3)	**12**
–	Conductor flat along the top, spinules on base of median apophysis, small *Karaops* (Fig. 32A)	***K.malumbu* sp. nov.**
12(11)	Medial part of conductor sinuous on retrolateral side, indentation between apical and middle portion small, spermophor curves do not touch (Fig. 31D)	***K.garyodwyeri* sp. nov.**
–	Medial part of conductor curved on retrolateral side, indentation between apical and middle portion larger, spermophor curves abut one another (Crews 2013: fig. 3)	***K.conilurus* sp. nov.**

#### The Pilbara-Gascoyne species group

**Diagnosis.** Several species have small accessory bulbs located on the copulatory ducts, and the spermathecae are allantoid and much larger than the accessory bulbs (figs 6, 8, 10, 26, 32 in Crews 2013; Figs 49C–F, 51C–F, 53B, C, 55B–E, 59B, C, 60C, D). If the accessory bulbs are equal to or larger than the spermathecae, and both are relatively large, the copulatory openings may be in an m-shaped opening (Crews and Harvey 2011: fig. 37; Crews 2013: fig. 33). If the accessory bulbs and spermathecae are both small, the copulatory ducts are long (Crews 2013: figs 14, 16).

The males have large, elaborate conductors that are often coiled or twisted and extend ventrally beyond the cymbium (Figs 50C, D, 56A, B, 58A, E, 60A, B, 63C, C, 65A, B, 69 A, C; Crews 2013: figs 11, 12, 17–22, 23, 27–30). They all have five pairs of ventral spines on Ti I and II and four pairs on Mt I and II.

The Pilbara, especially the Chichester subregion, is very diverse and has many endemic species of several taxa (e.g., schizomids [Abrams et al. 2019; Abrams et al. 2020]), including *Karaops*, with at least ten endemics. One species, *K.nyiyaparli*, is widespread in the area, whereas nearly all of the others have been collected only once. In the Hamersley subregion, there are at least five species, but they do not appear to have broad areas of overlap (Maps 1, 9A, B). The Gascoyne has not been explored as much as the Pilbara, so there are large gaps.

**Composition.** Eighteen species: *Karaopsmartamarta* Crews & Harvey, 2011 (♀♂), *K.nyangumarta* Crews, 2013 (♀♂), *K.nyamal* Crews, 2013 (♀), *K.joehaeneri* sp. nov. (♀♂), *K.morganoconnelli* sp. nov. (♀♂), *K.burbidgei* Crews & Harvey, 2011(♀♂), *K.karrawarla* Crews & Harvey, 2011(♀♂), *K.badgeradda* Crews & Harvey, 2011(♀), *K.julianneae* Crews & Harvey, 2011 (♀), *K.durrantorum* sp. nov. (♂), *K.banyjima* Crews, 2013 (♀♂), *K.kariyarra* Crews, 2013 (♀), *K.nyiyaparli* Crews, 2013(♀), *K.yurlburr* Crews, 2013 (♀♂), *K.feedtime* Crews, 2013(♀), *K.forteyi* Crews, 2013 (♀♂), *K.jaburrara* Crews, 2013 (♂), *K.ngarluma* Crews, 2013(♂).

**Distribution.** Throughout the Gascoyne and Pilbara of Western Australia (Maps 1, 9A, B).

### ﻿Key to the species of the Pilbara-Gascoyne species group

**Table:** 

1	Females	**2**
–	Males	**16**
2(1)	Copulatory ducts less sclerotized at origin than rest of duct (Figs 49D, F, 51D, F, 53C, 55C, E, 59C, 60D; Crews and Harvey 2011: figs 34, 42, 44, 46; Crews 2013: figs 26, 32)	**3**
–	Copulatory ducts otherwise (Crews and Harvey 2011: figs 38; Crews 2013: figs 6, 8, 10, 14, 16, 34)	**10**
3(2)	Copulatory openings beneath m-shaped hoods or margins (Figs 49C, E, 53B, 55B, D, 59B, 60C; Crews and Harvey 2011: figs 33, 41, 45; Crews 2013: figs 25, 31)	**4**
–	Copulatory openings beneath rounded margin (Fig. 51C, E; Crews and Harvey 2011: fig. 43)	***K.julianneae***
4(3)	Accessory bulbs anterior to copulatory openings (Figs 49D, F, 53C, 55E, 59C, 60D; Crews and Harvey 2011: figs 34, 42, 46; Crews 2013: figs 26, 32)	**5**
–	Accessory bulbs not anterior to copulatory openings (Fig. 55C)	***K.joehaeneri* sp. nov.**
5(4)	Copulatory openings posterior to anteriormost part of spermathecae (Fig. 53C; Crews and Harvey 2011: fig. 34; Crews 2013: fig. 26)	**6**
–	Copulatory openings anterior to or even with anteriormost part of spermathecae (Figs 49D, F, 55E, 59C, 60D; Crews and Harvey 2011: figs 42, 46; Crews 2013: fig. 32)	**7**
6(5)	Copulatory ducts extend well beyond spermathecae (Fig. 53C; Crews and Harvey 2011: fig. 34)	***K.badgeradda***
–	Copulatory ducts do not extend beyond spermathecae (Crews 2013: fig. 26)	***K.nyangumarta***
7(5)	Accessory bulbs located on copulatory ducts after first curve (Figs 49D, F, 55C, E, 59C; Crews and Harvey 2011: figs 42, 46)	**8**
–	Accessory bulbs located at top of first curve of copulatory ducts (Fig. 60D; Crews 2013: fig. 32)	***K.nyamal***
8(7)	At closest, copulatory ducts separated no more than 1 diameter of anteriormost part of spermathecae (Figs 49D, F, 59C; Crews and Harvey 2011: fig. 46)	**9**
–	At closest, copulatory ducts clearly separated by more than 1 diameter of anteriormost part of spermathecae (Fig. 55G; Crews and Harvey 2011: fig. 42	***K.karrawarla***
9(8)	Copulatory openings located in depression with an arch-shaped anterior margin; first curve of copulatory ducts with space between curve (Fig. 9C)	***K.morganoconnelli* sp. nov**.
–	No arch-shaped anterior margin; first curve of copulatory ducts tight with very little space between curve (Fig. 49D, F; Crews and Harvey 2011: fig. 46)	***K.martamarta***
10(2)	Copulatory openings beneath m-shaped hood or margin (Crews and Harvey 2011: fig. 38; Crews 2013: figs 7, 33)	**11**
–	Copulatory openings not located beneath m-shaped hood or margin (Crews 2013: figs 5, 9, 13, 15)	**13**
11(10)	Copulatory openings in depression with m-shaped anterior margin (Crews 2013: figs 7, 33)	**12**
–	Copulatory openings beneath m-shaped hoods (Crews and Harvey 2011: fig. 37)	***K.burbidgei***
12(11)	Copulatory openings in center of median field in heart-shaped depression (Crews 2013: fig. 7)	***K.kariyarra***
–	Copulatory openings in somewhat lemniscate depression in posterior of median field	***K.banyjima***
13(10)	Median field with depression (Crews 2013: figs 5, 9, 15)	**14**
–	Median field with large lobe (Crews 2013: fig. 13)	***K.feedtime***
14(13)	Lateral lobes distinct (Crews 2013: figs 5, 9)	**15**
–	Lateral lobes indistinct, median field depression oval (Crews 2013: fig. 15)	***K.forteyi***
15(13)	Copulatory openings close together in center of median field (Crews 2013: fig. 15)	***K.nyiyaparli***
–	Copulatory openings distant from one another at lateral edges of median field (Crews 2013: fig. 9)	***K.yurlburr***
16(1)	dRTA much longer than vRTA (Crews 2013: figs 11, 12, 17, 18, 21–24)	**17**
–	dRTA shorter than or not much longer than vRTA (Figs 50C, D, 56A, B, 58A, E, 60A, B, 63C, D, 65A, B, 69A–C; Crews and Harvey 2011: figs 35, 36; Crews 2013: figs 19, 20)	**20**
17(16)	With keel between vRTA and dRTA (Crews 2013: figs 11, 12, 23, 24)	**18**
–	Without keel between vRTA and dRTA (Crews 2013: figs 17, 18, 21, 22)	**19**
18(17)	In retrolateral view dRTA bent ventrally nearly 90° (Crews 2013: fig. 12)	***K.yurlburr***
–	In retrolateral view dRTA directed apically (Crews 2013: fig. 24)	***K.ngarluma***
19(17)	dRTA toothed along apical margin (Crews 2013: fig. 21)	***K.jaburrara***
–	dRTA not toothed along apical margin, (Crews 2013: fig. 17)	***K.forteyi***
20(16)	Embolus close to bulb perimeter, broadly curved (Figs 50C, 56A, 58A, 60A, 63C, 65A, 69A, B)	**21**
–	Embolus short, closer to center of bulb, directed apically, small hook (Crews 2013: fig. 19)	***K.nyiyaparli***
21(20)	Tegular lobe large, covering most of the cymbium basally, lobe narrowed to embolus from 7–9 o’clock (Figs 50C, 63C, 65A, 69A, B; Crews and Harvey 2011: fig. 35)	**22**
–	Tegular lobe mostly on retrolateral side of palp, narrowed to embolus at ~ 6 o’clock (Figs 56A, 58A, 60A)	**26**
22(21)	Median apophysis broad, tongue-like (Figs 50C, 69A, B)	**23**
–	Median apophysis narrowed abruptly (Figs 63C, 65A; Crews and Harvey 2011: fig. 35)	**24**
23(22)	dRTA longer than palpal tibia, widened distally, truncate, longer than vRTA (Fig. 50C, D)	***K.martamarta***
–	dRTA shorter than or of equal length to palpal tibia, uniform width throughout length, pointed distally, not longer than vRTA (Fig. 69A–C)	***K.banyjima***
24(22)	Spermophor abuts bottom of tegular lobe (Figs 63C, 65A)	**25**
–	Spermophor does not abut bottom of tegular lobe (Crews and Harvey 2011: fig. 35)	***K.burbidgei***
25(24)	Spermophor very wide in tegular lobe, part of conductor unpigmented, large retrobasal cymbial process (Fig. 63C)	***K.nyangumarta***
–	Spermophor narrow in tegular lobe, conductor of uniform pigmentation, retrobasal cymbial process present, but not exceptionally large (Fig. 65A)	***K.durrantorum* sp. nov.**
26(21)	Embolus comes to a point at tip (Figs 56E, 60E)	**27**
–	Embolus slightly broadened into a diamond shape at tip (Fig. 58D–F)	***K.karrawarla***
27	When palp is expanded, median apophysis directed basally, conductor not hammer shaped (Fig. 60E)	***K.morganoconnelli* sp. nov.**
–	When palp is expanded, median apophysis directed retrolaterally, conductor hammer shaped (Fig. 56E)	***K.joehaeneri* sp. nov.**

## ﻿Diagnoses and descriptions

### The Central Desert species group

#### ﻿ Karaops ngarutjaranya

Taxon classificationAnimaliaAraneaeSelenopidae

Crews & Harvey, 2011

C5712441-4D47-5E2E-9338-97A8C364925D

**Figure 1.**
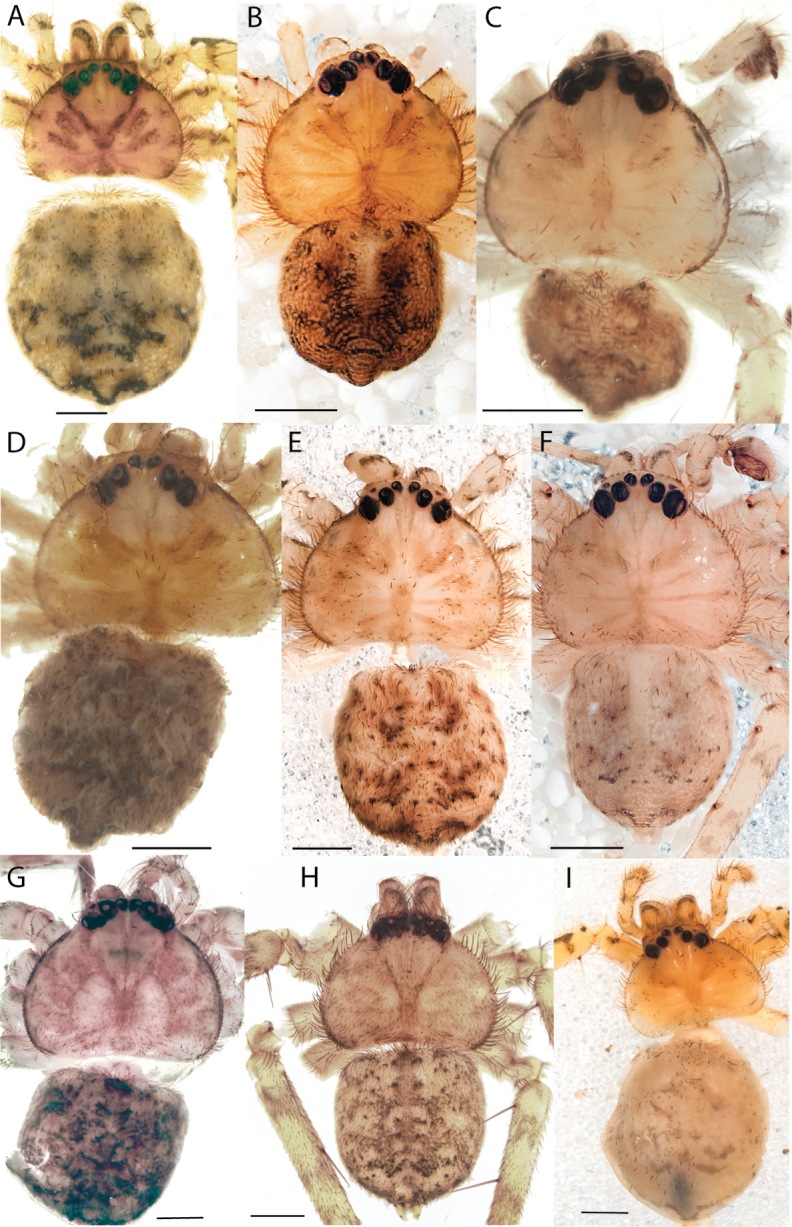
Species of the Central Desert species group **A***Karaopsngarutjaranya*, female paratype, southeast of Womikata Bore Homeland, South Australia (SAM NN10915) **B***Karaopsngarutjaranya*, male holotype, northeast of Mount Woodroffe, South Australia (SAM NN10914) **C***Karaopspilkingtoni*, male holotype, Trig Hill (but see text), Old Telegraph Station, Alice Springs, Northern Territory (WAM T76590) **D***Karaopspilkingtoni*, female paratype, Alice Springs, Northern Territory (SAM N199359) **E***Karaopsvadlaadambara*, female paratype, Arcoona Creek, near Sambot Waterhole, Gammon Ranges National Park, South Australia (SAM N199354) **F***Karaopsvadlaadambara*, male holotype, Arcoona Creek, near Sambot Waterhole, Gammon Ranges National Park, South Australia (SAM N199353) **G***Karaopsmanaayn*, female holotype, Kempsey Road, West Armidale, above Macleay River, New South Wales (AM KS043756) **H***Karaopsmanaayn*, male paratype, Kempsey Road, West Armidale, above Macleay River, New South Wales (AM KS113351) **I***Karaopsmparntwe* sp. nov., holotype female, Alice Springs, Northern Territory (AMT AA 10.852). Scale bars: 1 mm.

Fig. 1A, B , Maps 1 , 2

Karaops ngarutjaranya Crews & Harvey, 2011: 68, figs 61–64 (♂, ♀, examined).

##### Diagnosis.

The female of *Karaopsngarutjaranya* (Fig. 1A, B) can be separated from other members of the Central Desert group by the lateral lobes nearly touching toward the posterior of the epigynal plate, and the median field is shaped like a keyhole (Crews and Harvey 2011: fig. 59). The conductor of the male has a sinuous margin, and the median apophysis does not cover part of the conductor (Crews and Harvey 2011: figs 61, 63).

##### Description.

The description of the male and female can be found in Crews and Harvey (2011).

##### Distribution.

Known only from two nearby localities, Mount Woodroffe and Womikata Bore Homeland, South Australia (Map 2).

##### Natural history.

*Karaopsngarutjaranya* occurs in the Mann-Musgrave Block subregion of the Central Ranges in northern South Australia (Maps 1, 2; Suppl. material 2: table S1). The ranges have wattle scrub or *Callitrisglaucophylla* F. Muell woodlands over grasslands, and low, open woodlands of ironwood and corkwood over grasses on the edges of the ranges (Graham and Cowan 2001). The male was collected in a gorge.

##### Discussion.

*Karaopsngarutjaranya* is only known from one male (Fig. 1B) and one female (Fig. 1A) collected from different but nearby localities a few days apart. It is mentioned that the Mann-Musgrave block subregion is rich and diverse but that many species have broad ranges and occur in several adjoining subregions (Graham and Cowan 2001). There have been no collections made in the immediate subregions, but the ones nearby harbor different, though closely related, species. The spiders were collected in October, a hot, wetter part of the year.

#### ﻿ Karaops pilkingtoni

Taxon classificationAnimaliaAraneaeSelenopidae

Crews & Harvey, 2011

AA6D336C-EF3A-5DCE-B6F5-B51065ACA9AF

Fig. 1C, D , Maps 1 , 2

Karaops pilkingtoni Crews & Harvey, 2011: 64, figs 55–58 (♂, ♀, examined).

##### Diagnosis.

*Karaopspilkingtoni* (Fig. 1C, D) can be differentiated from almost all other members of the Central Desert species group by the embolus which is very short and in the middle of the bulb, rather than hooked and closer to the perimeter (Crews and Harvey 2011: fig. 55). The differences between this species and *K.kwartatuma* sp. nov. are given below in the diagnosis of the new species. The female has very large, round accessory bulbs that nearly come into contact at the midline and are much bigger than the spermathecae, not found in other group members (Crews and Harvey 2011: fig. 8).

##### Description.

The description of the male and female can be found in Crews and Harvey (2011).

##### Distribution.

The male and female are known only from the vicinity of Alice Springs, Northern Territory (Map 2).

##### Natural history.

The female (Fig. 1D) and the coordinates of the male (Fig. 1C) occur in the Hartz Range subregion of the MacDonnell Ranges. Trig Hill occurs in the MacDonnell subregion of the MacDonnell Ranges, though on the border of the Hartz Range subregion. The collections were made in May and June, a cooler, drier time of the year, transitioning from hotter and wetter (Suppl. material 2: table S1). The female was collected beneath rocks.

##### Discussion.

The holotype male of *Karaopspilkingtoni* (Fig. 1C) is from the Telegraph Station at Alice Springs. Despite looking around Trig Hill in Alice Springs, no selenopids were found. There is some confusion, though, because the latitude and longitude given on the label do not correspond to the locality given on the label, Trig Hill. The female (Fig. 1D) was matched with the male (Fig. 1C) based on the locality of the collections. With new data regarding the number of species that can occur in an area, it may be that this female is another species. For example, *K.mparntwe* sp. nov. is known from “Alice Springs”, and that is all of the information provided for this specimen. Only additional collecting and molecular data will help determine species boundaries, and no taxonomic changes are made at this time.

#### ﻿ Karaops vadlaadambara

Taxon classificationAnimaliaAraneaeSelenopidae

Crews & Harvey, 2011

FF3D276B-F35B-5933-97F1-80E5A4789E00

Fig. 1E, F , Maps 1 , 2

Karaops vadlaadambara Crews & Harvey, 2011: 59, figs 51–54 (♂, ♀, examined).

##### Diagnosis.

The male of *Karaopsvadlaadambara* (Fig. 1F) has a darkened tip of the conductor and a basal extension of the conductor that nearly covers the distal branch of the median apophysis (Crews and Harvey 2011: fig. 51). The median lobe of the female is nearly uniform in width throughout its length and the spermathecae and accessory bulbs form a straight vertical line (Crews and Harvey 2011: fig. 53). In *K.manaayn*, they are angled outward (Crews and Harvey 2011: figs 47, 48).

##### Description.

The description of the male and female can be found in Crews and Harvey (2011).

##### Distribution.

This species is known only from the Gammon and Flinders Ranges, South Australia (Map 2).

##### Natural history.

The climate of the collection localities is semiarid to arid with erratic rainfall. Females and males have been collected in cooler, slightly wetter times of the year, with one male collected at a warmer, wetter time of year (Suppl. material 2: table S1). At least one specimen was collected at night and another in a pitfall trap.

##### Discussion.

*Karaopsvadlaadambara* (Fig. 1E, F) is the only species of the Central Desert species group known from more than one or two specimens (three males and six females). It also does not technically occur in the Central Desert, but it is part of the Eyrean or Eastern Desert region of Ebach et al. (2013), or the Northern and Central Flinders subregions of the Flinders Lofty Block in the IBRA (Maps 1, 2).

#### ﻿ Karaops manaayn

Taxon classificationAnimaliaAraneaeSelenopidae

Crews & Harvey, 2011

8459675F-432A-56FA-A7D0-ECD4E4A5DC23

Fig. 1G, H , Maps 1 , 2

Karaops manaayn Crews & Harvey, 2011: 57, figs 47–50, 96 (♂, ♀, examined).

##### New record.

New South Wales • 1♀; Oxley Wild Rivers National Park, Yarrowitch River; -31.0759, 152.0563; 12 Nov. 2015; H.M. Smith leg.; (AM KS.124572).

##### Diagnosis.

The spermathecae of *Karaopsmanaayn* are further from the midline than are the accessory bulbs (Crews and Harvey 2011: fig. 48). The male has a sclerotized, quadrangular process on the conductor (Crews and Harvey 2011: fig. 49).

##### Description.

The description of the male and female can be found in Crews and Harvey (2011).

##### Distribution.

*Karaopsmanaayn* (Fig. 1G, H) is found only in a small area near the Macleay and Yarrowitch Rivers, New South Wales (Map 2).

##### Natural history.

This species has been collected in two adjacent subregions: the Macleay Hastings and Coffs Coast and Escarpment subregions of the New South Wales North Coast bioregion. While climate within the entire region ranges from subtropical to subhumid, the climate in the immediate area around Armidale is somewhat temperate (NSW National Parks and Wildlife Service (2003)) (Suppl. material 2: table S1). This species has been collected on a rock wall above a river and beneath bark.

##### Discussion.

*Karaopsmanaayn* (Fig. 1G, H) is known only from a small area in northeastern New South Wales, within the range of *K.raveni*, with which it has been collected (Maps 1, 2, 4). Another species, *K.marrayagong*, also occurs within the range of *K.raveni*, but it is in the *raveni* group. Molecular data has placed this species with members of the Central Desert Species group (Suppl. material 1). Although its placement with the group is stable, depending on the analysis, it is unstable within the group (unpubl. data). Morphologically, it also shares similarities with members of this group, such as the large median lobe of the epigyne, and the accessory bulb and spermathecae are connected by a duct with a loop (Crews and Harvey 2011: figs 47, 48), and the male has a two-branched median apophysis (Crews and Harvey 2011: fig. 49).

#### ﻿ Karaops deserticola

Taxon classificationAnimaliaAraneaeSelenopidae

Crews & Harvey, 2011

BE5C3BD2-95BB-567F-806C-31F6ABF5499C

**Figure 2.**
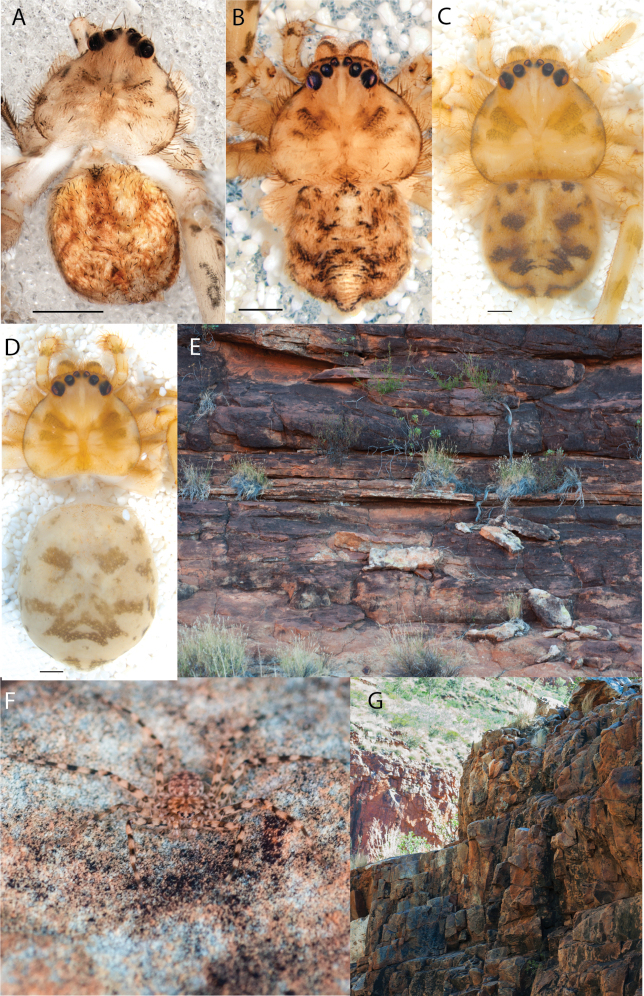
Species and habitats of the Central Desert species group **A***Karaopskwartatuma* sp. nov., holotype male, Ormiston Gorge, West MacDonnell Range National Park, Northern Territory (sel_1093, MAGNT A004858) **B***Karaopsdeserticola*, female holotype, Mount Lindsay, South Australia (SAM N199350) **C***Karaopslarapinta* sp. nov., holotype female, Finke River, Northern Territory (QMS 110852) **D***Karaopslarapinta* sp. nov., paratype female, Finke River, Northern Territory (QMS 110852) **E** Habitat of *Karaops* sp. from Watarrka National Park, Northern Territory **F***Karaops* sp., King’s Canyon Rim Walk, Watarrka National Park, Northern Territory **G** habitat of *Karaopskwartatuma* sp. nov., Ormiston Gorge, Northern Territory, Australia. Scale bars: 1 mm.

Fig. 2B , Maps 1 , 2

Karaops deserticola Crews & Harvey, 2011: 66, figs 59, 60 (♀, examined).

##### Diagnosis.

The median lobe of the epigyne gradually narrows distally unlike other species of the group (Crews and Harvey 2011: fig. 59).

##### Description.

The description of the female can be found in Crews and Harvey (2011).

**Male.** Unknown.

##### Distribution.

*Karaopsdeserticola* is only known from Mount Lindsay, South Australia (Map 2).

##### Natural history.

*Karaopsdeserticola* (Fig. 2B) has been collected in the Watarru subregion, a relatively small subregion, surrounded by the Kintore subregion of the Great Victoria Desert bioregion. The lone collection was made in August, a cooler, drier month of the year. The area is a relatively undisturbed wilderness of arid and semi-arid shrubland and grassland (Suppl. material 2: table S1). This species was collected under a rock slab on a bare granite slope.

##### Discussion.

This species is known from a single female specimen collected in 1980 (Fig. 2B). The locality is found in the Watarru subregion of the Central Ranges bioregion. This subregion is the home of the Watarru Indigenous Protected Area (Taylor Burrell Barnett Town Planning and Design 2007).

#### ﻿ Karaops kwartatuma sp. nov.

Taxon classificationAnimaliaAraneaeSelenopidae

46A9AF0D-4B9A-52ED-8446-15790D90358A

https://zoobank.org/5BDAB028-E2ED-4A26-BA4B-7DFA4943EAB8

Figs 2A, G , 4A–D , 5A, B , Maps 1 , 2

##### Material examined.

***Holotype***: Northern Territory • ♂ (reared in captivity); West MacDonnell Range National Park, Ormiston Gorge; 23°37.718'S, 132°43.662'E; ~ 674 m; 19 Apr. 2016; S. Crews leg.; in rock cracks on cliff face above waterhole; sel_1093; SCC16_012; (MAGNT A004858).

##### Diagnosis.

*Karaopskwartatuma* sp. nov. (Fig. 2A) is similar to the other members of the Central Desert species group, *K.vadlaadambara*, *K.ngarutjaranya*, and *K.pilkingtoni*, by having a short embolus that is nearer to the middle of the bulb rather than the cymbial margin and by having spinules on the median apophysis (although this was not noted for the other species in Crews and Harvey 2011) (Fig. 4A, C). The new species can be distinguished from *K.vadlaadambara* and *K.ngarutjaranya* by the RTA and median apophysis. The dRTA of the latter two species is directed away from the palpal tibia at a nearly 90° angle in ventral view, and both branches of the median apophysis are long. In the new species, the dRTA is not directed away from the palpal tibia at a nearly 90° angle in ventral view, and the two-branched median apophysis has a long branch and a smaller, broad, unsclerotized, inconspicuous lobe (Fig. 4A, C; Crews and Harvey 2011: figs 51, 52, 61, 62). *Karaopskwartatuma* sp. nov. can be differentiated from *K.pilkingtoni* by the RTA, the median apophysis, the conductor, and the embolus. In lateral view, the dRTA of the new species is narrow, very slightly curved ventrally, and pointed distally (Fig. 4D). In *K.pilkingtoni*, the dRTA is squared off distally (Crews and Harvey 2011: fig. 56). The conductor of *K.pilkingtoni* is somewhat narrow and longitudinal, arising medially and extended to the anterior edge of the bulb (Crews and Harvey 2011: fig. 55) ending in a point. In the new species, the conductor is broader, with the point directed ventrally and is somewhat pyramidal (Fig. 4A, C). Finally, the embolus remains broad throughout its length in *K.pilkingtoni* (Crews and Harvey 2011: fig. 55), but it narrows slightly in *K.kwartatuma* sp. nov. (Fig. 4A, C).

##### Description.

**Male** (holotype). Total length 3.34. Carapace: length 1.47, width 1.87. Chelicerae: promargin with three teeth, retromargin with two teeth, robust. Eyes: AER slightly recurved, PER recurved; diameters AME 0.10, ALE 0.06, PME 0.11, PLE 0.19; interdistances AME–PME 0.02, PME–ALE 0.09, ALE–PLE 0.11, PME–PME 0.60, ALE–ALE 0.83, AME–AME 0.38, PLE–PLE 1.09. Sternum: length 0.95, width 0.99. Abdomen: length 1.87, width 1.64. Color (in life Fig. 5A/preserved Fig. 2A): Carapace: yellowish brown with darker marks extended toward but not reaching margin, three dark marks at lateral edges, setose with slender setae and sparse, thick, stubby setae/pale yellowish to brown, dark marks visible, less distinct than in life. Chelicerae: pale yellowish with conspicuous, black, crescent-shaped marks, sort of like a little mustache (Fig. 5B), setae uniform. Maxillae: pale yellowish brown. Labium: brown, paler distally. Sternum: yellowish. Abdomen: dorsally golden brown, darker medially, chevron medially, sides of chevron angled anteriorly, darker posteriorly, laterally spotted/golden parts yellowish, darker parts orange-red, pattern inconspicuous, dark, thick, stubby setae giving abdomen spotted appearance from a distance; ventrally yellowish white. Legs: golden brown, Cx and Tr with dark prolateral lines, dot at Tr-Fm joint, Fm basally with two dark lines that do not completely encircle legs, not pigmented centrally, another ring medially, another distally, Pt with dark annulation basally, Ti with dark annulation at Pt-Ti joint, paler annulation medially, Mt with two dusky annulations, one basal, one proximal, Ta tip dusky; spination leg I Fm d 1-1-1, pr 1-1-0, Ti v 2-2-2-2-2, Mt v 2-2-2; leg II Fm d 1-1-1, Ti v 2-2-2-1-2, Mt v 2-2-2; leg III Fm d 1-1-1; leg IV Fm d 1-1-1; leg formula 3421; measurements leg I 6.60 (2.07, 0.64, 1.74, 1.40, 0.76); leg II 7.07 (2.58, 0.68, 1.68, 1.30, 0.83); leg III 8.54 (2.53, 0.76, 2.42, 1.85, 1.00); leg IV 8.30 (2.59, 0.70, 2.18, 1.91, 0.89). Palp: spination Fm d 0-1-1; 1.73 (0.52, 0.37, 0.32, 0.58); Ti with dark mark dorsally and retrolaterally, Cy with dark mark basodorsally (Fig. 4B, D); dRTA rectangular in ventral view, slightly curved ventrally, pointed apically in lateral view, conspicuously darker than vRTA, vRTA rounded distally in ventral view, wider, rounder, shorter (Fig. 4A–D); rbcp small; Cy oval-triangular, extended further retrobasally in ventral or dorsal views (Fig. 4A–C); C located anteromedially, pointed ventrally, pyramidal, sclerotized more at tip, margins raised around E, apicalmost part of C with lip (Fig. 4A, C); E short, arising from large TL, straight, near middle of bulb, beginning at 6:30 o’clock; MA with sclerotized, hooked branch, inconspicuous unsclerotized lobe, sparse Sp at basal and lateral margins (Fig. 4A, C).

**Female.** Unknown.

##### Etymology.

The species name is the indigenous word for the type locality in the Western Aranda language. Noun in apposition.

##### Distribution.

Known from only the type locality, Kwartatuma (Ormiston Gorge), Northern Territory (Map 2).

##### Natural history.

This spider was collected by coaxing from cracks with a piece of grass in rocks on a cliff face during the day (Fig. 2G). It was collected as a penultimate ♂ on 19 April 2016 and matured to an adult male on 19 June 2016, and it no longer ate after this time. The climate at the collecting locality becomes drier and cooler with increased humidity from April to June (Suppl. material 2: table S1). The species is from the xeric and desert scrubland ecoregion. The IBRA region and subregion are the MacDonnell Range, characterized by high-relief ranges and foothills, spinifex, and acacias, especially mulga (*Acaciaaneura* Mueller ex. Bentham) (Bastin 2008).

##### Discussion.

This species is part of a group of primarily Central Australian species comprising *Karaopspilkingtoni*, *K.deserticola*, *K.mparntwe* sp. nov., *K.larapinta* sp. nov., *K.ngarutjaranya*, *K.manaayn*, and *K.vadlaadambara*. It is one of the smallest selenopids. Data indicate that there are several species found in a rather small geographic area (Map 2). The group is poorly collected (except *K.vadlaadambara*, relatively), likely due to crypsis and living in remote locations. Targeted collecting will produce additional species and specimens in better condition and provide important distribution data to uncover the true diversity of Central Australian selenopids.

#### ﻿ Karaops larapinta sp. nov.

Taxon classificationAnimaliaAraneaeSelenopidae

EEF96E2F-B485-5B47-97AF-EA970D4D32B4

https://zoobank.org/3C4B9AAD-46DD-444C-9F69-1F5672622022

**Figure 3.**
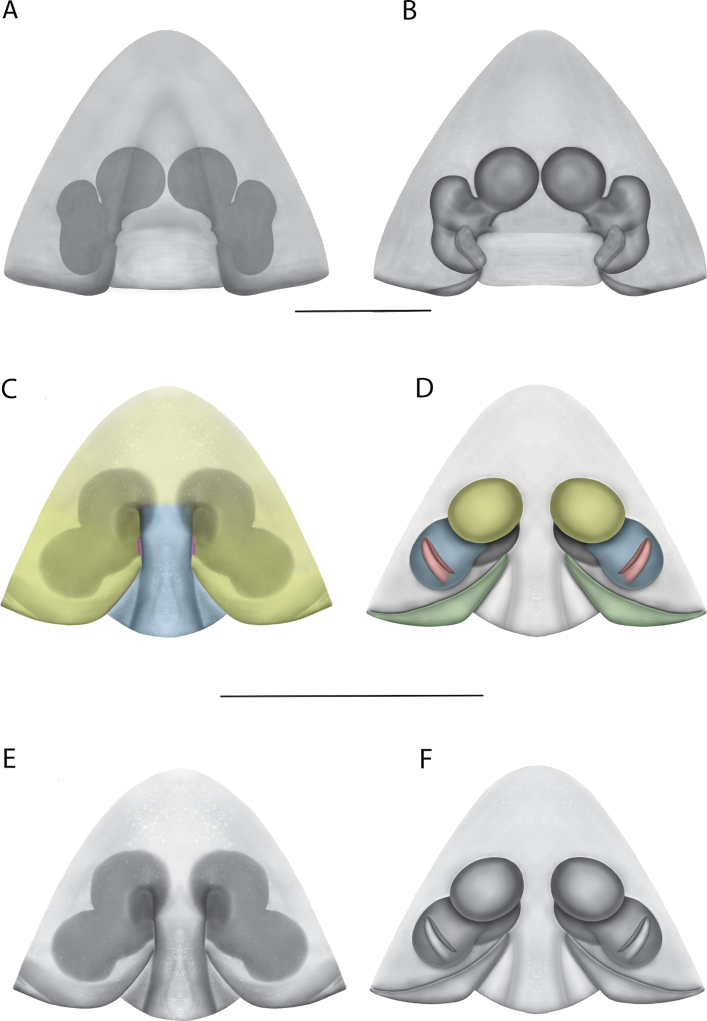
Epigynes and endogynes of members of the Central Desert species group **A***Karaopslarapinta* sp. nov., holotype female, epigyne, Finke River, Northern Territory (QMS 110852) **B** same, endogyne **C***Karaopsmparntwe* sp. nov., holotype female, epigyne, Alice Springs, Northern Territory (AMT AA 10.852), pink = copulatory openings, blue = median field, yellow = lateral lobes/epigynal plate **D** same, endogyne, yellow = accessory bulbs, blue = spermathecae, red = fertilization ducts, green = posterodorsal fold **E** same, epigyne **F** same, endogyne. Scale bars: 0.5 mm.

Figs 2C, D , 3A, B , 5C , Maps 1 , 2

##### Material examined.

***Holotype***: Northern Territory • ♀; Finke River; Oct. 1993; R. Raven leg.; No. 501; (QMS 110852). ***Paratype***: ♀; same data as holotype (QMS 12909).

##### Diagnosis.

*Karaopslarapinta* sp. nov. (Fig. 2C) is similar to other species of the Central Desert group by the median lobe that is unsclerotized posteriorly, the small copulatory ducts, and the large accessory bulbs (Fig. 3A, B). It can be differentiated from all of these except *K.pilkingtoni* by the accessory bulbs which in the other species are separated by at least half to more than one accessory bulb diameter (Crews and Harvey 2011: fig. 58). It can be separated from *K.pilkingtoni* by the accessory bulbs being nearly the same size as the spermathecae, whereas the accessory bulbs are much larger than the spermathecae in *K.pilkingtoni* (Fig. 2A, B; Crews and Harvey 2011: fig. 58). In the new species, there is a long, triangular depression in the median field that is lacking in the other species (Fig. 3A, B).

##### Description.

**Female** (holotype). Total length 7.49. Carapace: length 2.45, width 3.69. Chelicerae: promargin with three teeth, retromargin with two teeth. Eyes: AER slightly recurved, PER recurved; diameters AME 0.18, ALE 0.18, PME 0.29, PLE 0.36; interdistances AME–PME 0.04, PME–ALE 0.10, ALE–PLE 0.20, PME–PME 1.20, ALE–ALE 1.62, AME–AME 0.59, PLE–PLE 1.85. Sternum: length 1.67, width 1.89. Abdomen length 5.04, width 4.03. Color: Carapace: orange-yellow, with two dark markings lateroposterior to eye region, three marks each on lateral margins, faded, most setae worn off. Chelicerae: yellowish, paturon with a longitudinal curved mark frontally, setae white, long anteriorly. Maxillae: yellowish white. Labium: gray, pale distally. Sternum: yellowish. Abdomen: dorsally reddish orange, two dark spots at anterior margin, two laterally just posterior to these, two lateromedially posterior to these, two small ones just posterior to previous, two dark marks extend to lateral edges, connect to jagged markings lateromedially, two dark, wavy lines posterolaterally; ventrally grayish. Legs: pale yellow-orange, Cx with dusky mark prolaterally, Fm with dusky mark prolaterally, another medially, forming annulation, Pt with dark mark ventrally, Ti with dark mark ventrally at Pt-Ti joint, with annulation closer to Mt, dark annulation on Ti at Ti-Mt joint and halfway between that and Mt joint, dark annulation on Mt at Ti joint and at Mt-Ta joint, Ta tip not dark (may be faded); spines dark basally, pale distally; spination leg I Fm d 1-1-1, pr 1-1-0, Ti v 2-2-2-2-2-2, Mt v 2-2-2-2; leg II Fm d 1-1-1, Ti v 2-2-2-2-2-2, Mt v 2-2-2-2; leg III Fm d 1-1-1; leg IV Fm d 1-1-1, pr 0-0-1, rl 0-0-1; leg formula 4321; measurements leg I 13.86 (3.85, 1.65, 3.83, 3.24, 1.3); leg II 15.73 (4.95, 1.65, 4.13, 3.60, 1.40); leg III 16.13 (5.23, 1.40, 4.28, 3.70, 1.53); leg IV 16.36 (5.50, 1.48, 4.26, 3.71, 1.40). Palp: spination Fm d 0-1-3; 3.30 (1.05, 0.60, 0.75, 0.90); basally dusky, Ta dusky basodorsally; claw with eight teeth. Epigyne: EP triangular; MF with long, triangular depression, lobe posteriorly unsclerotized (Figs 3A, B, 5C); LLs separated at posterior 1/3 of EP; COs at anterolateral edges of lobe (Figs 3A, 5C). Endogyne: CDs very short; ABs larger than S, round; S oval; duct between S and ABs coiled once, FDs directed anteriorly (Fig. 3B).

**Figure 4.**
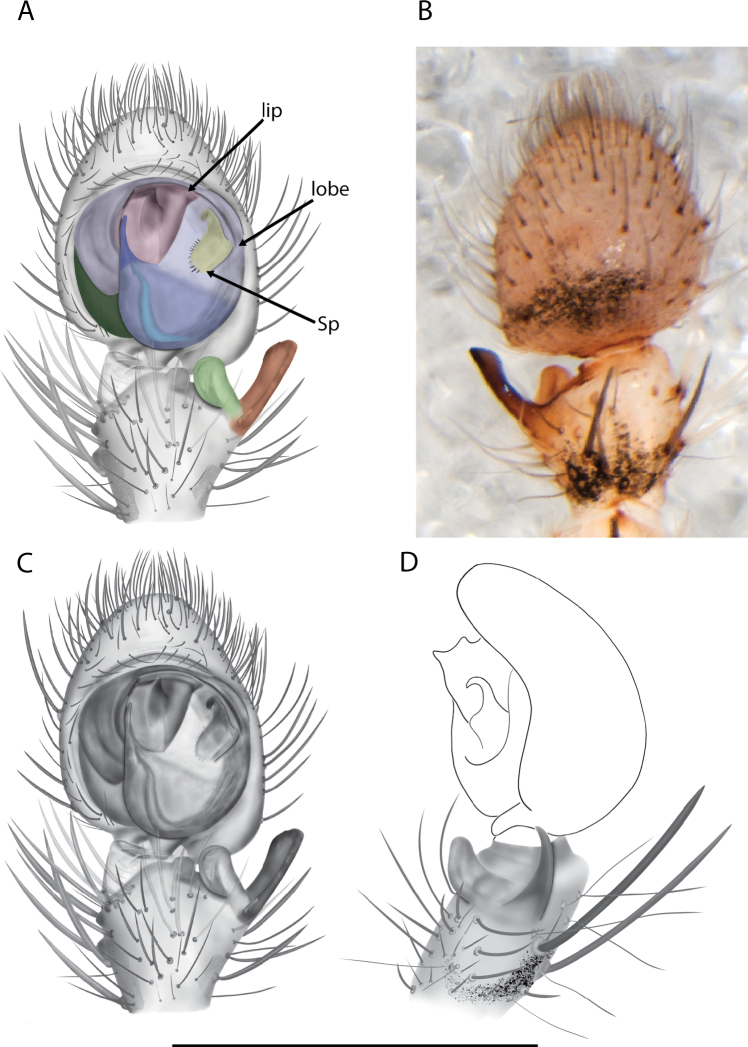
*Karaopskwartatuma* sp. nov., holotype male, (Kwartatuma) Ormiston Gorge, West MacDonnell Range National Park, Northern Territory (sel_1093, MAGNT A004858) **A** palp, ventral; green = vRTA, orange = dRTA, light gray-blue = tegulum, blue-violet = tegular lobe, turquoise = spermophor, blue = embolus, pink = conductor, yellow = MA, dark green = subtegulum, Sp = spinules **B** same, palp, dorsal **C** same, ventral **D** same, retrolateral. Scale bar: 0.5 mm.

**Figure 5.**
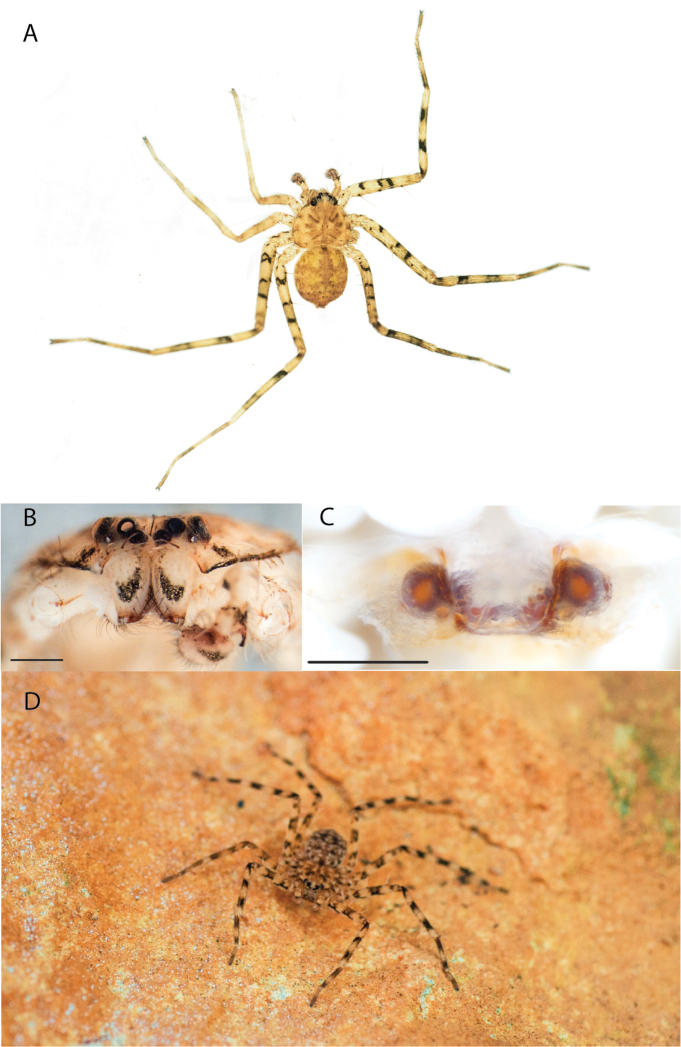
Species from the Central Desert species group **A***Karaopskwartatuma* sp. nov., holotype male, Ormiston Gorge, West MacDonnell Range National Park, Northern Territory (MAGNT A004858) **B** same, holotype male, face showing black marks near eyes and “mustache” **C***Karaopslarapinta* sp. nov., holotype, epigyne, caudal, Finke River, Northern Territory (QMS 110852) **D***Karaops* sp. from Watarrka National Park, Northern Territory. Scale bars: 0.5 mm.

**Male.** Unknown.

##### Variation.

The size of the paratype is 7.86.

##### Etymology.

The specific name is the indigenous word for the type locality, thought to be the world’s oldest river, in the Arrente language. Noun in apposition.

##### Distribution.

Known from only the type locality, Larapinta (Finke River), Northern Territory (Map 2).

##### Natural history.

*Karaopslarapinta* sp. nov. (Fig. 2C, D) is found in either the Finke or Henbury subregion of the Finke Bioregion (exact coordinates unavailable). This bioregion comprises arid sandplains, major rivers, valleys, mulga (*Acaciaaneura*) and other acacia, *Senna* Miller, *Eremophila* Brown, short grass, and forbs (Bastin 2008). These females were collected during the hottest part of the year, going from the dry into the wet (Suppl. material 2: table S1).

##### Discussion.

The region suffers from introduced species and grazing (Bastin 2008). It is biodiverse but has only been surveyed for birds and plants.

#### ﻿ Karaops mparntwe sp. nov.

Taxon classificationAnimaliaAraneaeSelenopidae

85B7F912-600A-5378-B7A4-BC3D2AF26891

https://zoobank.org/CBC9F9D6-DC69-4134-82E4-5F258C598C47

Figs 1I , 3C–F , Maps 1 , 2

##### Material examined.

***Holotype***: Northern Territory • ♀; Alice Springs; Aug.; (ZMT AA 10.852).

##### Diagnosis.

The female of *Karaopsmparntwe* sp. nov. (Fig. 1I) is similar to other members of the Central Desert species group, *K.pilkingtoni*, *K.ngarutjaranya*, *K.deserticola*, *K.vadlaadambara*, *K.larapinta* sp. nov., and *K.manaayn* in that the median field of the epigyne is a rectangular lobe with copulatory openings located anteriorly at either side of the lobe, the accessory bulbs are large, and the body lengths are 5.5–7 mm (Figs 1A–H, 2A–D). However, it can be differentiated by the raised medial portion of the lobe (Fig. 3E). The accessory bulbs are more oval than round as they are in the other species, with the spermathecae ventral and posterior to them, and the spermathecae and accessory bulbs are nearly the same size and connected by a short, thick duct (Fig. 2F). In the other species, the accessory bulbs are round, larger than the spermathecae, and the spermathecae are located posterior to the accessory bulbs, connected by a thin, coiled duct.

##### Description.

**Female** (holotype). Total length 6.97. Carapace: length 2.46, width 3.26. Chelicerae: promargin with five teeth, fourth tooth toward base of fang larger than others, retromargin with three teeth. Eyes: AER slightly recurved, PER strongly recurved; diameters, AME 0.17, ALE 0.14, PME 0.22, PLE 0.32; interdistances AME–PME 0.09, PME–ALE 0.14, ALE–PLE 0.34, PME–PME 0.84, ALE–ALE 1.23, AME–AME 0.51, PLE–PLE 1.59. Sternum: length 1.46, width 1.77. Abdomen: length 4.51, width 3.66. Color: Carapace: orangish yellow, dark patches behind eyes contiguous with darker area in median furrow, setose, with short, stiff setae, although several missing in this specimen. Chelicerae: orange-brown with dusky markings proximally and distally, nearly forming an unfilled oval on the paturon anteriorly (Fig. 1I), setae dark, more prominent anteriorly. Maxillae: orange-brown, pale distally. Labium: brownish, pale distally. Sternum: orangish. Abdomen: dorsally whitish orange, faded, with short, stiff setae, some dark spots laterally, dark chevrons medially, dark at posterior; ventrally, grayish brown. Legs: orangish, Cx II and III with black stripe prolaterally, Tr with dark mark prolaterally, dark dot on Fm at Tr-Fm joint, Fm with dark line proximally, prolaterally with horizontal extensions, nearly forming annulation, medially with dark line and two areas of horizontal extensions forming annulations, Pt with dusky area proximally, Ti with dark annulation at Pt-Ti joint and distally, Mt same, Ta tip dusky; spination leg I Fm d 1-1-1, pr 1-1-0, Ti v 2-2-2-2-2-2, Mt v 2-2-2; leg II Fm d 1-1-1, Ti v 2-2-2-2-2, Mt v 2-2-2; leg III Fm d 1-1-1; leg IV Fm d 1-1-1; leg formula 4321; measurements leg I 11.45 (3.27, 1.48, 3.19, 2.23, 1.28); leg II 12.97 (3.94, 1.55, 3.33, 3.01, 1.14); leg III 13.74 (4.78, 1.09, 3.56, 2.97, 1.34); leg IV 14.64 (4.88, 1.25, 3.66, 3.42, 1.43). Palp: spination Fm d 0-1-2; 2.99 (0.86, 0.70, 0.54, 0.89); dusky marking on Fm and Ti; claw with five teeth. Epigyne: EP triangular; MF with rectangular lobe, somewhat raised medially; COs located anteriorly on sides of lobe (Fig. 3C, E). Endogyne: CDs short, wide; ABs oval, large, anterior and dorsal to S; S large, oval; FDs directed anterolaterally; small pdf (Fig. 3D, F).

**Male.** Unknown.

##### Etymology.

The species name is the indigenous word for the type locality in the Western Arranda language. Noun in apposition.

##### Distribution.

Known from only the type locality, Northern Territory (see Discussion) (Map 2).

##### Natural history.

Nothing is known of this specimen other than it was collected in August; thus, adult females can be found in August, one of the cool, dry months in Alice Springs (Suppl. material 2: table S1).

##### Discussion.

The label appears to say “Alice Springs, Ctrl. Sta?. VIII”. There is no collector given, and searching the database at ZMT did not uncover any additional information. It was thought that Pekka Lehtinen may have been the collector, but he has never been to Alice Springs (pers. comm.). Presumably, “Ctrl. Stn.?” refers to the Old Telegraph Station. The holotype male of *Karaopspilkingtoni* is known from Alice Springs, Old Telegraph Station, base of Trig Hill. The female of *K.pilkingtoni* is from more than 60 km ENE of Alice Springs. The species were paired based on their general similarity and geographic proximity; however, it is now known that multiple species can occur in sympatry and/or syntopically, and similar species do occur nearby (*K.larapinta* sp. nov.). Based on morphology, no changes are made to the status of the female of *K.pilkingtoni*, and *K.mparntwe* sp. nov. and *K.pilkingtoni* are considered separate species. The region is poorly collected yet peppered with several species in somewhat close proximity (Map 2). Thorough collecting in the Northern Territory and South Australia as well as molecular data will help support or refute current species hypotheses and undoubtedly uncover new species. Nearby in Watarrka (Figs 2E, F, 5D) no adults of an undescribed species were found in April. Molecular data indicate that this is yet a different species from *K.kwartatuma* sp. nov. (Suppl. material 1).

### The *strayamate* species group

#### ﻿ Karaops strayamate sp. nov.

Taxon classificationAnimaliaAraneaeSelenopidae

FB7EC41C-4205-590E-8B85-7947AF0C6324

https://zoobank.org/2F0EFD0A-C9F8-4787-A8A3-FC2E84C57024

**Figure 6.**
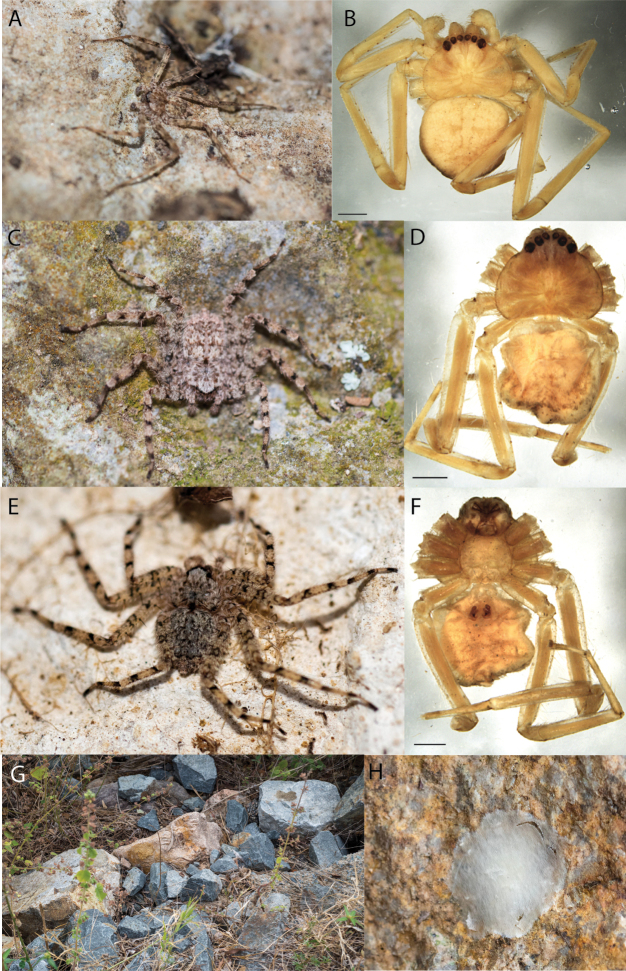
Species and habitats of the *strayamate* species group **A***Karaopsstrayamate* sp. nov., adult male, vic. Mt. Etna Caves National Park, Queensland **B***Karaopsaustraliensis* nom. dub., holotype, penultimate male, Bowen, Queensland (see text – this is likely a member of the *raveni* species group) (ZSMH A0000791) **C***Karaopsstrayamate* sp. nov., adult female, vic. Mt. Etna Caves National Park, Queensland **D***Karaopsaustraliensis* nom. dub., female, dorsal, Sydney, New South Wales (see text) (ZSMH A0000792) **E***Karaopsstrayamate* sp. nov., adult female, vic. Mt. Etna Caves National Park, Queensland **F***Karaopsaustraliensis* nom. dub., female, ventral, Sydney, New South Wales (see text) (ZSMH A0000792) **G***Karaopsstrayamate* sp. nov. habitat of rocks along roadside, Bowen, Queensland **H***Karaopsstrayamate* sp. nov., egg sac beneath rock, Bowen, Queensland. Scale bars: 1 mm.

Fig. 6A, C–H , Map 3

Karaops australiensis non L. Koch 1875: Crews and Harvey 2011: 24–27, figs 7–10 (♂♀, misidentification).

##### Material examined.

***Holotype***: Queensland • ♀; base of Jim Crow Mountain; 23°13'S, 150°38'E; Jul. 1982; A. Rozefelds leg.; (QMS 61054). ***Paratype***: ♂; Johansen’s Cave, 23°09'S, 150°28'E; 100 m; 29 May 2000; G.B. Monteith leg.; vine scrub; fogging trees with pyrethrum; (QMS 57515). **Other material examined**: 4♀, 7♂, 3 imm.; NNW of Rockhampton, just outside of Mt. Etna National Park; -23.167, 150.47; ~ 109 m; 1 Jun. 2019; S. Crews, M. Brandley leg.; under rocks, raining, 16 °C; sel_1424–1437; SCC19_011; (QMS 116840–116853) • 4♀; Bowen, Murray’s Bay Road, Rose Bay Walking Trail; -19.9863, 148.2608; ~ 26 m; 3 Jun. 2019; S. Crews, M. Brandley leg.; under rocks; sel_1438–1441; SCC19_012; (QMS 116854–116857) • 1♂; Brandy Creek; 20°21'S, 148°43'E; 15 May 1975; R. Monroe, J. Covacevich, P. Filewood leg.; (QMS 47115).

##### Diagnosis.

The female of *Karaopsstrayamate* sp. nov. can be separated from *K.monteithi* and *K.gangarie* by having fused lateral lobes and a large excavation of the posterior margin of the epigynal plate (Crews and Harvey 2011: fig. 9). The new species can be separated from *K.ellenae* by the accessory bulbs not extending beyond the copulatory ducts, whereas in *K.ellenae* they do (Crews and Harvey 2011: fig. 76). *Karaopsstrayamate* sp. nov. is also known only from the east coast and surrounds in Queensland, whereas *K.ellenae* is found in southwestern Western Australia (Map 3). The male can be separated from other species of the group by the dRTA longer than the vRTA in lateral view (Crews and Harvey 2011: figs 7, 8).

##### Description.

The description of the male and female can be found in Crews and Harvey (2011: sub *Karaopsaustraliensis*).

##### Etymology.

The name is a combination of two words: “Straya”, a term used by many Australians when referring to the country, and “mate”, meaning friend. The two terms together are quintessential Australian, which is appropriate since the name *australiensis* can no longer be used. Noun in apposition.

##### Distribution.

Known from coastal Queensland (Map 3).

##### Natural history.

Four of the five localities where *Karaopsstrayamate* has been collected are in the Brigalow Belt North bioregion, with the Bowen specimens (Fig. 6A, G, H) in the Bogie River Hills subregion, and the specimens from Johansen’s Cave, vic. Mt. Etna National Park (Fig. 6C, E), and Jim Crow Mountain from the Marlborough Plains subregion. The bioregion has tropical summer rain and is warm all year round. The Bogie River Hills comprises some lowlands, but it is mostly mountainous. The subregion has a mean rainfall of 700 mm/year. The Marlborough Hills consists of evergreen to semi-evergreen vine thicket in rare serpentinite ecosystems. The climate of these two subregions is very similar. The Brandy Creek locality occurs in the Whitsunday subregion of the Central Mackay Coast bioregion. The Central Mackay Coast bioregion has higher rainfall than some surrounding areas, and there are many endemic plants. The region is an overlap area of the wet tropics and rainforests (Reef Catchments 2014). Adult females and immatures have been collected in the cooler, drier months. Males have been collected in the drier months but during the coolest and warmest time of year as well as during the transition (Suppl. material 2: table S1). This species has been collected beneath rocks and from fogging trees with pyrethrum in vine scrub.

##### Discussion.

Crews and Harvey (2011: 24) identified several specimens from Queensland, i.e., QMS 57515, QMS 61054 and QMS 47115, as *Selenopsaustraliensis*. The types in ZSMH were not examined at the time. A re-examination of L. Koch (1875) lists the figure from page 615, pl. 48, fig. 6 as “Femina”, although the illustration appears to be of an immature male. The material at ZSMH was examined via photographs (Fig. 6B, D, F). ZSMH-A0000791, denoted as the holotype, is a juvenile male from Bowen, Queensland (Fig. 6B). The other specimen, ZSMH-A0000792, denoted as the syntype, is an adult female (Fig. 6D, F). The locality data indicate that the female specimen is from Sydney; however, this location is probably erroneous because it is unlikely that this species is found as far south as Sydney or indeed in New South Wales. Based on the curvature of the eye rows and leg spination of the holotype juvenile male, it is clear that it is a member of the *raveni* species group; thus, it may have been found in Sydney and the labels were mixed up. Because of the confusion and inability to determine the species of the juvenile male, *Karaopsaustraliensis* is considered a nomen dubium.

Fig. 10 of Crews and Harvey 2011 is labeled incorrectly: the SD and FD in fig. 10 are the copulatory ducts, SP in fig. 10 is the accessory bulb, the posteriormost part of the illustration in fig. 10 show the spermathecae which are connected to the fertilization ducts which are not illustrated in fig. 10.

#### ﻿ Karaops gangarie

Taxon classificationAnimaliaAraneaeSelenopidae

Crews & Harvey, 2011

0BD2CF36-5838-562E-B668-22F92F2BC543

Fig. 7A–C , Map 3

Karaops gangarie Crews and Harvey 2011: 28, figs 11–14 (♂, ♀, examined).

##### Diagnosis.

The lateral lobes of the epigyne of *Karaopsgangarie* are conspicuous posteriorly, and there is no excavation along the posterior margin as in *K.strayamate* sp. nov. (Crews and Harvey 2011: figs 9, 11). The lateral lobes do not frame the median field as they do in *K.monteithi* (Crews and Harvey 2011: fig. 15). In the males, the dRTA is not longer than the vRTA in lateral view as it is in *K.strayamate* sp. nov. (Crews and Harvey 2011: figs 13, 14).

##### Description.

The description of the male and female can be found in Crews and Harvey (2011).

##### Distribution.

This species is only known from Amos Bay and in the vicinity of Cooktown, on the Cape York Peninsula, northeastern Queensland.

##### Natural history.

The type locality of Amos Bay occurs in the Daintree-Bloomfield subregion of the Wet Tropics Bioregion. The second locality where the species has been collected is Cooktown in the Starke Coastal Lowlands of the Cape York Peninsula bioregion. Both immediate areas are considered tropical lowland rainforest. Rainfall throughout the year is 1300–3500 mm. The area is threatened by habitat loss, invasive species, and phenomena associated with climate change, like more frequent and larger tropical cyclones and fluctuations in precipitation. The Cape York Peninsula bioregion consists of eucalypt and melaleuca woodlands. Approximately half of the Starke Coastal lowlands are pastoral and other parts of the subregion are used for mining. The hottest months of the year in both Cooktown and Amos Bay occur from November–February, with the coolest months being June–August. The wettest months are from December–April, with the driest May–November. The holotype and paratype male and female were collected in May, a cooler, drier part of the year. The female from Cooktown was collected in January, a time of transition from drier to wetter in the hottest part of the year (Suppl. material 2: table S1). This species has been collected beneath bark in the rainforest.

##### Discussion.

**Figure 7.**
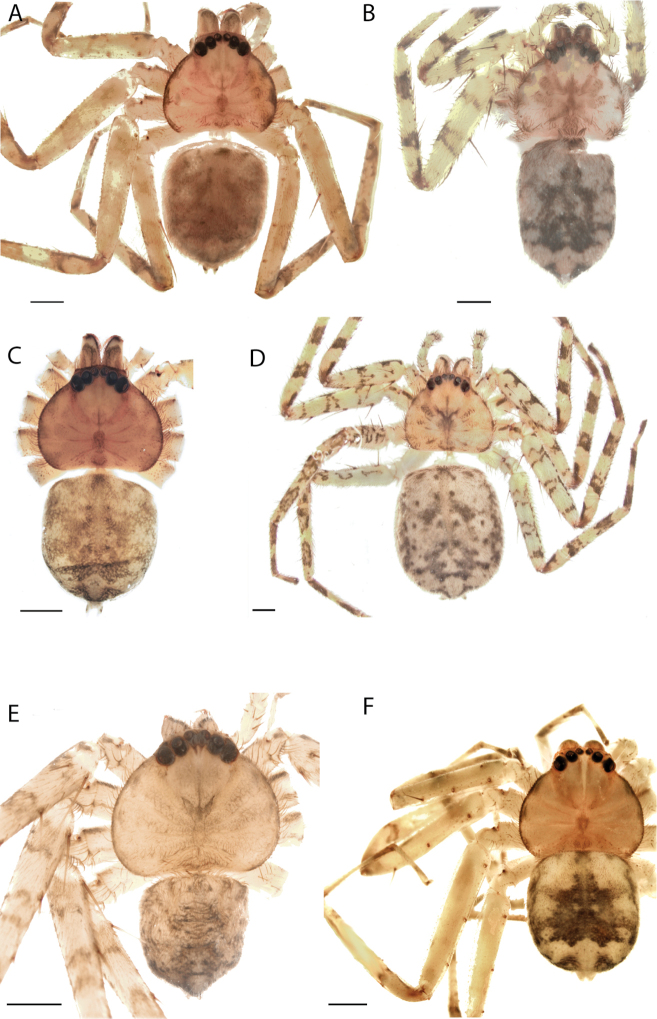
Members from the *strayamate* species group **A***Karaopsgangarie*, holotype female, rainforest near Amos Bay, Queensland (QM S52315) **B***Karaopsgangarie*, paratype female from Cooktown, Queensland (QM S88003) **C***Karaopsgangarie*, paratype male, same data as holotype (QM S88644) **D***Karaopsellenae*, paratype female, Mount Cooke, Western Australia (WAM T28043 ex. 93/1359) **E***Karaopsellenae*, holotype male, same data as paratype (WAM T28050 ex. T93/1366) **F***Karaopsmonteithi*, holotype female, Upper Lankelly Creek, Coen District, Queensland (QM S61052). Scale bars: 1 mm.

*Karaopsgangarie* has been collected once in 1973 and once in 2009. Molecular data were able to be obtained from a specimen and indicate that it is the sister taxon to *Karaopsstrayamate* in a clade with *K.ellenae* (no DNA was available for *K.monteithi*) which is corroborated by morphological data as being closely related (Suppl. material 1). The two localities where *K.gangarie* (Fig. 7A–C) has been collected are only ~ 25 km apart; however, they are in two separate bioregions. The Wet Tropics is a biodiversity hotspot, with many endemic taxa, threatened, and relictual species. The Daintree-Bloomfield subregion is considered an area of special concern because of its high density of threatened species (Pert et al. 2010).

#### ﻿ Karaops ellenae

Taxon classificationAnimaliaAraneaeSelenopidae

Crews & Harvey, 2011

28112AC6-0295-5386-8A20-E795EFE6163A

Fig. 7D, E , Map 3

Karaops ellenae Crews & Harvey, 2011: 76, figs 73–76 (♂, ♀, examined).

##### New records.

Western Australia • 1♂; Mount Cooke; 32°25'S, 116°18'E; 7 Aug. 1990; M.S. Harvey, J.M. Waldock, M. Peterson leg.; by hand; (WAM T28044) • 1♂; Wungong Dam; 32°11'42"S, 116°03'33"E; 14 Aug. 2010; F. Stahlavsky leg.; under stone; (WAM T104353) • same as previous; (WAM T104354) • same as previous; (WAM T104355) • 3♂; Jarrahdale area, ALCOA mine lease; 32°16'S, 116°06'E; 1997–1998; K.E.C. Brennan leg.; (WAM T113262) • 1 imm.; Serpentine; 32°22'S, 115°59'E; 1 Jun. 1927; L. Glauert leg.; by hand; under stone; (WAM T1586) • same as previous; (WAM T1587) • same as previous; (WAM T1588) • same as previous; (WAM T1589) • 1 imm.; Canning Dam; 32°09'S, 116°08'E; 20 Jul. 1985; D. Mead-Hunter leg.; (WAM T28032) • 1 imm.; Mount Cooke; 32°25'S, 116°18'E; 19 Sep. 1991; M.S. Harvey, J.M. Waldock leg.; by hand; under rock; (WAM T28041) • same as previous; 7 Aug. 1990; M.S. Harvey, J.M. Waldock, M. Peterson leg.; by hand; (WAM T28046) • same as previous; (WAM T28047) • same as previous; (WAM T28049) • 1 imm.; Mt. Dale; 32°08'S, 116°18'E; 27 Oct. 1998; J.M. Waldock et al. leg.; by hand; under rocks; (WAM T54981) • 1 imm.; ~ 15 km NW of Boddington Town; 32°39'45.3"S, 116°23'31.7"E; 23 Aug. 2011; A. Rakimov leg.; hand collection; granite outcrop; (WAM T117038) • 1 imm.; ~ 15 km NW of Boddington Town; 32°41'09.9"S, 116°27'10.6"E; 23 Aug. 2011; A. Rakimov leg.; hand collection; granite outcrop; (WAM T117039) • 3 imm.; same as previous; (WAM T119537) • 4 imm.; ~ 15 km NW of Boddington Town; 32°37'28.4"S, 116°20'54.4"E; 23 Aug. 2011; A. Rakimov leg.; hand collection; granite outcrop; (WAM T119538) • 1♀, 1 imm.; Toodyay; 31°33'S, 116°30'E; 25 Oct. 2009; J. Hynes leg.; on stones; (WAM T142633) • 1♀, 1 imm; ~ 15 km NW of Boddington Town; 32°41'09.9"S, 116°27'10.6"E; 23 Aug. 2011; A. Rakimov leg.; hand collection; granite outcrop; (WAM T119536).

##### Diagnosis.

Like other members of the *strayamate* species group, the male has a small tegular lobe and a long, thin embolus that follows the perimeter of the bulb. The median apophysis is larger and irregularly shaped rather than hooked like those of *Karaopsgangarie* and *K.strayamate* sp. nov., and the conductor of *K.ellenae* has a pointed projection terminally that extends beyond the cymbium (Crews and Harvey 2011: fig. 73).

The female has highly coiled ducts connecting the spermathecae and the accessory bulbs as in the other members of this species group; however, the epigyne is not indented along the bottom edge, there is no clear separation of the lateral lobes, and the accessory bulbs extend anteriorly beyond the coiled ducts, which they do not in the other species (Crews and Harvey 2011: figs 9–12, 15, 16).

##### Description.

The description of the male and female can be found in Crews and Harvey (2011).

##### Distribution.

This species is found near the west coast of southwestern Western Australia, primarily west of the Darlington Escarpment (Map 3).

##### Natural history.

*Karaopsellenae* (Fig. 7B, D) occurs in the Northern Jarrah subregion of the Jarrah bioregion, with a few collections from the Perth subregion of the Swan Coastal Plain, one record further south from the Warren subregion and bioregion, and one from the Avon Wheatbelt bioregion, subregion Katanning. As the name of the subregion suggests, the Jarrah Forest region and subregion are characterized by Jarrah-Marri forests and woodlands, and it is located primarily to the east of the Darling Escarpment. The area has a Mediterranean climate. One of the landscape features considered to be a special habitat are granite outcrops on which this species is most common (Williams and Mitchell 2001). It is warmest October through April, and also drier during this time, with the largest amount of rainfall from May through September. Males, females, and immatures have all been collected in hot, dry months and colder, wetter months with overlap of males and females during the transition from cold and wet to hot and dry. No spiders have been collected in April or May, which may be due to when collections were made rather than presence of spiders (Suppl. material 2: table S1).

##### Discussion.

**Figure 8.**
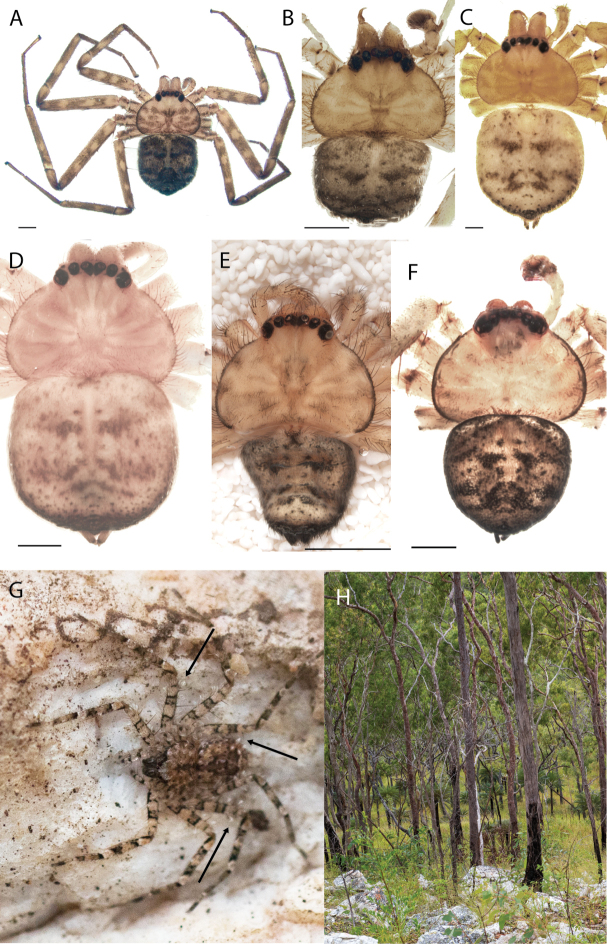
Members from the *raveni* species group **A***Karaopsjarrit*, paratype female, conveyor #2, Worsley Alumina Overland Conveyor Belt, SW of Boddington, Western Australia (WAM T87168) **B***Karaopsraveni*, holotype male, Brooyar State Forest, Queensland (QM S50593) **C***Karaopsraveni*, female paratype, Boat Mountain, Queensland (QM S47057) **D***Karaopsmarrayagong*, holotype female, Kitty’s Creek near Sydney, New South Wales (AM KS19743) **E***Karaopsmarrayagong*, female, Ku-ring-gai Wildflower Garden, New South Wales (AM KS. 126520) **F***Karaopsjarrit*, holotype male, 11 km NW of Roe’s Rock, Fitzgerald River National Park, Western Australia (WAM T55003) **G***Karaopsmonteithi*, adult female, under rock, outside of Coen, Queensland; arrows indicate tufts of white setae **H***Karaopsmonteithi*, habitat of outside of Coen, Queensland. Scale bars: 1 mm.

**Figure 9.**
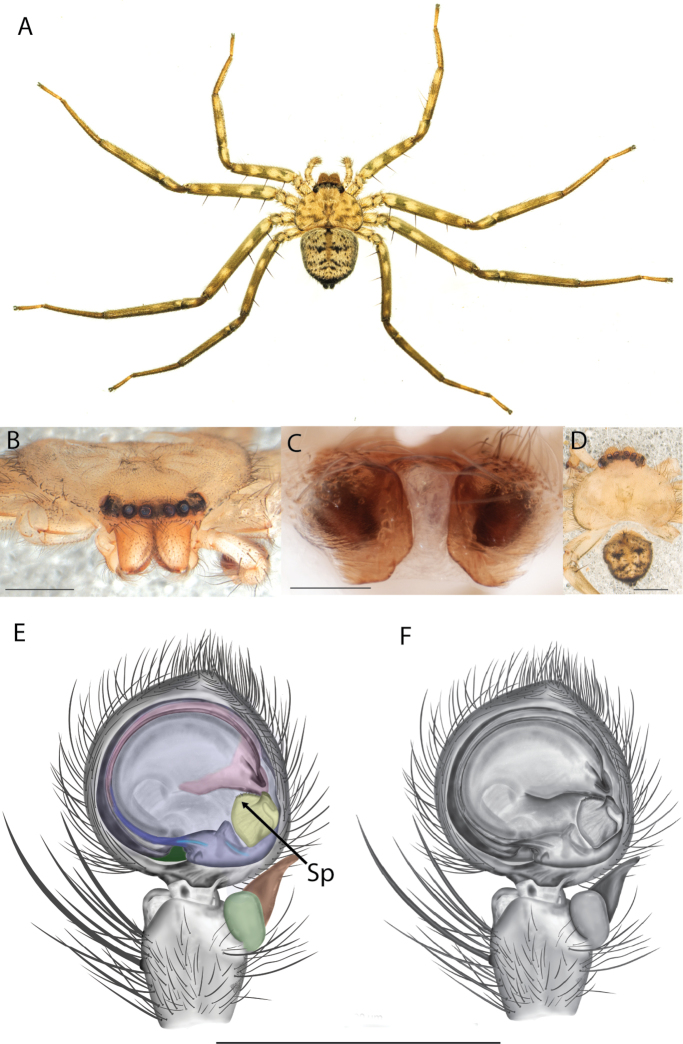
*Karaopsmarrayagong*, Ku-ring-gai Wildflower Garden, New South Wales **A** adult female (sel_1089, AM KS. 126520) **B** male, face (sel_1091, AM KS. 126522) **C** epigyne, caudal (sel_1089 AM KS. 126520) **D** male (sel_1091, AM KS. 126522) **E** same, palp, ventral; green = vRTA, orange = dRTA, light gray-blue = tegulum, blue-violet = tegular lobe, blue = embolus, turquoise = spermophor, pink = conductor/conductor sheath, yellow = median apophysis, dark green = subtegulum, Sp = spinules **F** same. Scale bars: 0.2 mm (**C**); 1 mm (**B, D–F**).

**Figure 10.**
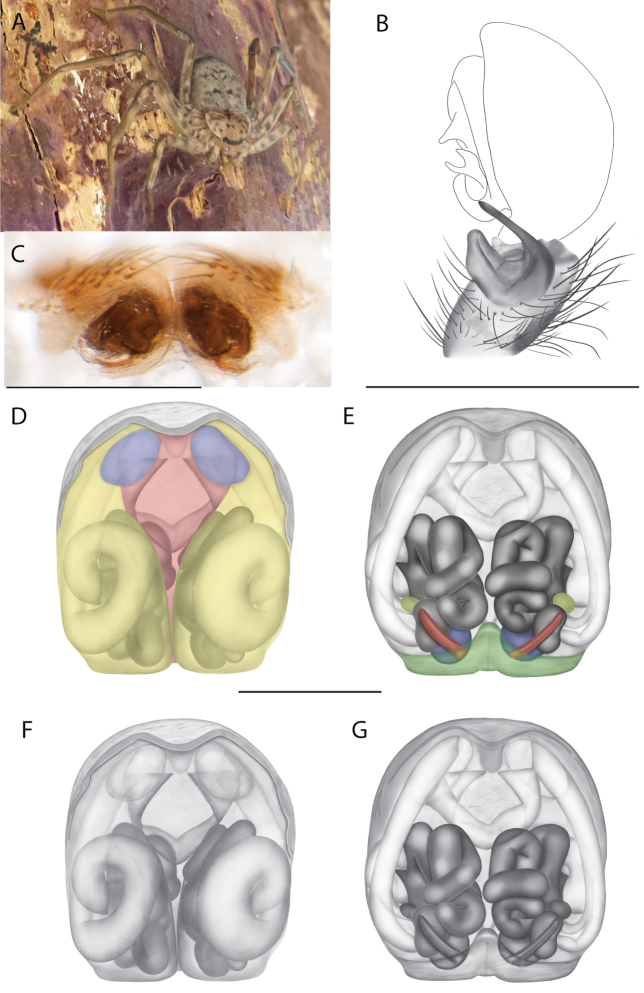
*Karaopsmarrayagong* and *Karaopsnitmiluk* sp. nov. **A***Karaopsmarrayagong* on tree, Ku-ring-gai Wildflower Garden, New South Wales (Photo: Wendy Grimm) **B***Karaopsmarrayagong*, male palp, retrolateral, same data as previous (sel_1091, AM KS. 126522) **C***Karaopsmarrayagong*, epigyne, caudal same data as previous (sel_1089, AM KS. 126520) **D***Karaopsnitmiluk* sp. nov., holotype, epigyne, Baruwei Walk, Nitmiluk National Park, Northern Territory (sel_1333, MAGNT A004906); pink = median field, blue-violet = epigynal windows, yellow = lateral lobes/epigynal plate **E** same, endogyne, yellow = accessory bulbs, blue = spermathecae, red = fertilization ducts, green = posterodorsal fold **F** same, epigyne **G** same, endogyne. Scale bars: 1 mm (**B**); 0.2 mm (**D–G**).

The other three species known to belong to this species group occur on the Queensland coast, north to at least Coen. This distribution pattern also shows up in a species with which it overlaps, *Karaopsjarrit* (Map 4) from the *raveni* species group, whose other members occur in New South Wales and Queensland. Although there are immatures in collections that could belong to either species based on locality, the *raveni* species group members are flatter than a typical *Karaops* and have a sort of squat appearance (Figs 8A–F, 9A, D, 10A). Members of the *strayamate* group are more often found under rocks, and *raveni* group members are more often found under bark.

#### ﻿ Karaops monteithi

Taxon classificationAnimaliaAraneaeSelenopidae

Crews & Harvey, 2011

6B38628F-EA87-57FF-93C1-A5D355BAF753

Figs 7F , 8G, H , Map 3

Karaops monteithi Crews & Harvey, 2011: 30, figs 15, 16 (♀, examined).

##### New record.

Queensland • 1♀ (matured in captivity 22 May 2019), 5 imm.; 30 km S of Coen on PDR, left side of road heading south; -14.2931, 143.3269; 14 May 2019; S. Crews leg.; under rocks at top of hill in woodland with *Cycas*; sel_1389–1394; SCC19_005; (QMS).

##### Diagnosis.

*Karaopsmonteithi* (Fig. 7F) can be distinguished from other members of the *strayamate* species group by the lateral lobes forming a diamond-shaped median field, which they do not in the other species of the group (Crews and Harvey 2011: fig. 15).

##### Description.

The description of the female can be found in Crews and Harvey (2011).

**Male.** Unknown.

##### Distribution.

This species is known only from the Cape York Peninsula, Queensland.

##### Natural history.

*Karaopsmonteithi* has been collected beneath rocks in a forest with *Cycas* (Fig. 8H). Both localities of *Karaopsmonteithi* (Fig. 8G, H) occur in the Coen-Yambo Inlier subregion of the Cape York Peninsula bioregion (Addicott et al. 2018). This subregion has a complex geology in the east with volcanics and metamorphic rocks. Primary vegetation is eucalypt woodland (Fig. 8H).

##### Discussion.

The type specimen was collected in the hottest, drier part of the year, while the other female and immatures were collected in the drier part of the year, but during a transition month from hot to cooler (Suppl. material 2: table S1).

### The *raveni* species group

#### ﻿ Karaops raveni

Taxon classificationAnimaliaAraneaeSelenopidae

Crews & Harvey, 2011

288D0635-2326-5606-853D-0443CB90BDF1

Fig. 8B, C , Map 4

Karaops raveni Crews & Harvey, 2011: 42, figs 29–32, 91, 111 (♂, ♀, examined).

##### New records.

New South Wales • 1♂; Oxley Wild Rivers NP, East Kunderang Track “Raspberry Rd” Dam; -30.813417, 152.084026; ~ 800 m; 5 Nov. 2015; H.M. Smith leg.; night collecting, on tree trunk; (AM KS.124443) • 1♀; same data as previous, Oxley Wild Rivers National Park, East Kunderang, Smail’s Creek, Upper Kunderang Brook; -31.0439, 152.2215; ~ 333 m; 14 Nov. 2015; H.M. Smith leg.; (AM KS.124613) • 1♂ (collected as juvenile, matured Spring 2016); same data as previous, ‘Mudaridge’, Gloucester River Rd.; -32.0547, 151.745; 3 Mar. 2016; G.M. Milledge, H.M. Smith leg.; under bark; (AM KS.126578).

##### Diagnosis.

The female of *Karaopsraveni* can be distinguished from *K.marrayagong* by the lateral lobes of the epigyne almost touching or touching posteriorly, and the accessory bulbs are separated by more than one accessory bulb in the latter (Crews and Harvey 2011: figs 27, 28, 31, 32).

The male of *Karaopsraveni* (Fig. 8B) is similar to the males of *K.marrayagong* and *K.jarrit* by the body shape (flatter and wider than species in other *Karaops* species groups; Fig. 9B, D), the horizontally oval sternum, and the long dRTA, curved ventrally and tapered to a point (Figs 8D, E, 9A, D, 10B). *Karaopsraveni* can be differentiated from *K.marrayagong* by lacking spinules on the median apophysis. It can also be differentiated from both *K.jarrit* and *K.marrayagong* by the dRTA, which is at least two times as long as the vRTA of *K.raveni* (Fig. 10B; Crews and Harvey 2011: fig. 30).

##### Description.

The description of the male and female can be found in Crews and Harvey (2011).

##### Distribution.

*Karaopsraveni* occurs in eastern Australia, in Queensland and New South Wales. It has primarily been collected to the east of the Great Dividing Range with some collections within the boundaries of the Great Dividing Range (Map 4).

##### Natural history.

This species occurs across many different subregions. The southernmost locality is in the Tinderry Range near the ACT, in the Kybeyan-Gourock subregion of the South Eastern Highlands bioregion, and the northernmost is the Burnett-Curtis Coastal Lowlands subregion of the South Eastern Queensland bioregion. The former has a temperate climate, and it does snow at the higher elevations; the northern bioregion is considered humid subtropical. The furthest inland that the species is known from is Warrumbungles National Park, of the Castlereach-Barwon subregion in the Darling Riverine Plains Bioregion. In Queensland, this species has been collected inside a house, by fogging trees with pyrethrum, and from under bark of *Ficus* and *Eucalyptus*. In New South Wales, *Karaopsraveni* has been collected from under bark, on a water tank, on trees at sunset and at night, and in the Tinderry Range it has been collected under rocks of scree slope.

##### Discussion.

*Karaopsraveni* (Fig. 8B, C) appears to be the most widespread species of *Karaops* (Map 4). There is some variation in the species as discussed in Crews and Harvey (2011), which may be more noticeable in this species because significantly more individuals over a broader range have been collected than in any of the other species. The sister taxon of *K.raveni*, *K.marrayagong*, is known only from a very small area within the Sydney Basin, inside the range of *K.raveni* (Map 4). They have been collected at the same time at the same place. The sister taxon to these two species, *K.jarrit*, occurs in the southwest of the continent, a pattern that shows up in other taxa, including other species of *Karaops*. Another species that occurs within the range of *K.raveni* is *K.manaayn*, a member of the Central Desert species group. Penultimate males have been taken at the beginning of the year in Queensland and New South Wales, including the Tinderry Range. In Queensland, no adults have been collected from February to May, generally, a transition time from hot and wet to cool and dry; and in New South Wales, no adults have been collected from August to October, a transition time from cool to warm, with not much change in rainfall (Suppl. material 2: table S1).

#### ﻿ Karaops jarrit

Taxon classificationAnimaliaAraneaeSelenopidae

Crews & Harvey, 2011

49A14BD6-6987-5893-8A34-96704B1E2DD9

Fig. 8A, F , Map 4

Karaops jarrit Crews & Harvey, 2011: 36, figs 23–26 (♂, ♀, examined).

##### New records.

Western Australia • 1♂; Rivervale, 177 Knutsford Avenue; 31°57'50"S, 115°55'43"E; 10 Nov. 2010; V.W. Framenau leg.; sifted litter; in house; (WAM T108825) • 1♀; ~ 48 km NNE Koolyanobbing; 30°21'51.48"S, 119°37'59.36"E; 9–17 Oct. 2013; C. Knuckey leg.; dry pitfall trap; minor creekline; (WAM T128844) • 1♂; same as previous; (WAM T128845) • 1 imm.; Bungalbin Hill, 48.2 km NNE of Koolyanobbing; 30°21'38.10"S, 119°41'53.67"E; 3 Apr. 2013; S. White, A. Heidrich, A. Nowicki, J. Vos, F. Bokhari leg.; hand; leaf litter; (WAM T130654) • 1♂; ~ 38 km WSW of Quindanning, Worsley Alumina conveyor #2, night shift; 33°05'10"S, 116°08'56"E; 22 Dec. 2007; J. Hynes leg.; (WAM T111761) • 1 imm.; ~ 26 km NE of Harvey, N of Worsley Alumina Overland Conveyor #1; 33°00'24"S, 116°10'17"E; 25 Oct. 2009; J. Hynes leg.; night shift; (WAM T143564).

##### Diagnosis.

The female of *Karaopsjarrit* can be distinguished from *K.raveni* by the lateral lobes of the epigyne almost touching or touching posteriorly, and the accessory bulbs are separated by more than one accessory bulb width in the latter species (Crews and Harvey 2011; figs 27, 28, 31, 32). The epigynes of *K.jarrit* and *K.marrayagong* are very similar (Crews and Harvey 2011: figs 25–28), and with only a few specimens of each, it is difficult to determine if differences between the two will remain useful for their separation. The median field is wider and shorter in *K.marrayagong* than in *K.jarrit*, and the accessory bulbs of the former are closer together. Additionally, *K.marrayagong* has 4 promarginal teeth and *K.jarrit* has 3. Although many people find the idea of using geography to separate species problematic, currently the easiest way to distinguish these two species is that *K.jarrit* is found in the southwest of Western Australia, and *K.marrayagong* is known from a single locality near Sydney, close to the east coast of the continent in New South Wales (Map 4).

The male of *Karaopsjarrit* (Fig. 8F) is similar to the males of *K.raveni* and *K.marrayagong* by the body shape (flatter and wider than species in other *Karaops* species groups (Fig. 9B, D)), the horizontally oval sternum, and the long dRTA, curved ventrally and tapered to a point (Figs 8D, E, 9A, D, 10B). It can be differentiated from *K.marrayagong* by lacking spinules on the median apophysis and from *K.raveni* by the tegular lobe which is located more toward the center of the bulb, whereas it is located more retrolaterally in this species. Additionally, it can be geographically differentiated from *K.jarrit* because they are located on opposite sides of the continent (Map 4).

##### Description.

The description of the male and female can be found in Crews and Harvey (2011).

##### Distribution.

This species is found in southwestern Western Australia (Map 4).

##### Natural history.

The greatest number of specimens is known from the Northern Jarrah Forest subregion of the Jarrah Forest bioregion, with two localities near Perth, from the Perth subregion of the Swan Coastal Plain bioregion, a single locality in the Fitzgerald subregion of the Esperance Plains bioregion, and most recently from the Southern Cross subregion of the Coolgardie bioregion. The first three subregions have a warm to hot Mediterranean climate. Data indicate that adults are primarily found in the warmer to hotter times of the year with little rainfall. This species has been collected in a pitfall trap, in leaf litter, and on an overland conveyer at night.

##### Discussion.

*Karaopsjarrit* (Fig. 8A, F) is known from four disjunct localities in southwestern Australia, likely reflecting collecting efforts rather than actual distribution. *Karaopsjarrit* overlaps in distribution with *K.ellenae* and *K.francesae*, none of which are in the same species group. The sister taxa of *K.jarrit* are *K.raveni* and *K.marrayagong* on the east coast from New South Wales to Queensland (Suppl. material 2: table S1).

#### ﻿ Karaops marrayagong

Taxon classificationAnimaliaAraneaeSelenopidae

Crews & Harvey, 2011

23DE16B8-A312-553B-9532-AD01705E73CA

Figs 8D, E , 9A–F , 10A, B , Map 4

Karaops marrayagong Crews & Harvey, 2011: 40, figs 27–28 (♀, examined).

##### New records.

New South Wales • 1 imm.; near Sydney, Ku-ring-gai Wildflower Garden; 33°42.383'S, 151°10.480'E; ~ 178 m; 9 Apr. 2016; S. Crews, P. Harlow leg.; under bark of tree; missing two legs when collected; sel_1088; SCC16_008; (AM KS.126519) • 1♀, 1 imm., 1♂ (reared in captivity); same data as previous, except 33°42.353'S, 151°10.402'E; ~ 125 m; 10 Apr. 2016; S. Crews, P. Harlow, H. Smith leg.; under bark of small eucalypts in gully area on Management Trail; sel_1089–1091; SCC16_009; (AM KS.126520–126522).

##### Diagnosis.

The female of *Karaopsmarrayagong* can be distinguished from *K.raveni* by the lateral lobes of the epigyne almost touching or touching posteriorly, and the accessory bulbs are separated by more than one accessory bulb width (Crews and Harvey 2011: figs 27, 28, 31, 32). The epigynes of *K.jarrit* and *K.marrayagong* are very similar (Crews and Harvey 2011: figs 25–28), and with only a few specimens of each, it is difficult to determine if differences between the two will remain useful for their separation. The median field is wider and shorter in *K.marrayagong* than in *K.jarrit*, and the accessory bulbs of the former are closer together. Although many people find the idea of using geography to separate species problematic, currently the easiest way to distinguish these two species is that *K.jarrit* is found in the southwest of Western Australia and *K.marrayagong* is known from a single locality near Sydney near the east coast of the continent in New South Wales (Map 4).

The male of *Karaopsmarrayagong* (Fig. 8E) is similar to the males of *K.raveni* and *K.jarrit* by the body shape (flatter and wider than species in other *Karaops* species groups (Fig. 9B, D)), the horizontally oval sternum, and the long dRTA, curved ventrally and tapered to a point (Figs 8D, E, 9A, D, 10B). It can be differentiated from *K.raveni* and *K.jarrit* by the median apophysis, which has spinules along the anterior margin and two distinct branches. It can also be differentiated by the dRTA, which is at least two times as long as the vRTA in *K.raveni* (Fig. 10B; Crews and Harvey 2011: fig. 30). In both *K.raveni* and *K.jarrit*, the vRTA is distally more knob-like due to an abrupt widening, and it is more uniform throughout its length in *K.marrayagong* (Fig. 9E, F; Crews and Harvey 2011: figs 23, 29). The tegular lobe of *K.jarrit* is located medially and extends basally to cover part of the cymbium (Crews and Harvey 2011: fig. 23), whereas in *K.marrayagong*, it is located more retrolaterally, and it is vertically narrow, not covering much of the cymbium basally (Fig. 9E, F). Additionally, it can be geographically differentiated from *K.jarrit* because they are located on opposite sides of the continent (Map 4).

##### Description.

**Male** (sel_1091, AM KS.126522). Total length 4.23. Carapace: length 2.41, width 3.50. Chelicerae: promargin with three teeth, retromargin with two teeth. Eyes: AER nearly straight, PER slightly recurved; diameters AME 0.14, ALE 0.08, PME 0.14, PLE 0.21; interdistances AME–PME 0.10, PME–ALE 0.28, ALE–PLE 0.22, ALE–ALE 1.26, AME–AME 0.51, PLE–PLE 1.60. Sternum: length 1.17, width 1.81. Abdomen: length 1.82, width 1.70. Color: Carapace: pale yellowish brown, with sparse, thin setae and longer, thick setae laterally, posteriorly. Chelicerae: brown laterally, lightened to yellowish brown medially, dusky marks, more setae anteriorly than laterally. Maxillae: pale yellowish brown. Labium dusky, pale distally. Sternum: pale yellowish brown, darker at margin. Abdomen: dorsally tan, dark laterally and around posterior, including spinnerets, with two large black marks on anterior half, some paler chevrons posteriorly, with several dark flecks; ventrally tan. Legs: pale yellowish brown, dusky, some paler spots on Fm; spination leg I missing; leg II Fm d 1-1-0, pr 1-1-0, Ti v Ti 1-1-1-1-1, weak, unpaired; leg III Fm d 1-1-0, pr 1-1-0; leg IV Fm d 1-1-1; measurements leg I missing; leg II 19.19 (6.14, 1.43, 5.43, 4.43, 1.76); leg III 14.9 (4.21, 1.43, 4.36, 3.43, 1.47); leg IV 11.86 (4.07, 0.71, 3.36, 2.5, 1.22). Palp: spination Fm d 0-1-3; 2.67 (0.83, 0.49, 0.53, 0.82); Pt dusky retrolaterally, Ti dusky basodorsally (Fig. 5D); dRTA longer than vRTA, curved ventrally, pointed at end, vRTA of uniform width, hollowed out dorsally; rbcp absent; Cy round, setae gradually denser apically, without conspicuous brush or chemosensory area; C large, CS around E, horizontally and vertically broad, top half of bulb, with sclerotized apophysis directed retrolaterally, rounded at tip, with medial groove; E long, arising from vertically narrow TL at 6 o’clock, along prolateral margin of cymbium, ending ~ 1 o’clock; MA wide, with striae, Sp along anterior margin, with two branches, anterior small, broad, sclerotized, posterior longer, narrower, less sclerotized (Fig. 9E, F).

**Female** (sel_1089, KS.126520). Habitus, live (Figs 9A, 10A), preserved (Fig. 8D, E); epigyne, caudal view (Fig. 9C). For a detailed description of the holotype, see Crews and Harvey (2011).

##### Distribution.

Known only from the type locality and locality provided above, vicinity of Sydney, New South Wales, see Discussion (Map 4).

##### Natural history.

*Karaopsmarrayagong* is found in Sydney coastal dry sclerophyll forest, under bark of large and small eucalypts. An adult female, a subadult male, and two immatures of different instars were encountered in April. Simultaneously collecting adults and immatures of multiple instars is common in the family. The male (sel_1091) and an immature (sel_1090) died soon after collection (April). The female (sel_1089) and the other immature (sel_1088) died in November 2016. This species is found in a temperate broadleaf and mixed forest ecoregion. The IBRA bioregion is the Sydney Basin, subregion Pittwater. Adults and penultimate females were collected in one of the hottest months, with rainfall transitioning from heavy to light. The Sydney Basin has a temperate climate with no dry season. *K.marrayagong* has been collected beneath bark.

##### Discussion.

There is ambiguity regarding the type locality of this species (Crews and Harvey 2011). The holotype is quite old, with little information other than “Kitty’s Creek”, an area that has been drastically developed in the last 100 years since the collection was made. The spider was encountered in a less-developed area thought to be near the type locality. It is difficult to determine if they are rare because they can be difficult to find, and typically no one is looking for them. *Karaopsmarrayagong* is morphologically similar to *K.raveni* and is found within the range of this species. *Karaopsraveni* and *K.marrayagong* are sister taxa (Suppl. material 1).

### The *dawara* species group

#### ﻿ Karaops dawara

Taxon classificationAnimaliaAraneaeSelenopidae

Crews & Harvey, 2011

A7DB9A1F-278A-52BA-BB42-890D4B4952AF

**Figure 11.**
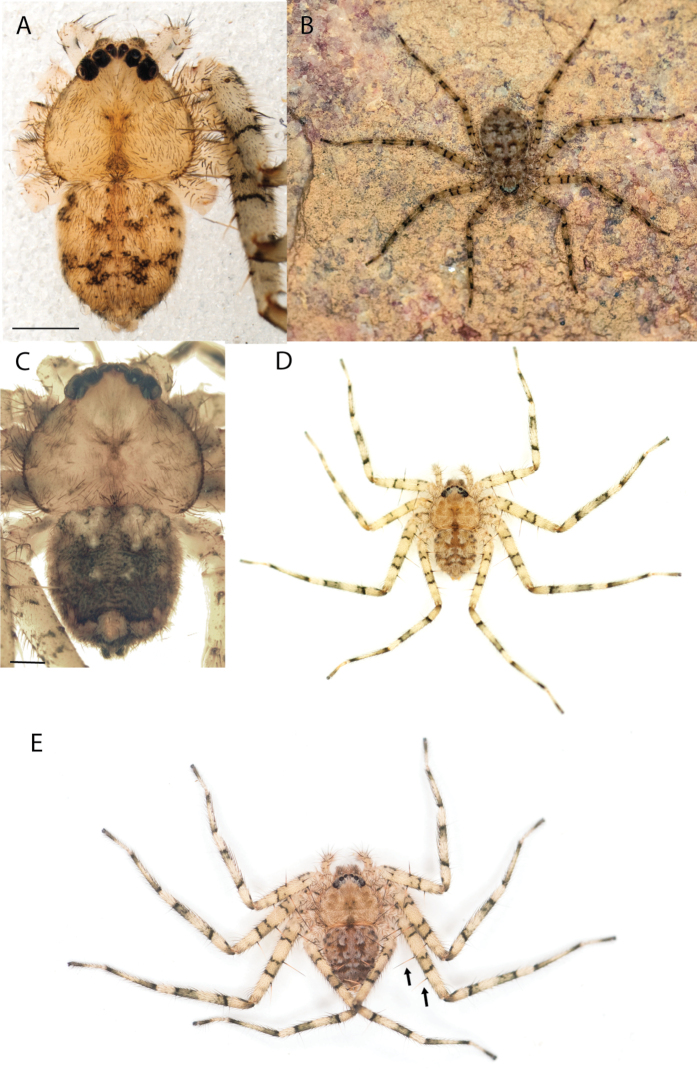
*Karaopsnitmiluk* sp. nov. and *Karaopsdawara* from the *dawara* species group **A***Karaopsnitmiluk* sp. nov., holotype, Baruwei Walk, Nitmiluk National Park, Northern Territory (sel_1333, MAGNT A004906) **B***Karaopsnitmiluk* sp. nov. **C***Karaopsdawara*, female holotype, Kakadu National Park, Kapalga, Northern Territory (WAM T54998) **D***Karaopsnitmiluk* sp. nov., holotype, (sel_1333, MAGNT A004906) **E***Karaopsnitmiluk* sp. nov., arrows indicate orange and black spines (sel_1334, MAGNT A004890). Scale bars: 1 mm.

Fig. 11C , Maps 1 , 5

Karaops dawara Crews & Harvey, 2011: 81, figs 79, 80 (♀, examined).

##### New record.

Northern Territory • 1 imm.; Kakadu National Park, Kapalga, north side of site E; 12°38.038'S, 132°23.122"E; 18 Jan. 2009; S. Crews, G. Brown leg.; (WAM T97230).

##### Diagnosis.

*Karaopsdawara* (Fig. 11C) can be differentiated from other members of the *dawara* species group by the endogyne. The distance between turns of the copulatory ducts is longer, the ducts in general are more horizontal and thinner, and the turns are much tighter. The accessory bulbs are also more easily seen in this species as they occur at the end of the ducts (Crews and Harvey 2011: fig. 80).

##### Description.

The description of the female can be found in Crews and Harvey (2011).

**Male.** Unknown.

##### Distribution.

This species has been found at the northern part of the Top End, Northern Territory.

##### Natural history.

Females have been collected in November and January. In both months temperatures are at their highest, though they do not differ much throughout the year. The former is going into the wet season, and the latter is one of the wettest months of the year. *Karaopsdawara* occurs in the Darwin Coastal region and subregion. The generally low area is drained by many large rivers and comprises forests of eucalypts with grasses (Crews and Harvey 2011: fig. 102) (Suppl. material 2: table S1). This species has been collected beneath wood/logs on the ground and beneath rocks.

##### Discussion.

No collections of *Karaopsdawara* (Fig. 11C) have been made since 2009, thus the male remains unknown. In *K.nitmiluk* sp. nov., there is a lot of genitalic variation (see below). There are only two female specimens of *K.dawara*. When more specimens are found, there may be a lot of variation in this species, and diagnoses will need to be emended.

#### ﻿ Karaops nitmiluk sp. nov.

Taxon classificationAnimaliaAraneaeSelenopidae

9C441BE4-24F1-5EB7-8327-E17097A6E563

https://zoobank.org/183953E4-D180-462D-B4EF-C502FD9E0604

**Figure 12.**
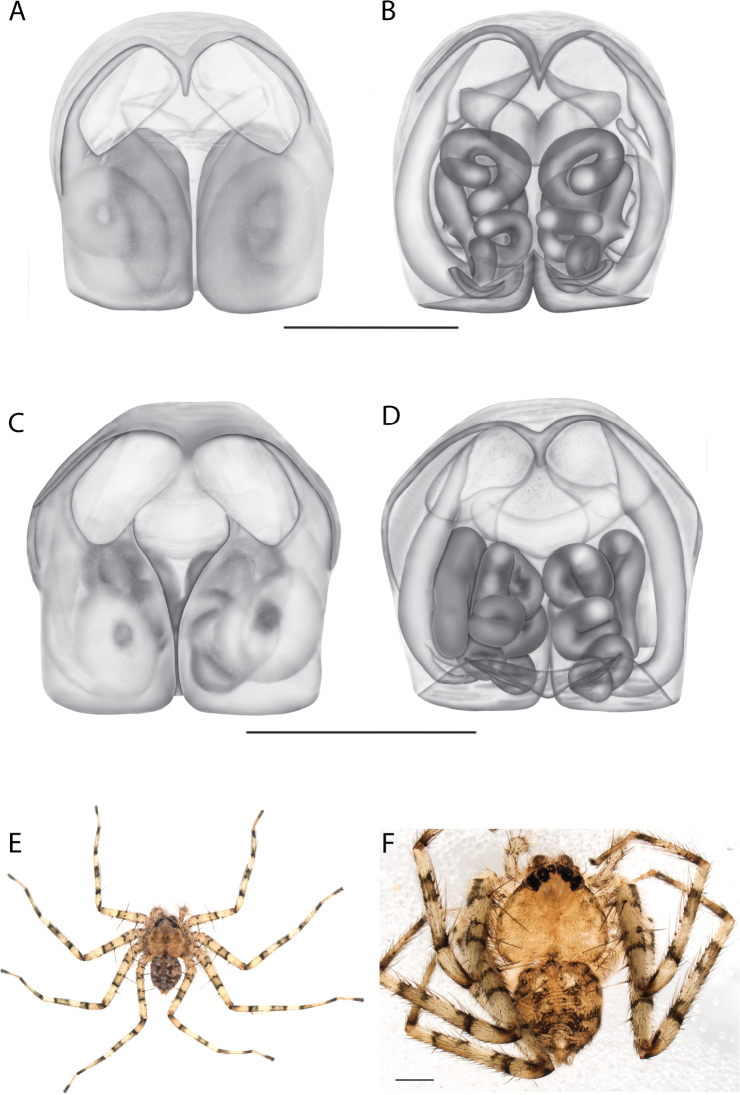
*Karaopsnitmiluk* sp. nov. from Nitmiluk National Park, Northern Territory **A** epigyne, Baruwei Walk (sel_1334, MAGNT A004890) **B** same, endogyne **C** paratype, epigyne, Leliyn (sel_1339, MAGNT A004895) **D** same, endogyne **E** same **F** same. Scale bars: 0.2 mm (**A–D**); 1 mm (**F**).

**Figure 13.**
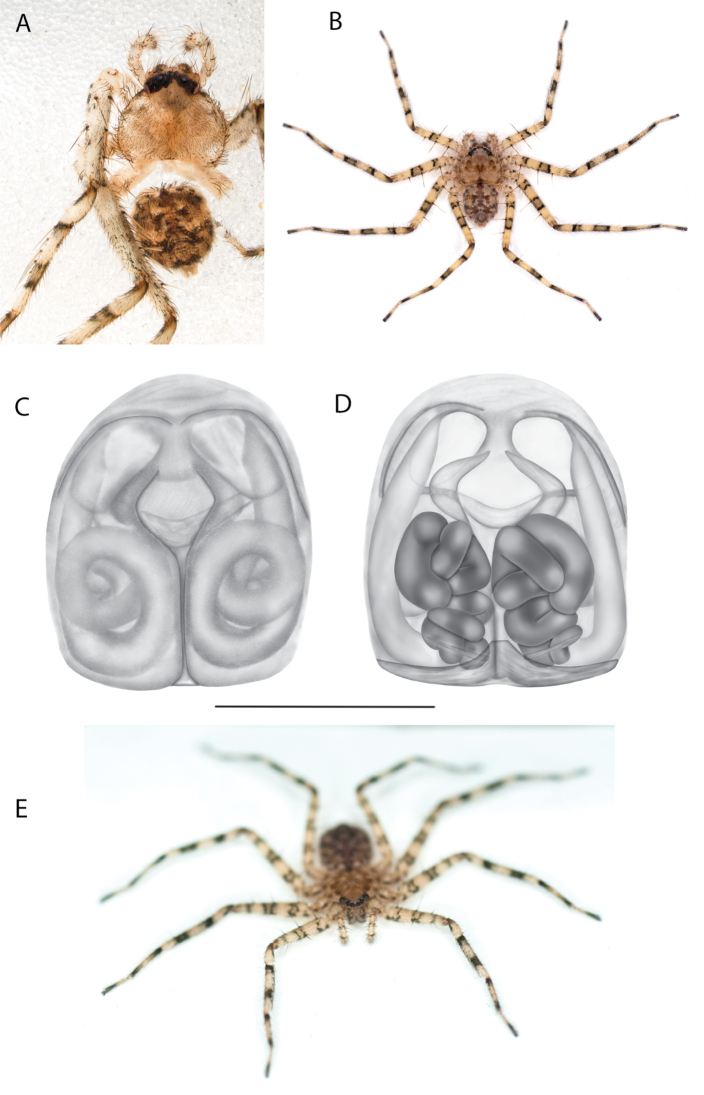
*Karaopsnitmiluk* sp. nov. and *Karaopsjawayway* sp. nov. from the *dawara* species group **A***Karaopsnitmiluk* sp. nov., Leliyn, Nitmiluk National Park, Northern Territory (sel_1342, MAGNT A004898) **B** same **C** same, epigyne **D** same, endogyne **E***Karaopsjawayway* sp. nov., holotype, Savannah Way, Northern Territory (sel_1349, MAGNT A004905). Scale bar: 0.2 mm.

Figs 10C–G , 11A, B, D, E , 12A–F , 13A–D , Maps 1 , 5

##### Material examined.

***Holotype***: Northern Territory • ♀ (reared in captivity); Nitmiluk National Park, Baruwei Walk; vic. 14°18'50.36"S, 132°25'23.16"E; 1 Jun. 2016; S. Crews, J. DeJong leg.; on rocks at night; sel_1333; SCC16_071; (MAGNT A004906). ***Paratype***: ♀ (reared in captivity); Nitmiluk National Park, Leliyn; vic. 14°10'45.40"S, 132°11'19.58"E; 2 Jun. 2016; S. Crews, J. DeJong leg.; under rocks along trail during the day; sel_1339; SCC16_072; (MAGNT A004895). **Other material examined**: 1♀ (reared in captivity), 3 imm., same data as holotype; sel_1334–1337; (MAGNT A004890–A004893) • 1♀ (reared in captivity), 3 imm.; same data as paratype; sel_1338, 1340–1342; (MAGNT A004894, A004896–A004898).

##### Diagnosis.

**Figure 14.**
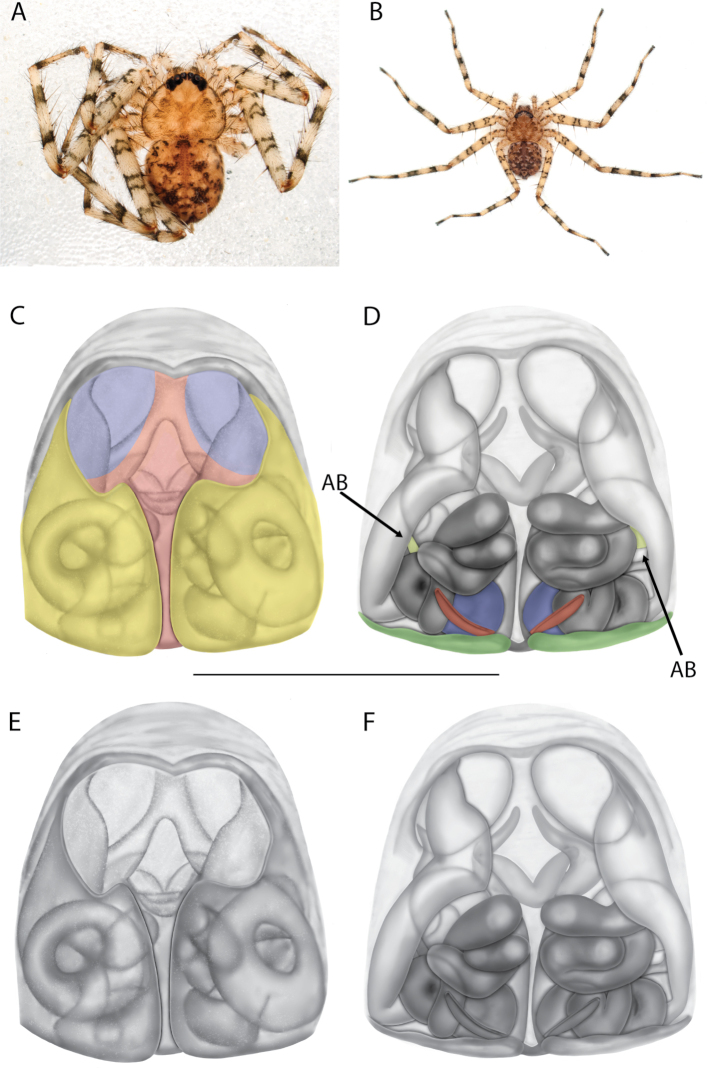
*Karaopsjawayway* sp. nov., holotype, Savannah Way, Northern Territory (sel_1349, MAGNT A004905) **A** preserved **B** alive **C** epigyne, pink = median field, blue-violet = epigynal windows, yellow = lateral lobes **D** endogyne, yellow = accessory bulbs, blue = spermathecae, red = fertilization ducts, green = posterodorsal fold, AB = accessory bulb **E** epigyne **F** endogyne. Scale bar: 0.2 mm.

Females of *Karaopsnitmiluk* sp. nov. (Figs 11B, D, E, 12E, 13B) are most similar to *K.jawayway* sp. nov. (Figs 13E, 14B) and *K.dawara* by the tortuous copulatory ducts (Figs 10F, 13C, D, 14C–F; Crews and Harvey 2011: figs 79, 80) and the unsclerotized part of the copulatory ducts. In *K.nitmiluk* sp. nov., the copulatory ducts are of mostly uniform diameter throughout their length and are straight or slightly curved anterior to posterior (Figs 10C, 11E, 12D, 13D), but in *K.jawayway* sp. nov., they are wide from the copulatory openings, narrowing posteriorly and curving outward within the first third of their length (Fig. 14D, F). In *K.nitmiluk* sp. nov., the sclerotized portion of the copulatory ducts nearly reaches the epigynal windows where the copulatory openings are located. Additionally, the sclerotized portion of the ducts has coils that largely run anterior to posterior, whereas in *K.jawayway* sp. nov., they are more than one diameter of the sclerotized ducts away from the epigynal windows, and the ducts are mostly horizontal. In *K.dawara*, the ducts are horizontal and thin, with longer lengths between turns, and the accessory bulbs are easily visible at the anterior part of the copulatory duct. In *K.nitmiluk* sp. nov., the accessory bulbs are very difficult to discern (Fig. 10E).

##### Description.

**Female** (holotype). Total length 4.55. Carapace: length 2.18, width 2.42. Chelicerae: promargin with five teeth, the fourth one, closest to base of fang, larger than others, retromargin with three teeth. Eyes: AER recurved, PER strongly recurved; diameters AME 0.13, ALE 0.10, PME 0.20, PLE 0.26; interdistances AME–PME 0.06, PME–ALE 0.11, ALE–PLE 0.27, PME–PME 0.77, ALE–ALE 1.05, AME–AME 0.37, PLE–PLE 1.34. Sternum: length 1.08, width 1.05. Abdomen: length 2.37, width 2.01. Color (in life/preserved): Carapace: pale brownish yellow with two pairs of dark spots behind ocular area laterally, dark near furrow, dark patch posteriorly, setose with white setae behind eyes, three pairs of dark spots laterally, some whitish patches/more orangish tan with markings less conspicuous. Chelicerae: tan, paturon with a longitudinal curved mark frontally, setae sparse, pale, darkened anteromedially. Maxillae: yellowish brown. Labium: tan, pale distally. Sternum: yellowish. Abdomen: dorsally with anterior reddish brown medial anchor-shaped marking extended about halfway posteriorly, dark markings laterally, two dark chevrons ~ 1/3 from posterior, anteriormost extended to lateral edges, posterior are not/same markings but colors more orangey than yellowish; ventrally yellowish brown. Spinnerets: orangey/yellowish. Legs: grayish in situ (Fig. 11B), yellowish ex situ (Figs 11D, 13B)/yellowish tan (Figs 11A, 12F, 13A), Cx anteriorly with dark mark, Tr with dark dot, Fm I basally with dark markings anteriorly, proximally with annulation with slightly dusky area, dusky area at Fm-Pt joint, Pt with dark annulation at Pt-Ti joint, Ti with dusky annulation with darker edges, dark annulation at Ti-Mt joint, Mt with dark annulation at Mt-Ta joint, Ta tip dusky; Fm ventrally with flat, white setae enlarged distally, spines dark at base, lightened to orange distally, sometimes darkened at tip (Fig. 11E); spination leg I Fm d 1-1-1, pr 1-1-0, Ti v 2-2-2-2-2-2, Mt v 2-2-2-2; leg II Fm d 1-1-1, Ti v 2-2-2-2-2-2, Mt v 2-2-2-2; leg III Fm d 1-1-1; leg IV Fm d 1-1-1; leg formula 3241; measurements leg I 9.36 (2.77, 1.07, 2.42, 1.97, 1.13); leg II 10.81 (3.40, 1.01, 2.93, 2.26, 1.21); leg III 11.14 (3.54, 1.09, 2.82, 2.35, 1.34); leg IV 10.38 (3.26, 1.02, 2.55, 2.30, 1.25). Palp: spination Fm d 0-1-3; 2.39 (0.77, 0.38, 0.54, 0.70); claw with five teeth. Epigyne: EP squarish, rounded or truncate anteriorly; MF unsclerotized, diamond to oval shaped, with EWs; LLs distinct, abutting one another posteriorly, COs located at EWs; Endogyne: CDs asymmetrical, unsclerotized at origin, forming one large coil dorsally, sclerotized at point where they coil ventrally, tortuous; ABs are difficult to see; S located posteriorly, FDs long, extended anterolaterally, small pdf (Figs 10C, 12B–D, 10G, 13D).

**Male.** Unknown.

##### Variation.

(*n* = 4) The epigyne and endogyne of each individual differ in EW size and shape, MF shape, overall shape of the EP, anterior sclerotized portion of the CDs, the direction of ducts, the number of coils, where and how the unsclerotized portion connects to the sclerotized portion, size and shape of pdf, and size and shape of FDs (Figs 12A–D, 10F, G, 13C, D). They are different enough that one might consider them separate species had they not been collected from the same place at the same time and did not have independent molecular data supporting the hypothesis that they are the same species. The sample sel_1342 is more reddish brown dorsally/brown. Body length range: 4.40–4.71.

##### Etymology.

The species name is derived from the name of the type locality. Nitmiluk is the Jawoyn name for Katherine Gorge and means “Cicada Place”. Noun in apposition.

##### Distribution.

Known from only Nitmuluk National Park, Northern Territory.

##### Natural history.

*Karaopsnitmiluk* sp. nov. occurs in the Pine Creek subregion of the Pine Creek bioregion. It contains eucalypt woodland and monsoon forest patches. The climate is tropical monsoonal, with most rainfall between November and March. Surveys have been conducted for birds, mammals, reptiles and plants, but there is no information on the bioregion’s invertebrate fauna. For information related to rearing, see Suppl. material 2: tables S1, S2). This species has been collected under rocks and on rock walls at night.

##### Discussion.

The internal ducts and other features of the endogyne are transparent and thus very difficult to see. They were temporarily stained with chlorazol black. Despite the very small sample size, it is notable that the two adult females from Leliyn were penultimate and matured at nearly the exact same time, but there were more months in between the penultimate and adult stages in the specimens from Baruwei. The latter during hot, transitioning to wet, the others, cool, dry and hot, dry to penultimate, and hot and wet to adulthood. Most lived several months after reaching adulthood. The two populations are genetically distinct (Suppl. material 1), but the sample size is small and no consistent morphological differences have been found, so they are described as the same species. Further collecting and rearing of males will help to arrive at conclusions regarding temporal overlap and species boundaries.

#### ﻿ Karaops jawayway sp. nov.

Taxon classificationAnimaliaAraneaeSelenopidae

7D668AB3-2833-55BD-BF31-BAA8F33E305D

https://zoobank.org/95C612EA-22EB-4B38-84FE-5C656DDFCC1F

**Figure 15.**
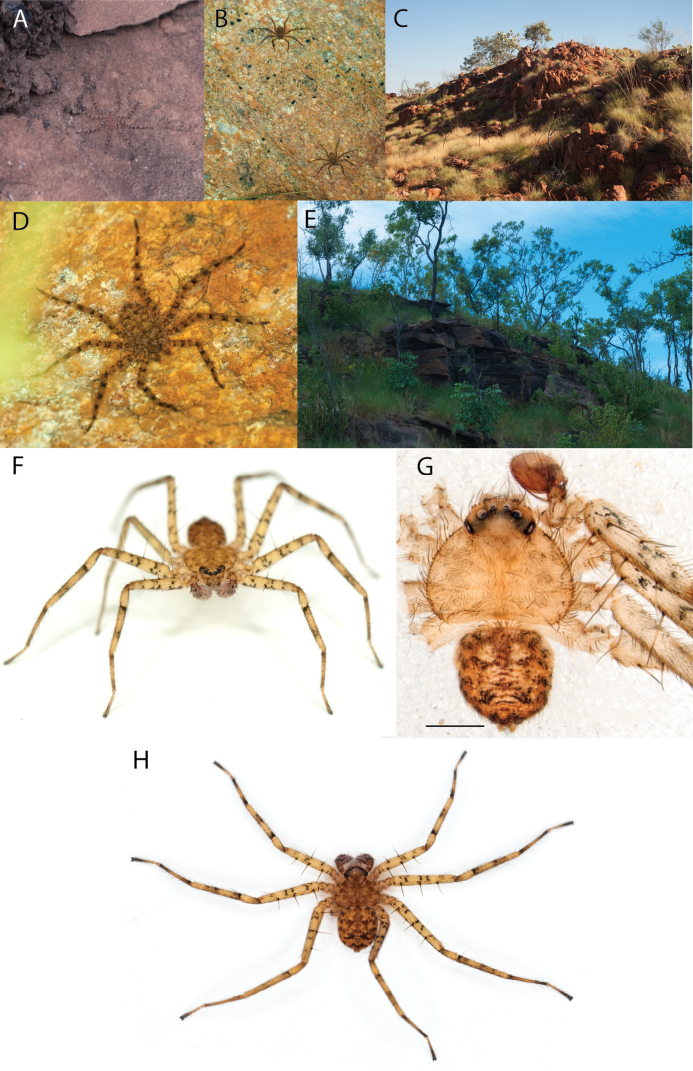
*Karaopsjawayway* sp. nov. and *Karaopsyumbu* of the *dawara* species group **A***Karaopsjawayway* sp. nov., Savannah Way, Northern Territory **B***Karaopsyumbu*, Browns Range, Western Australia, night **C***Karaopsyumbu* habitat, Wolverine pre-pit, Browns Range, Western Australia **D***Karaopsyumbu*, Browns Range, Western Australia, night **E** habitat of *Karaopsjawayway* sp. nov., Savannah Way, Northern Territory **F***Karaopsyumbu*, Browns Range, Western Australia (sel_1293, WAM T155669) **G** same **H** same. Scale bar: 1 mm.

Figs 14A–F , 13E , 15A, E , Maps 1 , 5

##### Material examined.

***Holotype***: Northern Territory • ♀ (reared in captivity); Savannah Way, on road to Roper Bar, out of Limmen NP; vic. 14°45'50.44"S, 134°29'50.47"E; 11 Jun. 2016; S. Crews leg.; at dusk, under rocks on steep, shaley hillside with many, many shed snakeskins; sel_1349; SCC16_075; (MAGNT A004905). **Other material examined**: 1 imm., same data as holotype; sel_1350; (MAGNT A004906).

##### Diagnosis.

Females of *Karaopsjawayway* sp. nov. are similar to those of *K.nitmiluk* sp. nov. and *K.dawara* but can be distinguished by the copulatory ducts. In *K.jawayway* sp. nov., they are wide from their origin at the epigynal windows, narrowed posteriorly, and curved outward within the first third of their length (Fig. 14D, F), whereas in the other two species, the copulatory ducts are of mostly uniform diameter throughout their length, and are straight or only slightly curved anterior to posterior. In *K.jawayway* sp. nov. the sclerotized parts of the copulatory ducts are more than one diameter of the sclerotized ducts away from the epigynal windows, and the ducts are mostly horizontal. In *K.nitmiluk* sp. nov., the sclerotized portion of the copulatory ducts nearly reaches the epigynal windows, and the sclerotized portion of the ducts have coils that generally run anterior to posterior. In *K.dawara*, the ducts are horizontal and quite thin, with longer lengths between turns, and the accessory bulbs are easy to discern, found at the anterior end of the copulatory ducts.

##### Description.

**Female** (holotype). Total length 4.69. Carapace: length 2.07, width 2.56. Chelicerae: promargin with five teeth, two near base of fang smaller than others, retromargin with three teeth. Eyes: AER slightly recurved, PER strongly recurved; diameters AME 0.13, ALE 0.10, PME 0.19, PLE 0.24; interdistances AME–PME 0.04, PME–ALE 0.09, ALE–PLE 0.26, PME–PME 0.78, ALE–ALE 1.12, AME–AME 0.39, PLE–PLE 1.31. Sternum: length 1.23, width 1.30. Abdomen: length 2.62, width 2.38. Color (in life Figs 13E, 14B, 15A/preserved Fig. 14A): Carapace: brown with two pairs of darker marks behind eye area, three pairs of dark marks laterally/orange-yellow, more contrast between dark and pale parts, setose with patches of white setae behind and lateral to eyes. Chelicerae: yellow-brown, paturon with a longitudinal curved mark frontally, setae sparse. Maxillae: whitish orange. Labium: brown, pale distally. Sternum: yellowish brown. Abdomen: dorsally mostly different shades of brown, from dark to pale, white setal patches across anterior, dark brown spots laterally, brown medial line extended laterally ~ 1/3 way from anterior to posterior, then narrowed, then extended outward again posteriorly, white setal tufts at edges of dark brown areas, dark brown at posterior/mostly dark with a few orangish spots; ventrally yellowish. Legs: brownish yellow, Cx II and III with black stripe prolaterally, Tr with dark mark prolaterally, Fm with dark mark/dusky area at Tr-Fm joint, Fm I with pair of dark lines centrally on prolateral side, dusky annulation near Pt, Fm II–IV with jagged pairs of lines basally and medially, with dusky annulation near Pt, Pt dusky at Fm-Pt joint, Ti with dark annulations, one at Pt-Ti joint, other between that and Ti-Mt joint, Mt with dark annulation at either end, Ta tip dark; spination leg I Fm d 1-1-1, pr 1-1-0, Ti v 2-2-2-2-2-2, Mt v 2-2-2-2; leg II Fm d 1-1-1, Ti v 2-2-2-2-2-2, Mt v 2-2-2-2; leg III Fm d 1-1-1; leg IV Fm d 1-1-1; leg formula 3241; measurements leg I 7.37 (2.27, 0.95, 1.87, 1.32, 0.96); leg II 8.92 (2.76, 0.99, 2.44, 1.59, 1.14); leg III 10.44 (3.27, 1.03, 2.68, 2.26, 1.2); leg IV 9.68 (3.18, 0.82, 2.32, 2.29, 1.07). Palp: spination Fm d 0-1-2 (left), 0-1-3 (right); 1.87 (0.55, 0.36, 0.44, 0.52; both claws missing,). Epigyne: EP somewhat rectangular, rounded anteriorly, wider posteriorly than anteriorly; MF with large unsclerotized area and EWs; LLs close but not in contact medially Fig. 14C, E). Endogyne: CDs unsclerotized from EWs, then connected to sclerotized portion anterolaterally; ABs small, difficult to discern; S located posteriorly; FDs directed posterolaterally (Fig. 14D, F).

**Male.** Unknown.

##### Etymology.

Jawayway is the name of a spring near the type locality in the Ngalakgan language of the Ngalakgan people that are the traditional owners of the area. Noun in apposition.

##### Distribution.

Known from only the type locality (Fig. 15E), Savannah Way near Roper Bar, Northern Territory.

##### Natural history.

*Karaopsjawayway* sp. nov. occurs in the McArthur subregion of the Gulf Fall and Uplands bioregion. This region comprises spinifex grasslands with eucalypt woodlands. The climate is monsoonal, with more rainfall in the north than the south. There appears to be little known about the arthropods of the subregion (Bastin 2008). The female matured in the rainiest, hottest part of the year. This species was found under smaller rocks on top of larger rocks on a shaley, steep hillside with an inordinate amount of shed snake skins (Fig. 15A, E, Suppl. material 2: tables S1, S3).

##### Discussion.

The internal ducts and other features of the endogyne are very difficult to see and were temporarily stained with chlorazol black. This species did extremely well in captivity, with sel_1350 molting 11 times and still not reaching adulthood or even penultimate stage (given the size, this was probably the antepenultimate molt). Other research indicates that *Selenops* spp. go through numerous instars before reaching adulthood (18) (unpubl. data). Specimen sel_1350 molted quite often, sometimes slightly more than two weeks apart. For additional information, see the Suppl. material 2. The collection site was quite close to that of *Karaopskennerleyorum* sp. nov.; however molecular data indicate that these two species are not closely related (Suppl. material 1).

#### ﻿ Karaops yumbu

Taxon classificationAnimaliaAraneaeSelenopidae

Crews, 2013

DAFEC8A6-59B8-5ED7-B367-91B597217F53

**Figure 16.**
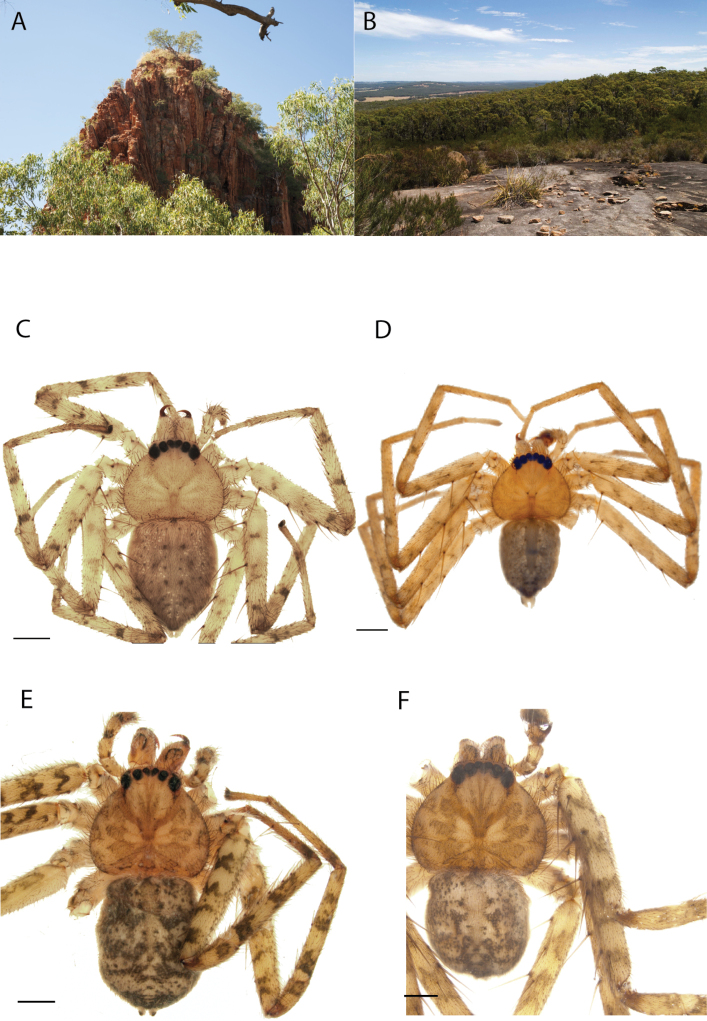
*Karaopsyumbu* and members of the *francesae* species group **A***Karaopsyumbu*, habitat, Sawpit Gorge, Western Australia **B** habitat of *Karaopsfrancesae*, Mt. Lindesay, Western Australia **C***Karaopsfrancesae*, female paratype, east Mount Barren, Fitzgerald River National Park, Western Australia (WAM T54994) **D***Karaopsfrancesae*, male holotype, northeast of slope of West Mount Barren, Fitzgerald River National Park, Western Australia (WAM T54996) **E***Karaopstoolbrunup*, female holotype, Toolbrunup, Stirling Ranges National Park, Western Australia (WAM T76592) **F***Karaopstoolbrunup*, male paratype, Toolbrunup, Stirling Ranges National Park, Western Australia (WAM T62231). Scale bars: 1 mm.

Figs 15B–D, F–H , 15C, F, H , 16A , Maps 1 , 5

Karaops yumbu Crews, 2013: 467, figs 35–37 (♂, examined).

##### New records.

Western Australia • 2 imm.; vic. Hall’s Creek on Tanami Track; 18°25'53.97"S, 127°32'56.81"E; ~ 471 m; 27 May 2016; S. Crews, J. DeJong leg.; under shale; sel_1283–1284; SCC16_060; (WAM T155659–T155660) • 4 imm.; Browns Range, Northern Minerals Camp; 18°52'56.80"S, 128°56'47.34"E; ~ 466 m; 27 May 2016; S. Crews, J. DeJong leg.; on rocks on hillsides above camp at night; sel_1285–1288, 1290; SCC16_061; (WAM T155661–T155664, T155666) • 1 imm.; Wolverine pre-pit; 18°51'37.47"S, 128°56'37.63"E; 28 May 2016; S. Crews, D. Brinsden leg.; under rocks during the day; sel_1289; SCC16_062; (WAM T155665) • 2♂ (reared in captivity), 2 imm.; range west of Browns Range on Duncan Road, mesa south of Kundat Djaru; 18°51'8.35"S, 128°38'33.11"E; 29 May 2016; S. Crews, J. DeJong leg.; at top of hill (but not in scree leading up to top); 29 May 2016; sel_1291–1293; SCC16_063; (WAM T155667–T155669) • 1♂ (reared in captivity), 1 imm.; Sawpit Gorge, south of Hall’s Creek, 18°25'30.44"S, 127°49'13.87"E; 347 m; 29 May 2016; S. Crews, J. DeJong leg.; under rocks up hill near cliff; sel_1294–1295; SCC16_064; (WAM T155670–T155671).

##### Diagnosis.

*Karaopsyumbu* is the only male known from the *dawara* species group; however, it can be differentiated from all other species by having the median apophysis arise from an unsclerotized retromedially oriented extension of the tegulum (Crews 2013: fig. 35).

##### Description.

The description of the male can be found in Crews (2013).

**Female.** Unknown.

##### Distribution.

Known from Sawpit Gorge (Fig. 16A) in the vicinity of Hall’s Creek and Browns Range (Fig. 15C) in northeastern Western Australia.

##### Natural history.

Two males of *Karaopsyumbu* were collected previously in wet pitfalls on a sand plain and a stony rise between January and March. More recently, this species was collected by hand on rocks at night, and under rocks on a steep hill during the day, in May (Fig. 15D, E).

All specimens were collected as immatures and reared in captivity. They were collected in two different subregions of two different bioregions. Specimens from Browns Range and vicinity are from the Tanami Desert subregion of the Tanami bioregion. This area is characterized by sandplains, hills, and ranges with shrub steppe over soft spinifex and wattle scrub and hummock grass over soft spinifex on ranges (Graham 2001a). The climate is arid and tropical with summer rain. The specimens collected closer to Halls Creek are in the Purnululu subregion of the Ord Victoria Plain bioregion. This region is characterized by plains and hills, with grassland, bloodwoods, and snappy gum. The climate is dry hot tropical, semi-arid, with summer rain.

##### Discussion.

In general, this species did well in captivity, only dying after adulthood or in the care of someone else, occasionally while molting. Two lived more than a year in captivity, 16 and 18 months, respectively, molting 6 and 7 times, respectively. January–March, when the holotype and paratypes were collected, is the wettest and hottest part of the year in the Tanami bioregion. In captivity, one reached adulthood in December, the wettest, hottest part of the year, and the other just prior, when it was beginning to get warm and rain more. This is the least known bioregion of the Kimberley (Graham 2001a). There has been some survey work in this area, but no systematic review. It would appear that changes to plant and mammal communities are occurring, probably due to feral cats, stock, fire regimes, and weeds. Although the climate in the Ord Victoria Plain bioregion is slightly different than that of the Tanami, the penultimate male was on track to become an adult in the hottest, wettest time of the year (Suppl. material 2: tables S1, S4, S5).

Despite the search efforts and rearing, there are no female specimens of this species. The palp of this species is unique amongst all the *Karaops* (Crews 2013: figs 35, 36; Figs 15G, 15F, H), but molecular data indicate that it is a member of the *dawara* species group (Suppl. material 1). This is the only male known from this species group; however, molecular data do not indicate this species matches with any females of other species (Suppl. material 1). The females have tortuous copulatory ducts to reach the spermathecae, and this male has an extremely long embolus that corresponds with the long ducts.

### The *francesae* species group

#### ﻿ Karaops francesae

Taxon classificationAnimaliaAraneaeSelenopidae

Crews & Harvey, 2011

D9E4F0AB-7AE1-53D0-AF40-DD3777442A38

Fig. 16B–D , Maps 1 , 6

Karaops francesae Crews & Harvey, 2011: 70, figs 65–68 (♂, ♀, examined).

##### New records.

Western Australia • 1 imm; Cape Le Grand National Park, Lucky Bay camping area, site 4; 33°59'37"S, 122°13'06.9"E; 18 Jun. 2014; 33 m; J.M. Waldock, C.A. Car leg.; hand search; base of granite outcrop; (WAM T134604) • 1♀, 1♂; Fitzgerald River National Park, Mt. Drummond; 33°54'37"S, 119°36'42"E; 7 Oct. 2007; 297 m; M.L. Moir, J. Newell, S. Brett leg.; (WAM T136891) • 1♀; Two Peoples Bay Nature Reserve, Upper Firebreak Gully, Firebreak Track near Little Beach; 34°58'36"S, 118°11'41"E; 27 Nov. 2014; M.S. Harvey, M.G. Rix, M.A. Castalanelli leg.; by hand; under eucalypt bark; (WAM T134761) • 1♀; Fitzgerald River National Park, gully E of Two Bump Hill; 34°00'07.3"S, 119°46'06.3"E; 06 Oct. 2007; J. Newell, S. Comer leg.; under rocks; (WAM T119331) • 1♀; Cape Le Grand National Park, Lucky Bay, granite outcrop, site 7; 33°59'45.1"S, 122°13'11.7"E; 19 Jun. 2014; 29 m; J.M. Waldock, C.A. Car leg.; by hand; under rock on granite; (WAM T133030) • 1♀; same as previous; 44 m; WAM T133031.

##### Diagnosis.

Males of *Karaopsfrancesae* (Fig. 16D) can be distinguished from *K.toolbrunup* by the smaller tegular lobe and the embolus that is at the perimeter of the cymbium (Crews and Harvey 2011: figs 65, 66, 71, 72). Females of *K.francesae* (Fig. 15C) have the ducts between the spermathecae and accessory bulbs coiled and the accessory bulbs extend anteriorly beyond the middle of the genitalia.

##### Description.

The description of the male and female can be found in Crews and Harvey (2011).

##### Distribution.

This species is found along the southern coast of Western Australia, including offshore islands (Maps 1, 6).

##### Natural history.

*Karaopsfrancesae* (Fig. 16C, D) is known from the southwest of Western Australia and has primarily been collected along the coast (Fig. 16B). It is thus far known from three subregions: Southern Jarrah Forest of the Jarrah Forest bioregion and the Fitzgerald and Recherche subregions of the Esperance Plains bioregion. The Southern Jarrah Forest climate is warm Mediterranean, with Jarrah-Marri woodlands interspersed with shrublands. The Esperance bioregion is characterized by scrub and mallee heaths. The vegetation of the Fitzgerald subregion is diverse, with many localized endemics. The Fitzgerald and Recherche subregions both have a temperate Mediterranean climate. The Recherche subregion vegetation is primarily heath, and it gets slightly more rainfall than the Fitzgerald subregion. The subregions are warmest and driest November to March, and coolest and wettest May to August. Adults seem to occur during the transitional times between hot and dry and cool and wet; however, this may reflect when collections are made as it is not optimal to collect during the hottest or wettest times of the year (Suppl. material 2: table S1). This species is often collected beneath granite slabs (Fig. 16B).

##### Discussion.

This species overlaps with two species, *Karaopstoolbrunup*, its sister taxon, and there has been a single collection of *K.jarrit* from within the range. *Karaopstoolbrunup* has thus far only been collected from the Stirling Ranges National Park. It is closely related to *K.francesae* and looks similar, but all specimens collected can be distinguished (i.e., they are not variants). Molecular data show some clades of *K.francesae* that are as different from one another as they are from as *K.toolbrunup*; however, no morphological differences have been found in these groups, and it is possibly due to introgression, intermixing, or the genes used are not good for determining these relationships (Suppl. material 1). These two species comprise the *francesae* species group.

#### ﻿ Karaops toolbrunup

Taxon classificationAnimaliaAraneaeSelenopidae

Crews & Harvey, 2011

98742690-82B0-5929-B078-55C2B3DFF3DE

Fig. 16E, F , Maps 1 , 6

Karaops toolbrunup Crews & Harvey, 2011: 74, figs 69–72, 95 (♂, ♀, examined).

##### New record.

Western Australia • 1 imm.; Stirling Range National Park, Bluff Knoll, track to summit; -34.37583, 118.2544; 15 Apr. 2015; M.S. Harvey, M.G. Rix, N.J. Tatarnik, A. Coles leg.; under rock montane heathland; (WAM T135856).

##### Diagnosis.

See diagnosis for *Karaopsfrancesae* above.

##### Description.

The description of the male and female can be found in Crews and Harvey (2011).

##### Distribution.

This species is known only from the Stirling Ranges National Park (Maps 1, 6).

##### Natural history.

The Stirling Ranges are within the Fitzgerald subregion of the Esperance bioregion. There is more relief in this part of the subregion, with Bluff Knoll the highest peak in the south of Western Australia. It also has a slightly wetter and cooler climate, with rare ecosystems, like the Stirling Range Montane Thicket and Heath of the SW Botanical Province. This species has been collected under rocks.

##### Discussion.

*Karaopstoolbrunup* (Fig. 16E, F) is found within the range of the similar, closely related and more widespread *K.francesae*. The mountaintops and gulleys of the Stirling Ranges are thought to be refugia for some organisms (Comer et al. 2001; Rix, Roberts and Harvey 2009) (Suppl. material 2: table S1).

### The Kimberley species group

#### ﻿ Karaops keithlongbottomi

Taxon classificationAnimaliaAraneaeSelenopidae

Crews & Harvey, 2011

802275FD-E49A-519D-985F-CA1C85E4DBF6

**Figure 17.**
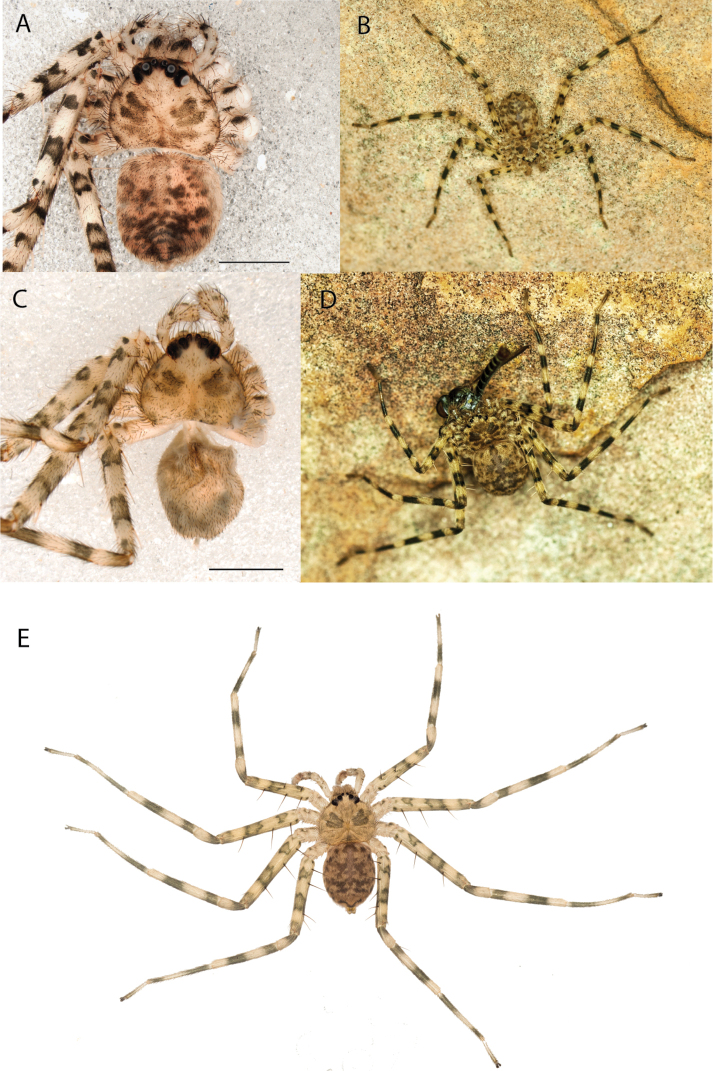
Members of the Kimberley species group **A***Karaops* sp., Kelly’s Knob, Western Australia **B***Karaops* sp., Kelly’s Knob, Western Australia **C***Karaops* sp., Keep River National Park, Northern Territory (sel_1320, MAGNT A004876) **D***Karaops* sp. eating a fly, Kelly’s Knob, Western Australia **E***Karaopskeithlongbottomi*, Bujani (Little Merten’s Falls), Mitchell River National Park, Western Australia (sel_1244, WAM T155620).

**Figure 18.**
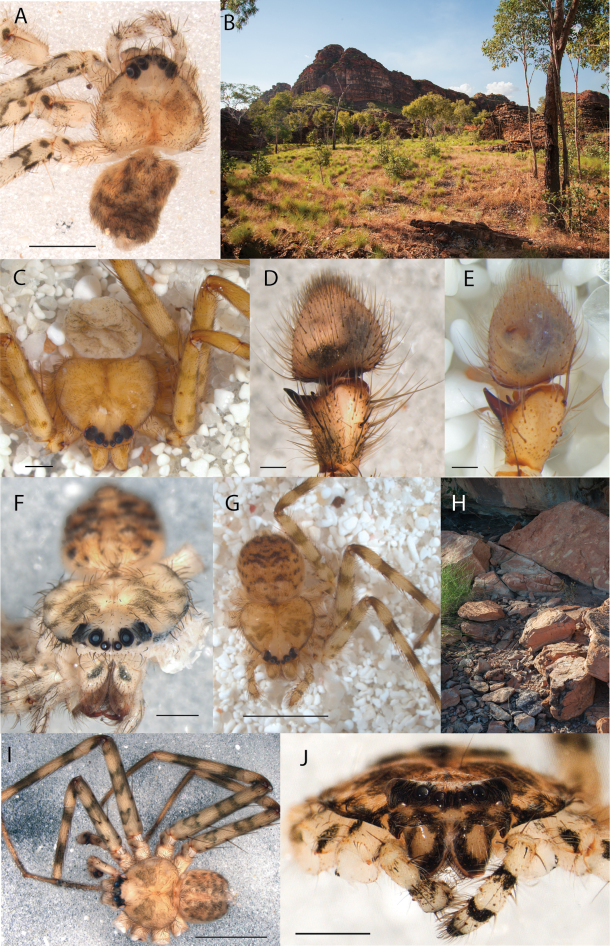
Members of the Kimberley species group **A***Karaops* sp., Keep River National Park, Northern Territory (sel_1315, MAGNT A004871) **B** habitat, Keep River National Park, Northern Territory **C***Karaopskeithlongbottomi*, holotype male, Drysdale River Station, Western Australia (WAM T55002) **D***Karaopskeithlongbottomi*, palp, dorsal, Bujani (Little Merten’s Falls), Mitchell River National Park, Western Australia (sel_1244, WAM T155620) **E***Karaopskeithlongbottomi*, holotype male, palp, dorsal, Drysdale River Station, Western Australia (WAM T55002) **F***Karaopsdejongi* sp. nov., holotype female, RAAF Boab Quarry, Western Australia (sel_1273, WAM T155649) **G***Karaopsumiida*, holotype female, Irvine Island, Western Australia (WAM T110393) **H** habitat, Keep River National Park, Northern Territory **I***Karaopskeithlongbottomi*, Bujani (Little Merten’s Falls), Mitchell River National Park, Western Australia (sel_1244 WAM T155620) **J** same, face showing marks on chelicerae. Scale bars: 0.5 mm (**D, E**); 1 mm (**A, C, F, J**); 5 mm (**G, I**).

**Figure 19.**
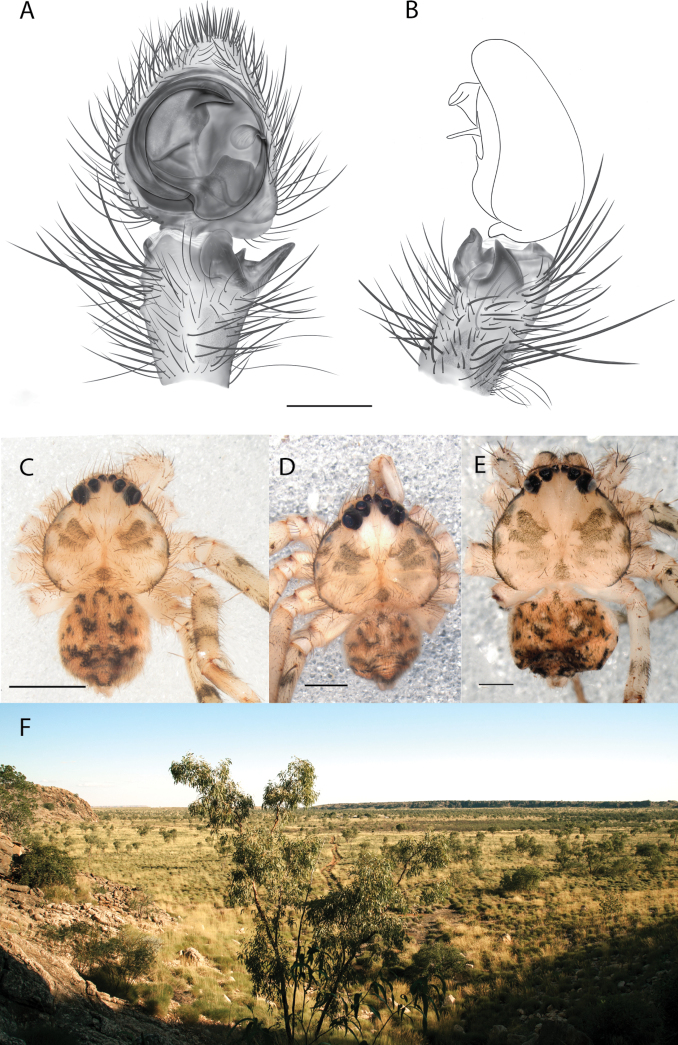
Members of the Kimberley species group **A***Karaopskeithlongbottomi*, palp, ventral, Bujani (Little Merten’s Falls), Mitchell River National Park, Western Australia (sel_1244, WAM T155620) **B** same, retrolateral **C***Karaops* sp., Mimbi Caves, Western Australia (sel_1276, WAM T155652) **D***Karaopsdejongi* sp. nov., paratype male, RAAF Boab Quarry, Western Australia (sel_1272, WAM T155648) **E***Karaops* sp., Geike Gorge, Western Australia (sel_1267, WAM T155643) **F** habitat, Mimbi Caves, Western Australia. Scale bars: 0.5 mm (**A, B**); 1 mm (**C–E**).

Figs 17E , 18C–E , 18I, J , 19A, B , Maps 1 , 7

Karaops keithlongbottomi Crews & Harvey, 2011: 34, figs 19–20 (♂, examined).

##### Material examined.

Western Australia • ♂ (reared in captivity), 6 imm.; Mitchell River National Park, Bujani (Little Merten’s Falls); -14.82218, 125.7131; 21 May 2016; J. DeJong leg.; on rocks at night; sel_1244–1245; SCC16_047; (WAM T155620–T155621).

##### Emended diagnosis.

Given the number of the new, morphologically similar species described here, the diagnosis is updated, and new illustrations are provided for ease of identification. The male is similar to other members of the group but is most easily differentiated by the RTA (Figs 18D, E, 19A, B). In ventral view, the vRTA is somewhat shaped like the tube and bell of a sousaphone, rather than the oblong or spoon-shaped vRTA of other species. The dRTA is short and plicate.

##### Description.

The description is updated here based on the recently collected additional male. The full description of the male can be found in Crews and Harvey (2011).

**Male** (sel_1244, WAM T155620). Color (in life Fig. 17E/preserved Fig. 18C, I, J): Carapace: pale tan-yellow with two dark marks mediolaterally on either side of fovea and a small, dark mark posteromedially/orangish brown, darker markings not as conspicuous, with dark, slender setae, darker where two dark marks located, distributed somewhat densely but integument still visible beneath, a few dark, stiff setae posteriorly. Chelicerae: yellowish tan, paturon with dark edges (Fig. 18J)/orangish with dark marks less conspicuous than in life. Abdomen: pale brown with dark brown spots anteriorly and laterally, slightly dark in cardiac area, two dark patches medially, chevrons posteriorly, black, undulate mark posteriorly/orangish yellow, with spots and markings less conspicuous, long, dark, slender setae. Legs: pale yellowish white, Cx with dark marks prolaterally, Tr, Fm with small black dots at Tr-Fm joint, dark marks paler centrally, Fm with dark annulation at Fm-Pt joint, Pt with dark annulation at Pt-Ti joint, Ti with dark annulation mid-Ti and one closer to the Ti-Mt joint, Mt with annulation at both ends, Ta dusky distally/orangish brown, dark marks less conspicuous; spination leg I Ti v 2-2-2-2-2-2, Mt v 2-2; leg II Ti v 2-2-2-2-2, Mt v 2-2-2.

**Female**. Unknown.

##### Distribution.

Known from Ngauwudu (Mitchell Plateau), Northern Kimberley, Western Australia (Map 7).

##### Natural history.

Very little is known about the type other than it was collected from Drysdale River Station, late 1995. The precise locality, time of year, and microhabitat are unknown. The abdomen of the type is damaged (Fig. 18C). The new specimens were collected on rocks at night at Bujani (Little Merten’s Falls), Mitchell River National Park. Males and penultimate males are known from the drier, cooler part of the year. This species is known from the Mitchell subregion of the North Kimberley bioregion. The area has a tropical savannah climate, and it is very wet in the wet season, often rendering roads impassable (Graham 2001b).

##### Discussion.

This specimen and the penultimate female are quite large – the largest *Karaops* species known. The length of the holotype is unknown due to abdominal damage, but the carapace is 3.3. The length of the newly collected specimen is 8.41, with a carapace length of 4.64. The female remains undescribed. Although we only know of two areas where it is found, the new data do help to provide a bit more knowledge of its range, highlighting the importance targeted collecting and rearing to adulthood (Suppl. material 2: tables S1, S6).

#### ﻿ Karaops dejongi sp. nov.

Taxon classificationAnimaliaAraneaeSelenopidae

C4330253-72BA-59AA-B922-F3CD97BEA4B2

https://zoobank.org/C4131AB8-08AC-46F3-B5A9-F2C88FE337BA

**Figure 20.**
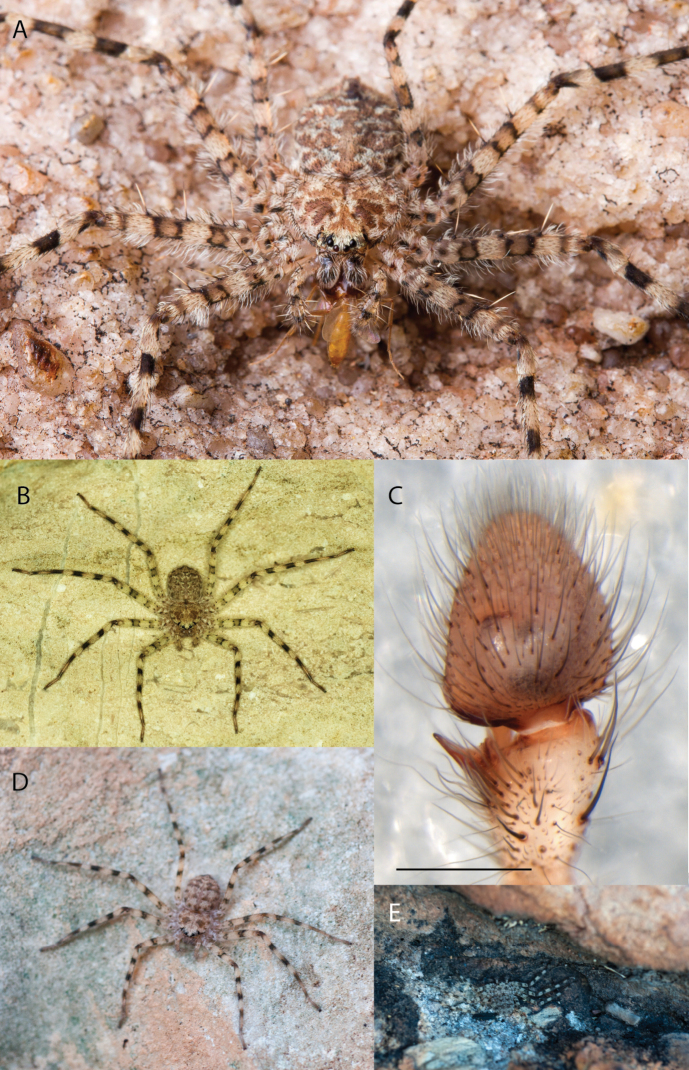
Members of the Kimberley species group **A***Karaopsdejongi* sp. nov., eating a hymenopteran, RAAF Boab Quarry, Western Australia (photo: J. DeJong) **B***Karaops* sp., Geike Gorge, Western Australia **C***Karaopsdejongi* sp. nov., paratype male, palp, dorsal, RAAF Boab Quarry, Western Australia (sel_1272, WAM T155648) **D***Karaops* sp., Mimbi Caves, Western Australia **E***Karaops* sp., Keep River National Park, Northern Territory. Scale bar: 0.5 mm.

**Figure 21.**
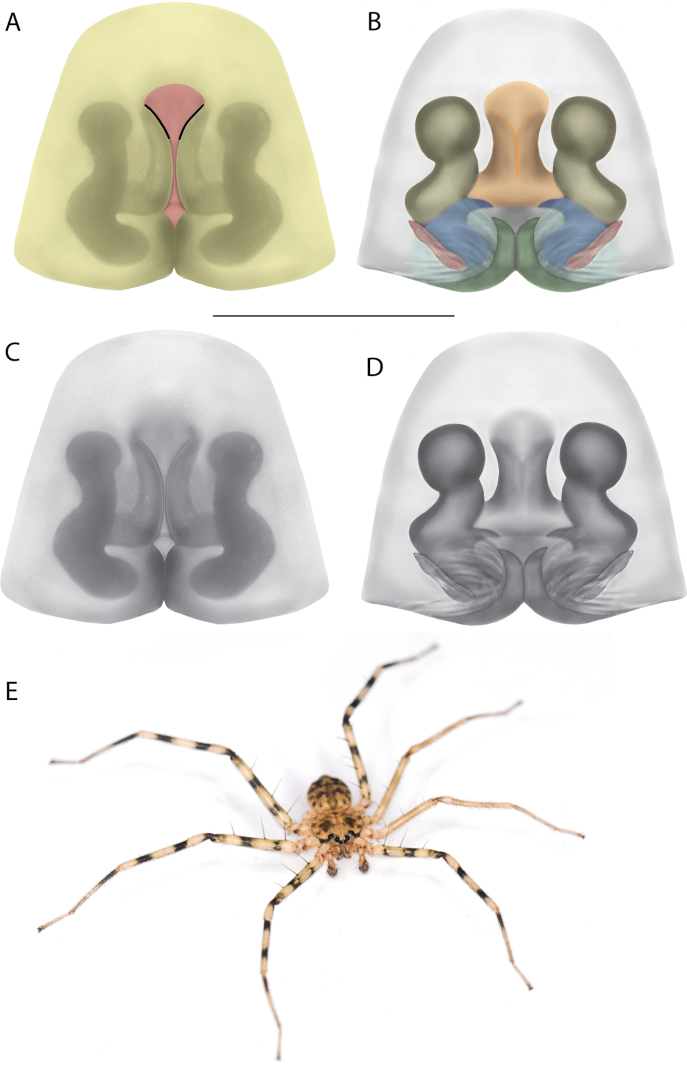
*Karaopsdejongi* sp. nov., RAAF Boab Quarry, Western Australia **A** holotype female, epigyne, (sel_1273, WAM T155649); black = copulatory openings, pink = median field/atrium, yellow = lateral lobes/epigynal plate **B** same, endogyne; yellow = accessory bulbs, blue = spermathecae, orange = atrium/copulatory ducts; red = fertilization ducts, green = posterodorsal fold, turquoise = uterus externus **C** same, epigyne **D** same, endogyne **E** paratype male (sel_1272, WAM T155648). Scale bar: 0.5 mm.

**Figure 22.**
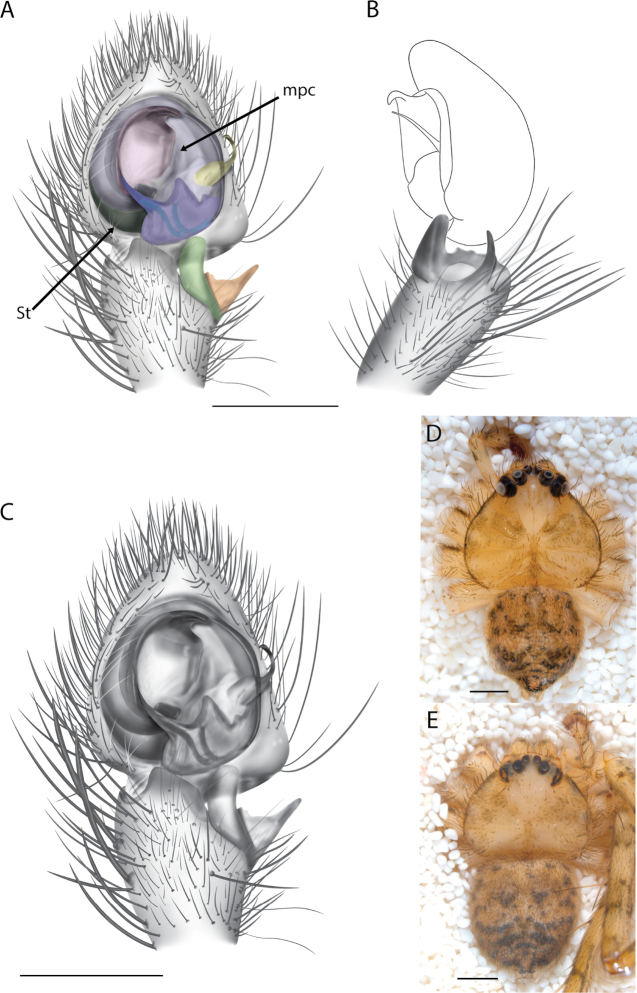
*Karaopsdejongi* sp. nov. and *Karaopsdalmanyi* sp. nov. of the Kimberley species group **A***Karaopsdejongi* sp. nov., paratype male, palp, ventral, RAAF Boab Quarry, Western Australia (sel_1272, WAM T155648); green = vRTA, orange = dRTA, light gray-blue = tegulum/tegulum sheath, blue-violet = tegular lobe, blue = spermophor and embolus, pink = conductor, yellow = median apophysis, dark gray-green = subtegulum. St = subtegulum, mpc = medial part of the conductor **B** same, retrolateral **C** same, ventral **D***Karaopsdalmanyi* sp. nov., adult male, Dalmanyi (Bell Gorge), Western Australia (sel_1234, WAM T155610) **E***Karaopsdalmanyi*, (sel_1237, WAM T155613). Scale bars: 0.5 mm (**A–C**); 1 mm (**D, E**).

Figs 18F , 19D , 20A, C , 21A, B, D, E , 22A–C , Maps 1 , 7

##### Material examined.

***Holotype***: Western Australia • ♀ (reared in captivity); RAAF Boab Quarry camping area; 17°54.56.60"S, 125°18'5.94"E; 24 May 2016; S. Crews, J. DeJong leg.; on limestone rocks at night; sel_1273; SCC16_055; (WAM T155650). ***Paratype***: ♂ (reared in captivity); same data as previous; sel_1272; (WAM T155649). **Other material examined**: 2 imm.; same data as previous; sel_1270–1271; (WAM T155646–T155647) • 4♀, 4♂; RAAF Boab Quarry; 17°54'59.14"S, 125°18'05"E; 9 Sep. 2018; J. DeJong leg.; (WAM T147699–147706).

##### Diagnosis.

**Figure 23.**
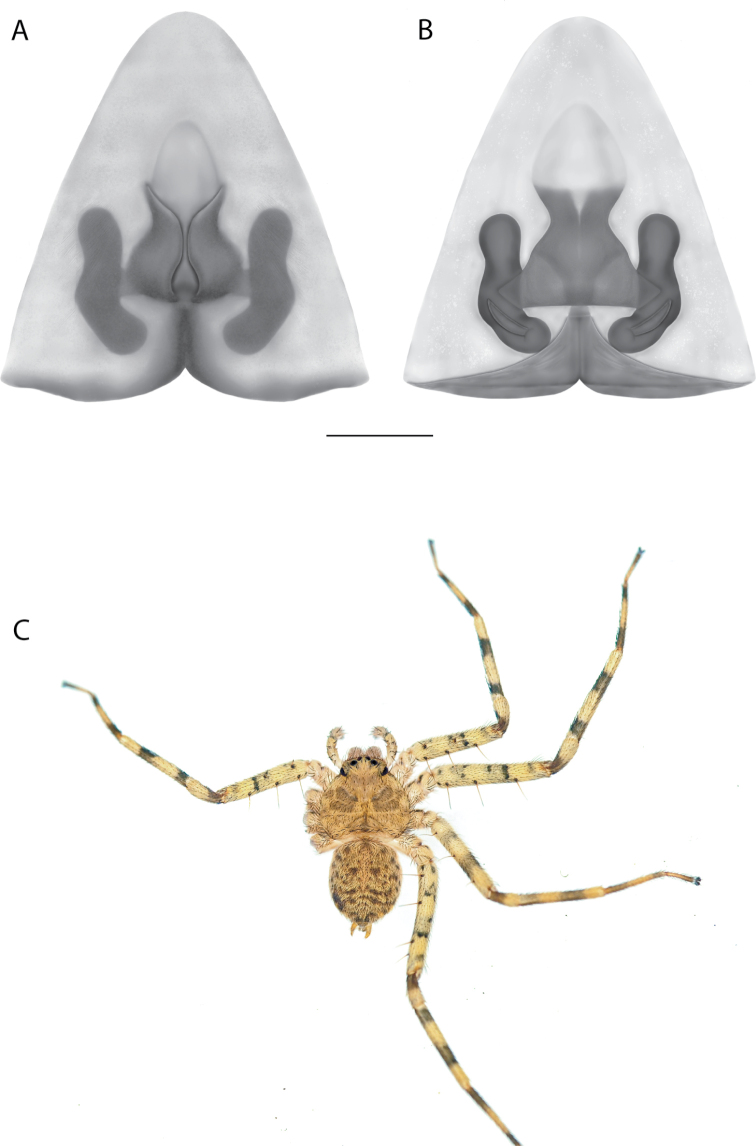
*Karaopsdalmanyi* sp. nov., holotype female, Dalmanyi (Bell Gorge), Western Australia (sel_1236, WAM T155612) **A** epigyne **B** endogyne **C** adult female. Scale bar: 0.2 mm.

The female of *Karaopsdejongi* sp. nov. is most similar to *K.dalmanyi* sp. nov. in that there is a slit-like opening medially between the lateral lobes (Figs 21A, 23A, B). However, the epigynal plate is more triangular in *K.dalmanyi* sp. nov. and more rounded anteriorly in *K.dejongi* sp. nov., the median depression anterior to the atrium in *K.dalmanyi* sp. nov. is much larger than the median depression in *K.dejongi* sp. nov., in dorsal view, the atrium and copulatory ducts are shaped like an inverted T in *K.dejongi* sp. nov., whereas they are narrowed medially in *K.dalmanyi* sp. nov.

The male can be distinguished from the other members of the group by the RTA (Figs 20C, 22A–C). The dRTA is extended prolaterally into a keel that is jagged along the anterior margin (Fig. 22B). The vRTA is long and slender, arched prolaterally.

##### Description.

**Female** (holotype). Total length 5.28. Carapace: length 2.71, width 3.04. Chelicerae: promargin with three teeth, retromargin with two teeth. Eyes: AER slightly recurved, PER recurved; diameters AME 0.16, ALE 0.14, PME 0.25, PLE 0.33; interdistances AME–PME 0.07, PME-PLE 0.16, ALE–PLE 0.31, PME–PME 0.99, ALE–ALE 1.43, AME–AME 0.48, PLE–PLE 1.79. Sternum: length 1.43, width 1.52. Abdomen: length 2.57, width 2.17. Color: Carapace: yellowish white with dark patches extending posteriorly from PLEs, two dark patches medially on either side of fovea, three pairs of lateral dark spots and single medial dark spot posteriorly, white, flattened setae around eyes and anterolateral margin, some short, thick, sparse setae and long, thick, sparse setae, the latter mostly toward the posterior and anterior, darker, thin setae distributed evenly, not dense. Chelicerae: whitish yellow, paturon with longitudinal curved mark frontally, dark setae more prominent anteriorly. Maxillae: whitish yellow. Labium: tan, pale distally. Sternum: whitish yellow. Abdomen: dorsally orangish brown with dark spots around margin, two dark patches anteriorly, several dark chevrons extending to posterior; ventrally yellow-gray. Legs: whitish yellow, Cx and Tr with dusky areas proximally; Fm with annulations at Fm-Tr joint and medially, Pt with annulations at Fm-Pt joint, Ti with annulations basally and distally, Mt with annulations basally and distally; Ta dusky; spination leg I Fm d 1-1-1, pr 1-1, Ti v 2-2-2-2-2-2-2, Mt v 2-2-2; leg II F d 1-1-1; Ti v 2-2-2-2-2, Mt v 2-2-2; leg III Fm d 1-1-1, pr 2-0-0; leg IV Fm d 1-1-1, Ti v 2-2; leg formula 3421; measurements leg I 12.09 (3.07, 1.34, 3.50, 2.75, 1.43); leg II 12.70 (3.54, 1.46, 3.57, 2.86, 1.25); leg III 14.59 (4.63, 1.27, 3.76, 3.41, 1.51); leg IV 13.17 (4.39, 1.12, 3.17, 3.12, 1.37). Palp: spination Fm 0-1-2; 2.46 (0.70, 0.54, 0.61, Ta 0.63); claw with ~ 7 teeth. Epigyne: EP arched; MF with At; LLs separated by a narrow, longitudinal slit, widens anteriorly; COs in At (Fig. 21C). Endogyne: At narrow with nearly straight sides, leading posteriorly to wide, lateral CDs, At+CDs shaped like inverted T; ABs+S abruptly bent medially in lateral direction, ABs round, do not extend beyond atrium anteriorly; S allantoid; long FDs directed anterolaterally; small pdf medially.

**Male** (paratype) (Figs 19D, 21E). Total length 4.98. Carapace: length 3.01, width 3.21. Chelicerae: promargin with three teeth, retromargin with two teeth. Eyes: AER recurved, PER strongly recurved; diameters AME 0.14, ALE 0.11, PME 0.25, PLE 0.42; interdistances AME–PME 0.06, PME–ALE 0.10, ALE–PLE 0.25, PME–PME 0.91, ALE–ALE 1.24, AME–AME 0.47, PLE–PLE 1.68. Sternum: length 1.26, width 1.68. Abdomen: length 1.97, width 2.09. Color (in life Figs 20A, 21E/preserved Fig. 18F): Carapace: yellow-tan with two black patches anteromedially, three pairs of dark patches on lateral margins, one dark spot posteromedially, black patches from PLEs laterally along front of carapace (Fig. 21E)/orangish brown, markings conspicuous. Chelicerae: brownish, paturon with a longitudinal curved mark frontally, like a little mustache, sparse, dark setae. Maxillae: whitish. Labium: tan, pale distally. Sternum: orangish white. Abdomen: dorsally hirsute, brownish yellow with dark spots anteriorly, laterally, cardiac area brownish, ending in point medially on abdomen, two triangular marks anteromedially, chevron mark at posterior of cardiac mark, two dark horizontal lines, dusky area, dark, wavy line at posterior/reddish orange, dark marks conspicuous, edges of markings less distinct; ventrally yellowish brown. Legs: yellowish, Tr with dark mark prolaterally, Fm with dark marks basally, center pigmentation paler, an annulation distally, dusky at Fm-Pt joint, Pt with dark annulation at Fm-Pt joint, Ti with dark annulation at Pt-Ti joint and medially, Mt with dark annulation at Ti-Mt joint, slightly dusky at Mt-Ta joint; spination leg I Fm d 1-1-1, pr 1-1-0, Ti v 2-2-2-2-2, pr 0-0-1, rl 0-0-1, Mt v 2-2-2; leg II Fm d 1-1-1, Ti v 2-2-2-2, Mt v 2-2-2 (leg regrown); leg III Fm d 1-1-1 (leg regrown); leg IV Fm d 1-1-1, rl 0-1-1, Ti v 2-2, pr 2-2, rl 1-1, Mt v 2-2; leg formula 4132; measurements leg I 13.91 (3.82, 1.28, 3.76, 3.51, 1.56); leg II 11.79 (3.64, 1.06, 3.63, 2.51, 0.96); leg III 13.80 (3.42, 1.16, 3.94, 4.08, 1.20); leg IV 13.93 (3.88, 1.44, 4.08, 3.39, 1.14). Palp: spination Fm d 0-1-2; 2.91 (1.20, 0.49, 0.51, 0.71); dRTA long, pointed, prolaterally, connected to vRTA via keel, keel jagged, teeth seen in v and rl views (Fig. 22B, C), vRTA in v view long, narrow, arched prolaterally; rbcp smallish; Cy triangular; C crescent shaped prolaterally, with short hook anteriorly, extends just beyond mpc, with CS, TS around CS (Fig. 22A, C); E hook shaped, arises from medium-sized TL with a slightly plicate extension on anterior margin, begins at approximately 6 o’clock, ends at approximately 12 o’clock, closer to middle of bulb than following edge of Cy; MA only lightly sclerotized basally, sclerotized distally, base quadrangular with long, narrow branch (Fig. 22B, C).

##### Etymology.

This species is named after Jordan DeJong, world’s best selenopid finder/collector (and a darn good gecko spotter). Noun in the genitive case.

##### Distribution.

Known from only the type locality, RAAF Boab Quarry, Kimberley region, Western Australia (Map 7).

##### Natural history.

*Karaopsdejongi* sp. nov. is found in the Fitzroy Trough subregion of the Dampierland bioregion. The climate is semi-arid with summer rainfall. Vegetation includes woodlands of pindan, boab, and eucalypts, with some rainforest patches and hummock grassland (Graham 2001c). Penultimate males and females were collected in May, reaching maturity in June and September, respectively, the cooler, drier times of the year (Suppl. material 2: tables S1, S7).

##### Discussion.

According to IBRA, the area is experiencing a loss of species and turnover due to changes in vegetation structure (Graham 2001c).

#### ﻿ Karaops umiida

Taxon classificationAnimaliaAraneaeSelenopidae

Crews, 2013

6487D286-350A-59EE-B5B6-146D414B5B1C

Fig. 18G , Maps 1 , 7

Karaops umiida Crews, 2013: 447, figs 1, 2. (♀, examined; males mismatched, see below).

##### Diagnosis.

In light of the new species described here, a new diagnosis is provided. This species can be distinguished from all other members of the group by the genitalia. On the epigyne, the atrium where the copulatory openings are located is u-shaped. The copulatory ducts are directed posterolaterally and there is no larger receptacle between them. The accessory bulbs do not extend anteriorly past the small atrium leading to the ducts. In Crews 2013 (fig. 2), the accessory bulbs were incorrectly labeled as the spermathecae.

##### Description.

The description of the female can be found in Crews (2013).

**Male.** Unknown.

##### Distribution.

Known from only the type locality, Irvine Island, in the Buccaneer Archipelago, Western Australia (Map 7).

##### Natural history.

The specimen was collected in the cooler, drier time of the year, under rocks on boulder scree during the day. Irvine Island is located in the Mitchell subregion of the Northern Kimberley bioregion. It is savannah woodland with small patches of monsoonal rainforest in the region, and the climate is hot tropical with lots of rain in the wet season (Suppl. material 2: table S1).

##### Discussion.

The holotype (Fig. 18G) is from Irvine Island. Two males were collected nearby with a label indicating they had been collected on Margaret Island, though the coordinates placed them on Conilurus Island, and this is where they were actually collected (R. Teale, pers. comm.). Due to proximity and general similarities of the habitus of the three specimens, the males were described as *Karaopsumiida*. Recent targeted collecting, however, has uncovered a sizeable species radiation in the Kimberley, and the distribution and phylogenetic relationships of these species indicate there are more to come (Figs 20D, E, 26D, Suppl. material 1, Map 7). In a molecular analysis of *Karaops* species (Suppl. material 1), the holotype female of *K.umiida* from Irvine Island is not recovered with the males from Conilurus Island. The males are recovered near *K.dalmanyi* sp. nov. but are morphologically different. Based on these data, the males of *K.umiida* were misidentified and considered to be unknown, and the males previously misidentified as *K.umiida* are now *K.conilurus* sp. nov. Although Conilurus Island is nearby to Irvine Island, they are on different geological formations.

#### ﻿ Karaops dalmanyi sp. nov.

Taxon classificationAnimaliaAraneaeSelenopidae

DB2B36B5-2037-58BB-AB7B-C88A87C7E361

https://zoobank.org/421A1161-15FF-4C1B-BAD1-48B429F74DB5

Figs 22D, E , 23A–C , 24A–C , 25A–F , 26A–C , 27A, B, E, F , Maps 1 , 7

##### Material examined.

***Holotype***: Western Australia • ♀; Dalmanyi (Bell Gorge), vic. Dulundi (Silent Grove) Campground, 8 km from Imintji Roadhouse; 16°59'58.28"S, 125°12'25.33"E; ~ 319 m; 20 May 2016; S. Crews, J. DeJong leg.; on rocks and a tree at night in gorge; sel_1236; (WAM T155612). ***Paratype***: ♂ (reared in captivity); same data as previous; sel_1233; (WAM T155609). **Other material examined**: 2 ♂ (reared in captivity), 2 imm.; same data as previous; sel_1234–1235, 1237–1238; (WAM T155610–T155611, T155613–T155614).

##### Diagnosis.

**Figure 24.**
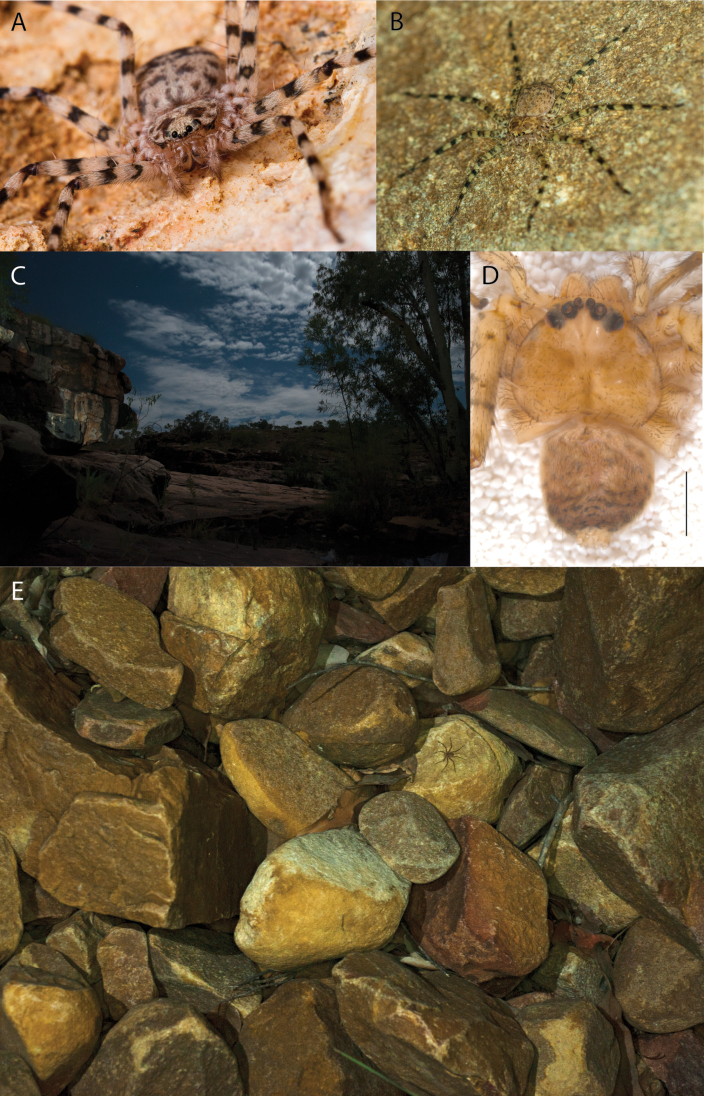
*Karaopsdalmanyi* sp. nov., Dalmanyi (Bell Gorge), Western Australia **A** adult female (photograph: J. DeJong) **B** same **C** Dalmanyi at night, type locality **D** holotype female (sel_1236, WAM T155612) **E** on rocks at night. Scale bar: 2 mm.

The female of *Karaopsdalmanyi* sp. nov. (Figs 23C, 24A) can be distinguished from the other species in the Kimberley group by the genitalia. In dorsal view, the atrium that leads to the copulatory ducts narrows, then widens, leading to the spermathecae and accessory bulbs. The atrium extends anteriorly beyond the accessory bulbs (Fig. 23A, B). In all other species, the sides of the atrium are straight or only widen, and the atrium does not extend anteriorly beyond the accessory bulbs.

The male can be distinguished from the other members of the group except for *Karaopsalanlongbottomi* by the conductor (Fig. 26B). The tip is extended toward the retrolateral side of the palp, beyond the medial part of the conductor. In *K.alanlongbottomi*, the median apophysis is small, the tegular lobe is small and located at ~ 3–4 o’clock, and the embolus follows the edge of the cymbium. In *K.dalmanyi* sp. nov., the median apophysis is large, the tegular lobe is not small and is located at 5–6 o’clock, and the embolus is more toward the middle than the edge of the cymbium.

##### Description.

**Female** (holotype). Total length 7.01. Carapace: length 3.90, width 4.39. Chelicerae: promargin with three teeth, retromargin with two teeth. Eyes: AER slightly recurved, PER recurved; diameters AME 0.15, ALE 0.11, PME 0.21, PLE 0.32; interdistances AME–PME 0.05, PME-PLE 0.16, ALE–PLE 0.29, PME–PME 0.88, ALE–ALE 1.37, AME–AME 0.43, PLE–PLE 1.70. Sternum: length 2.20, width 2.10. Abdomen: length 3.11, width 3.36. Color (in life Figs 23C, 24A, B/preserved Fig. 23D): Carapace: golden brown with two dark patches medially on either side of fovea/tan to orange, dark patches more orangish, not as distinct as in live animal, with short, thick, sparse but evenly distributed, pale and darker colored slender setae, setae around eyes. Chelicerae: yellowish white, paturon with a longitudinal, curved, dark mark anteriorly, like a little mustache/mark faded. Maxillae: yellowish white. Labium: orangish, pale distally. Sternum: yellowish white, with pale setae laterally, darker toward anterior. Abdomen: dorsally golden tan with dark spots anteriorly and laterally, not dark in cardiac area, two dark patches medially, chevrons posteriorly/orange-brown with black spots; ventrally yellowish gray. Spinnerets: anterior orange-tan, without marks. Legs: yellow, Cx, Tr with dark marks prolaterally; Fm with dark flecks nearly connecting into full annulations, but not encircling entire leg, dark annulation at Fm-Pt joint, Ti with dark annulation at Pt-Ti joint, another distal to that, but not at joint, one at Ti-Mt joint, one at Mt-Ta joint; Ta tip dark; macrosetae orangish basally, darker distally; spination leg I Fm d 1-1-1, pr 1-1, Ti v 2-2-2-2-2, Mt v 2-2-2; leg II Fm d 1-1-1, Ti v 2-2-2-2-2, Mt v 2-2-2; leg III missing; leg IV missing; measurements leg I 14.94 (4.24, 1.80, 4.29, 3.17, 1.43); leg II 17.14 (5.43, 1.93, 4.50, 3.86, 1.43); leg III missing; leg IV missing. Palp: spination Fm d 0-1-3; 3.73 (1.16, 0.75, 0.75, 1.07); claw with ~ 9 teeth. Epigyne: EP triangular; MF with sclerotized area medially, more broadly separated, then narrowed, with atrium; LLs with posterior suture, COs in atrium. Endogyne: At conspicuous in dorsal view, CDs short, wide, lead to ABs, S; ABs do not extend beyond anterior part of At; S + ABs allantoid; FDs directed anterolaterally; small pdf.

**Figure 25.**
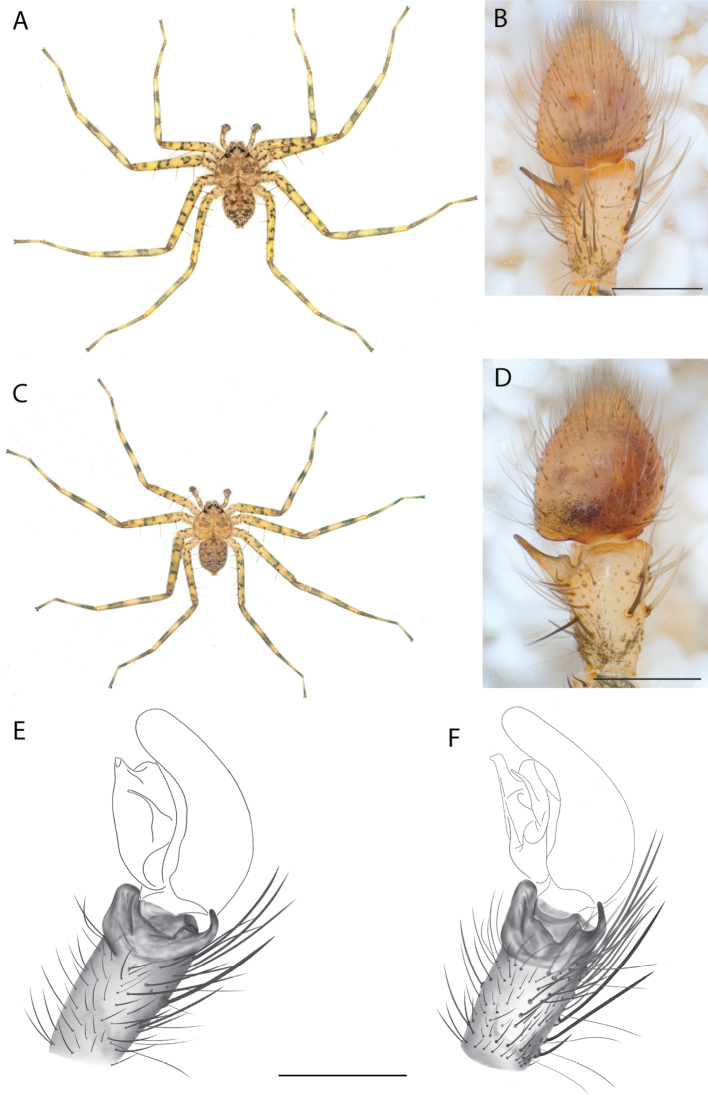
Males of *Karaopsdalmanyi* sp. nov., Dalmanyi (Bell Gorge), Western Australia **A** adult male (sel_1234, WAM T155610) **B** same, palp, dorsal **C** adult male (sel_1237, WAM T155613) **D** same, palp, dorsal **E** palp, retrolateral (sel_1234, WAM T155610) **F** palp, retrolateral (sel_1237, WAM T155613). Scale bars: 0.5 mm.

**Figure 26.**
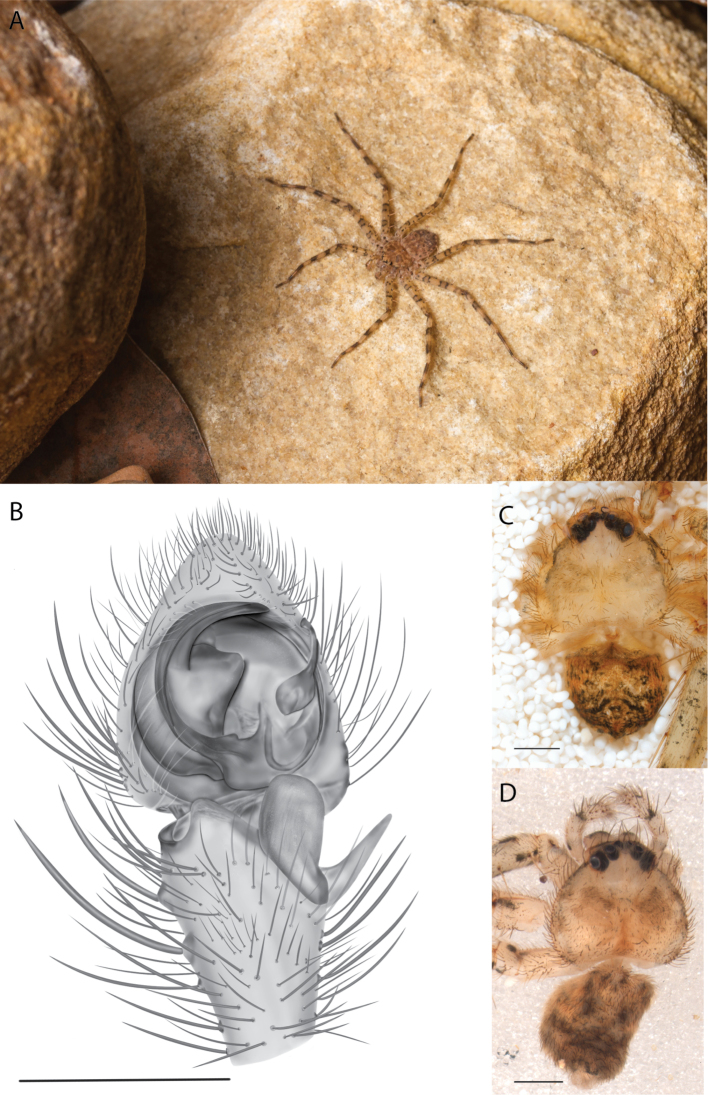
Members of the Kimberley species group **A***Karaopsdalmanyi* sp. nov., penultimate male on rock at night (photo: J. DeJong) **B***Karaopsdalmanyi* sp. nov., palp, ventral (sel_1237, WAM T155613) **C***Karaopsdalmanyi* sp. nov., paratype male (sel_1233, WAM T155609) **D***Karaops* sp., Keep River National Park, Northern Territory (sel_1315, MAGNT A004871). Scale bars: 0.5 mm (**B**); 1 mm (**C, D**).

**Figure 27.**
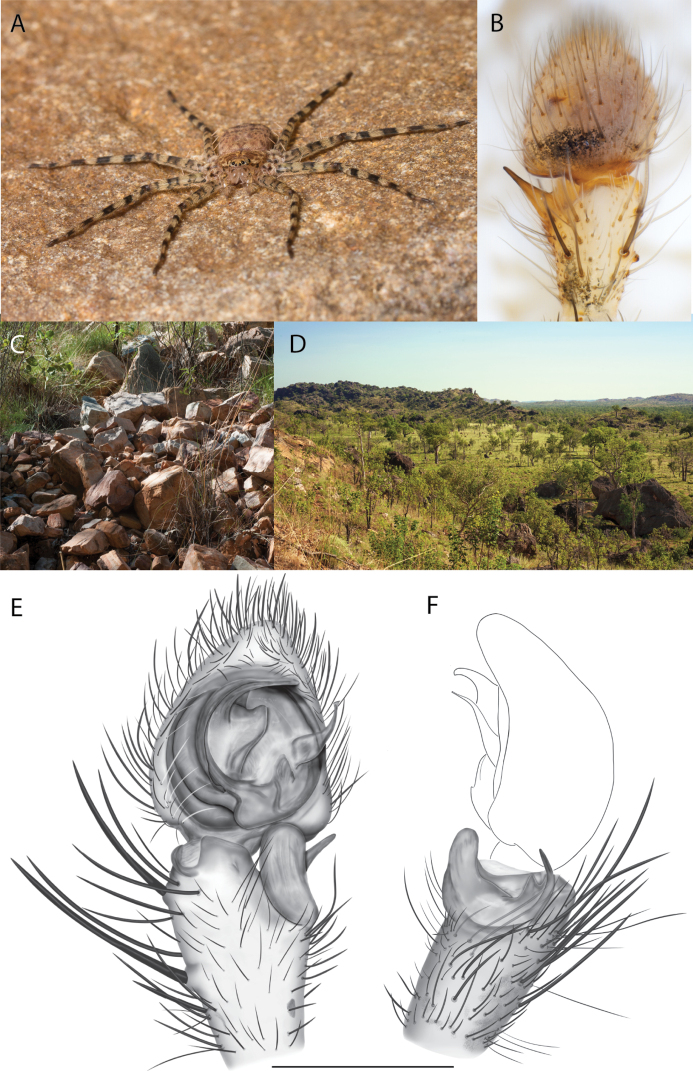
Members of the Kimberley species group **A***Karaopsdalmanyi* sp. nov., Dalmanyi (Bell Gorge), Western Australia **B***Karaopsdalmanyi* sp. nov., paratype male, palp, dorsal (sel_1237, WAM T155613) **C** habitat, Emma Gorge, Western Australia **D***Karaopsjenniferae* habitat, Mowambini (Oscar Range), Western Australia **E***Karaopsdalmanyi* sp. nov., paratype male, palp, ventral (sel_1237, WAM T155613) **F** same, palp, retrolateral. Scale bar: 0.5 mm.

**Figure 28.**
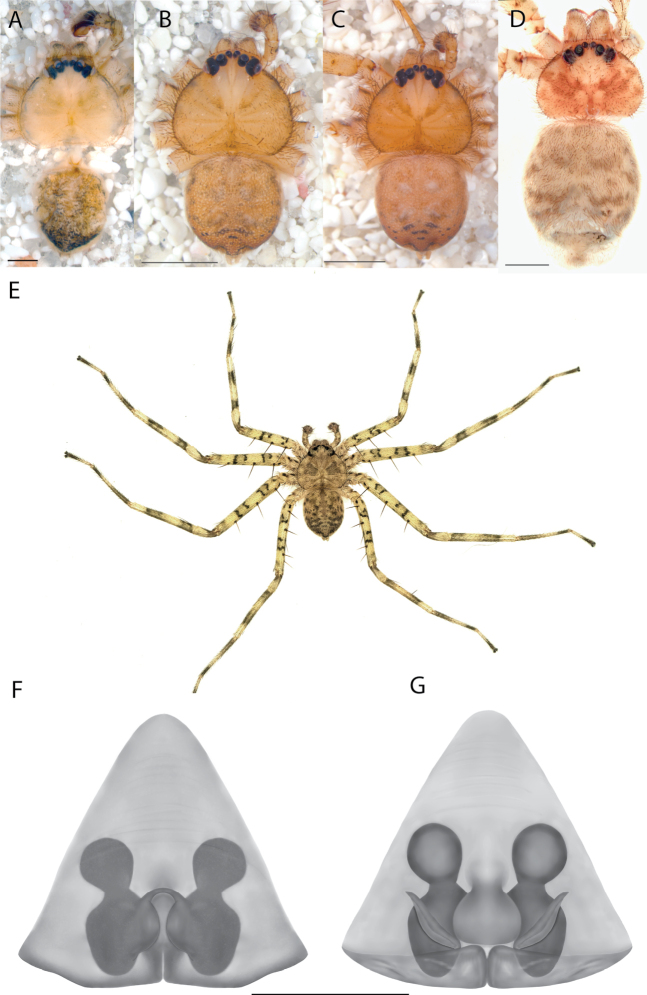
Members of the Kimberley species group **A***Karaopsalanlongbottomi*, holotype male, Degerando Island, Champagny Islands, Western Australia (WAM T28014 ex 93/1330) **B***Karaopsconilurus* sp. nov., holotype male, Conilurus Island, Buccaneer Archipelago, Western Australia (WAM T110400) **C***Karaopsconilurus* sp. nov., paratype male, Conilurus Island, Buccaneer Archipelago, Western Australia (WAM T110401) **D***Karaopsjenniferae*, holotype female, Mowambini (Oscar Range), Western Australia (WAM T65078) **E***Karaopsdalmanyi* sp. nov., paratype male, Dalmanyi (Bell Gorge), Western Australia (sel_1233, WAM T155609) **F***Karaopsjenniferae*, holotype female, epigyne (WAM T65078) **G** same, endogyne. 0.5 mm (**F, G**); 1 mm (**A–D**).

**Male** (paratype) (Fig. 22D). Total length 6.11. Carapace length 2.82, width 3.01. Chelicerae: promargin with three teeth, retromargin with two teeth. Eyes: AER recurved, PER strongly recurved; diameters AME 0.16, ALE 0.07, PME 0.28, PLE 0.37; interdistances AME–PME 0.06, PME–ALE 0.20, ALE–PLE 0.29, PME–PME 0.94, ALE–ALE 1.30, AME–AME 0.48, PLE–PLE 1.67. Abdomen: length 3.29, width 2.68. Color (in life Figs 25A, C, 26A, 27A, 28E/preserved Fig. 22D, E): Carapace: tan with darker brown marks medially on either side of fovea, smaller brown patches laterally/in nature, spider grayer overall (Figs 23C, 24A, B)/yellowish white, darker markings barely visible; setae as in female. Chelicerae: yellowish orange, paturon with a longitudinal curved mark frontally, setae pale, sparse. Maxillae: yellowish white. Labium: dusky, pale distally. Sternum: yellowish white. Abdomen: dorsally golden tan with dark brown spots anteriorly, laterally, slightly dark in cardiac area, two dark patches medially, chevrons posteriorly/orange-red with black spots; ventrally yellowish white. Legs: yellow with black marks, Cx, Tr with dark spot prolaterally, Fm with dots, jagged lines that do not completely encircle legs, Pt with annulation at Fm-Pt joint, Ti with annulations at Pt-Ti joint and ~ midway toward Mt, Mt with dark annulations at Ti-Mt joint and Mt-Ta joint, Ta dark distally; spination leg I Fm d 1-1-1, pl 1-1-0, Ti d 1-1-1, v 2-2-2-2-2, Mt v 2-2-2-2; leg II Fm d 1-1-1, Ti v 2-2-2-2-2, Mt v 2-2-2-2; leg III Fm d 1-1-1, rl 0-0-1, Ti v 2-2-2, Mt 2; leg IV Fm d 1-1-1, pr 1-1-1, v 0-1-1, Ti v 2-2, pr 0-1, rl 1-1, Mtpr 1; leg formula 3241; measurements leg I 12.62 (3.76, 1.40, 3.49, 2.40, 1.57); leg II 16.13 (5.00, 1.82, 4.38, 3.22, 1.71); leg III 16.85 (5.37, 1.67, 4.49, 3.71, 1.61); leg IV 14.52 (4.53, 1.35, 3.87, 3.13, 1.64). Palp: spination Fm d 0-1-2; 2.55 (0.7, 0.4, 0.65, 0.8); dark marks dorsally at Pt-Ti joint, one on Pt, one on Ti, Cy with dusky mark basally; dRTA with two apophyses, one furthest from Cy long, narrow, pointed, slightly curved ventrally, other small, triangular, not visible in ventral view, vRTA in ventral view long, rounded, slightly plicate; palpal Ti noticeably long; rbcp smallish; Cy triangular; C crescent shaped, distally narrowed, directed retrolaterally, with CS, mpc protruded, squared off, curved laterally, C extended ventrally at angle so anterior, distal-most part extends furthest (Figs 25E, F, 27E, F); E hook shaped, arises from medium-sized TL with slightly ribbed part next to MA, begins at approximately 6 o’clock, ends at approximately 12 o'clock, most hidden by CS, between middle of bulb and edge of Cy; MA not very sclerotized, very long, thin, with slightly sclerotized flattened knob distally, projected ventrally.

##### Variation.

(*n* = 3) Rearing produced three adult males. The abdomen of sel_1234 is similar to the paratype, but the cardiac area is dark (Figs 25A, 28E). Total length ranges from 4.40–6.11. The leg formula in sel_1237 is 2341; however, in groups where legs often come off and regenerate, leg formulae and spination vary. There is also some variation in the RTA (Figs 25B, D–F, 26B, 27B, E, F).

##### Etymology.

The species name is the Ungarinyin word for the type locality. Noun in apposition.

##### Distribution.

Known from only the type locality, Dalmanyi (Bell Gorge), in the Kimberley region, Western Australia (Fig. 24C, Map 7).

##### Natural history.

Dalmanyi is located in the Pentecost subregion of the Central Kimberley bioregion. It is a mountainous area with a dry, hot tropical and sub-humid to semi-arid climate with summer rainfall and a total of 750–1000 mm/year. This subregion is largely sandstone. The vegetation is *Triodia* Brown spp. hummock grasses and savannah woodlands of eucalypts (Graham 2001d). Adults seem to be common in the cooler, drier season.

Juveniles and four penultimate males and penultimate females were collected in May, a month when it is becoming drier and cooler. All adults matured in captivity from June–Sep., in the dry cool part of the year, the dry-getting warmer part of the year, and the dry, hot part of the year. None during what would be the wettest time of year. The female and a male lived for three months after maturity, whereas the other males died within a month of reaching maturity. Most specimens of this species were found on rock walls at night. This is one of the few localities where a spider (a single spider) from the Kimberley group was found on a tree rather than rocks (Suppl. material 2: tables S1, S8).

##### Discussion.

In the bioregion and subregion, rainforest patches are areas of endemism for some invertebrate taxa, with dry ones acting as refugia during the dry season. Sandstone can also act as refugia to protect organisms from fire. There have been no systematic faunal surveys in the region.

#### ﻿ Karaops jenniferae

Taxon classificationAnimaliaAraneaeSelenopidae

Crews & Harvey, 2011

9E33B040-C1FB-52F6-9075-52C3B20640E0

Figs 27D 28D, F, G, Maps 1 , 7

Karaops jenniferae Crews & Harvey, 2011: 80, figs 77, 78 (♀, examined).

##### New records.

Western Australia • 2 imm.; Devonian Reef Conservation Park, Oscar Range; 17°38.15.73"S, 125°10'3.31"E; 25 May 2016; S. Crews, J. DeJong leg.; under large limestone rocks; sel_1274–1275; SCC16_056; (WAM T155650–T155651).

##### Diagnosis.

This species can be differentiated from other members of the group by the genitalia. The accessory bulbs are conspicuously anterior to the atrium in this species than they are in any of the others in the Kimberley group (Fig. 28F, G).

##### Description.

The description of the female can be found in Crews and Harvey (2011).

**Male.** Unknown.

##### Distribution.

Known from only the type locality, Oscar Range, Western Australia (Map 7).

##### Natural history.

The Oscar Range (Fig. 27D) is located in the Mount Eliza subregion of the Central Kimberley bioregion. The climate is dry, hot tropical to sub-humid to semi-arid. The area is craggy with savannah woodland and vine thickets (Graham 2001e). The spiders were found under large rocks in an area shaded by a large rock formation. The immature specimens were collected in a cooler month with little rain, and the adult female was collected in a cooler month with very little rain (Suppl. material 2: tables S1, S9).

##### Discussion.

*Karaopsjenniferae* (Fig. 28D) is part of a recent, sizeable radiation in the Kimberley, including the offshore islands. Because of its similarity to other species nearby, new illustrations are provided for ease of comparison (Fig. 28F, G). The male remains unknown. The two immatures were assigned to this species because they were collected in the same locality as the type, and molecular data also recover them with the type (Suppl. material 1).

#### ﻿ Karaops alanlongbottomi

Taxon classificationAnimaliaAraneaeSelenopidae

Crews & Harvey, 2011

AB4919FD-DC88-5BE8-883D-954709FBDB24

**Figure 29.**
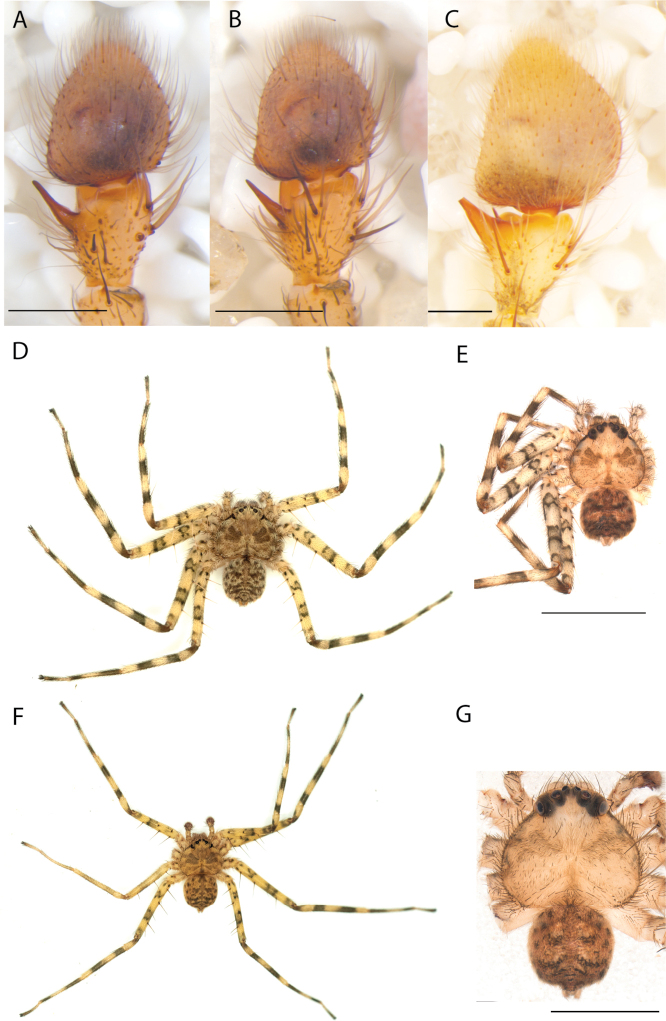
Members of the Kimberley species group **A***Karaopsconilurus* sp. nov., paratype male, palp, dorsal, Conilurus Island, Buccaneer Archipelago, Western Australia (WAM T110401) **B***Karaopsconilurus* sp. nov., holotype male, palp, dorsal, Conilurus Island, Buccaneer Archipelago, Western Australia (WAM T110400) **C***Karaopsalanlongbottomi*, holotype male, palp, dorsal, Degerando Island, Champagny Islands, Western Australia **D***Karaopsmalumbu* sp. nov., holotype female, El Questro Gorge, Western Australia (sel_1305, WAM T155681) **E** same **F***Karaopsmalumbu* sp. nov., paratype male, El Questro Gorge, Western Australia (sel_1309, WAM T155685) **G** same. Scale bars: 0.5 mm (**A–C**); 5 mm (**E, G**).

Figs 28A , 29C , Maps 1 , 7

Karaops alanlongbottomi Crews & Harvey, 2011: 32, figs 17, 18 (♂, examined).

##### Diagnosis.

*Karaopsalanlongbottomi* can be differentiated from other species of the Kimberley group by the conductor and the median apophysis. Distally, the conductor is very narrow and extends retrolaterally beyond the edge of the cymbium (Crews and Harvey 2011: fig. 17). Additionally, the median apophysis is very small and narrow, and the tegular lobe is almost non-existent.

##### Description.

The description of the male can be found in Crews and Harvey (2011).

**Female.** Unknown.

##### Distribution.

Known from only the type locality, Degerando Island, Champagny Islands, Western Australia (Map 7).

##### Natural history.

Nimemba (Degerando Island) is a small island (1337 ha) of the Champagny Islands in the Bonaparte Archipelago. It is in the Mitchell subregion of the Northern Kimberley bioregion. The climate is tropical monsoonal. While there are plants and animals of special interest in the subregion, very little is known of the terrestrial arthropod fauna, and there have been no systematic surveys of flora or fauna in the area. Some of the islands are even free of invasive animal species and uninhabited by humans. Thus, they are likely to harbor unique species. The single adult male was collected in July, in the dry season (Suppl. material 2: table S1).

##### Discussion.

The holotype (Fig. 16D) was collected on the northwest tip of the island, and there are not many rocks or much vegetation. The island is low elevation and will be affected by sea level rise due to climate change. The female remains unknown.

#### ﻿ Karaops conilurus sp. nov.

Taxon classificationAnimaliaAraneaeSelenopidae

CB360B32-5700-5854-A74C-CD6ECE4D6996

https://zoobank.org/9105DA5C-BB3A-4AA3-B8C3-FA4F10E8342C

Figs 28B, C , 29A, B , Maps 1 , 7

Karaops umiida Crews, 2013: 447, figs 3–4 (♂ only, mismatched).

##### Material examined.

***Holotype***: Western Australia • ♂, Buccaneer Archipelago, Conilurus Island, 127 km north of Derby; 16°9'12"S, 123°34'42"E; 18 Jul. 2010; R. Teale, M. Greenham leg.; opportunistic collecting; (WAM T110400). ***Paratype***: ♂; same data as previous; (WAM T110401). **Other material examined**: 1 imm., same data as previous; 135 km north of Derby; 16°5'0"S, 123°32'28"E; 20 Jul. 2010; (WAM T110402).

##### Diagnosis.

In light of the new species described here, a new diagnosis is provided. This species can be distinguished from all other members of the group by the genitalia. *Karaopsconilurus* sp. nov. (Fig. 28B, C) is most similar to *K.garyodwyeri* sp. nov. (Fig. 31B, D, E), *K.dejongi* sp. nov., and *K.malumbu* sp. nov. (Fig. 32A–C) but differs by the conductor and dRTA (Fig. 29A, B). In *K.garyodwyeri* sp. nov., the tip of the conductor curves posteriorly and does not extend past the medial part of the conductor, and the dRTA is broad, with a spine on the tip of the outer branch. In *K.conilurus* sp. nov., the tip of the conductor extends beyond the medial part, and the dRTA is long and narrow in ventral view (Crews 2013: figs 3, 4). In *K.dejongi* sp. nov., the tip of the conductor is similar to *K.conilurus* sp. nov., but the medial part of *K.conilurus* sp. nov. is more upturned, the median apophysis is located more distally, and the dRTA does not have a keel. Additionally, the vRTA of *K.dejongi* sp. nov. is narrow and arched, whereas in *K.conilurus* sp. nov. it is straighter on the retrolateral side and more spoon shaped overall in ventral view. Finally, *K.conilurus* sp. nov. differs from *K.malumbu* sp. nov. by the conductor being more indented laterally below the tip/above the medial part, and the distal part of the conductor is flat rather than arched, with a larger sheath. The dRTA is also connected to a prolateral keel.

##### Description.

The description of the male can be found in Crews (2013: sub *Karaopsumiida*).

##### Etymology.

The name is taken from the type locality, Conilurus Island. Noun in apposition.

##### Distribution.

Known from only the type locality, Conilurus Island, Buccaneer Archipelago, Western Australia (Map 7).

##### Natural history.

The species was collected in the cooler, drier time of the year, under rocks on boulder scree during the day. It is found in the Mitchell subregion of the Northern Kimberley bioregion (Suppl. material 2: table S1).

##### Discussion.

*Karaopsconilurus* sp. nov. was originally described as the male of *K.umiida*. At the time, the rich diversity of the Kimberley species group was unknown, and the general similarity and proximity of the specimens made the decision reasonable. Based on molecular data (Suppl. material 1) and the new knowledge of the large number of species, it is clear that the males here are not conspecific with *K.umiida* or any of the other species in the group and thus represent a new species. Given the diversity of species in close proximity on the mainland, it is expected that Kimberley islands also harbor multiple species that will only be found with targeted surveys.

#### ﻿ Karaops malumbu sp. nov.

Taxon classificationAnimaliaAraneaeSelenopidae

D24D4F6D-2D0E-53B8-8651-480FE52B247A

https://zoobank.org/F1F3D7E3-180A-4AD0-825C-AB1DEC8D3E76

**Figure 30.**
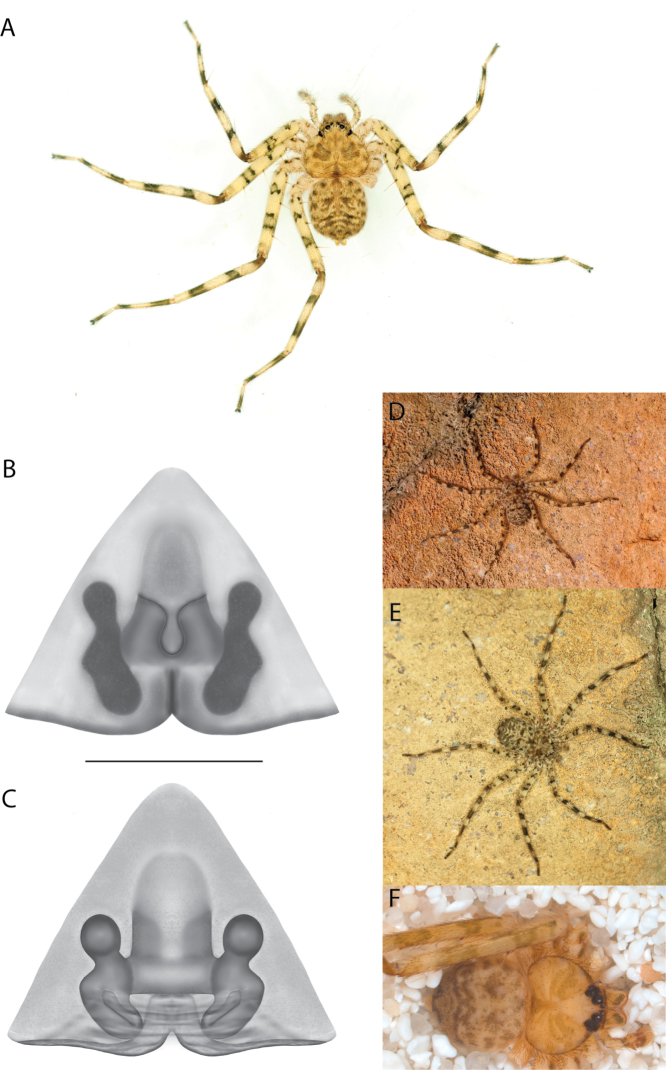
**A***Karaopsmalumbu* sp. nov., El Questro Gorge, Western Australia (sel_1306, WAM T155682) **B** paratype male, El Questro Gorge, Western Australia (sel_1309, WAM T155685) **C** same **D** El Questro Gorge, Western Australia (photo: J. DeJong) **E** El Questro Gorge, Western Australia **F***Karaopslarryoo*, holotype male, Larryoo, Drysdale River National Park, Western Australia. Scale bar: 0.5 mm.

Figs 29D–G , 30A–E , 32A–C , Maps 1 , 7

##### Material examined.

***Holotype***: Western Australia • ♀ (reared in captivity); El Questro Wilderness Area, El Questro Gorge 16°1'16.32"S, 128°1'23.47"E; 30 May 2016; col. S. Crews, J. De Jong leg.; at night on rock walls near overhangs; sel_1305; SCC16_066; (WAM T155681). ***Paratypes***: ♂, ♀ (reared in captivity); same data as previous; sel_1306, 1309; (WAM T155685, T155682). **Other material examined**: 3 imm.; same data as previous; sel_1307, 1308, 1310; (WAM T155683–T155684, T155686).

##### Diagnosis.

The female of *Karaopsmalumbu* sp. nov. (Fig. 30A, E) is similar to other Kimberley group species but can be separated from all but *K.umiida* by the lateral lobe suture which does not reach the atrium in the median field. It can be separated from *K.umiida* by the sides of the lateral lobes which form a pendant droplet shape (Fig. 30B), whereas those of *K.umiida* form a u-shape. The copulatory openings in *K.malumbu* sp. nov. are inside of a large atrium with a horizontal posterior edge, but the copulatory openings in *K.umiida* are in a small atrium, with the copulatory ducts branching posterolaterally.

The male of *Karaopsmalumbu* sp. nov. (Figs 29F, 30D) is most similar to *K.dejongi* sp. nov., but it can be differentiated by the median apophysis, which has spinules on the base (Fig. 32A), not found in *K.dejongi* sp. nov. Additionally, in lateral view, the dRTA of *K.malumbu* sp. nov. has an outer, longer branch, and an inner, shorter, more conical branch, whereas in *K.dejongi* sp. nov., there is no shorter branch but a jagged structure that connects the dRTA to the vRTA.

**Figure 31.**
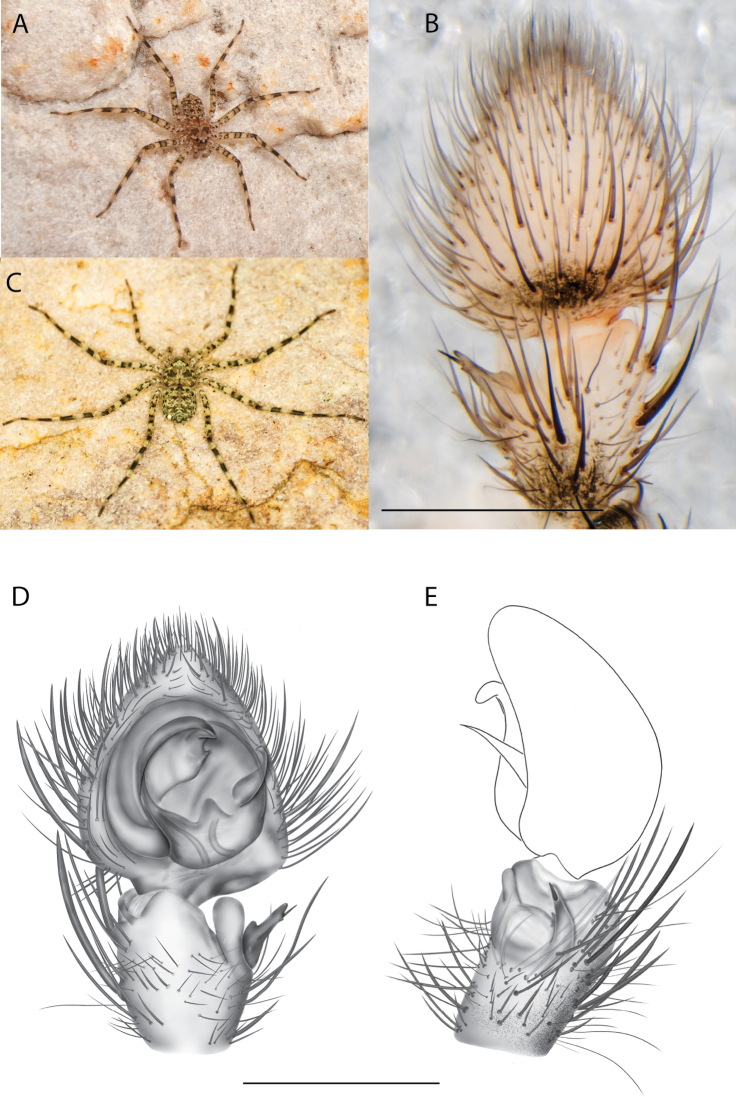
*Karaopsgaryodwyeri* sp. nov., 167 km west of Wyndham, Gibb River Road, Western Australia **A** juvenile on sandstone (photo J. DeJong) **B** palp, dorsal **C** juvenile on sandstone **D** palp, ventral (sel_1256, WAM T155632) **E** palp retrolateral. Scale bars: 0.5 mm.

**Figure 32.**
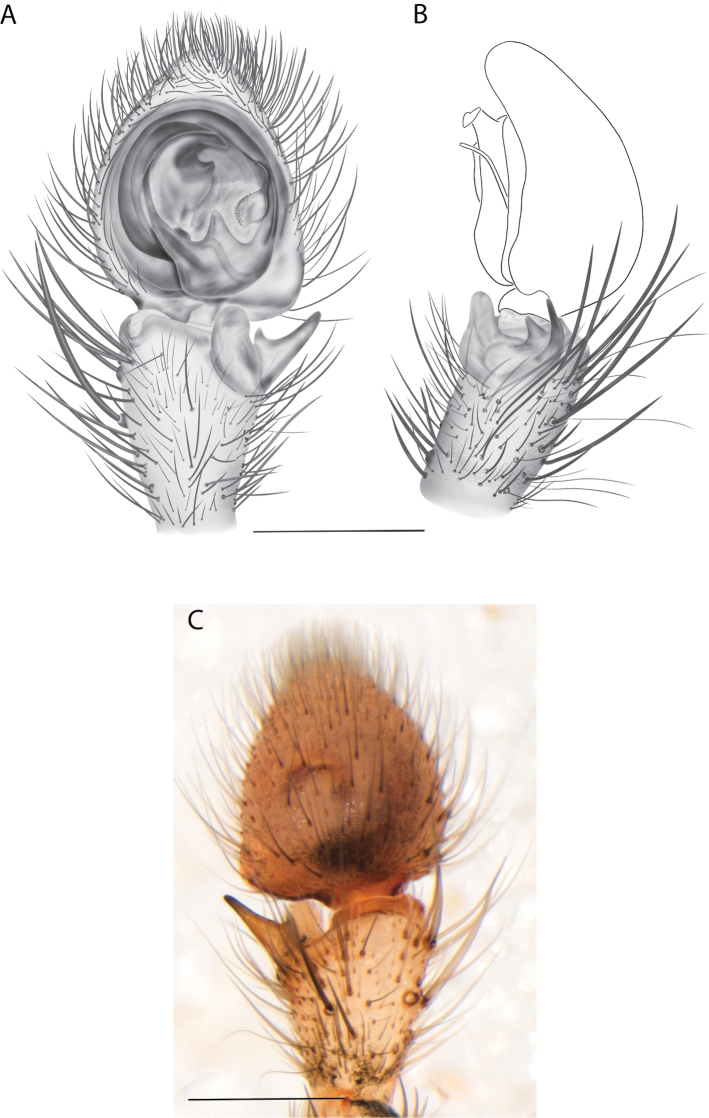
*Karaopsmalumbu* sp. nov., El Questro Gorge, Western Australia **A** palp, ventral (sel_1309, WAM T155685) **B** palp, retrolateral **C** palp, dorsal. Scale bars: 0.5 mm.

##### Description.

**Female** (holotype) (Fig. 30A, E). Total length 4.20. Carapace: length 2.21, width 2.60. Chelicerae: promargin with three teeth, retromargin with two teeth. Eyes: AER slightly recurved, PER recurved; diameters AME 0.14, ALE 0.07, PME 0.19, PLE 0.26; interdistances AME–PME 0.06, PME-PLE 0.13, ALE–PLE 0.24, PME–PME 0.79, ALE–ALE 1.11, AME–AME 0.39, PLE–PLE 1.41. Sternum: length 1.59, width 1.64. Abdomen: length 1.99, width 1.79. Color (in life Fig. 29D, 30E/preserved Fig. 29E): Carapace: brownish yellow, with two dark marks below PLE, two large dark patches behind eyes extended to center of carapace, dark mark extended from center just behind furrow to posterior edge of carapace, three pairs of dark spots laterally/orangish white, dark patches less visible except under PLEs. Chelicerae: yellow-brown, longitudinal curved mark frontally, setae pale and thin laterally, darker and thicker anteromedially. Maxillae: yellowish brown. Labium: yellowish tan, pale distally. Sternum: yellowish brown. Abdomen: dorsally, brownish yellow, black dots anteriorly, laterally, two dark patches anteriorly, some chevrons posteriorly/grayish orange; ventrally yellow-gray. Legs: pale brown-yellow, Cx I–III with dark marks dorsally and ventrally (prolaterally and retrolaterally), Tr with dark mark ventrally, Fm with dark spots basally, marks nearly forming annulations medially, dark on edges, not centrally, dusky ring distally at joint, Pt with dark annulation at Fm-Pt joint, Ti with two annulations, one at Pt-Ti joint, other closer to Mt joint, Mt with two annulations, one at Ti-Mt joint, one at Mt-Ta joint, Ta dusky distally; setae on dorsal Fm orangish, dark at tip; spination leg I Fm d 1-1-1, pr 1-1-0, Ti v 2-2-2-2-2, Mt v 2-2-2; leg II Fm d 1-1-1, Ti v 2-2-2-2-2-2, Mt v 2-2-2-2; leg III Fm d 1-1-1; leg IV Fm d 1-1-1; leg formula 3241; measurements leg I 9.01 (2.62, 1.12, 2.54, 1.78, 0.95); leg II 10.29 (3.27, 0.99, 2.96, 2.15, 0.92); leg III 11.08 (3.69, 1.11, 2.88, 2.26, 1.14); leg IV 9.60 (3.18, 0.92, 2.32, 2.15, 1.03). Palp: spination Fm 1-1-2, 2.96 (1.11, 0.39, 0.57, 0.89), claw with seven teeth. Epigyne: EP triangular; MF with pendant droplet-shaped At; LLs suture posteriorly, does not reach posterior edge of At; COs in At. Endogyne: large At; CDs large, wide, short, leading to S, ABs, both more sclerotized than CDs, ABs nearly even with top of At, round; S allantoid; FDs directed anterolaterally; small pdf.

**Male** (paratype). Total length 3.79. Carapace: length 2.20, width 2.55. Chelicerae: promargin with three teeth, retromargin with two teeth. Eyes: AER slightly recurved, PER recurved; diameters AME 0.20, ALE 0.08, PME 0.21, PLE 0.27; interdistances AME–PME 0.03, PME–ALE 0.15, ALE–PLE 0.22, PME–PME 0.72, ALE–ALE 1.02, AME–AME 0.36, PLE–PLE 1.39. Sternum: length 1.42, width 1.61. Abdomen: length 1.59, width 1.58. Color (in life Figs 29F, 30D/preserved Fig. 29G): Carapace: brownish yellow with dark patches behind eyes, medially to furrow, three pair of dark spots laterally, dark area posterior to furrow to posterior edge of carapace/orangish white. Chelicerae: tan, with dark patches anteromedially, paturon with longitudinal curved mark frontally/faded. Maxillae: yellowish white. Labium: brownish yellow, pale distally. Sternum: yellowish white. Abdomen: dorsally yellowish brown with dark mark anteriorly, dark spots anteriorly, laterally, two dark patches in anterior 1/3, some chevrons posteriorly/reddish orange, dark patches visible; ventrally yellow-gray. Legs: yellowish white, Cx I–III with dark marks dorsally, ventrally, Tr with dark mark ventrally, Fm with dark spots basally, with annulations medially, dark on edges, not centrally, dusky ring distally at joint, Pt with dark annulation at Fm-Pt joint, Ti with two annulations, one at Pt-Ti joint, one closer to the Mt joint, Mt with two annulations, one at Ti-Mt joint, one at Mt-Ta joint, Ta dusky distally; spination leg I Fm d 1-1-1, pr 1-1-0, Ti v 2-2-2-2-2, Mt v 2-2-2; leg II Fm d 1-1-1, Ti v 2-2-2-2-2, Mt v 2-2-2; leg III Fm d 1-1-1, pr 0-0-1, Ti v 2-2; leg IV Fm d 1-1-1; leg formula 3241; measurements leg I 10.91 (3.23, 1.18, 3.06, 2.30, 1.14); leg II 12.97 (4.04, 1.19, 3.62, 2.86, 1.26); leg III 14.31 (4.57, 1.35, 3.83, 3.19, 1.37); leg IV 12.48 (3.86, 1.04, 3.28, 3.00, 1.30). Palp: spination Fm d 0-1-2; 2.93 (0.93, 0.55, 0.62, 0.83); vRTA spoon shaped to oblong, ventrally, dRTA with two points visible in ventral or lateral view (Fig. 32A–C); rbcp smallish; Cy triangular; C with CS that covers ~ 1/2 of E, CS not as sclerotized as C tip; E emerging from large TL with anteriorly projecting middle part, begins at 6 o’clock, ends at 12 o’clock; MA broad at base, with Sp, abruptly narrowed, widened, more sclerotized at tip.

##### Etymology.

The species name comes from the indigenous name for the area, Malumbu, in the Ngarinyin language. The Ngarinyin are one of three nations of the Wanjina Cultural Group whose Country is in the North West of the Kimberley Region of Western Australia.

##### Distribution.

Known from only the type locality, El Questro Gorge, Western Australia.

##### Natural history.

The type locality is in the Victoria Bonaparte I subregion of the Victoria Bonaparte bioregion. The dominant plant community is eucalypt woodlands (Graham 2001f). The climate is subtropical and most rainfall occurs from December to March. Penultimate females and males were around in the cooler, drier part of the year. Adult males were present at the end of the cooler, drier season and females during the dry, hot (Suppl. material 2: tables S1, S10). This species was collected on rock walls of a gorge at night.

##### Discussion.

According to IBRA, centers of endemism include rainforest patches, and “dry” rainforest patches may act as refugia. There has been specific survey work in the region but no systematic review of biodiversity. Graham (2001f) stated that there is reasonable evidence of loss of species and assemblage changes. One of the ecosystems at risk is the invertebrate community that occurs at springs found in the area of the type locality.

#### ﻿ Karaops larryoo

Taxon classificationAnimaliaAraneaeSelenopidae

Crews & Harvey, 2011

AA27C9B8-BF85-5552-ACD3-8C67B0314857

Fig. 30F , Maps 1 , 7

Karaops larryoo Crews & Harvey, 2011: 35, figs 21, 22 (♂, examined).

##### Diagnosis.

In light of the new species described here, a new diagnosis is provided for *Karaopslarryoo* (Fig. 30F). Although this species is similar to other members of the group, it can be distinguished by the conductor, median apophysis, and RTA. The conductor is nearly semi-circular, with only a very small indentation between the anterior tip and the medial part. The median apophysis is short and wide at the base. The dRTA is longer than the vRTA in ventral view, and the vRTA is narrow (Crews and Harvey 2011: figs 20, 21).

##### Description.

The description of the male can be found in Crews and Harvey (2011).

**Female.** Unknown.

##### Distribution.

Known only from the type locality, Larryoo, Drysdale River National Park, Western Australia (Map 7).

##### Natural history.

The specimen was collected in the cooler, drier time of the year, under rocks. Larryoo is located in the Northern Kimberley bioregion in the Berkeley subregion; however, it is very close to the Mitchell subregion. The subregion is classified by medium summer rainfall with savannah woodland and high sorghum grasses and is less rugged than the Mitchell subregion (Suppl. material 2: table S1).

##### Discussion.

This area is difficult to reach, which is both good and bad. It’s inaccessibility to humans has left it relatively free of grazing and introduced, invasive species, but very little is known about the subregion’s flora and fauna. The female remains unknown.

#### ﻿ Karaops garyodwyeri sp. nov.

Taxon classificationAnimaliaAraneaeSelenopidae

709C35C7-2657-5D08-B547-2BB6EA3521FB

https://zoobank.org/DB8EAB79-08E2-4A41-8DB1-B7DBB231CC45

Fig. 31A–E , Maps 1 , 7

##### Material examined.

***Holotype***: Western Australia • ♂ (reared in captivity); Gibb River Road, ~ 167 km west of Wyndham, on north side of road; 15°49'27.64"S, 127°31'34.03"E; ~ 269 m; 22 May 2016; S. Crews, J. DeJong leg.; on boulders at night; sel_1256; (WAM T155632). **Other material examined**: 4 imm.; same data as previous; sel_1254–1255, 1257–1258; (WAM T155630–T155631, T155633–T155634).

##### Diagnosis.

This species is most similar to *Karaopsconilurus* sp. nov., *K.dejongi* sp. nov., and *K.malumbu* sp. nov. but differs by the conductor, median apophysis, and RTA. In *K.conilurus* sp. nov., the anterior part of the conductor extends beyond the medial part, and the dRTA is a very long and slender single branch. In *K.garyodwyeri* sp. nov. (Fig. 31A, C), the anterior part of the conductor barely extends beyond the medial part of the conductor (Fig. 31D), and the dRTA is shorter and wider, with two branches. In *K.dejongi* sp. nov., the medial part of the conductor is straight, giving it a mostly quadrangular appearance, the dRTA extends into a keel prolaterally, and the vRTA is arched on the retrolateral side. In *K.malumbu* sp. nov., the conductor is flatter along the anterior margin, the base of the median apophysis has several short spinules, and the dRTA widens distally.

##### Description.

**Male** (holotype). Total length 5.75. Carapace: length 2.80, width 3.33. Chelicerae: promargin with three teeth, retromargin with two teeth (1-0-1). Eyes: AER recurved, PER recurved; diameters AME 0.13, ALE 0.08, PME 0.15, PLE 0.30; interdistances AME–PME 0.12, PME-PLE 0.16, ALE–PLE 0.32, PME–PME 0.94, ALE–ALE 1.42, AME–AME 0.48, PLE–PLE 1.73. Sternum: length 1.78, width 1.82. Abdomen: length 2.95, width 2.97. Color: Carapace: yellowish brown with two large dark patches medially on either side of fovea, three pairs of dark spots on lateral margins, one small dark patch medioposteriorly at margin, short, thick, sparse but evenly distributed setae, pale, long, slender setae, denser than short setae, fairly sparse. Chelicerae: yellowish brown, paturon with a longitudinal curved mark frontally, pale setae laterally, darker toward anterior. Maxillae: whitish. Labium: yellowish tan, pale distally. Sternum: whitish yellow. Abdomen: dorsally reddish brown with dark spots around anterior margin, two darker patches anteromedially, dark chevrons from middle to posterior; ventrally grayish tan. Spinnerets: yellowish tan without dusky marks. Legs: yellowish, Cx with dark mark prolaterally, Tr with dark spots prolaterally at Tr-Fm joint, Fm with dark, rectangular markings with dusky centers basally and medially, annulation at Fm-Pt joint, Pt with annulation basally, Ti with annulation at Pt-Ti joint, another distally, Mt with annulations basally, distally, Ta dusky; spination leg I Fm d 1-1-1, pr 1-1-0, Ti v unpaired pl 1-1-1-1, rl 1-1-1-1-1, Mt v 2-2-2; leg II missing; leg III Fm d 1-1-1, Ti 1-1; leg IV Fm d 1-1-1, pl 0-0-1; measurements leg I 13.23 (4.10, 1.52, 3.46, 2.93, 1.22); leg II missing; leg III 15.78 (5.07, 1.20, 4.15, 3.76, 1.61); leg IV 16.24 (5.21, 1.07, 4.39, 3.90, 1.66). Palp: spination Fm 0-1-2; 2.07 (0.63, 0.34, 0.42, 0.68); dark marks dorsally on Ti at Pt-Ti joint, dusky mark basally on Cy (Fig. 31B); dRTA widened distally, slightly bifurcate, rl branch with spine at tip (see Discussion) (Fig. 31B, D, E), vRTA narrowed, spoon shaped in ventral view; Cy triangular; C somewhat crescent shaped, pointed at tip, curved laterally, mpc basal margin diagonal, tip of C does not extend beyond mpc, slightly hooked, with CS around E (Fig. 31D); E hook shaped, arises from medium-sized TL, wide basally, narrowed distally, originating at ~ 6 o’clock, ending at ~ 12 o‘clock, most beneath CS, E closer to middle of bulb than following edge of Cy; MA very long, thin, with slightly sclerotized, flattened knob distally, projected ventrally.

**Female.** Unknown.

##### Etymology.

This species is named in memory of Gary O’Dwyer. It is a noun in the genitive case.

##### Distribution.

Known from only the type locality, Gibb River Road west of Wyndham, Central Kimberley, Western Australia (Map 7).

##### Natural history.

This species is found in the Pentecost subregion of the Central Kimberley bioregion. The area is mostly savannah woodland of eucalypts over hummock grasses. The climate is sub-humid to semi-arid tropical with lots of rainfall in the summer (Graham 2001d). A penultimate male was found in a cooler, drier part of the year and became an adult male during the coolest, driest part of the year. A specimen molted to a penultimate female in the coolest, driest part of the year. The samples were all large immatures when collected. They were found on boulders at night (Fig. 31A, C). One specimen lived for a year after collection and never molted (Suppl. material 2: tables S1, S11).

##### Discussion.

The palps were not completely sclerotized after molting. It is unclear if the spine on the dRTA is anomalous because there is only a single adult male sample, and the dRTA on the right palp is deformed. There have been no systematic fauna or flora surveys in the subregion.

### *Karaops* species not placed in a species group

#### ﻿ Karaops yumbubaarnji sp. nov.

Taxon classificationAnimaliaAraneaeSelenopidae

75B13A04-9E53-5DE8-B3EE-6433A419E811

https://zoobank.org/478EA254-B064-4D1B-A5DA-C358AF239208

**Figure 33.**
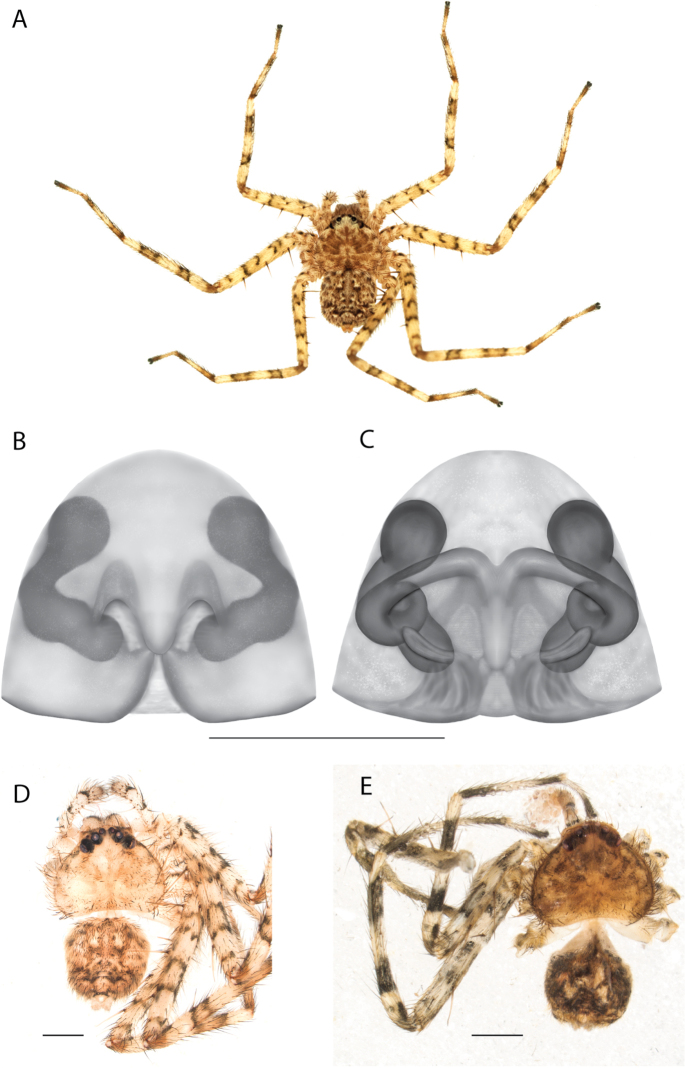
*Karaopsyumbubaarnji* sp. nov. and *Karaops* sp. **A***Karaopsyumbubaarnji* sp. nov., holotype female, Cathedral Gorge Lookout, Purnululu National Park, Western Australia (sel_1302, WAM T155677) **B** same, epigyne **C** same, endogyne **D** same **E***Karaops* sp., Ruby Plains, Western Australia (sel_1279, WAM T155655). Scale bars: 0.5 mm (**B, C**); 1 mm (**D, E**).

**Figure 34.**
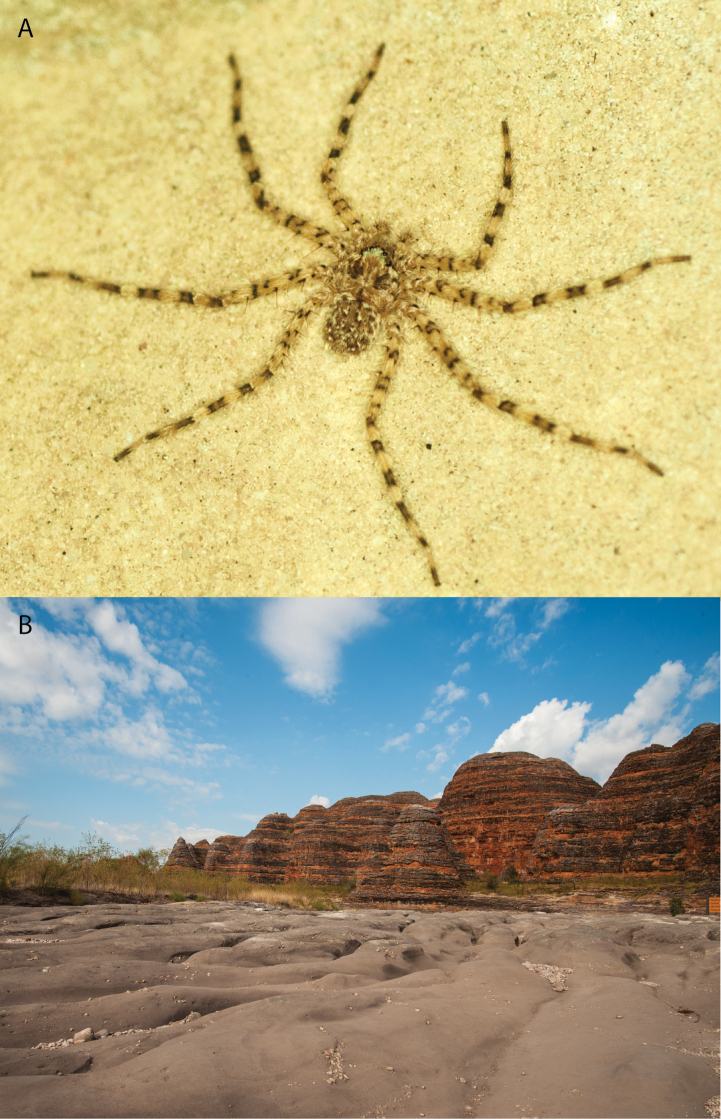
*Karaopsyumbubaarnji* sp. nov., Cathedral Gorge Lookout, Purnululu National Park, Western Australia **A** live spider on sandstone **B** habitat, Bungle Bungles.

**Figure 35.**
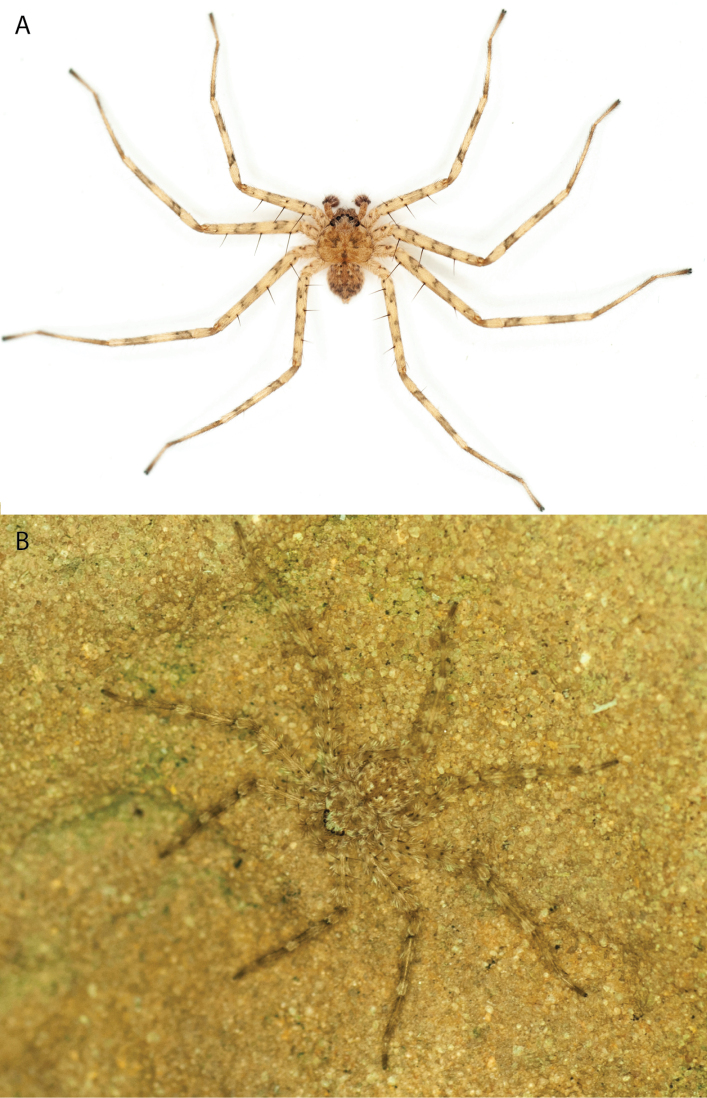
*Karaopsyumbubaarnji* sp. nov., Cathedral Gorge Lookout, Purnululu National Park, Western Australia **A** holotype male (sel_1300, WAM T155676) **B** adult female.

**Figure 36.**
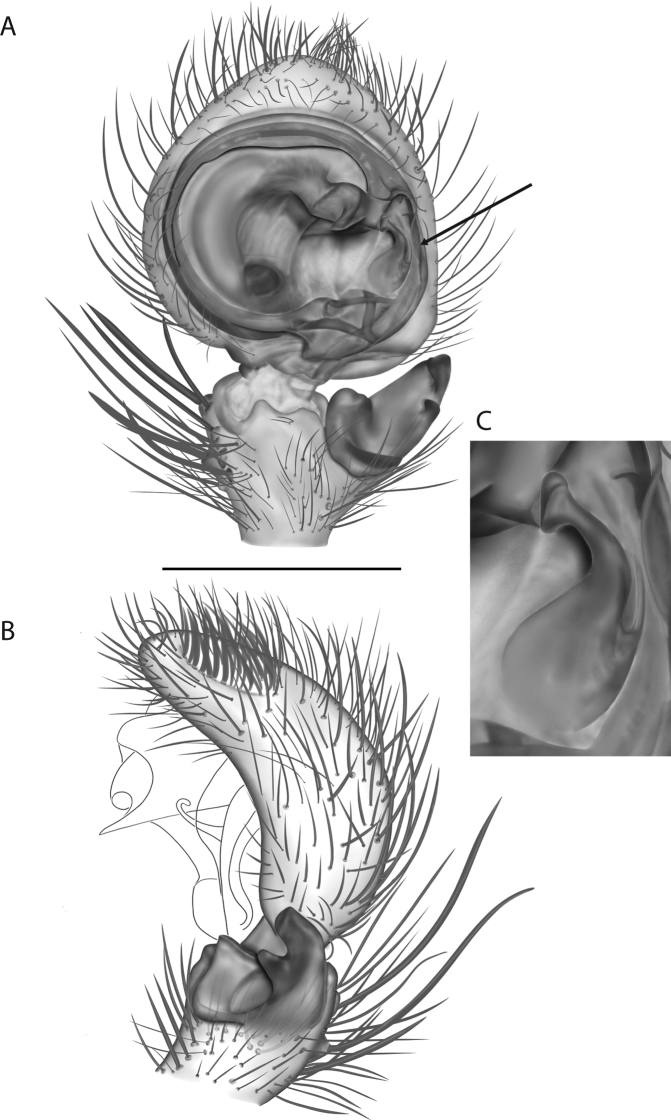
*Karaopsyumbubaarnji* sp. nov., holotype male, Cathedral Gorge Lookout, Purnululu National Park, Western Australia (sel_1300, WAM T155676) **A** palp, ventral, arrow indicates expanded part below **B** palp, retrolateral **C** close up of the median apophysis showing small, spine-like process. Scale bar: 0.5 mm.

**Map 8.**
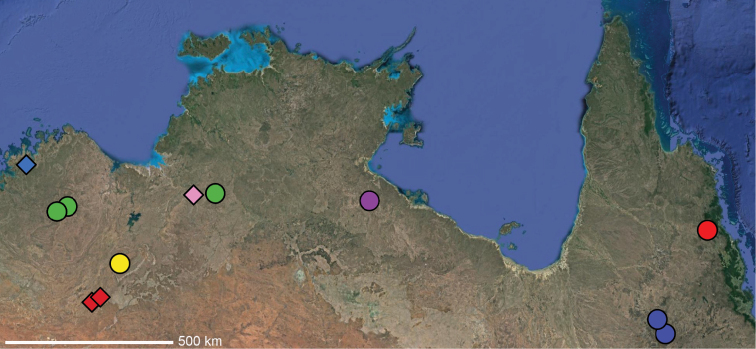
Species that are not currently placed in a species group. Diamonds (juveniles): blue = Little Mertens, red = Ruby Plains, pink = Nackeroo Lookout; Circles: yellow = *Karaopsyumbubaarnji* sp. nov., green = *Karaopsmarkharveyi* sp. nov., purple = *Karaopskennerleyorum* sp. nov., blue = *Karaopsmadhawundu* sp. nov., red = *Karaopsmareeba* sp. nov. Juveniles are treated as distinct species based on molecular data.

**Figure 37.**
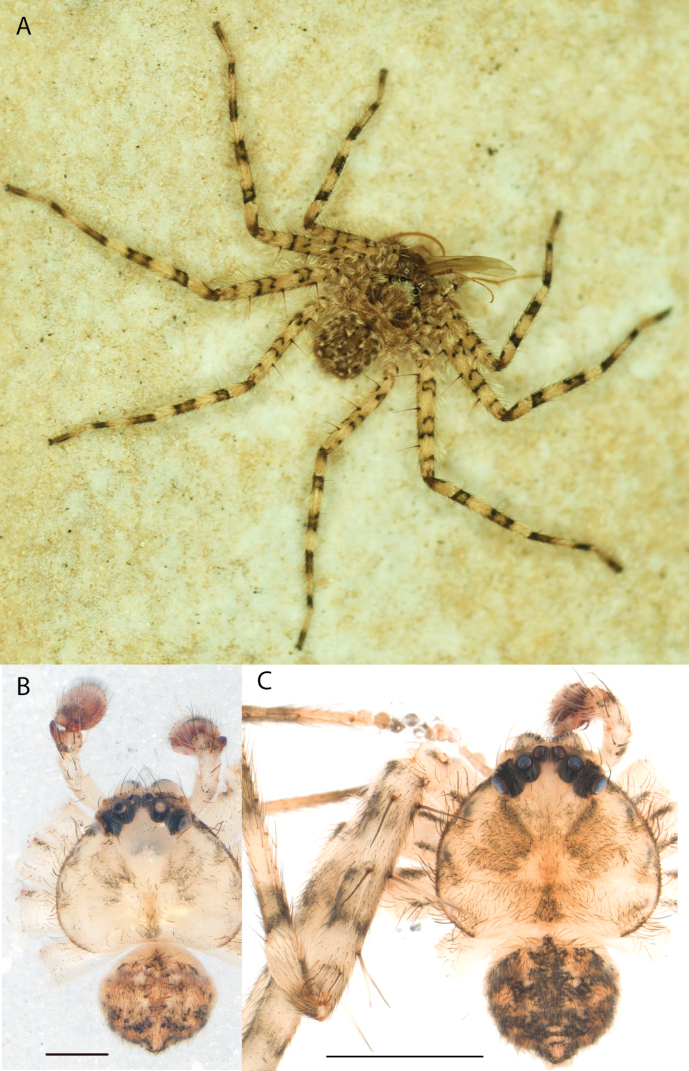
*Karaopsyumbubaarnji* sp. nov. and *Karaopskennerleyorum* sp. nov. **A***Karaopsyumbubaarnji* sp. nov., with hymenopteran prey, Cathedral Gorge Lookout, Purnululu National Park, Western Australia **B***Karaopsyumbubaarnji*, paratype male (sel_1300, WAM T155676) **C***Karaopskennerleyorum* sp. nov., holotype male, Southern Lost City, Limmen National Park, Northern Territory (sel_1343, MAGNT A004899). Scale bars: 1 mm (**B**); 2 mm (**C**).

Figs 33A–E , 34A, B , 35A, B , 36A, B , 37A, C , Maps 1 , 8

##### Material examined.

***Holotype***: Western Australia • ♀ (reared in captivity); Purnululu National Park, Bungle Bungles, Cathedral Gorge, Picaninny Lookout; vic. 17°29'20.67"S, 128°22'29.27"E; 29 May 2016; S. Crews, J. DeJong leg.; at night on rock walls near overhangs; sel_1302, SCC16_065; (WAM T155678). ***Paratype***: ♂ (reared in captivity); same data as previous; sel_1300; (WAM T155676). **Other material examined**: 7 imm., same data as holotype; sel_1296–1299, 1301, 1303–1304; (WAM T155672–T155675, T155677, T155679–T155680).

##### Diagnosis.

Females of *Karaopsyumbubaarnji* sp. nov. (Figs 33A, 35B, 37A) are superficially similar to those of *K.burbidgei* and *K.nyamal* as they all have m-shaped hoods on the median field. *Karaopsyumbubaarnji* sp. nov. can be distinguished by having long, narrow copulatory ducts that are less sclerotized at their origin and extend lateroposteriorly (Fig. 33C). *Karaopsburbidgei* has very short copulatory ducts, and the copulatory ducts of *K.nyamal* extend anteriorly then sharply curve posteriorly (Crews 2013: figs 32, 34).

The males of *Karaopsyumbubaarnji* sp. nov. (Figs 34A, 35A) are not really similar to any other known species. They do have a chemosensory patch on the cymbial tip (Fig. 36A, B) as in *K.markharveyi* sp. nov.; however, the patch is small and restricted to the retrolateral side of the cymbium apically. The conductor of *K.yumbubaarnji* sp. nov. has an apophysis that is projected ventrally like several *Karaops* species in the Pilbara, but it is connected to the larger tegular sheath (Fig. 36A). Unique features include a bifid dRTA (in lateral view) and the median apophysis has a small, spine-like apophysis that is difficult to see (Fig. 36C).

##### Description.

**Female** (holotype). Total length 5.22. Carapace: length 2.55, width 3.37. Chelicerae: promargin with three teeth, retromargin with two teeth. Eyes: AER recurved, PER strongly recurved; diameters AME 0.14, ALE 0.08, PME 0.23, PLE 0.29; interdistances AME–PME 0.04, PME–ALE 0.10, ALE–PLE 0.28, PME–PME 0.80, ALE–ALE 1.11, AME–AME 0.39, PLE–PLE 1.38. Sternum: length 1.43, width 1.70. Abdomen: length 2.67, width 2.04. Color (in life Figs 33A, 35B, 37A/preserved Fig. 33D): Carapace: yellowish with dark marks medially just behind eyes, two dark patches laterally extending to middle of carapace, dark spot behind furrow, white setae behind eyes, tufts laterally that extend along radial furrows, dispersed, short, dark, thick setae/whitish yellow, dark spots, pale setal tufts not as conspicuous. Chelicerae: yellow-brown with small dark mark just below clypeus, paturon with longitudinal curved mark frontally, cheliceral setae pale, darkened anteromedially. Maxillae: yellowish white. Labium: tan, pale distally. Sternum: yellow-brown. Abdomen: dorsally brownish with dark and pale spots and patches, most prominent are two anterior white spots and a darker posterior chevron, hirsute, with longer, thick, dark setae and longer dark, thin setae interspersed/more yellowish orange, dark and pale spots and patches less conspicuous; ventrally yellowish white. Legs: yellowish with unfilled annulations, Cx, Tr all with an anterior dot, slightly dusky jagged annulations on Fm, Pt dark at Fm-Pt joint, two jagged dusky annulations on Ti, Mt with dark annulations on Ti-Mt joint and Mt-Ta joint, Ta tip dusky; Fm ventrally with flat, enlarged, white setae, spines on all legs dark at base, lightened to orange distally; spination leg I Fm d 1-1-1, pr 1-1-0, Ti v 2-2-2-2-2-2, there is a small 7^th^ spine toward pl side, and 6^th^rl is also small, Mt v 2-2-2-2; leg II Fm d 1-1-1, pr 0-0-1, Ti v 2-2-2-2-2-2, Mt v 2-2-2-2-2, single, very small spine toward pl side; leg III Fm d 1-1-1, pr 0-0-1, Ti v 1-1-1; leg IV Fm d 1-1-1; leg formula 3241; measurements leg I 9.56 (3.03, 0.98, 2.59, 1.96, 1.00); leg II 10.81 (3.45, 1.15, 2.98, 2.14, 1.09); leg III 11.03 (3.55, 1.16, 3.04, 2.04, 1.24); leg IV 9.66 (3.19, 0.97, 2.61, 1.90, 0.99). Palp: spination Fm d 0-1-2; 2.41 (0.89, 0.36, 0.45, 0.71); claw with eight teeth. Epigyne: EP rounded anteriorly, with straight posterior margin, not much longer than wide, MF with hoods forming an m-shape; LLs separated posteriorly; COs beneath hoods, anteriorly (Fig. 33B). Endogyne: CDs originate medially, extend lateroposteriorly, coiling anteriorly, less sclerotized than AB and S; oval ABs reaching lateral edges of EP; S more oblong; FDs extended anterolaterally; very small pdf that grades into UE (Fig. 33C).

**Male** (paratype). Total length 3.77. Carapace: length 2.12, width 2.64. Chelicerae: promargin with three teeth, retromargin with two teeth. Eyes: AER recurved, PER strongly recurved; diameters AME 0.10, ALE 0.08, PME 0.14, PLE 0.22; interdistances AME–PME 0.04, PME–ALE 0.10, ALE–PLE 0.21, PME–PME 0.57, ALE–ALE 0.80, AME–AME 0.28, PLE–PLE 0.97. Sternum: length 1.16, width 1.48. Abdomen: length 1.65, width 1.19. Color (in life Fig. 35A/preserved Fig. 37C): Carapace: yellowish brown, two dark patches behind PLEs extended posteriorly nearly midway, dark patch extended from posterior part of furrow to posterior of carapace, two pairs of lateral dark patches/yellowish white, paler, less conspicuous markings, pale setae just behind and lateral to eyes, slender, short, stiff setae. Chelicerae: yellowish brown, paturon with a longitudinal curved mark frontally, setae pale, darkened anteromedially. Maxillae: yellowish white. Labium: dusky, pale distally. Sternum: yellowish white. Abdomen: dorsally yellowish brown, two pairs of white dots anteromedially and medially, dark patches from center to posterior/orangish with markings less conspicuous; ventrally yellowish brown. Legs: yellowish tan with darker markings, Cx, Tr with dark spot prolaterally, Fm with dark dot near Fm-Tr joint, jagged dusky annulations, pale in centers, Pt with dusky annulation at Fm-Pt joint, Ti with two jagged annulations, centers slightly darker than those on Fm, Mt same as previous but space between annulations pigmented, Ta dusky/pale, yellowish white; Fm with white setal tufts enlarged distally along ventral surface of legs; spination leg I Fm d 1-1-1, pr 1-1-0, Ti v 2-2-2-2-2-2-2, Mt v 2-1-2; leg II Fm d 1-1-1, pr 0-0-1, Ti v 2-2-2-2-2-2, Mt v 2-2-2-2; leg III Fm d 1-1-1, rl 0-0-1, Ti v 2-2, Mt v 2-0; leg IV Fm d 1-1-1, rl 0-1-1, Ti v 1-1; leg formula 3241; measurements leg I 8.26 (2.40, 0.79, 2.27, 1.80, 1.00); leg II 10.21 (3.10, 0.92, 2.79, 2.30, 1.10); leg III 10.91 (3.51, 0.93, 3.01, 2.40, 1.06); leg IV 9.53 (3.06, 0.80, 2.43, 2.23, 1.01). Palp: spination Fm d 0-1-2; 2.20 (0.77, Pt 0.37, Ti 0.31, Ta 0.75); RTA darker than Ti, vRTA smaller than dRTA, squared off distally, dRTA bifid, with more basal branch rounded, apical one squared off; rbcp small; Cy roundish, patch of sensory setae conspicuous in retrolateral view; C located ~ 9 o’clock, CS completely covering ~ 1/2 of E, at 2 o’clock position, TS connects to the CS and the distal end of C that arises anteromedially from the T (Fig. 36A), projected ventrally (Fig. 36B); E long, beginning at ~ 6 o’clock, ending ~ 2 o’clock, arising from a small TL; MA uniformly sclerotized, base wide, narrowed distally, slightly widened at tip, truncate, narrow spine-like process retrolaterally (Fig. 36C).

##### Etymology.

The species name is a combination of the Kija/Gija word for spider and the Jaru word for spider, *baarnji* (Gija) (Purdie et al. 2018) and *yumbu* (Jaru) (Deegan et al. 2010). Noun in apposition.

##### Distribution.

Known only from the type locality Purnululu National Park, northeastern Western Australia (Fig. 20F, Map 8).

##### Natural history.

This species occurs in the Purnululu subregion of the Ord Victoria Plains bioregion. A major river system, that of the Ord River, is found in the subregion, draining plains and hillier areas. Rainfall is 500–800 mm/year. Vegetation includes *Eucalyptus* spp. and several grasses (Mitchell grass, curly bluegrass, golden beard grass, spinifex). There are many localized and rare species in the area associated with the Bungle Bungle Ranges, such as the skink *Leristabunglebungle* Storrand, and many plants, and the Bungle massif is an important refuge (Morton et al. 1995). Although there have been targeted surveys of plants and animals in Purnululu National Park, this species was unknown. The climate is dry monsoonal.

All specimens were collected as immatures and reared in captivity. They were all collected at night on sandstone walls beneath overhangs (Fig. 35B). Females matured when the climate is transitioning from cooler to warmer, and adult males soon after, when it begins to get wetter and hotter (Suppl. material 2: tables S1, S12).

##### Discussion.

*Karaopsyumbubaarnji* sp. nov. can be added to the list of endemics for Purnululu National Park. Despite the area being surveyed for plants and animals previously, this spider went undetected, indicating that there are probably other species, including endemics, that were missed during previous surveys. This is one of the smallest selenopid species, only surpassed by *K.kwartatuma* sp. nov. and *K.markharveyi* sp. nov. *Karaopsyumbu* also occurs in this subregion, but it has not been found in the park.

#### ﻿ Karaops kennerleyorum sp. nov.

Taxon classificationAnimaliaAraneaeSelenopidae

3CE0893F-01CC-5ECA-A7FE-E5C1ECCC7880

https://zoobank.org/5F77D93F-8817-4F2A-969E-4D90A0D8C704

**Figure 38.**
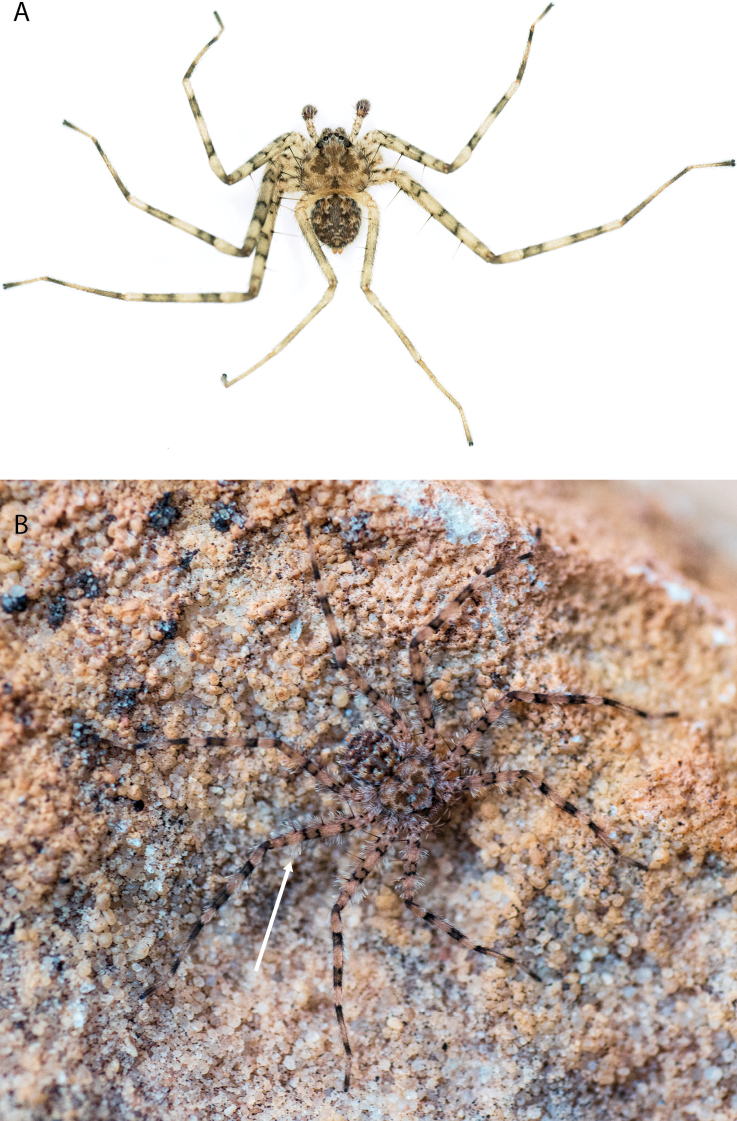
*Karaopskennerleyorum* sp. nov., holotype male, Southern Lost City, Limmen National Park, Northern Territory **A** holotype male (sel_1343, MAGNT A004899) **B** arrow indicates white setal tufts on legs.

**Figure 39.**
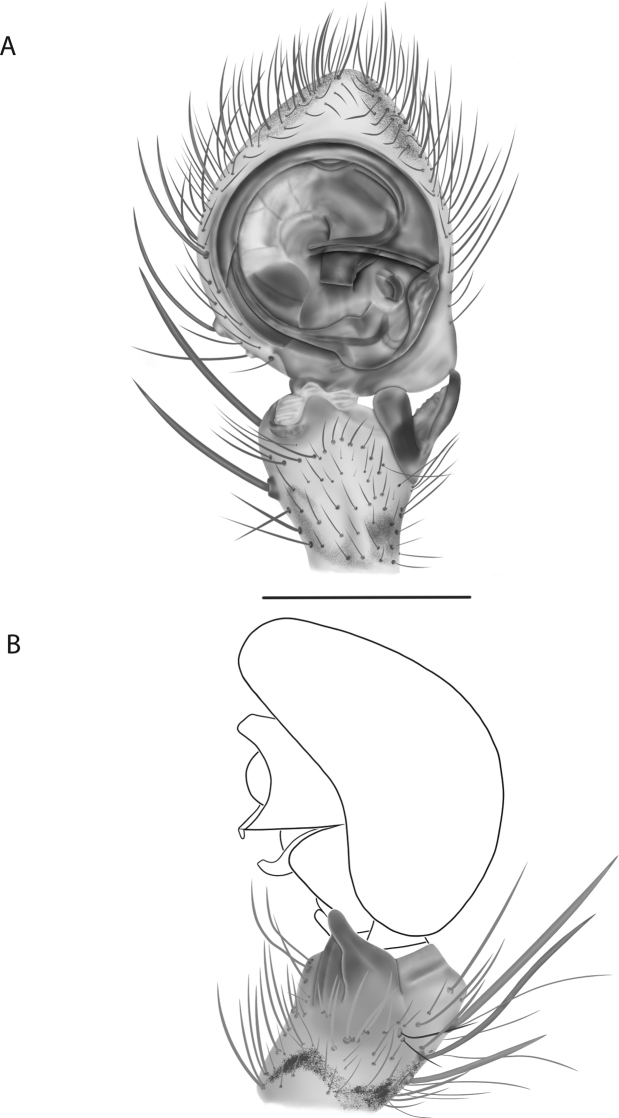
*Karaopskennerleyorum* sp. nov., holotype male, Southern Lost City, Limmen National Park, Northern Territory **A** palp, ventral **B** same, retrolateral. Scale bar: 0.5 mm.

**Figure 40.**
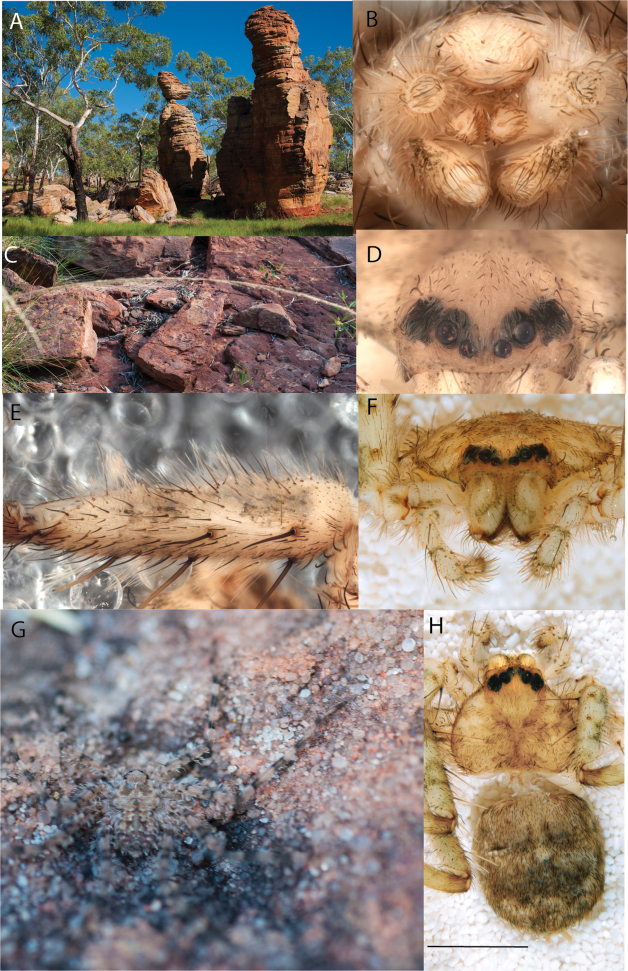
*Karaopskennerleyorum* sp. nov. and *Karaopsmadhawundu* sp. nov. **A***Karaopskennerleyorum* sp. nov. habitat, Southern Lost City, Limmen National Park, Northern Territory **B***Karaopsmadhawundu* sp. nov., holotype female, spinnerets, Gilberton Station, Forsayth, Queensland (QMS 5430) **C** habitat of *Karaopskennerleyorum* sp. nov., Southern Lost City, Limmen National Park, Northern Territory **D***Karaopsmadhawundu* sp. nov., holotype female, eyes, Gilberton Station, Forsayth, Queensland (QMS 5430) **E** same, femur showing claviform white setae **F***Karaopsmadhawundu* sp. nov., paratype female, face, Cobbold Gorge, Queensland (QMS 5413) **G***Karaopskennerleyorum* sp. nov., camouflaged against sandstone, Southern Lost City, Limmen National Park, Northern Territory **H***Karaopsmadhawundu* sp. nov., paratype female, Cobbold Gorge, Queensland (QMS 5413). Scale bar: 5 mm.

Figs 37B , 38A, B , 39A, B , 40G , Maps 1 , 8

##### Material examined.

***Holotype***: Northern Territory • ♂ (reared in captivity); Limmen National Park, Southern Lost City loop trail; vic. 15°48'22.28"S, 135°27'25.19"E; 11 Jun. 2016; S. Crews leg.; under sandstone rocks during the day; sel_1343; SCC16_074; (MAGNT A004899). **Other material examined**: 5 imm., same data as holotype; sel_1344–1348; (MAGNT A004900–A004904).

##### Diagnosis.

*Karaopskennerleyorum* sp. nov. (Fig. 38A, B) can be differentiated from other males by the palps. It is not very similar to any other species, the most similar being *K.yumbubaarnji* sp. nov. (Figs 34A, 35A). The only similarity is that part of the conductor originates anteromedially, is projected ventrally, and the cymbial sheath that covers the embolus connects to this projection on the retrolateral side of the palp. They can be easily differentiated by the shape of the conductor, which in *K.yumbubaarnji* sp. nov. is slightly twisted, narrowed distally, and directed ventrally, and in *K.kennerleyorum* sp. nov., the conductor comes to a point that is directed laterally over the center of the cymbium and connects with the blunted part of the conductor arising from the tegulum. Additionally, the median apophysis of the new species is unique in that it is directed ventrally rather than apical to the palp, is of uniform width, and somewhat smaller than in many other species of the genus.

##### Description.

**Male** (holotype). Total length 4.57. Carapace: length 2.34, width 2.79. Chelicerae: promargin with three teeth, the one closest to base of fang smaller than others, retromargin with two teeth (1-0-1). Eyes: AER slightly recurved, PER recurved; diameters AME 0.17, ALE 0.12, PME 0.22, PLE 0.33; interdistances AME–PME 0.03, PME–ALE 0.17, ALE–PLE 0.27, PME–PME 0.86, ALE–ALE 1.24, AME–AME 0.44, PLE–PLE 1.45. Sternum: length 1.25, width 1.34. Abdomen: length 2.23, width 2.06. Color (in life Figs 38A, 39A, 40G/preserved Fig. 37B): Carapace: pale brown with darker brown patches extended from PLE to middle of carapace, three pairs of spots laterally, one dark patch posteriorly/orangish white, dark patches still visible; setose with patches of white setae behind eye area and lateral to PLEs. Chelicerae: tan, paturon with longitudinal curved mark frontally, setae pale laterally, dark medially. Maxillae: whitish. Labium: dusky, pale distally. Sternum: whitish. Abdomen: dorsally different shades of brown, dark brown laterally, posteriorly, cardiac area brown, red-brown laterally, with some pale patches medially and laterally/pale patches orangey, dark patches dark brown to black; ventrally yellow-gray. Legs: yellowish brown, Cx I with dark stripe dorsally, Cx II, III with two dark stripes dorsally, Tr with dark marks prolaterally and retrolaterally, Fm with pairs of jagged stripes basally, medially, dusky between them, dusky stripe at Pt joint, Pt with dusky annulation at Fm-Pt joint, Ti with two pairs of jagged black stripes, dusky between them, Mt with dusky ring basally and distally, Ta tip dark; Fm spines dark at base, lightened to orange distally, sometimes darkened at tip; spination leg I Fm d 1-1-1, pr 1-1-1, Ti v 2-2-2-2-2-2, Mt v 2-2-2-2; leg II Fm d 1-1-1, pr 0-0-1, Ti v 2-2-2-2-2-2, Mt v 2-2-2-2; leg III Fm d 1-1-1; leg IV Fm d 1-1-1; leg formula 3214 (but see Discussion); measurements leg I 11.44 (3.27, 1.21, 3.28, 2.59, 1.09); leg II 13.30 (4.00, 1.37, 3.93, 2.74, 1.26); leg III 14.80 (4.67, 1.36, 4.01, 3.20, 1.56); leg IV 11.39 (3.51, 0.97, 2.89, 2.60, 1.42). Palp spination Fm d 0-1-2; 2.36 (0.70, 0.39, 0.43, 0.84); Cy with dark marks apically, Ti with dark marks basally; vRTA small, quadrangular in ventral view, dRTA pointed, slanted dorsally (Fig. 39B); Cy oval to triangular; rbcp large; CS covers distal half of E, starting ~ 9 o’clock, and at 3 o’clock coming to a point directed at 9 o’clock, connecting with longer, blunted part of C that arises apicomedially from T; MA small, uniform width, directed ventrally; E long, emerging from small TL, beginning ~ 6 o’clock, ending ~ 2:30 o’clock, curved near tip of Cy (but see Discussion), edge of TL curled toward MA.

**Female.** Unknown.

##### Etymology.

This species is named for the Kennerley family who helped me out greatly toward the end of my fieldwork. Name in genitive case.

##### Distribution.

Known from only the type locality, Limmen National Park, Northern Territory (Fig. 40C, Map 8).

##### Natural history.

*Karaopskennerleyorum* sp. nov. occurs in the McArthur subregion of the Gulf Fall and Uplands bioregion. It comprises spinifex grasslands with eucalypt woodlands (Bastin et al. 2008). The climate is monsoonal, with more rainfall in the north than the south. There appears to be little known about the arthropods of the subregion (Suppl. material 2: tables S1, S13). This species was collected from beneath sandstone rocks during the day.

##### Discussion.

This species is not closely related to any nearby species, but more closely related to species found at Ruby Plains (Fig. 33E; Suppl. material 1) and Purnululu (Figs 35A, B, 37A) in Western Australia. The male matured at the beginning of the hottest months at the start of the wet season. Although leg III is longest and leg IV is shortest, they were both recently re-grown. In the description it is mentioned that the embolus is curved outward near the tip of the cymbium. It is possible that this is an anomaly and will not characterize other specimens of this species.

#### ﻿ Karaops madhawundu sp. nov.

Taxon classificationAnimaliaAraneaeSelenopidae

B8B952FB-A994-5848-944A-4C7D482DC762

https://zoobank.org/EF6E49FA-C914-42B1-9B64-01EDBCFCCBB8

**Figure 41.**
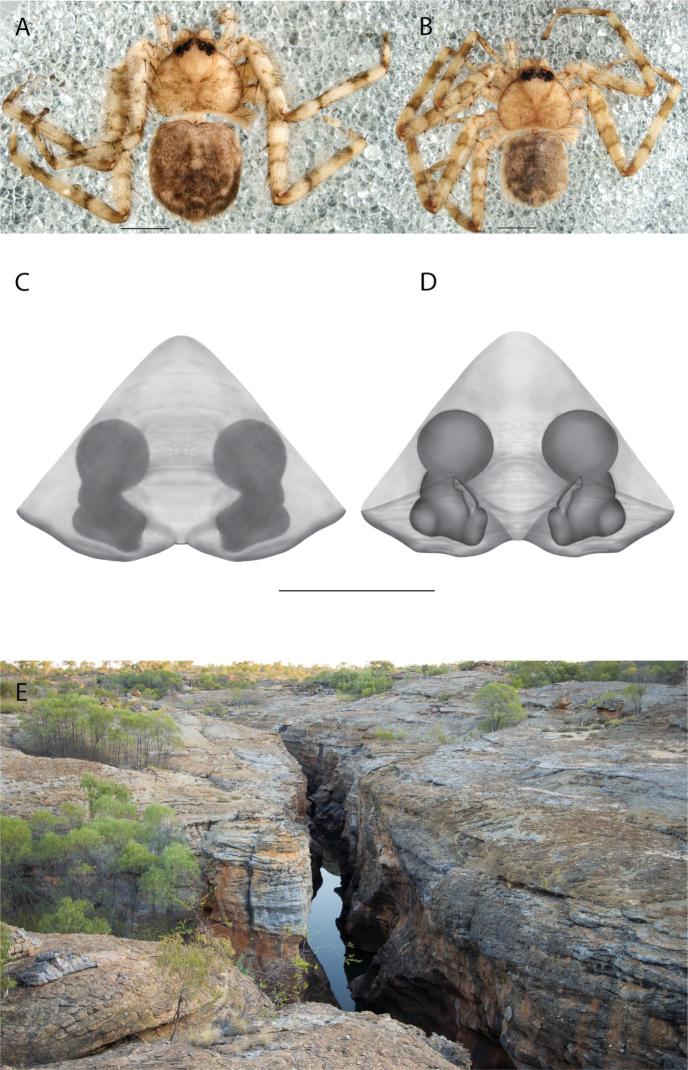
*Karaopsmadhawundu* sp. nov., Cobbold Gorge and Gilberton, Queensland **A***Karaopsmadhawundu* sp. nov., holotype female, Cobbold Gorge (QMS 5430) **B***Karaopsmadhawundu* sp. nov., paratype female, Gilberton Station (QMS 5413) **C***Karaopsmadhawundu* sp. nov., holotype female, Cobbold Gorge (QMS 5430) **D** same, endogyne **E** Cobbold Gorge, Queensland, habitat of *Karaopsmadhawundu* sp. nov. (Photo: J. DeJong). Scale bars: 0.5 mm (**C, D**); 2 mm (**A, B**).

Figs 40B, D–F, H , 41A, B , 41C–E , Maps 1 , 8

##### Material examined.

***Holotype***: Queensland • ♀; Forsayth, Gilberton Station; -19.225431, 143.644531; 19 Sep. 2017; S. Zozaya leg.; SMZ0955; (QMS 5430). ***Paratypes***: 3♀; Cobbold Gorge, west of Forsayth; -18.816908, 143.406830; 20 Sep. 2017; S. Zozaya leg.; SMZ0956; (QMS 5413). **Other material examined**: 4 imm.; same data as holotype; (QMS 5425) • 1 imm.; same data as paratype; (QMS 5423).

##### Diagnosis.

**Figure 42.**
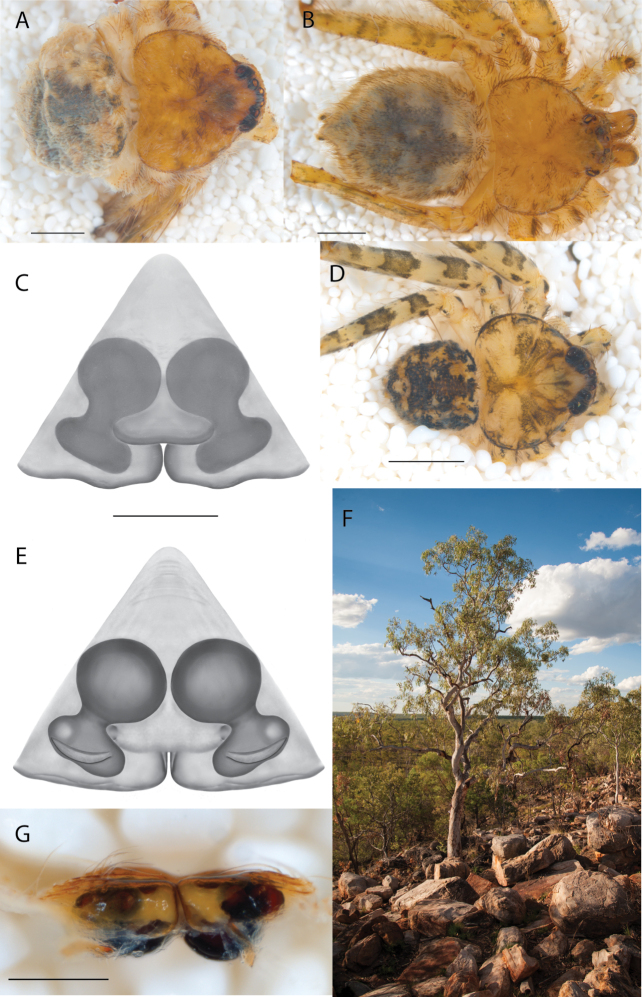
*Karaopsmareeba* sp. nov. and *Karaopsmarkharveyi* sp. nov. **A***Karaopsmareeba* sp. nov., paratype female, Desailly Range, Queensland (QMS 120908) **B***Karaopsmareeba* sp. nov., holotype female, Desailly Range, Queensland (QMS 110872) **C** same, epigyne **D***Karaopsmarkharveyi* sp. nov., 4.6 km from Drysdale River Road, Gibb River Road, Western Australia, holotype female (sel_1249, WAM T155625) **E***Karaopsmareeba* sp. nov., holotype female, endogyne, Desailly Range, Queensland (QMS 110872) **F** sandstone ridge habitat of *Karaopsmarkharveyi* sp. nov. **G***Karaopsmareeba* sp. nov., holotype female, epigyne, caudal, Desailly Range, Queensland (QMS 110872). Scale bars: 0.5 mm (**C, E, G**); 2 mm (**A, B, D**).

Females of *Karaopsmadhawundu* sp. nov. (Figs 40F, H, 41A, B) are similar to those of *K.markharveyi* sp. nov. and *K.mareeba* sp. nov. by the large, round accessory bulbs (Figs 41D, 42E, 44C–F, 47E), but the new species can be differentiated from these congeners by the distance between the accessory bulbs: The distance is small in *K.markharveyi* sp. nov. (except for one variant (Fig. 44F), and that can be separated by the posterior separation of the lateral lobes) and *K.mareeba* sp. nov. but nearly one accessory bulb diameter apart in *K.madhawundu* sp. nov. Additionally, *K.madhawundu* sp. nov. is much larger than *K.markharveyi* sp. nov. (7.00–7.86 vs. 3.07–4.09).

##### Description.

**Female** (holotype) (Figs 40D, 41A). Total length 7.00. Carapace: length 3.15, width 3.60. Chelicerae: promargin with three teeth, middle one largest, retromargin with two teeth. Eyes (Fig. 40D): AER slightly recurved, PER recurved; diameters AME 0.18, ALE 0.12, PME 0.25, PLE 0.29; interdistances AME–PME 0.05, PME–ALE 0.17, ALE–PLE 0.18, PME–PME 1.18, ALE–ALE 1.62, AME–AME 0.54, PLE–PLE 1.44. Sternum: length 1.62, width 1.89. Abdomen: length 3.85, width 4.24. Color: Carapace: brownish yellow with pair of dark marks lateromedially, three dark marks each on lateral margins, dark area posteromedially, setose, with dark, slender setae, dispersed, dark, thick setae, patches of pale setae. Chelicerae: yellowish white, paturon with a longitudinal curved mark frontally, setae paler laterally, darker anteriorly. Maxillae: yellowish white. Labium: gray, pale distally. Sternum: yellowish. Abdomen: dorsal anterior third pale yellow-brown, pale cardiac area, dark lateral to cardiac area giving appearance of two vertical lines extended into first third, wide, dark chevron posterior to this area, extended from center to lateral margins, nearly to tip of abdomen, narrow, pale area near posterior, dark posteriorly and posterolaterally; ventrally grayish yellow. Spinnerets: with dusky markings laterally (Fig. 40B). Legs: pale yellowish brown, ventral tufts of white, clavate setae (Fig. 40E), Cx with prolateral dark mark, Tr with prolateral dark spot, Fm all with dusky area at Tr-Fm joint, Fm I with pair of dusky lines medially forming a dusky annulation, Pt dusky at Fm-Pt joint, Ti with dark annulations at Pt-Ti joint, and between that and Ti-Mt joint, Mt with dark annulation at both Ti-Mt joint and Mt-Ta joint, Ta dark at tip; spines dark basally, pale distally; spination leg I Fm d 1-1-1, pr 1-1-0, Ti v 2-2-2-2-2, Mt v 2-2-2-2; leg II Fm d 1-1-1, Ti v 2-2-2-2-2, Mt v 2-2-2-2; leg III Fm d 1-1-1; leg IV Fm d 1-1-1; leg formula 2431; measurements leg I 12.49 (3.60, 1.35, 3.49, 2.70, 1.35); leg II 14.54 (4.68, 1.71, 3.52, 3.44, 1.20); leg III 12.62 (3.85, 1.08, 3.23, 2.96, 1.50); leg IV 14.25 (5.23, 1.46, 3.83, 2.25, 1.49). Palp: spination Fm d 0-1-3; 3.24 (1.06, 0.68, 0.60, 0.90); claw with six teeth. Epigyne: EP triangular; MF with small, oval depression; LLs fused; COs inconspicuous, located at posterolateral margin of depression. Endogyne: CDs very short; ABs large, round; S smaller, more oval; FDs directed anterolaterally.

**Male.** Unknown.

##### Variation.

(*n* = 5) Length ranges from 7.00–7.86. The leg spination of one of the paratypes differs from the type as follows: leg I Fmpr 1-1-1 on L and R; leg II Fmpr 0-0-1; Mt v 2-2-2-2-2-2.

##### Etymology.

The name is from two words used to describe the Cobbold Gorge area in Wamin, Madha (mountain) and Wúndu (forest), spoken by the Ewamian people, the traditional owners of the Cobbold Gorge area (Fig. 41E). Noun in apposition.

##### Distribution.

Known from Northeast Queensland (Map 8).

##### Natural history.

*Karaopsmadhawundu* sp. nov. is found in the Kidston subregion of the Einasleigh Uplands bioregion. This bioregion consists of savanna and woodland on a plateau (Bastin 2008). The wet season occurs from December–March. Adult females, immatures, and a penultimate male were all collected in mid-September, when the climate is drier and slightly cooler than other times of year (Suppl. material 2: table S1).

##### Discussion.

The Einasleigh Uplands are home to many unique habitats, including gorges and caves, that are home to many endemic species of plants and animals (Bastin 2008). Little is known of the terrestrial arthropod fauna with the exception of a few wetland insect species. It is likely that arthropods will mirror the endemism of other organisms found in the area.

#### ﻿ Karaops mareeba sp. nov.

Taxon classificationAnimaliaAraneaeSelenopidae

FCD1846C-DF5C-56BC-982D-AE03952FBFF7

https://zoobank.org/F0CD75DB-16FF-4526-932F-DBD5DECE9264

Fig. 42A–C, E, G , Maps 1 , 8

##### Material examined.

***Holotype***: Queensland • ♀; Desailly Range, base on south side, 16°28'56.8"S, 144°53'03.2"E; 6 Sep.–18 Oct. 2008; R. Raven, G.B. Monteith leg.; (QMS 110872). ***Paratype***: ♀; same data as holotype (QMS 120908).

##### Diagnosis.

The endogyne of *Karaopsmareeba* sp. nov. (Fig. 42A, B) is somewhat similar to that of *K.madhawundu* sp. nov. by the large, round accessory bulbs, but medially they are nearly in contact with one another in *K.mareeba* sp. nov. (Figs 41C, D, 42C, E) but separated in *K.madhawundu* sp. nov. *Karaopsmareeba* sp. nov. also differs in having the lateral lobes distinct posteriorly and a lobe in the median field whereas *K.madhawundu* sp. nov. has a depression in the median field, and there is no clear separation of the lateral lobes.

##### Description.

**Female** (holotype). Total length 7.06. Carapace: length 2.96, width 3.40. Chelicerae: promargin with three teeth, retromargin with one tooth on left side, two teeth on right. Eyes: AER slightly recurved, PER recurved; diameters AME 0.13, ALE 0.08, PME 0.19, PLE 0.27; interdistances AME–PME 0.04, PME–ALE 0.23, ALE–PLE 0.18, PME–PME 0.84, ALE–ALE 1.43, AME–AME 0.45, PLE–PLE 1.50. Sternum: length 1.50, width 1.46. Abdomen: length 4.10, width 3.15. Color: Carapace: brownish orange, three marks each on lateral margins, somewhat faded, flat, white setae around eyes, patches elsewhere interspersed with slender, dark setae. Chelicerae: brownish orange, paturon with longitudinal curved mark frontally, setae white, long. Maxillae: yellowish white. Labium: gray, pale distally. Sternum: tan. Abdomen: dorsally grayish, yellow around perimeter, faded, markings indistinguishable; ventrally grayish yellow. Spinnerets: black dorsally. Legs: orangish brown, Cx with jagged, dusky mark prolaterally, Tr with pr dark spot, Fm with two jagged lines basally and two apically, forming annulations, orange on both Fm and Pt at joint, Pt with dark mark ventrally, Ti orange basally and apically, dark mark on Ti at Pt-Ti joint, dark annulation apically, Mt orange with dark annulations at Ti-Mt joint and Mt-Ti joint, Ta orange and dusky at tip; spination leg I missing; leg II Fm d 1-1-1, pr 0-0-1, Ti v 2-2-2-2-2-2, Mt v 2-2-2-2; leg III Fm d 1-1-1, pr 0-0-1; leg IV Fm d 1-1-1, pr 0-0-1, rl 0-0-1; measurements leg I missing; leg II 11.44 (3.94, 1.30, 2.96, 2.16, 1.08); leg III 12.01 (3.85, 1.15, 3.15, 2.66, 1.02); leg IV 11.21 (3.82, 0.90, 2.78, 2.50, 1.22). Palp Fm spination d 0-1-3; 2.66 (0.82, 0.48, 0.54, 0.83); claw with five teeth. Epigyne: EP triangular; MF with truncate lobe extending slightly posteriorly over LLs; LLs separated posteriorly; COs at lateral edges of lobe (Fig. 23D). Endogyne: CDs very short; ABs large, round; S small, oval; FDs directed laterally.

**Male.** Unknown.

##### Variation.

(*n* = 2) Paratype with two very small teeth on left promargin and two regular size teeth on right; retromargin with two teeth on left, three on right.

##### Etymology.

This species is named after the Shire of Mareeba, where the type locality is located. Noun in apposition.

##### Distribution.

Known only from the type locality, Desailly Range, Queensland (Map 8).

##### Natural history.

*Karaopsmareeba* sp. nov. is found in the Hodgkinson Basin subregion of the Einasleigh Uplands bioregion (Bastin 2008). This bioregion is savanna and woodland on a plateau. There is a wet season December–March. Adult females were collected between September and October when the climate is drier and slightly cooler than other times of year (Suppl. material 2: table S1).

##### Discussion.

The Einasleigh Uplands are home to many unique habitats, including gorges and caves, that are home to many endemic species of plants and animals (Bastin 2008). Little is known of the terrestrial arthropod fauna with the exception of a few wetland insect species. Endemism is likely in arthropods found in the subregion.

#### ﻿ Karaops markharveyi sp. nov.

Taxon classificationAnimaliaAraneaeSelenopidae

5ADC6A85-461C-521E-9FD2-E36D7D84A334

https://zoobank.org/01C8D958-A493-45B8-9C3F-6F6A83AE44EC

**Figure 43.**
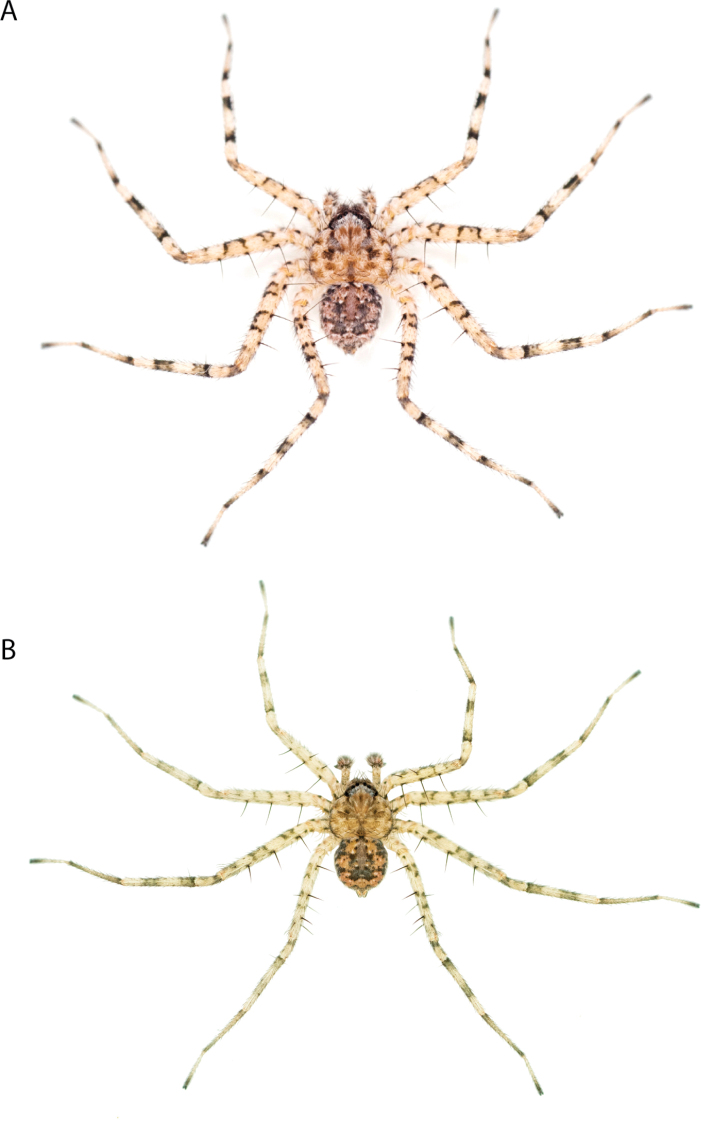
*Karaopsmarkharveyi* sp. nov. **A** paratype female, Gibb River Road, Western Australia (sel_1253, WAM T155629) **B** paratype male, ~ 4.6 km from Drysdale River Road, Gibb River Road, Western Australia (sel_1250, WAM T155626).

**Figure 44.**
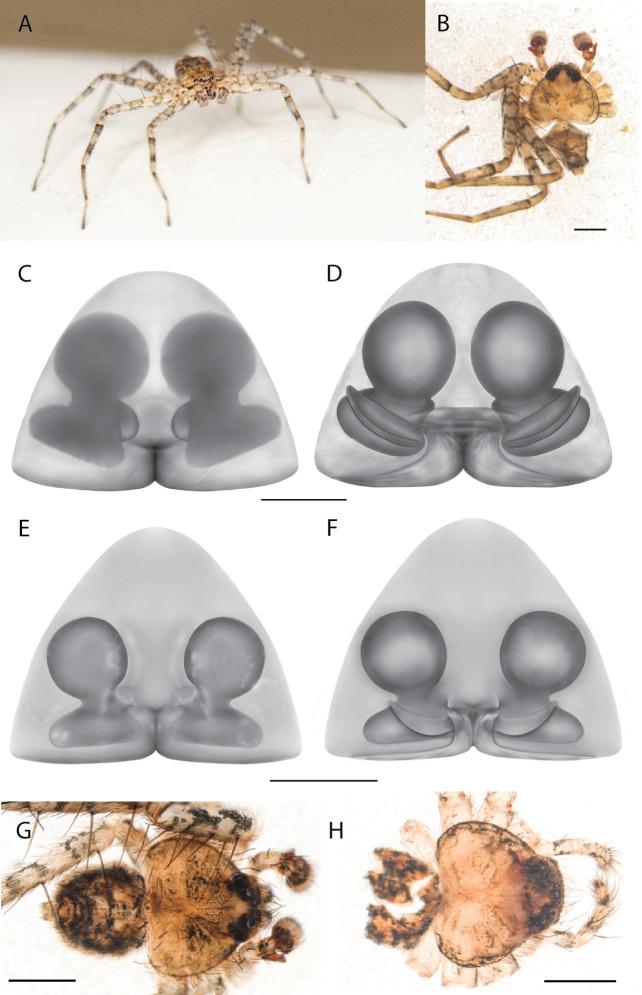
*Karaopsmarkharveyi* sp. nov. **A** paratype male, 4.6 km from Drysdale River Road, Gibb River Road, Western Australia (sel_1250, WAM T155626) **B** adult male, Judbarra National Park, Northern Territory (sel_1329, MAGNT A004885) **C** holotype female, epigyne, ~ 4.6 km from Drysdale River Road, Gibb River Road, Western Australia (sel_1249, WAM T155625) **D** same, endogyne **E** paratype female, epigyne, Gibb River Road, Western Australia (sel_1253, WAM T155629) **F** same, endogyne **G** adult male, Judbarra National Park, Northern Territory (sel_1330, MAGNT A004886) **H** adult female, Judbarra National Park, Northern Territory (sel_1332, MAGNT A004888). Scale bars: 0.5 mm (**C–F**); 1 mm (**B, G, H**).

**Figure 45.**
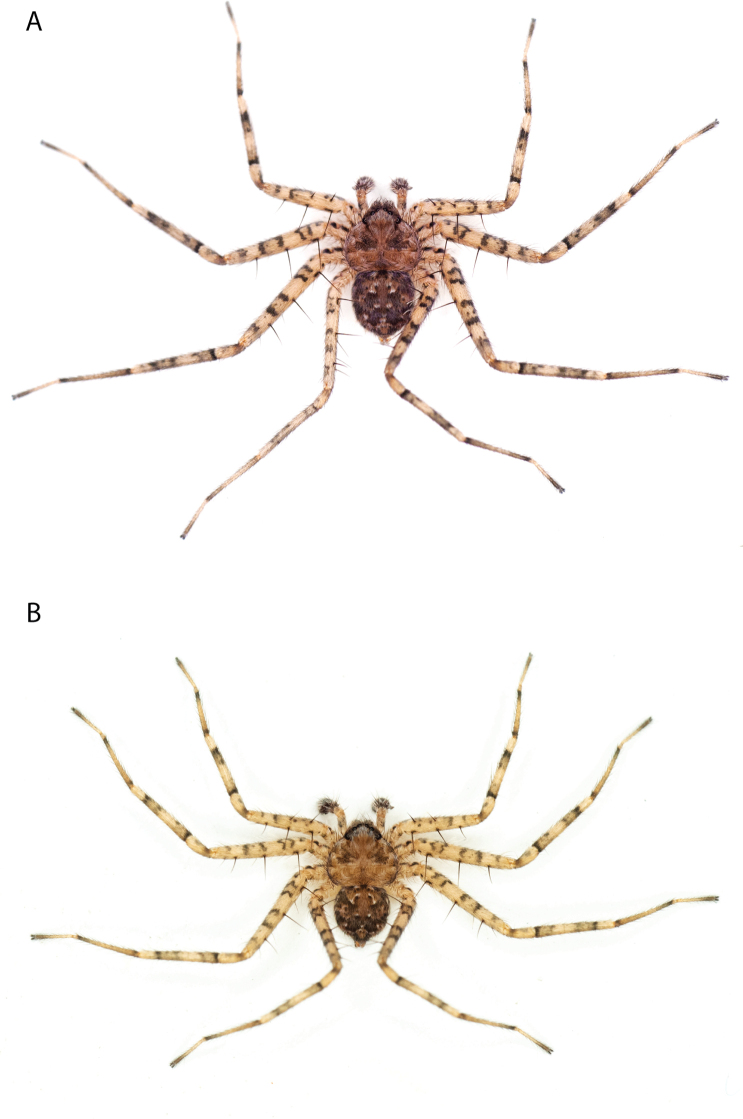
*Karaopsmarkharveyi* sp. nov. from Judbarra National Park, Northern Territory **A** adult male (sel_1330, MAGNT A004886) **B** adult male (sel_1329, MAGNT A004885).

Figs 42D, F , 43A, B , 44 A–H , 45 A, B , Maps 1 , 8

##### Material examined.

***Holotype***: Western Australia • ♀ (reared in captivity); Gibb River Road, ~ 4.6 km from Drysdale River Road, heading east; 16°6'12.69"S, 126°34'44.60"E; ~ 491 m; 22 May 2016; S. Crews, J. DeJong leg.; sandstone outcrop on south side of road; sel_1249; SCC16_050; (WAM T155625). ***Paratypes***: ♂ (reared in captivity); same data as previous; sel_1250; (WAM T155626) • ♀ (reared in captivity); Gibb River Road, heading east; 15°57'37.61"S, 126°52'57.32"E; ~ 410 m; 12 May 2016; S. Crews, J. DeJong leg.; sandstone outcrop on south side of road; sel_1253; SCC16_051; (WAM T155629) • Northern Territory • ♀, ♂ (reared in captivity); Judbarra National Park, Joe Creek Walk; 15°36'20.63"S, 131°4'45.02"E; 1 Jun. 2016; S. Crews, J. DeJong leg.; under pink sandstone rocks beneath escarpment; sel_1330, 1332; SCC16_070; (MAGNT A004886, A004888). **Other material examined**: Western Australia • 2 imm., same data as holotype; sel_1248, 1251; (WAM T155624, T155627) • 1 imm.; same data as sel_1253; sel_1251; (WAM T155627) • ♂ (reared in captivity); same data as previous; sel_1252; (WAM T155628) • 2♂ (reared in captivity); same data as sel_1330, 1332; sel_1329, 1331; (MAGNT A004885, A004887).

##### Diagnosis.

Females of *Karaopsmarkharveyi* sp. nov. are most similar to *K.madhawundu* sp. nov. and *K.mareeba* sp. nov. by the large, round accessory bulbs (Figs 44C–F; 47D, E) but can be differentiated by the much smaller size: females of the new species average 3.58 (*n* = 3), whereas *K.madhawundu* sp. nov. have a median size of 7.31 (*n* = 3) and *K.mareeba* sp. nov. a median size of 7.63 (*n* = 2). Additionally, the spermathecae of *K.mareeba* sp. nov. are directed medially at ~ 45°angle, and the copulatory openings are beneath a lobe (42C, E).

**Figure 46.**
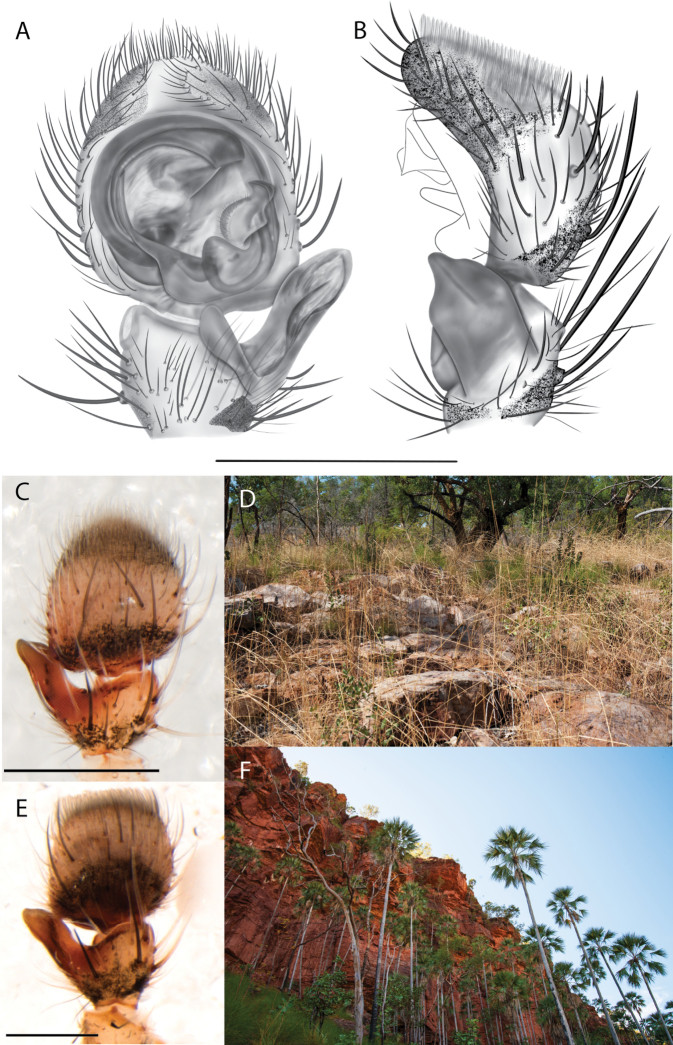
*Karaopsmarkharveyi* sp. nov. and *Karaops* sp. **A** paratype male, palp, ventral, Gibb River Road, ~ 4.6 km from Drysdale River Road, Western Australia (sel_1250, WAM T155626) **B** same, retrolateral **C** same, dorsal **D** habitat of *Karaops* sp., Timber Creek, Nackeroo Lookout, Northern Territory **E***Karaopsmarkharveyi* sp. nov., palp, dorsal, tilted forward to see chemosensory brush, Judbarra National Park, Northern Territory (sel_1329, MAGNT A004885) **F** habitat of *Karaopsmarkharveyi* sp. nov., Judbarra National Park, Northern Territory. Scale bars: 0.5 mm.

The male palps do not resemble any other known Australian selenopid (Fig. 46A–C, E); however, the males are unknown for *Karaopsmadhawundu* sp. nov. and *K.mareeba* sp. nov., the two species that most closely resemble this new species, and molecular data suggest they are closely related to one another (Suppl. material 1). The cymbium has a superficial resemblance to that of *K.yumbubaarnji* sp. nov. by the chemosensory setae on the tip (Fig. 36A, B), and the body size of both species is small, but the other features of the palp are not similar, and the abdominal pattern differs between the two (Figs 34A, 35A, 44B, 45A, B).

##### Description.

**Figure 47.**
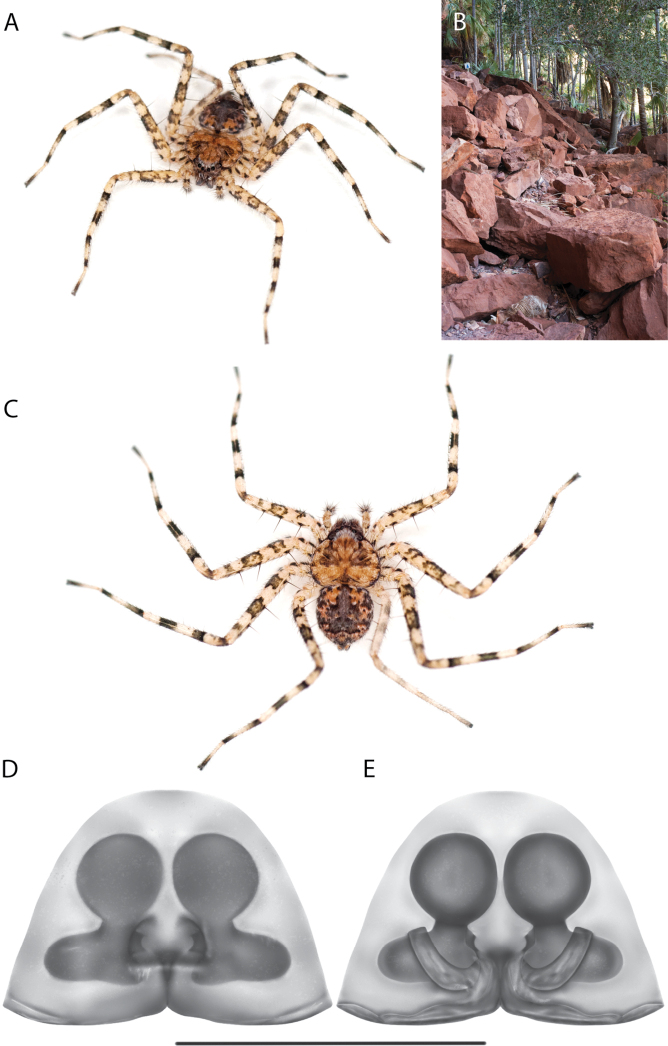
*Karaopsmarkharveyi* sp. nov. **A** holotype female, Gibb River Road, ~ 4.6 km from Drysdale River Road, Western Australia (sel_1249, WAM T155625) **B** habitat, Judbarra National Park, Northern Territory **C** holotype female, Gibb River Road, ~ 4.6 km from Drysdale River Road, Western Australia (sel_1249, WAM T155625) **D** epigyne, Judbarra National Park, Northern Territory (sel_1332, MAGNT A004888) **E** same, endogyne. Scale bar: 0.5 mm.

**Female** (holotype). Total length 4.09. Carapace length 1.99, width 2.39. Chelicerae: promargin with three teeth, retromargin with two teeth (1-0-1). Eyes: AER recurved; PER strongly recurved; diameters, AME 0.12, ALE 0.08, PME 0.16, PLE 0.26; interdistances AME–PME 0.04, PME–ALE 0.11, ALE–PLE 0.17, PME–PME 0.72, ALE–ALE 1.01, AME–AME 0.38, PLE–PLE 1.22. Sternum length 0.88, width 1.18. Abdomen length 2.10, width 1.52. Color (in life Figs 43A, 47A, C/preserved Figs 42D, 44H): Carapace: reddish brown with two large dark patches behind PLEs extending to middle of carapace next to furrow, some dark patches laterally and posterior to furrow, white setae behind eyes, and in patches on carapace. Chelicerae: yellow-brown, anterior black except medially. Maxillae: yellow, dusky on outer edge. Labium: brownish yellow, pale distally. Sternum: pale yellowish white. Abdomen: dorsally dark brown with orangish spots anterolaterally, mediolaterally, posterolaterally, posteriorly, all but posterior patches with black dot, all orangish areas with tufts of white setae on edges, posterior dark; ventrally yellowish white. Anterior spinnerets: yellowish brown, posterior spinnerets black except ventrally yellowish brown. Legs: yellowish with black marks dorsally and ventrally on Cx I–III, dark mark anteriorly on all Tr,, annulations on all legs, only completely encircle legs at Mt, centers open but dusky on Fm, Pt, and Fm-Pt joint, dark annulations on Ti at Pt-Ti joint and medially, dark annulations on Mt at Ti-Mt joint and at Mt-Ta joint, Ta tips dark; flat, white setae enlarged distally ventrally on all Fm, Pt, Ti, annulation at Pt-Mt joint, tips of Ta dark; spination leg I Fm d 1-1-1, Ti v 2-2-2-2-2, Mt v 2-2-2; leg II Fm d 1-1-1, Ti v 2-2-2-2-2, Mt v 2-2-2; leg III Fm d 1-1-1, Ti v 1-1, Mt v 2-0, 5 pair of small spines near Mt-Ta; leg IV Fm d 1-1-1, rl 0-0-1, rl 0-1-1, Ti v 1-1-1, Mt v 1-1; leg formula 2341; measurements leg I 7.43 (2.41, 0.90, 2.00. 1.23, 0.89); leg II 9.78 (3.82, 0.88, 2.34, 1.64, 1.10); leg III 9.56 (3.40, 0.83, 2.39, 1.18, 1.76); leg IV 8.33 (2.94, 0.60, 2.03, 1.65, 1.11). Palp spination Fm d 0-1-2; 1.84 (0.59, 0.36, 0.27, 0.63); claw with five teeth; dark spots dorsolaterally and ventrally on Fm. Epigyne: EP rounded triangular; MF slightly depressed; LL conspicuous posteriorly, touching; COs laterally in depression. Endogyne: CDs very short; ABs large, round; S oblong; FDs large, directed anterolaterally; small pdf.

**Figure 48.**
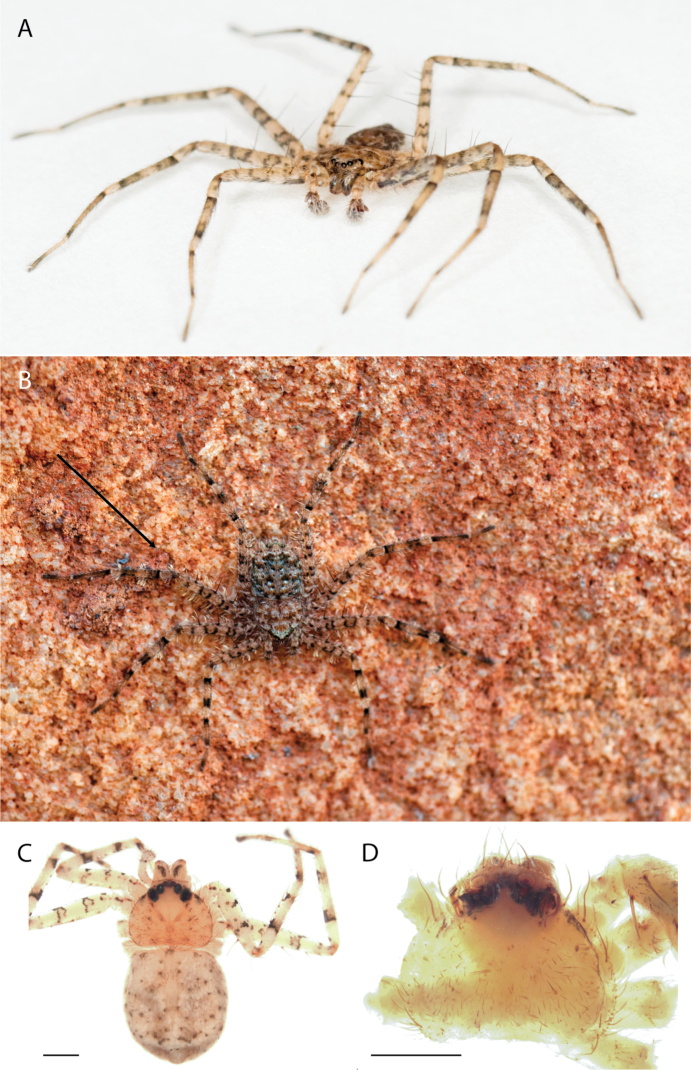
*Karaopsmarkharveyi* sp. nov. and members of the Pilbara-Gascoyne species group **A***Karaopsmarkharveyi* sp. nov., adult male, Judbarra National Park, Northern Territory (sel_1329, MAGNT A004885) **B***Karaops* sp., Timber Creek, Nackeroo Lookout, Northern Territory. Arrow indicates setal tufts **C***Karaopsburbidgei*, paratype female, Barrow Island (WAM T76698) **D***Karaopsburbidgei*, holotype male, Barrow Island (WAM T55000). Scale bars: 1 mm.

**Male** (paratype). Total length 3.10. Carapace length 1.71, width 2.07. Chelicerae: promargin with three teeth, retromargin with two teeth (1-0-1). Eyes: AER recurved, PER strongly recurved; diameters, AME 0.12, ALE 0.06, PME 0.16, PLE 0.22; interdistances AME–PME 0.02, PME–ALE 0.11, ALE–PLE 0.15, PME–PME 0.63, ALE–ALE 0.88, AME–AME 0.33, PLE–PLE 1.09. Sternum: length 0.89, width 1.16. Abdomen length 1.39, width 1.29. Color (in life Figs 43B, 44A, 45A, B, 48A/preserved Fig. 44B, G): Carapace: reddish brown with two large dark patches originating behind PLEs, extending to middle of carapace next to furrow, some dark patches laterally, posterior to furrow, white setae behind eyes, in patches on carapace/yellow-brown, dark patches visible but dispersed, white setae not as conspicuous. Chelicerae: yellowish, anterior black except basolaterally, setae pale, darkened towards middle. Maxillae: yellowish with black marks anterolaterally. Labium tan, paler distally. Sternum: yellowish white. Abdomen dorsally brownish orange with dark areas medially, laterally, with four dots anteriorly, small patches of white setae at edges of dark medial mark; ventrally whitish gray. Spinnerets: anterior yellowish brown, posterior black except ventrally yellowish brown. Legs: yellowish with black marks anteriorly and posteriorly on Cx, anteriorly on Tr, black bands and spots on Fm, Pt dusky at Fm-Pt joint ventrally, black annulation at Pt-Ti joint, two additional dusky areas, dark annulation on Mt at Ti-Mt joint and at Mt-Ta joint, Ta dark at tip; white setae enlarged distally ventrally on Fm, Pt, Ti; spination leg I Fm d 1-1-1, pr 0-0-1, rl 0-0-1, Ti v 2-2-2-2-2-2, Mt v 2-2-2-2; leg II Fm d 1-1-1, pr 0-0-1, Ti v 2-2-2-2-2-2, Mt v 2-2-2-2; leg III Fm d 1-1-1, Ti v 2-2; leg IV Fm d 1-1-1, Ti v 2-2; leg formula 3241; measurements leg I 7.23 (2.19, 0.73, 1.85, 1.48, 0.98); leg II 8.71 (2.73, 0.79, 2.18, 1.89, 1.12); leg III 9.29 (2.99, 0.82, 2.33, 2.04, 1.11); leg IV 7.97 (2.60, 0.62, 1.84, 1.95, 0.96). Palp: spination Fm d 0-0-1; 1.73 (0.63, 0.22, 0.27, 0.61); dark spot retrolaterally on Fm, retrolaterally and dorsally on Ti, Cy with dark marking basodorsally, apically; dRTA very large, much larger than vRTA, wide, nearly as wide as Ti, pointed at tip, in ventral view, convex medially, plicate distally, vRTA narrow, spoon shaped in ventral view; rbcp smallish; Cy oval in ventral view, laterally, can see indentation along top with patch of chemosensory setae (Fig. 46A, B); C large, heavily sclerotized, arced along top, similar to a wave, obscuring ~ 1/2 of E in CS, C covered by TS; E arises from large TL with anterior extension, hook shaped, originating at 7 o’clock, ending at 1 o’clock; MA large, roundish base, with small lobe retrolaterally, tapers only slightly to tip shaped like a bird’s head, heavily sclerotized at base, base covered with Sp.

##### Variation.

Females (*n* = 3): In sel_1253, the AME and PME are equal in size, but in sel_1249 and sel_1332, the PME are larger than the AME. Leg III is longest in sel_1253, leg II is longest in sel_1249; however, leg II and leg III are very similar in length. Body length 3.07–4.09. Colors slightly vary, sel_1253 is paler than sel_1249. There is a lot of variability in genitalia – in fact, of the three, none are identical (Figs 44C–F, 47D, E). In sel_1249 the accessory bulbs are very close together, the accessory bulbs are slanted anteriorly from medial to lateral, in sel_1253 the accessory bulbs are much further apart – nearly 3/4 of one accessory bulb diameter. The spermathecae are horizontal. In sel_1249, the copulatory openings are located in a small depression, in sel_1253, there is not much of a depression, and in sel_1332, the depression more conspicuous, uterus externus and pdf quite different, the accessory bulbs are very close together, the spermathecae are horizontal, and the fertilization ducts are of a completely different shape.

Males (*n* = 4): In sel_1329, the AME=PME, whereas in sel_1250 and sel_1330, the PME are larger than the AME, and there is some variation in leg spination. Body length is 3.10–3.36. sel_1250 is a little paler, more orangey pink, but the pattern the same.

##### Etymology.

This species is named after Mark S. Harvey, friend, mentor, and person responsible for getting me into the mess of Strayan selenopids. Name in genitive case.

##### Distribution.

Known from along the Gibb River Road, northeastern Western Australia (Fig. 42F), and Judbarra National Park, northwestern Northern Territory (Figs 46F, 47B) (Map 8).

##### Natural history.

The collecting localities for this species occur in the Pentecost subregion of the Central Kimberley, the Mitchell subregion of the Northern Kimberley (but very close to a junction with subregion Pentecost from the Central Kimberley region and the Berkeley subregion of the North Kimberley), and the Victoria Bonaparte bioregion and the Keep subregion. The Pentecost subregion has a dry, hot tropical and sub-humid to semi-arid climate with summer rainfall of 750–1000 mm. The area is a savannah woodland of eucalypts and hummock grasses. These spiders were found under sandstone, and it is suggested that these sandstone communities may be areas of high diversity. There have been no systematic faunal surveys of the area, and ecological and life history data of organisms are missing.

The climate of the Mitchell subregion of the Northern Kimberley bioregion is dry, hot tropical to sub-humid with 1100–1500 mm of mostly summer rain. The area is home to many endemic vertebrates and plants, though little is known about the terrestrial arthropods. Like the Pentecost subregion of the Central Kimberley, it is thought that the sandstone communities may be highly diverse. The Berkeley subregion of the North Kimberley bioregion has a dry, hot tropical sub-humid climate with high summer rainfall (Graham 2001g).

The third locality is the Keep subregion of the Victoria Bonaparte bioregion. The part of this bioregion in Western Australia is not divided into subregions. In the Northern Territory, this is considered the Keep subregion. Judbarra National Park is in the Victoria Basin and has many sandstone ranges. It has a tropical semi-arid climate with summer monsoons.

All specimens were collected as immatures beneath sandstone rocks during the day or on/under the rocks at dusk in the drier, cooler time of the year. The were reared in captivity, with most maturing in the wetter, hotter time of the year. Although this is a small sampling, the females matured after almost all of the adult males were dead. This could be indicative of many things – in captivity, the molting/feeding/growing does not reflect what happens in nature, there are other males that were not collected that would overlap with the females, in nature the females would live until the following season when males matured, etc. Given what we know from other studies, it is likely that there are adults all year round, and overlaps do occur at different times which is not reflected in the small sample (Suppl. material 2: tables S1, S14).

##### Discussion.

Given the dissimilarity of the female genitalia of specimens from the three different localities, it was assumed that they were three different species. Therefore, finding corresponding differences in the males was expected. However, there are no differences in the males, including the palps, which have been thoroughly examined and expanded. DNA data indicate that there is no mixing between the three populations, and that they may be related to a species from Timber Creek collected at Nackeroo Lookout (Figs 46D, 48B; Suppl. material 1). Some analyses even render the Judbarra population and the Gibb populations paraphyletic (Suppl. material 1). Because there are no corroborating morphological data, these three populations are currently considered to be the same species, but with more sampling, this is likely to change. These are among the smallest Selenopidae, with the male paratype being the smallest known (3.10 mm).

### The Pilbara-Gascoyne species group

#### ﻿ Karaops burbidgei

Taxon classificationAnimaliaAraneaeSelenopidae

Crews & Harvey, 2011

1E81B6E8-9703-5CFB-B614-AA9045B5D46D

**Map 9.**
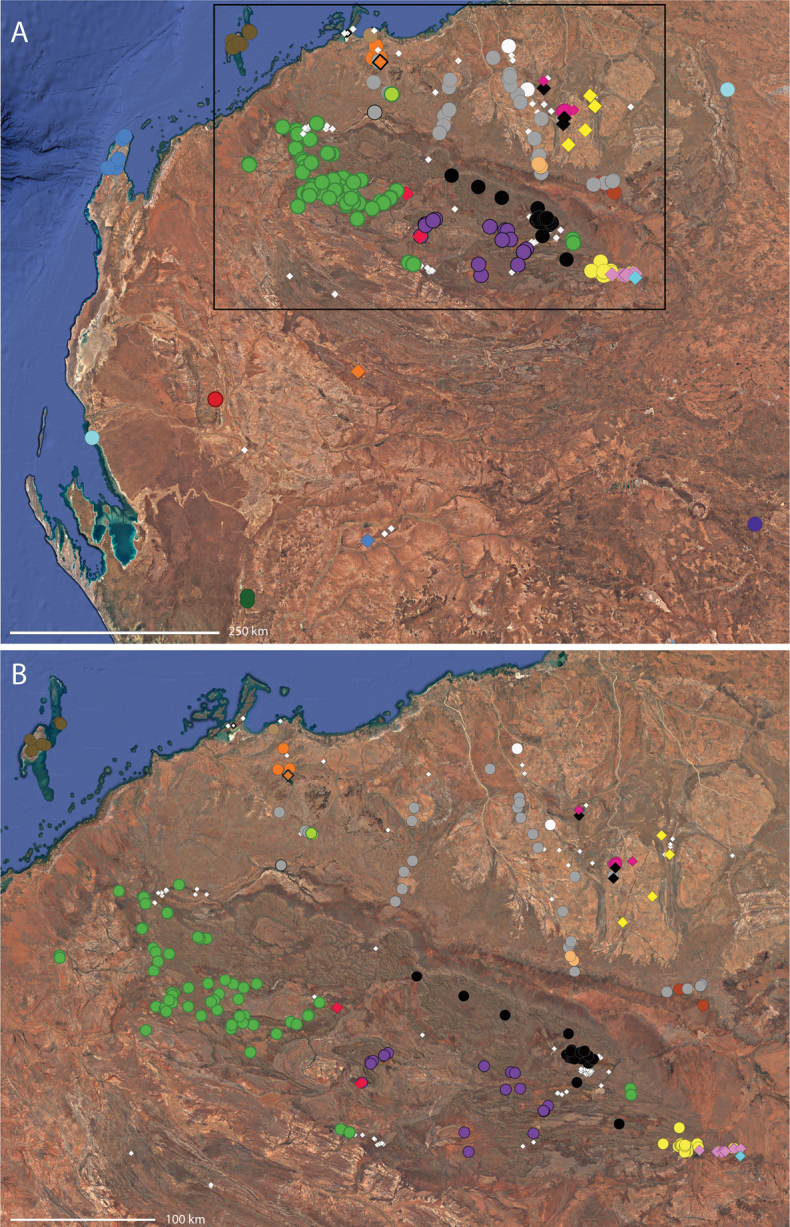
Species of the Pilbara-Gascoyne species group **A** diamonds (juveniles): blue = Erawandoo Hill, orange = Mt. Augustus, red = Nammuldi, yellow = Hillside-Corunna-Glenn Herring, black = Marble Bar-Glacier Valley, pink = Orebody 19, 31, teal = Wheelara North, orange with thick outline = Harding Dam (maybe *ngarluma*, see text); Circles: dark blue = *Karaopsjulianneae*, dark green = *Karaopsbadgeradda*, red = *Karaopsjoehaeneri* sp. nov., teal = *Karaopskarrawarla*, blue = *Karaopsdurrantorum* sp. nov., brown = *Karaopsburbidgei*, green = *Karaopsmartamarta*, purple = *Karaopsnyangumarta*, black = *Karaopsbanyjima*, yellow = *Karaopsmorganoconnelli* sp. nov., gray = *Karaopsnyiyaparli*, red-brown = *Karaopsfeedtime*, bright pink = *Karaopsnyamal* (probably, see text), white = *Karaopskariyarra*, bright green = *Karaopsyurlburr*, orange = *Karaopsngarluma*, tan = *Karaopsjaburrara*, peach = *Karaopsforteyi*, teal = new species yet undescribed; small white diamonds are unidentifiable immatures for which no molecular data are available **B** boxed area expanded for clarity.

Fig. 48C, D , Maps 1 , 9A, B

Karaops burbidgei Crews & Harvey, 2011: 48, figs 35–28 (♂♀, examined).

##### Diagnosis.

The males can be differentiated from other members of the group by the large tegular lobe that covers nearly all of the basal part of the cymbium and the spermophor does not come into contact with the lower margin of the lobe (Crews and Harvey 2011: fig. 35). The female can be differentiated from other species by having short copulatory ducts, and the copulatory openings are located beneath an m-shaped hood in the center of the epigynal plate (Crews and Harvey 2011: figs 37, 38).

##### Description.

The description of the male and female can be found in Crews and Harvey (2011).

##### Distribution.

This species is known only from Barrow and Varanus Islands, Western Australia.

##### Natural history.

*Karaopsburbidgei* (Fig. 48C, D) is only known from Barrow Island and Varanus Island. The islands are primarily in the Carnarvon ecoregion in the Cape Range subregion. The south peninsula of Barrow Island is part of the Pilbara bioregion, Roebourne subregion, and no specimens have been collected here, but it is unclear if the area has been searched. Males and females have been collected in both the hotter, wetter season and the cooler, drier season. This species has been collected on or beneath rocks at night and in pitfall traps set around the rocks.

##### Discussion.

This species is morphologically similar and genetically closely related to *Karaopsdurrantorum* sp. nov. (Suppl. material 1). Barrow Island is a land-bridge island, and there have been several fluctuations of sea level connecting and disconnecting it from the mainland. The last time there was a connection to the mainland was 8–10 kya. Sea levels have also caused the island to be divided into smaller islands until ~ 2 kya (Moro and Lagdon 2013) (Suppl. material 2: table S1).

#### ﻿ Karaops martamarta

Taxon classificationAnimaliaAraneaeSelenopidae

Crews & Harvey, 2011

C595A45D-E057-5FCC-95F4-01F19B9633FE

**Figure 49.**
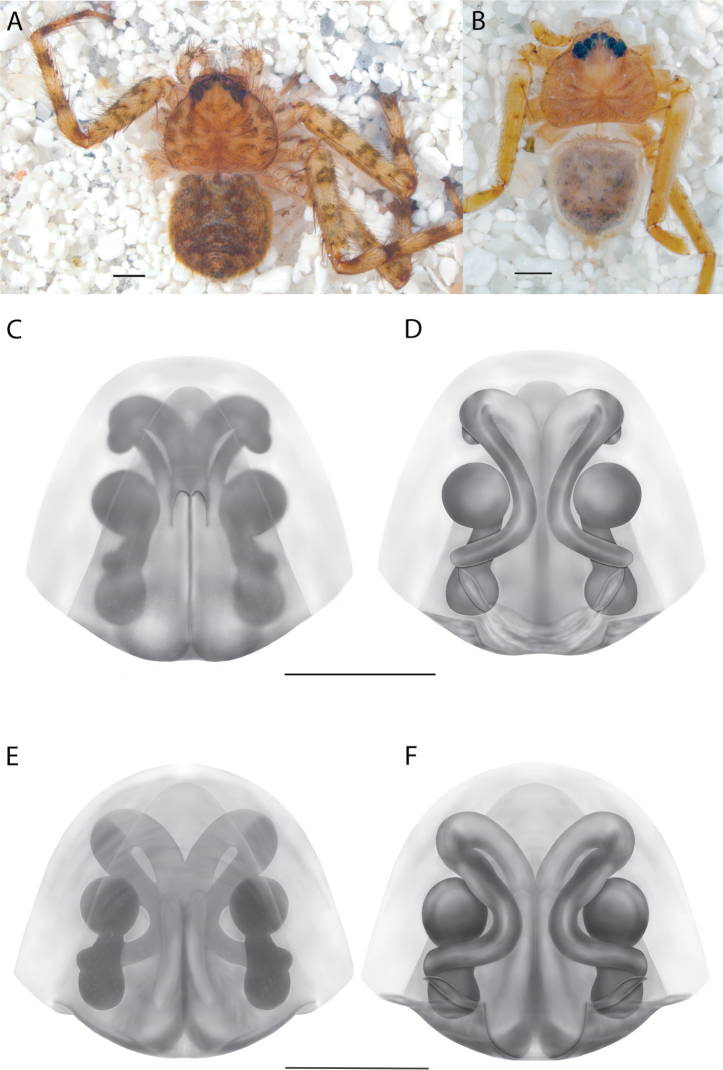
*Karaopsmartamarta* of the Pilbara-Gascoyne species group, Western Australia **A** female holotype, Trinity Bore South, vic. Cardo Camp, Red Hill (WAM T97482) **B** adult male (WAM T79413) **C** female holotype, epigyne, Trinity Bore South, vic. Cardo Camp, Red Hill (WAM T97482) **D** same, endogyne **E** epigyne, 19.8 km W Mt. Berry (WAM T94997) **F** same, endogyne. Scale bars: 0.5 mm (**C–F**); 1 mm (**A, B**).

**Figure 50.**
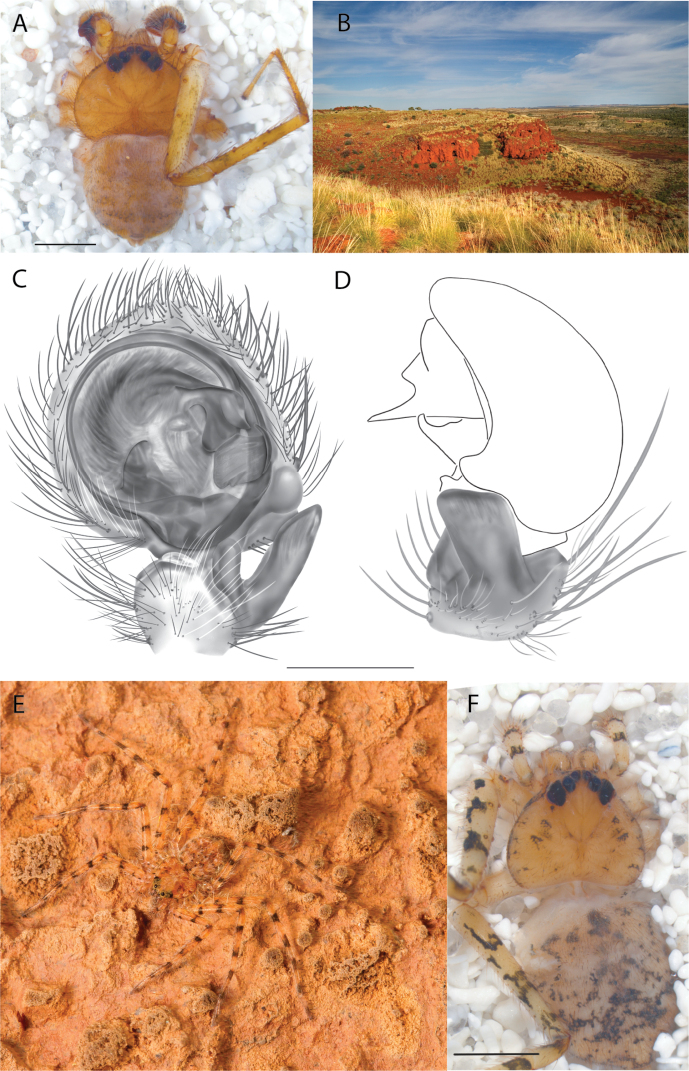
Members of the Pilbara-Gascoyne species group, Western Australia **A***Karaopsmartamarta*, adult male, 8 km NW of Mt. Berry (WAM T79413) **B** habitat of *Karaopsmartamarta*, Robe Valley **C***Karaopsmartamarta*, palp ventral, vic. Mt. Berry (WAM T79411) **D** same, retrolateral **E***Karaops* sp., Mundee, Mt. Augustus National Park (photo: J. DeJong) **F***Karaopsjulianneae*, adult female, Lorna Glen Station (WAM T107714). Scale bars: 0.5 mm (**C, D**); 1 mm (**A**); 2 mm (**F**).

Figs 49A–F , 50A–D , Maps 1 , 9A, B

Karaops martamarta Crews & Harvey, 2011: 56, figs 45–46, 93 (♀, examined); Crews 2013: 463, figs 29–30 (♂, examined).

##### New records.

Western Australia • 1 imm.; 42 km SSE of Pannawonica, site 1004-BUN01; 21°59'16.03"S, 119°07'39.52"E; 10 May 2012; col. staff from Phoenix Environmental leg.; foraging; (WAM T124900) • 1 imm.; 30–45 km S of Pannawonica, site 1004-Dragon; 21°55'41.54"S, 116°36'42.01"E; 6 Jul. 2012; no collector given; foraging; (WAM T124901) • 1♂; Mesa H, 12.5 km SW of Pannawonica; 21°42'47.59"S, 116°13'53.31"E; 5 May 2016; M. Love, J. Trainer leg.; railway culvert; hand collected; (WAM T141164) • 1♂; Mesa H, 19 km SW of Pannawonica; 21°46'30.84"S, 116°12'32.87"E; 7 Jun. 2016; C. Cole, N. Watson leg.; drainage/breakaway; leaf/soil sieving; (WAM T141165) • 1 imm.; Red Hill Station, ~ 10 km NE of homestead, Cochrane and Jewell bore site RNRC083; 21°55'55.25"S, 116°07'42.49"E; 13 May 2009; S. Crews leg.; (WAM T97474) • 1 imm.; Red Hill Station, ~ 8 km NE of homestead, Cochrane and Jewell bore site RNRC140; 21°56'11.40"S, 116°07'13.80"E; 13 May 2009; S. Crews leg.; (WAM T97475) • 1 imm.; Red Hill Station, ~ 20 km SE of homestead, Kens Bore site KBRC081; 22°03'26.83"S, 116°11'46.82"E; 13 May 2009; S. Crews leg.; (WAM T97476) • 1 imm.; Red Hill Station, ~ 22 km SE of homestead, Kens Bore site KBRC076; 22°04'51.10"S, 116°12'53.52"E; 13 May 2009; S. Crews leg.; (WAM T97477) • 1 imm.; Red Hill Station, ~ 27 km SE of homestead, Cardo Bore North, site CBRC099; 22°08'25.51"S, 116°13'48.22"E; 14 May 2009; S. Crews leg.; (WAM T97478) • 1 imm.; Red Hill Station, ~ 24 km SE of Cardo Outstation, Trinity Bore site TBRC031; 22°20'26.89"S, 116°20'20.28"E; 14 May 2009; S. Crews leg.; (WAM T97479) • 1 imm.; Red Hill Station, ~ 22 km SE of Cardo Outstation, Trinity Bore site TBRC119; 22°20'19.09"S, 116°19'22.42"E; 14 May 2009; S. Crews leg.; (WAM T97480) • 1 imm.; Red Hill Station, ~ 23 km SE of Cardo Outstation, Trinity Bore site TBRC151; 22°21'15.66"S, 116°19'33.42"E; 14 May 2009; S. Crews leg.; (WAM T97481) • 1♀; ~ 25 km SE of Cardo Outstation, Trinity Bore South site TBRC078; 22°23'54.89"S, 116°19'32.43"E; 14 May 2009; S. Crews leg.; (WAM T97482) • 1 imm.; ~ 23 km SE of Cardo Outstation, Trinity Bore South site TBRC062; 22°23'02.35"S, 116°18'12.65"E; 14 May 2009; S. Crews leg.; (WAM T97483) • 1 imm.; Red Hill Station, ~ 10 km NE of Cardo Outstation, Cardo Bore East site CBRC300; 22°11'57.67"S, 116°12'00.69"E; 15 May 2009; S. Crews leg.; (WAM T97485) • 1 imm.; Mesa G-Warramboo, 22.6 km WSW of Pannawonica; 21°44'25"S, 116°08'05"E; 25 Aug. 2009; M. Greenham leg.; (WAM T100066) • 1 imm.; Mesa G-Warramboo, 22.4 km WSW of Pannawonica; 21°44'15"S, 116°07'49"E; 26 Aug. 2009; M. Greenham leg.; (WAM T100067) • 1 imm.; Mesa G-Warramboo, 22.6 km WSW of Pannawonica; 21°44'25"S, 116°08'05"E; 25 Aug. 2009; M. Greenham leg.; (WAM T100068) • 1 imm.; same as previous except T. Sachse leg.; (WAM T100069) • 1 imm.; same as previous except M. Greenham leg.; (WAM T100070) • 1 imm.; Nammuldi-Silvergrass, 584 km NW of Tom Price; 22°23'58"S, 117°18'56"E; 15 Nov. 2009; M. Greenham leg.; (WAM T100075) • 1 imm.; ~ 8 km, 217° from Mt Delphine, site 999-D08; 22°16'51.06"S, 116°34'00.28"E; 28 Apr. 2012–20 Jun. 2012; P. Langlands leg.; wet pitfall trap; (WAM T124790) • 1 imm.; same as previous; (WAM T124791) • 1 imm.; ~ 24.5 km S of Mt. Delphine, site 999-D02; 22°26'42.09"S, 116°37'37.72"E; 19 Jun. 2012; P. Langlands leg.; foraging; (WAM T124796) • 1 imm.; ~ 18 km, 182° from Mt. Delphine, site 999-D04; 22°23'03.07"S, 116°36'30.67"E; 6 Jun. 2012; P. Langlands leg.; foraging; (WAM T124797) • 1 imm.; ~ 4 km, 299° from Mt. Farquhar, site 999-D12; 22°17'07.18"S, 116°44'07.57"E; 21 Jun. 2012; P. Langlands leg.; foraging; (WAM T124798) • 1 imm.; ~ 18 km SW of Pannawonica; 21°46'20.73"S, 116°12'15.24"E; 7 May 2012; D. Kamien, M. Greenham, D. Keirle leg.; foraging; (WAM T136475) • 1 imm.; ~ 13.3 km ENE of Mt. Farquhar, site 999-F5; 22°16'58.50"S, 116°53'32.76"E; 22 Jun. 2012; P. Langlands leg.; foraging; (WAM T124800) • 1 imm.; ~ 8 km, 120° from Mt. Delphine, site 999-D10; 22°15'34.21"S, 116°40'50.24"E; 21 Jun. 2012; P. Langlands leg.; foraging; (WAM T124801) • 1 imm.; 20 km WSW. of Mt. Brockman, site 999-E1; 22°31'11.92"S, 117°07'17.83"E; 24 Jun. 2012; P. Langlands leg.; foraging; (WAM T124802) • 1 imm.; 7.5 km WSW of Mt. Brockman, site 999-E9; 22°28'53.47"S, 117°14'18.22"E; 24 Jun. 2012; P. Langlands leg.; foraging; (WAM T124803) • 1 imm.; ~ 14 km, 169° from Mt. Delphine, site 999-D05; 22°20'47.74"S, 116°38'25.48"E; 28 Apr.–20 Jun. 2012; P. Langlands leg.; wet pitfall trap; (WAM T124804) • 1 imm.; ~ 18 km, 182° from Mt. Delphine, site 999-D04; 22°23'03.07"S, 116°36'30.67"E; 27 May 2012; P. Langlands leg.; foraging; (WAM T124805) • 1 imm.; ~ 8 km, 217° from Mt. Delphine, site 999-D08; 22°16'51.06"S, 116°34'00.28"E; 28 Apr. 2012; P. Langlands leg.; foraging; (WAM T124806) • 1 imm.; 6.5 km WSW of Mt. Brockman, site 999-E7; 22°28'31.05"S, 117°14'34.95"E; 3 May 2012; P. Langlands leg.; foraging; (WAM T124807) • 1 imm.; ~ 21 km, 167° from Mt. Farquhar, site 999-E5; 22°28'53.67"S, 116°48'38.79"E; 2 May 2012; P. Langlands leg.; foraging; (WAM T124808) • 1 imm.; 7.5 km WSW of Mt. Brockman, site 999-E9; 22°28'53.47"S, 117°14'18.22"E; 3 May 2012; P. Langlands leg.; foraging; (WAM T124809) • 1 imm.; 17 km WSW of Mt. Brockman, site 999-E3; 22°32'03.03"S, 117°09'37.90"E; 23 Jun. 2012; P. Langlands leg.; foraging; (WAM T124810) • 1 imm.; ~ 19 km, 188° from Mt. Delphine, site 999-D03; 22°23'44.62"S, 116°35'07.95"E; 19 Jun. 2012; P. Langlands leg.; foraging; (WAM T124814) • 1 imm.; 42 km SSE of Pannawonica, site 1004-BUN06; 21°59'16.03"S, 116°29'51.31"E; 9 May 2012 leg.; foraging; (WAM T124903) • 1 imm.; Cane River Conservation Park, N end of Parry Range, site CR02; 22°07'03.9"S, 115°34'10.3"E; 21 Jun. 2011; J.M. Waldock leg.; by hand; under rock; (WAM T125619) • 1 imm.; ~ 6 km SE of Pannawonica; 21°39'39.35"S, 116°22'14.48"E; 10 Sep. 2012; S. White leg.; vert trap; leaf litter; (WAM T126894) • 1 imm.; ~ 100 km W of Tom Price; 22°33'44.25"S, 116°43'13.04"E; 21 Apr. 2013; A. Leung leg.; foraging; (WAM T128012) • same as previous; footslope, gully base; (WAM T128013) • 1 imm.; ~ 100 km W of Tom Price; 22°32'17.40"S, 116°52'11.71"E; 19 Apr. 2013; A. Leung leg.; foraging; (WAM T128014) • 1 imm.; ~ 100 km W of Tom Price; 22°31'33.18"S, 116°48'02.55"E; 19 Apr. 2013; A. Leung leg.; foraging; gorge sides and base; (WAM T128015) • same as previous (WAM T128016) • 1♀; ~ 100 km W of Tom Price; 22°22'42.91"S, 116°46'13.83"E; 18 Apr. 2013; A. Leung leg.; foraging; (WAM T128017) • 2 imm.; ~ 100 km W of Tom Price; 22°32'20.37"S, 116°43'29.91"E; 16 Apr. 2013; A. Leung leg.; foraging; (WAM T128018) • 1 imm.; ~ 100 km W of Tom Price; 22°35'39.83"S, 117°01'34.19"E; 16 Apr. 2013; A. Leung leg.; foraging; (WAM T128019) • same as previous; foraging; gully sides; (WAM T128020) • 1 imm.; ~ 100 km W of Tom Price; 22°42'31.24"S, 116°51'00.35"E; 17 Apr. 2013; A. Leung leg.; foraging; (WAM T128021) • 1 imm.; ~ 130 km WNW of Tom Price; 22°18'26.37"S, 116°48'20.37"E; 15 Apr. 2013; A. Leung leg.; foraging; (WAM T128022) • 1 imm.; ~ 90 km WNW of Tom Price; 22°29'06.48"S, 116°46'42.67"E; 15 Apr. 2013; A. Leung leg.; foraging; (WAM T128023) • 1 imm.; ~ 130 km WNW of Tom Price; 22°18'29.14"S, 116°26'02.09"E; 16 Apr. 2013; A. Leung leg.; foraging; (WAM T128024) • same as previous; footslope, gorge; (WAM T128025) • 1 imm.; ~ 90 km WNW of Tom Price; 22°29'06.49"S, 116°46'42.68"E; 15 Apr. 2013; A. Leung leg.; foraging; (WAM T128026) • 1 imm.; ~ 23 km SW of Pannawonica; 21°46'46.99"S, 116°08'46.00"E; 15 May 2013; N. Dight leg.; foraging; (WAM T128055) • 1 imm.; ~ 100 km W of Tom Price; 22°35'39.83"S, 117°01'34.19"E; 30 May 2013; A. Leung leg.; foraging; (WAM T128148) • 1 imm.; ~ 100 km W of Tom Price; 22°35'39.83"S, 117°01'34.19"E; 16 Apr.–30 May 2013; A. Leung leg.; wet pit (eth. glyc); (WAM T128149) • same as previous; (WAM T128155) • same as previous; (WAM T128156) • same as previous; (WAM T128157) • same as previous; (WAM T128163) • same as previous; (WAM T128165) • 1 imm.; ~ 100 km W of Tom Price; 22°28'55.59"S, 116°31'10.13"E; 20 Apr. 2013–30 May 2013; A. Leung leg.; wet pitfall trap; (WAM T128173) • 1 imm.; ~ 22 km, S from Pannawonica; 21°50'22.98"S, 116°17'41.94"E; 12–19 May 2014; N. Dight leg.; foraging; (WAM T128899) • 1 imm.; ~ 22 km, S from Pannawonica; 21°50'13.98"S, 116°17'28.40"E; 12–19 May 2014; N. Dight leg.; wet pitfall (prop.); (WAM T128900) • 1 imm.; Red Hill Creek, 90.3 km S of Pannawonica; 22°27'17"S, 116°17'06"E; 24 May 2015; R. Teale, C. Cole leg.; under rocks; (WAM T137101) • 1 imm.; Red Hill Creek, 50.4 km S of Pannawonica; 22°05'35"S, 116°18'17"E; 21 May 2015; D. Kamien, C. Cole leg.; under rocks; (WAM T137102) • 1 imm.; ~ 50 km W of Pannawonica, SRE-M04, Mesa B C (West Robe); 21°42'01.9"S, 115°57'58.0"E; 7 Aug. 2015; F. Leng leg.; targeted searches; mesa; (WAM T137136) • 1 imm.; ~ 45 km NW Newman, Mindy South; -22.93, 119.40; 29 Apr. 2022; S. Ronan leg.; invertebrate pitfall trap; (N23137-1); • 1 imm.; ~ 45 km NW Newman, Mindy South; -22.97, 119.41; 29 Apr. 2022; S. Ronan leg.; invertebrate pitfall trap; (N23137-1).

##### Diagnosis.

The female (Fig. 49A, B) is most similar to *Karaopskarrawarla* by the m-shaped depression where the copulatory openings are located; however, in this species, the lateral lobes do not come into contact posteriorly and in *K.martamarta*, the copulatory ducts nearly come into contact where they curve from anterior to posterior, whereas the curve is much broader in *K.karrawarla* (Figs 49C–F, 55D, E). The male (Fig. 50A) has an extremely short palpal tibia and a very prominent, knobby retrobasal cymbial process (Fig. 50C, D).

##### Description.

The description of the female can be found in Crews and Harvey (2011), and the description of the male can be found in Crews (2013).

##### Distribution.

This species is found in the Pilbara, Western Australia.

##### Natural history.

The Hamersley subregion is located in the Pilbara bioregion. It is mountainous with many gorges and with low mulga woodland, bunch grasses, hummock-forming grasses, and snappy gum (Kendrick 2001a). The climate is semi-desert tropical, and it usually rains in the summer, although sometimes rain also occurs in winter. This differs somewhat from the Gascoyne to the south, as it is a desert climate with summer and winter rainfall. In the Hamersley subregion, rainfall is highest December to March and lowest August to November; temperatures are highest during the wetter months and drier in the cooler months.

No collections of any life stage of *Karaopsmartamarta* have been made during the wet, hot season of December to March; however, this likely reflects collecting efforts as summer rainfall can make travel difficult, and the heat can be oppressive, although many other species of *Karaops* in the Chichester subregion have been collected during this time (see Suppl. material). All life stages have been collected April to June, no adults in July, a dry cooler time, and no adults in November. This could also represent collecting efforts as the immatures represent different instars. It appears that *K.martamarta* is one of the most commonly collected *Karaops*; however, again, this likely reflects collecting efforts, as surveys in the Pilbara occur before the onset of major mining operations, and the collection localities of this species reflect that (Suppl. material 2: table S1). Additionally, there have been collecting efforts that have specifically targeted this species. *Karaopsmartamarta* has been collected by hand under rocks and in a railway culvert, by sifting leaves and soil from a drainage/breakaway, leaf litter in a vertebrate trap and pitfall traps in gorges, on mesas, and in a footslope gully base and sides.

##### Discussion.

The Hamersley subregion (Fig. 50B) is the southern part of the Pilbara Craton, a geologically stable region that is one of two places on earth with Archaean crust. There is high species and ecosystem diversity, and the region is known for many SREs (Huey, Hillyer and Harvey 2019). There are more species of *Karaops* in the Pilbara bioregion than any other bioregion.

The genitalia of the holotype female (WAM T97482) (Fig. 49A, C, D) and a male (WAM T79143) (Fig. 50A, C, D) are illustrated for ease of comparison with similar species. The genitalia of another female (WAM T94997) (Fig. 49E, F) is also illustrated to show variation from the holotype. Measurements were made of some additional males—total length variation: 5.08–6.25. *Karaopsmartamarta* and a species known only from immature specimens have primarily been collected in the Hamersley subregion of the Pilbara bioregion (Fig. 50B), with the former having a few collections in the Chichester subregion to the north. *Karaopsbanyjima* has also only been found in this subregion, as well as *K.nyangumarta*, *K.morganoconnelli* sp. nov., and a few species known only from immature samples with a few collections made just at the border of the Chichester bioregion (Map 9A). This could reflect a lack of collecting efforts in the surrounding areas to the south, although that is part of the Gascoyne bioregion and is likely to harbor different taxa. Recently, the species was found further to the east, quite far from other collections, expanding the range. There is little overlap between species, but some are very close and must come into contact; however, multiple species have not been collected in the same place at the same time even though adults have temporal overlap, and immatures can be found throughout most of the year. This pattern is very similar to that of Kimberley species group members (Map 7).

Molecular data show that the Pilbara/Gascoyne clade is separated into two clades that do not completely reflect geography, e.g., there is no Gascoyne clade or a Pilbara clade (Suppl. material 1). All of the Gascoyne species are in the same clade, but this clade also contains *Karaopsmartamarta*, *K.morganoconnelli* sp. nov., and two undescribed species known from juveniles: one from Nammuldi/Tom Price and the other Wheelara. Members of this clade all have very similar genitalia. The species in the other clade mostly comprise those found in the Pilbara, but there is possibly a juvenile undescribed species from Mt. Augustus that is recovered within this clade.

#### ﻿ Karaops julianneae

Taxon classificationAnimaliaAraneaeSelenopidae

Crews & Harvey, 2011

5E286B2C-5804-5765-820D-E91F1A8CC8F2

**Figure 51.**
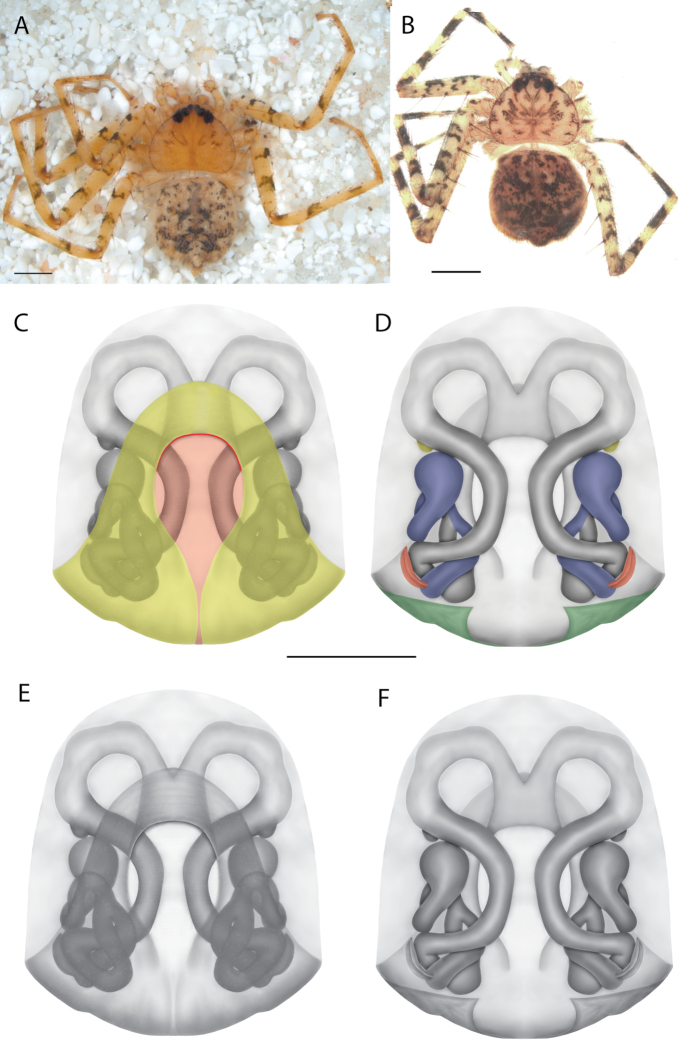
*Karaopsjulianneae* and *Karaopsbadgeradda* of the Pilbara-Gascoyne species group, Western Australia **A***Karaopsjulianneae*, holotype female, Lorna Glen Station (WAM T64748) **B***Karaopsbadgeradda*, adult female, Badgeradda Range (WAM T97213) **C***Karaopsjulianneae*, holotype female, epigyne, Lorna Glen Station; red = copulatory opening, pink = median field, yellow = lateral lobes **D** same, endogyne; blue = spermathecae, yellow = accessory bulbs, red = fertilization ducts, green = posterodorsal fold **E** same, epigyne **F** same, endogyne. Scale bars: 0.5 mm (**C–F**); 2 mm (**A, B**).

Figs 50F , 51A, C–F , Maps 1 , 9A

Karaops julianneae Crews & Harvey, 2011: 53, figs 43, 44 (♀, examined).

##### Diagnosis.

This species can be differentiated from the other members of the Pilbara/Gascoyne species group by the genitalia. There is one copulatory opening, and the copulatory ducts aren’t fully separated at the opening, then are split into two separate ducts. The ducts that connect the accessory bulb and the spermathecae are much longer than in any of the other species (Fig. 51E, F).

##### Description.

The description of the female can be found in Crews and Harvey (2011).

**Male.** Unknown.

##### Distribution.

This species is known only from the type locality, Lorna Glen Station in the Gascoyne Region, Western Australia (Map 9A).

##### Natural history.

This species remains only known from a few specimens from the type locality, located in the Gascoyne bioregion, Carnegie subregion. This area has a desert climate, hummock grassland and shrub steppe, and succulent steppe/low woodland with mulga (Cowan 2001; Bastin et al. 2008). It is known for several rare animal species and is a center of endemism for a gecko and a skink. It is coolest in the drier months and warmer in the wetter months. Rainfall begins to increase in December, is highest January–March, and begins to decrease in April, with the lowest amount occurring from August–October. Adult females and immatures have been collected in November (dry, cooler, beginning to get wetter/warmer) and April (beginning to get drier/cooler). Only an immature was collected in March (wettest/warmest); however, it is likely females were around as they were collected the previous month (Suppl. material 2: table S1).

##### Discussion.

The epigyne and endogyne of the holotype (WAM T64748) have been re-figured for easier comparison with other group members (Fig. 51C–F). Additionally, images of the holotype and another specimen (WAM T107714) are provided (Figs 50F, 51A). There is no variation in the genitalia or habitus of the two specimens. Total length variation: 6.54–6.77.

#### ﻿ Karaops badgeradda

Taxon classificationAnimaliaAraneaeSelenopidae

Crews & Harvey, 2011

6474DDC5-11CA-572B-9A7E-45C885D89F19

**Figure 52.**
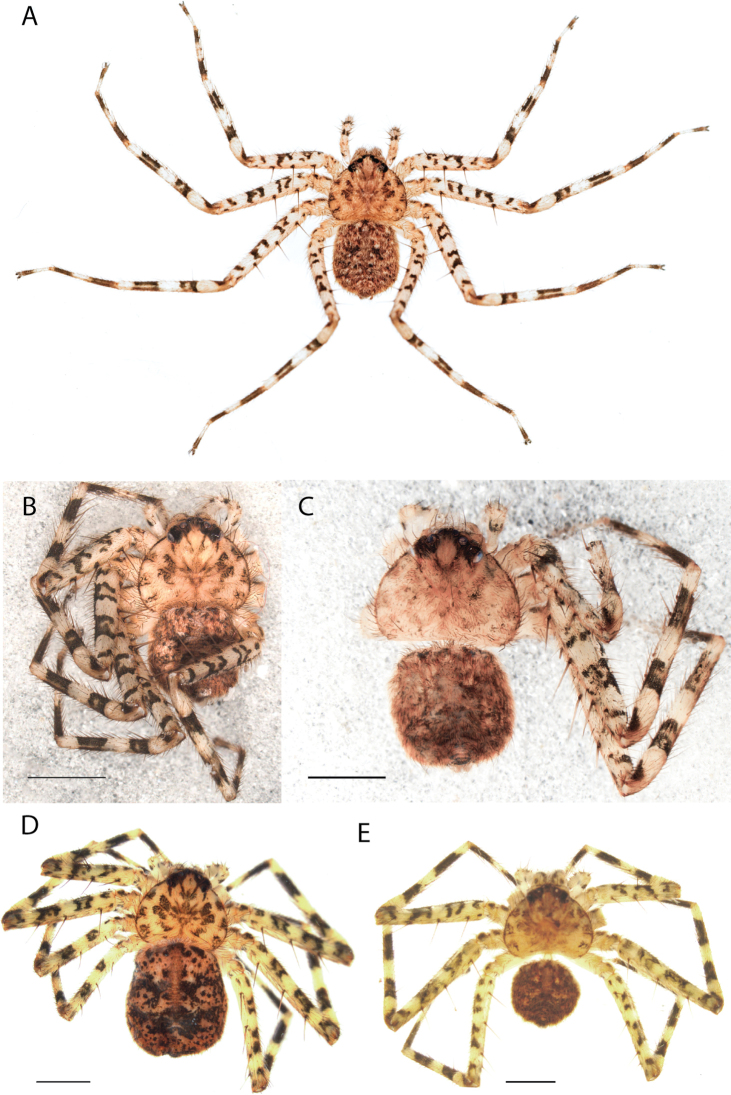
*Karaopsbadgeradda*, Badgeradda Range, Western Australia **A** adult female (sel_1118, T155494) **B** same (sel_1127, WAM T155503) **C** same (sel_1118, WAM T155494) **D** holotype female (WAM T97214) **E** adult female (WAM T97215) **E** same (WAM T97213). Scale bars: 2 mm.

**Figure 53.**
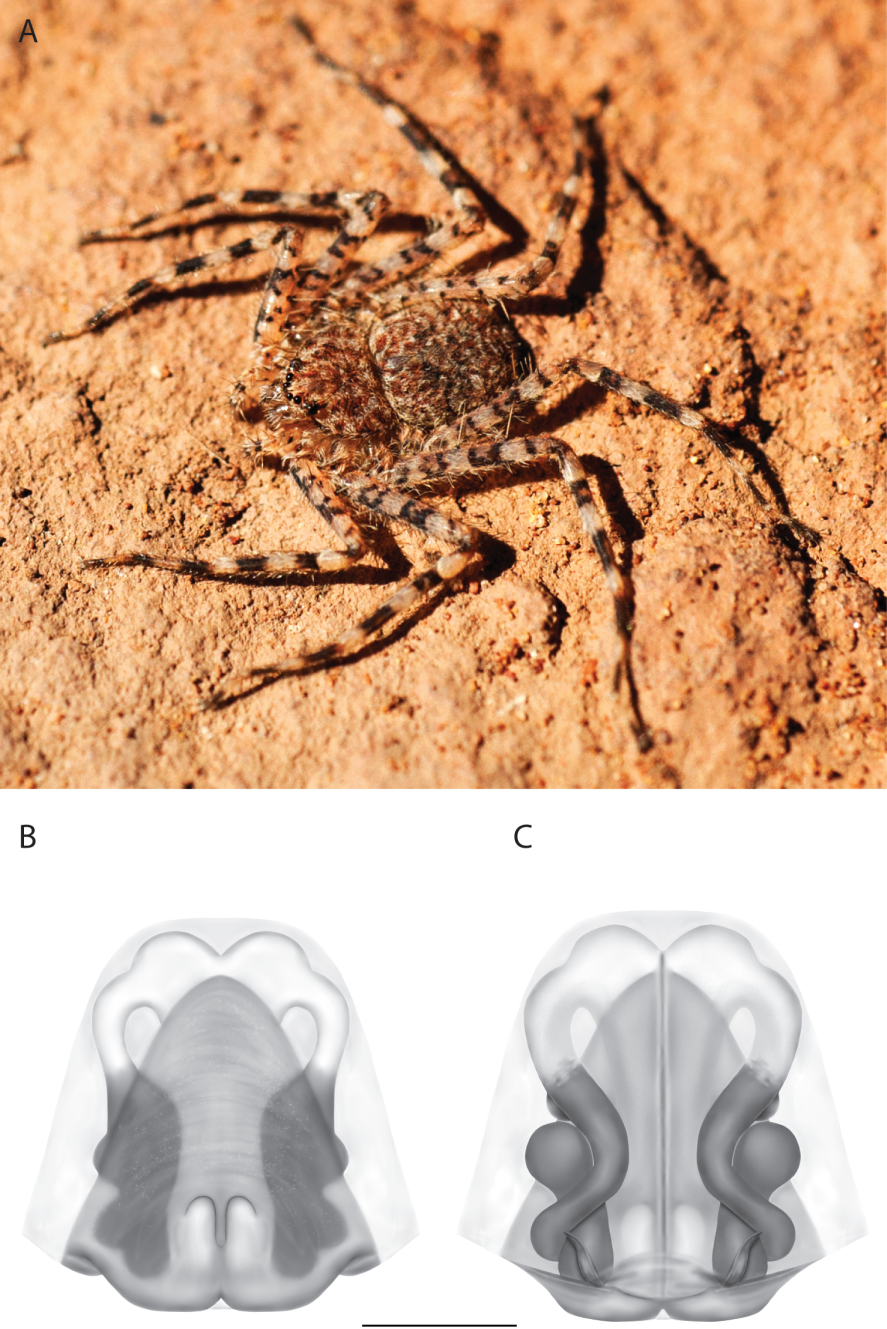
*Karaopsbadgeradda*, Badgeradda Range, Western Australia **A** adult female **B** epigyne (sel_1118, WAM T155494) **C** endogyne. Scale bar: 0.5 mm.

**Figure 54.**
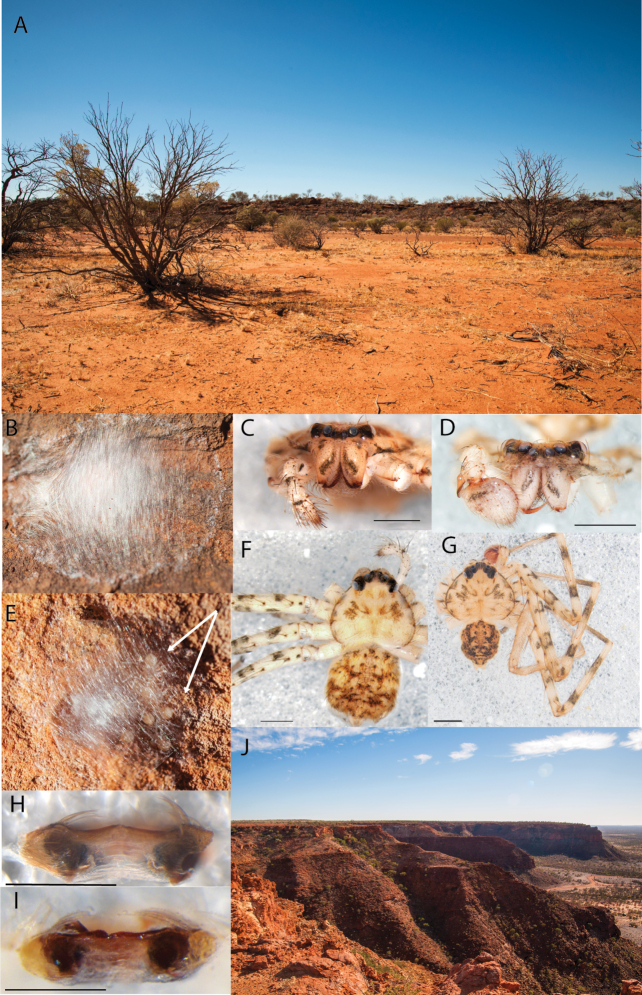
Members and habitats of the Pilbara-Gascoyne species group, Western Australia **A** Badgeradda Range, habitat of *Karaopsbadgeradda***B***Karaopsbadgeradda* egg sac, under rock, Badgeradda Range **C***Karaopsjoehaeneri* sp. nov., holotype female, Escarpment Trail, Kennedy Range National Park (sel_1130, WAM T155506) **D** same, paratype male (sel_1131, WAM T155507) **E** same, egg sac with spiderlings, arrows indicate spiderlings **F** same, holotype female, Escarpment Trail, Kennedy Range National Park (sel_1130, WAM T155506) **G** same, paratype male (sel_1134, WAM T155510) **H***Karaopsbadgeradda*, epigyne, caudal view (sel_1118, WAM T155494) **I***Karaopsjoehaeneri* sp. nov., holotype female, epigyne, caudal view (sel_1130, WAM T155506) **J** habitat of *Karaopsjoehaeneri* sp. nov., Kennedy Range National Park.

Figs 51B , 52A–E , 53A–C , 54A, B, H , Maps 1 , 9A

Karaops badgeradda Crews & Harvey, 2011: 46, figs 33, 34 (♀, examined).

##### New records.

Western Australia • 3♀, 9 imm; Badgeradda Range, Muggon Station Road off Butcher’s Track; 26°46.220'S, 115°32.927'E; ~ 260 m; 8 May 2016; col. S. Crews, J. De Jong leg.; under rocks along range; sel_1118–1129; SCC16_017; (WAM T155494–155505).

##### Diagnosis.

*Karaopsbadgeradda* is similar to other members of the species group but can easily be distinguished by the genitalia. The copulatory openings are closer to the posterior margin of the epigyne, and the lateral lobes cannot be distinguished on the epigyne (Fig. 53B, C).

##### Description.

The description of the female can be found in Crews and Harvey (2011).

**Male.** Unknown.

##### Distribution.

This species is known only from the type locality in the Gascoyne Region, Badgeradda Range, Western Australia (Map 9A).

##### Natural history.

*Karaopsbadgeradda* (Figs 51B, 52A–E, 53A) remains known only from the type locality (Fig. 54A), which is located in the Murchison bioregion, subregion West Murchison. This region consists of mulga low woodlands on hardpan washplains and hummock grassland. The headwaters of both the Murchison and Wooramel Rivers are in the subregion. It has been highly altered by pastoral use over the years. There is bimodal rainfall that usually occurs in winter, May–July. January–March also sees rainfall, with less in April and August, and very little September–December. Adult females have been found in March, when it is warmer and wetter than other times of year. Females and egg sacs (Figs 53A, 54B) have been found in March and May, when it is cooler and drier. The female guards the egg sac as in many selenopid species. The egg sac comprises thin silk, such as that of *K.joehaeneri* sp. nov. (Fig. 54E), and the eggs and spiderlings are easily visible, rather than the papery egg sac of some other species, such as *K.strayamate* sp. nov. (Fig. 6H). In captivity, immatures are found throughout the year. Males or penultimate males have never been found. Despite collecting many specimens at this locality, none were able to be reared adulthood. Males remain unknown despite pitfall traps remaining for quite a long time (Suppl. material 2: tables S1, S15). The spiders have been collected in pitfall traps and under stones on the range.

##### Discussion.

The genitalia of a newly collected specimen (sel_1118, WAM T155494) (Figs 51E, F, 53B, C, 54H, I) has been illustrated for ease of comparison with similar species. Measurements of additional specimens have been made, and lengths range from 5.36–7.45.

#### ﻿ Karaops joehaeneri sp. nov.

Taxon classificationAnimaliaAraneaeSelenopidae

E2BD63C4-34D8-585C-9A9B-15C8D6B5CE11

https://zoobank.org/D7F58579-0DBE-4D2F-A942-23253DF94536

**Figure 55.**
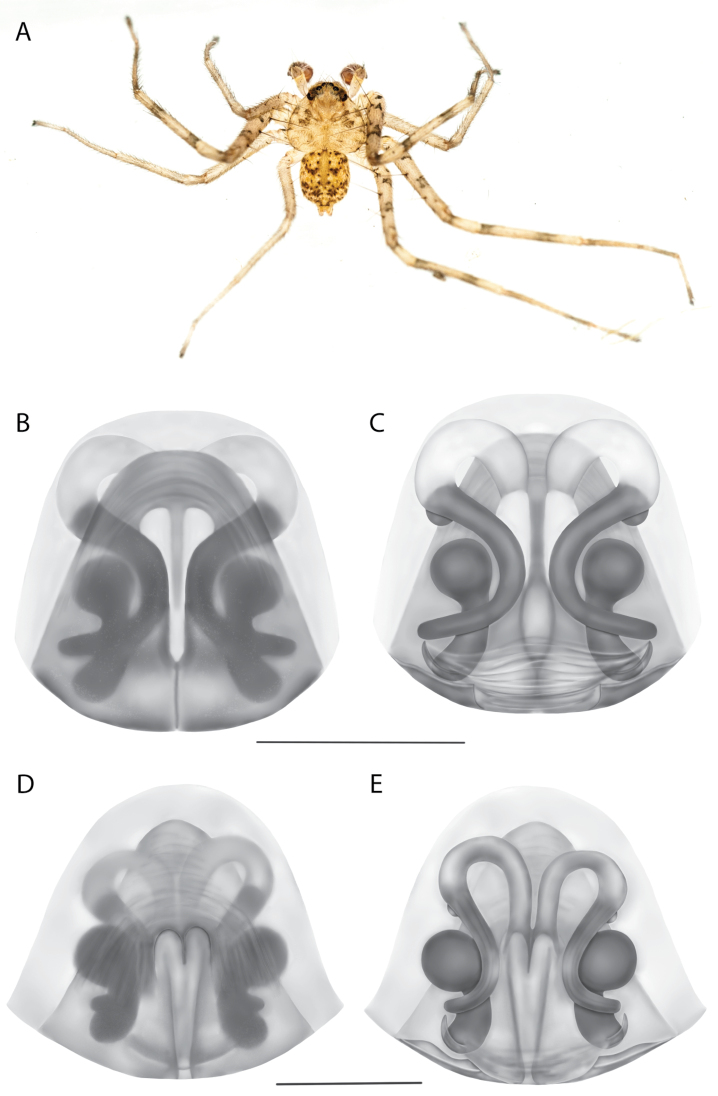
Members of the Pilbara-Gascoyne species group, Western Australia **A***Karaopsjoehaeneri* sp. nov., paratype male, Escarpment Trail, Kennedy Range National Park (sel_1131, WAM T155507) **B** same, holotype female, epigyne (sel_1130 WAM T155506) **C** same, endogyne **D***Karaopskarrawarla*, female paratype, epigyne, Bush Bay, Western Australia (WAM T76700) **E** same, endogyne. Scale bars: 0.5 mm.

**Figure 56.**
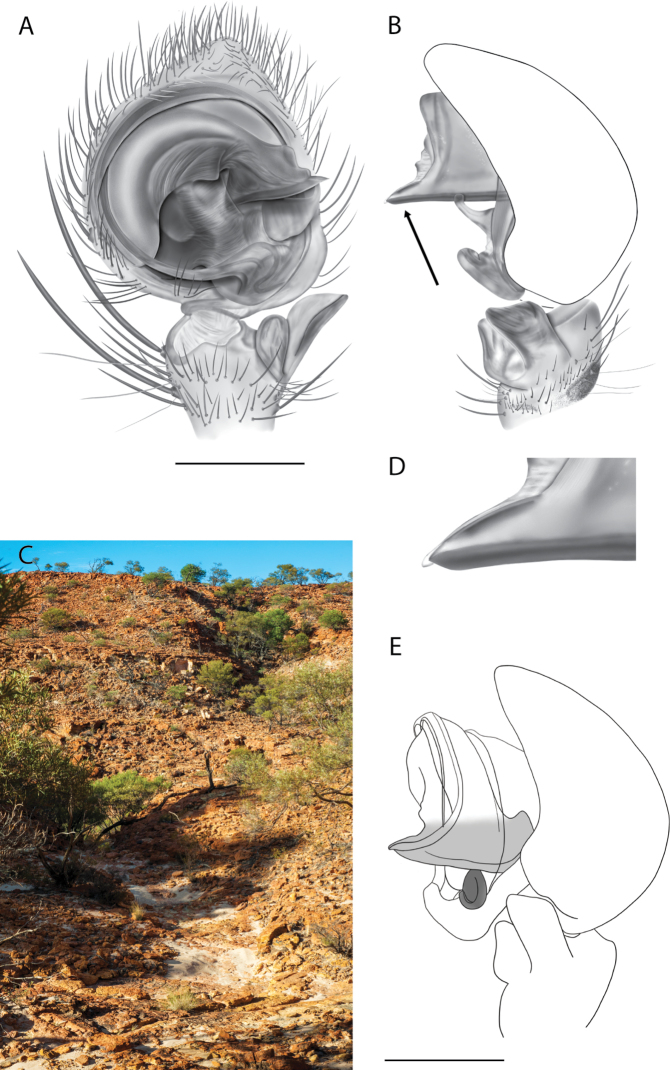
*Karaopsjoehaeneri* sp. nov., Escarpment Trail, Kennedy Range National Park, Western Australia **A** paratype male, palp, ventral (sel_1131, WAM T155507) **B** same, retrolateral, arrow indicates close-up in D, below **C** habitat of *Karaopsjoehaeneri* sp. nov., Kennedy Range National Park **D** paratype male, close up of the tip of the conductor as indicated by arrow in B, above **E** same, palp expanded, retrolateral, light gray = tip of conductor, dark gray = median apophysis. Scale bars: 0.5 mm.

**Figure 57.**
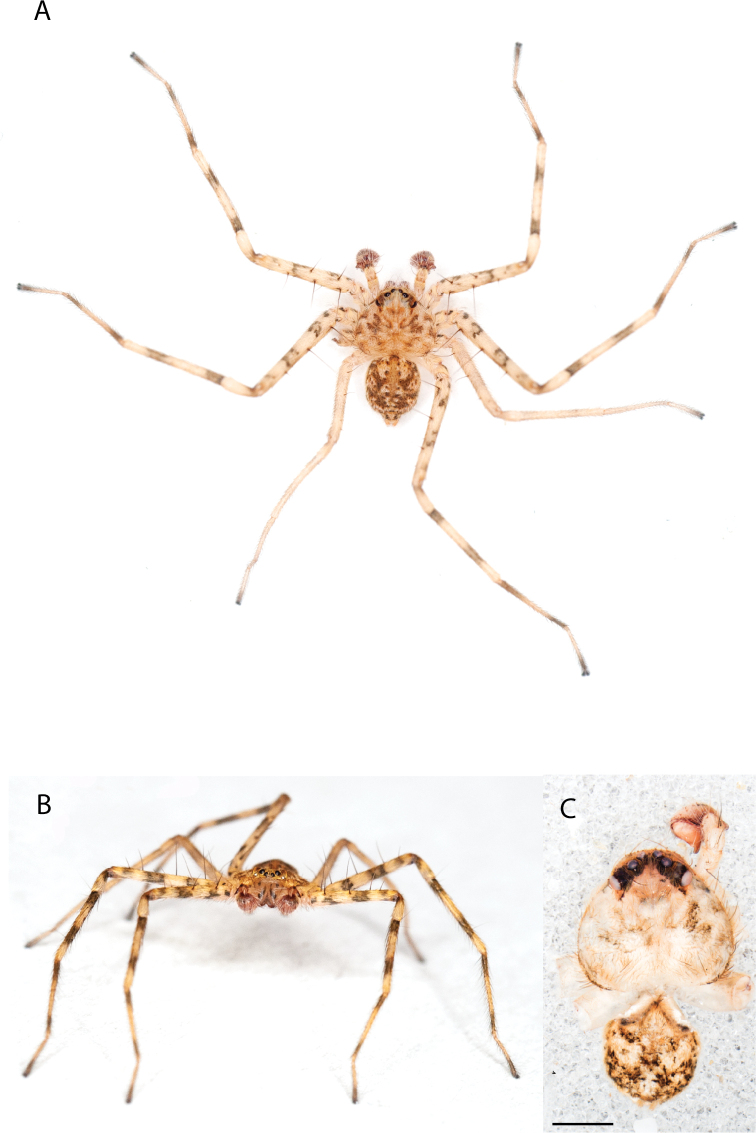
*Karaopsjoehaeneri* sp. nov., paratype male, Escarpment Trail, Kennedy Range National Park, Western Australia (sel_1131, WAM T155507) **A** adult male **B** same **C** same. Scale bar: 1 mm.

Figs 54C–G, I, J , 55A–C , 56A, B, D, E , 57A–C , Maps 1 , 9A

##### Material examined.

***Holotype***: Western Australia • ♀; Kennedy Range National Park, along Escarpment Trail; 24°39.909'S, 115°10.178'E; ~ 322 m; 9 May 2016; S. Crews, J. DeJong leg.; under rocks; sel_1130; SCC16_018; (WAM T155506) ***Paratypes***: 2♂; same data as for holotype; sel_1131, 1134; (WAM T155507, T155510) **Other material examined**: 1 imm., 1 penultimate ♀; same data as previous; sel_1132–1133; (WAM T155508–155509).

##### Diagnosis.

The female of *Karaopsjoehaeneri* sp. nov. is similar to other members of the group (*K.julianneae*, *K.karrawarla*, *K.martamarta*, *K.nyamal*, *K.badgeradda*, *K.morganoconnelli* sp. nov.) in that there are two copulatory openings, close together, in the middle of the epigynal plate, connected to separate copulatory ducts (Fig. 55B). In these species, the copulatory ducts are unsclerotized from their origin, becoming sclerotized to varying degrees depending on the species. They extend anteriorly, typically beyond the more sclerotized part of the plate, curve posterodorsally, with the small accessory bulb near the curve, come together medially, then extend laterally, wrapping around variously sized spermathecae which have an anterior bulb and may be shaped like a dumbbell or a light bulb. The new species differs from the others in that the copulatory openings have no hoods, and they are anterior of the accessory bulbs, nearer the level that the copulatory ducts curve in toward one another (Fig. 55B, C).

**Figure 58.**
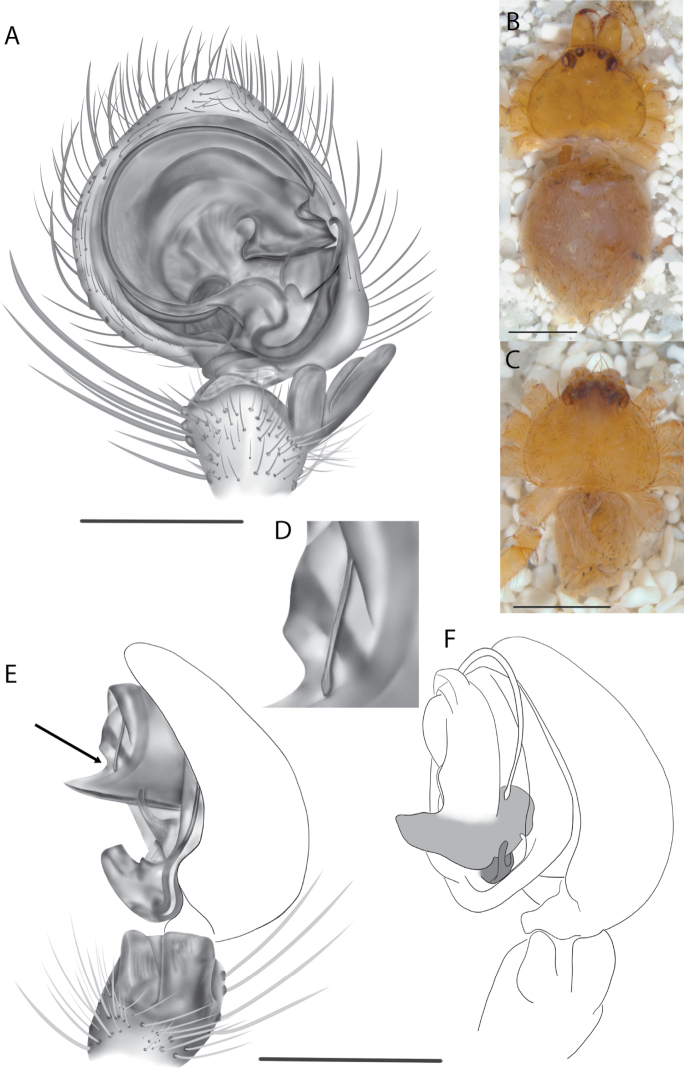
*Karaopskarrawarla*, Bush Bay, Western Australia **A** male holotype, palp, ventral (WAM T55001) **B** female paratype (WAM T76700) **C** male holotype (WAM T55001) **D** close up of embolus tip as indicated by arrow in E, below **E** palp, retrolateral **F** palp, expanded, light gray = tip of conductor, dark gray = median apophysis. Scale bars: 0.5 mm (**A, E, F**); 2 mm (**B, C**).

The male is similar to several species found in the species group (*K.karrawarla*, *K.martamarta*, *K.morganoconnelli* sp. nov.) by the large conductor that extends dorsally and ventrally (Fig. 56A, B, E). It can be differentiated from *K.morganoconnelli* sp. nov. by the wider cymbium and smaller tegular lobe (Fig. 60A, B, E) and from *K.martamarta* (Fig. 50C, D) by the smaller, more ventrally projecting tegular lobe, the small retrobasal cymbial process, and the smaller RTA and longer tibia. It can be separated from *K.karrawarla* by the differently shaped conductor and the smaller tegular lobe (Fig. 58A, E, F). Differences in the males of *K.joehaeneri* sp. nov., *K.morganoconnelli* sp. nov., and *K.karrawarla* are most easily seen in the conductor and median apophysis when the palp is expanded (Figs 56E, 58F, 60E).

##### Description.

**Female** (holotype). Total length 4.72. Carapace: length 2.33, width 2.73. Chelicerae: promargin with three teeth, retromargin with two teeth (1-0-1). Eyes: AER recurved; PER strongly recurved; diameters AME 0.13, ALE 0.10, PME 0.18, PLE 0.24; interdistances AME–PME 0.04, PME-PLE 0.15, ALE–PLE 0.19, PME–PME 0.77, ALE–ALE 1.16, AME–AME 0.38, PLE–PLE 1.47. Sternum: length 1.27, width 1.50. Abdomen: length 2.39, width 2.32. Color (in life/preserved Fig. 54F): Carapace: yellowish, with a pair of dark areas lateromedially and three pairs of dark areas laterally, with reddish setae. Chelicerae: yellowish brown, more reddish brown toward fang, paturon with curved, dark marks frontally and a small dark spot just below the clypeus (Fig. 54C), setae paler laterally, darker anteriorly. Maxillae: pale yellowish white. Labium: pale yellowish white. Sternum: yellowish white. Abdomen: dorsally reddish orange, with two small, u-shaped dark marks between medial and lateral areas, one small u-shaped mark medially, larger dark marks extending from medial to lateral, two dark marks posterior to those, with several dots across the abdomen, with red and brown setae, brown setae sparse, but thicker and conspicuously long; ventrally yellowish white. Spinnerets: with dark marks laterally. Legs: yellowish white, Cx with dusky mark prolaterally, Tr with dark line prolaterally and retrolaterally, spots at Tr-Fm joint, dark mark prolaterally on Fm, wrapping around but not completely enclosed, annulation at Fm-Pt joint, darker around edges, dusky mark basally on Pt, Ti, Mt each with two annulations, one basal, one distal, Ta dusky at tip; spination leg I Fm pl 1-1-1, d 1-1-1, Ti v 2-2-2-2-2, Mt v 2-2-2-2; leg II Fm d 1-1-1, Ti v 2-2-2-2-2, Mt v 2-2-2-2; leg III F d 1-1-1; leg IV F d 1-1-1; leg formula 3241; measurements leg I 9.71 (2.86, 1.07, 2.5, 2.14, 1.14); leg II 11.10 (3.32, 1.21, 3, 2.29, 1.32); leg III 11.40 (3.57, 0.86, 2.86, 2.71, 1.36); leg IV 10.60 (3.36, 0.89, 2.57, 2.57, 1.25). Palp: spination Fm 0-1-3; 2.65 (0.77, 0.36, 0.61, 0.75); claw with six teeth. Epigyne: EP longer than wide, slightly plicate; MF keyhole shaped; LLs mostly separated but touching posteriorly; COs located anteriorly. Endogyne: CDs wide, unsclerotized, extend anterolaterally beyond more sclerotized part of EP, narrowing, curved posteromedially and dorsally, more sclerotization at this juncture, curved posterolaterally, wrapped around S; ABs small, located where CDs become more sclerotized; S allantoid, but large, round; FDs directed anterolaterally; small pdf laterally (Figs 54I, 55B, C).

**Male** (paratype, sel_1131). Total length 4.18. Carapace: length 2.42, width 2.69. Chelicerae: promargin with three teeth, retromargin with two teeth. Eyes: AER recurved, PER strongly recurved; diameters AME 0.16, ALE 0.06, PME 0.22, PLE 0.27; interdistances AME–PME 0.02, PME–ALE 0.12, ALE–PLE 0.16, PME–PME 0.78, ALE–ALE 1.12, AME–AME 0.43, PLE–PLE 1.39. Sternum: length 1.26, width 1.52. Abdomen: length 1.76, width 1.64. Color (in life Figs 55A, 57A, B/preserved Figs 54D, G, 57C): Carapace: yellowish white, with two dark marks anterolaterally, followed by three pairs of marks laterally/more whitish yellow with patches of red setae conspicuous. Chelicerae: yellowish white, slightly red hue, paturon with dark, curved mark frontally and a dark mark just below clypeus, setae sparse, pale laterally, darker anteriorly where chelicerae meet. Maxillae: yellowish white. Labium: yellowish tan with dusky mark, pale distally. Sternum: yellowish white. Abdomen: dorsally, yellow brown, red spots anteriorly and anteromedially, four around cardiac area, followed by pair of u-shaped marks, four more spots at cardiac area, two pairs of dark spots posteriorly, with white flecks/more orange-red with black marks, spots not as conspicuous; ventrally yellowish white. Legs: yellowish brown with black marks, not quite forming full annulations on Fm, dusky annulations on rest of legs; spination leg I Fm d 1-1-1, pl 1-1-1, Ti v 2-2-2-2-2-2, Mt v 1-1-2-1; leg II Fm d 1-1-1, pl 1-1-1, Ti v 2-2-2-2-2, Mt v 2-2-2-2; leg III Fm d 1-1-1; leg IV Fm d 1-1-1; leg formula: 3421; measurements leg I 8.42 (2.41, 1.33, 2.96, 1.06, 0.66); leg II 9.96 (2.54, 1.39, 3.09, 2.03, 0.91); leg III 13.80 (3.53, 1.45, 3.62, 3.45, 1.81); leg IV 12.4 (2.83, 1.08, 3.48, 3.30, 1.71). Palp: spination Fm d 0-1-0; 2.65 (0.87, 0.37, 0.39, 1.02); with some dark splotches dorsally on Ti and Cy (Figs 54D, 56B); vRTA in ventral view inverted teardrop shaped, dRTA quadrangular in retrolateral view; rbcp smallish; Cy oval-triangular; C projects well beyond the cymbium ventrally and well into the cymbium dorsally (Fig. 56A, B, E), the tip has a small groove (Fig. 56D), plicate; E long, thin, begins at ~ 6 o'clock, ends at 2:30 o'clock, originating from a small TL that is projected ventrally, in sheath around edge of Cy; MA with large base, narrowed to short branch.

##### Variation.

**Male** (paratype, sel_1134, WAM T155510): spination leg I Fm 1-1-1, pl 1-1-0, rl 1-1-1, Ti 1-2-2-1, rl 1-1, d 1-0-0, Mt v 2-2-2; leg II Fm 1-1-1, pl 1-1-0, rl 0-1-0, Ti pl 1-0-1, rl 1-1-0, Mt v 2-2-2; leg III Fm, d 1-1-1 (leg Is new); leg IV Fm d 1-1-1, pr 0-1-1, rl 0-1-1; Ti d 1-1-1-1, pl 1-1, rl 1-1; leg formula different than sel_1131, but at least one leg re-grown. Total length 3.70.

##### Etymology.

The species is named in memory of Joe Haener. Name in genitive case.

##### Distribution.

Known from only the type locality, Kennedy Range National Park, in the Gascoyne Region, Western Australia (Figs 54J, Map 9A).

##### Natural history.

The Gascoyne has an arid tropical climate, warm throughout the year, with mean maximum daily temperatures ranging from 22° in July to 35 °C in January. The region receives ~ 320 days of sunshine per year. The Kennedy Range is located in the Carnarvon xeric shrublands bioregion, with spinifex and mulga, and little tree cover. The region receives < 250 mm of rain a year that occurs bimodally, a large portion from cyclonic activity. The IBRA bioregion is the Carnarvon, and the subregion is Wooramel, a shrubby steppe with red sand dunes that even occur on top of the Kennedy Range (Desmond and Chant 2001).

Multiple instars were collected simultaneously and egg sacs with spiderlings present in May (Fig. 54E). The egg sacs were quite thin to where the eggs and spiderlings were easily visible inside them, similar to the egg sacs of *Karaopsbadgeradda* (Fig. 54B). This is in contrast to other species of *Karaops*, like *K.strayamate* (Fig. 6H), and many *Selenops* species whose egg sacs are much more papery and stronger. Data indicate that adult females, spiderlings, and egg sacs are present from April to May, when temperatures are cooling, and it is becoming wetter. Males are present in November, when it is hotter and drier. After a full year, sel_1133 was not yet an adult (Suppl. material 2: tables S1, S16).

##### Discussion.

This ecoregion is on the onshore part of the Carnarvon Basin, part of the West Australian Shield. The terrain is low, and sediments are recent alluvial, aeolian, and marine sediments over Cretaceous strata (Curry et al. 2008). The Gascoyne is poorly surveyed but from what is known, there are many endemic taxa, including *Karaopsjoehaeneri* sp. nov.

#### ﻿ Karaops karrawarla

Taxon classificationAnimaliaAraneaeSelenopidae

Crews & Harvey, 2011

FA86E567-BEE6-5F90-82EC-1D397E8C6468

Figs 55D, E , 58A–F , Maps 1 , 9A

Karaops karrawarla Crews & Harvey, 2011: 51, figs 39–42 (♂♀, examined).

##### Diagnosis.

This species can be differentiated from other similar species by the genitalia. The copulatory openings are located in the middle of the epigynal plate beneath an m-shaped margin. The lateral lobes can be easily discerned (Fig. 55B, C). The male can be distinguished by the embolus that widens slightly at the tip and the position of the conductor and median apophysis after expansion (Fig. 58A, D–F).

##### Description.

The description of the male and female can be found in Crews and Harvey (2011).

##### Distribution.

This species is only known from the type locality, Bush Bay in the Gascoyne Region, Western Australia (Map 9A).

##### Natural history.

This species is known from the Carnarvon bioregion, Wooramel subregion of the Gascoyne, as is *Karaopsjoehaeneri* sp. nov. For additional information about the bioregion and subregion, see discussion for *K.joehaeneri* sp. nov. above.

##### Discussion.

**Figure 59.**
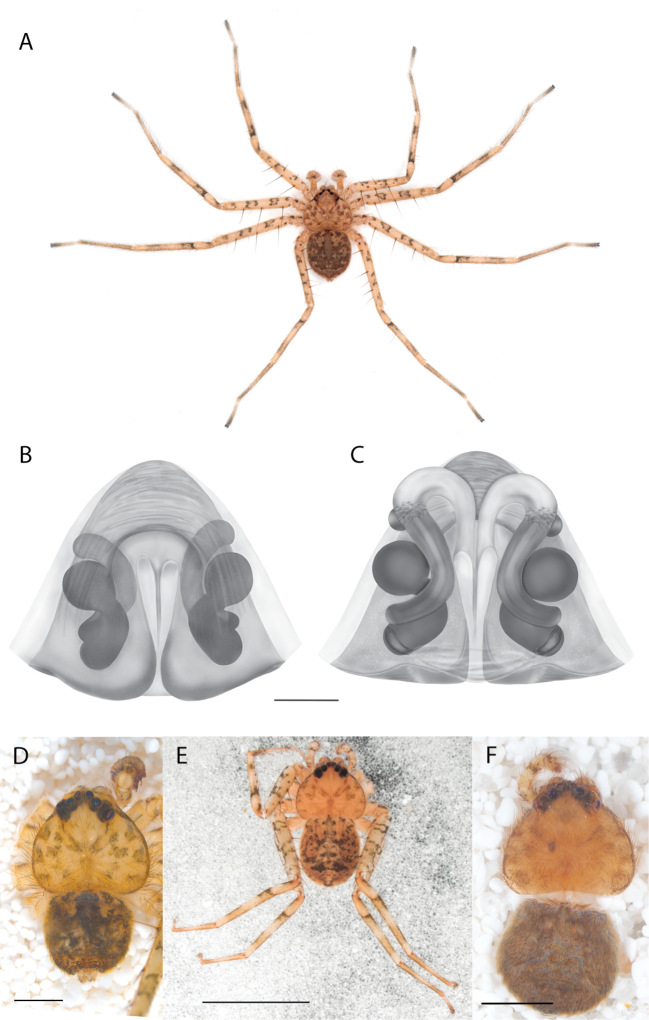
*Karaopsmorganoconnelli* sp. nov. and *Karaopsnyamal* from the Pilbara-Gascoyne species group **A***Karaopsmorganoconnelli* sp. nov., paratype male, Cathedral Gorge, ~ 40 km from Newman (sel_1207, WAM T155583) **B** same, holotype female, epigyne, Orebody 24, ~ 7 km N of Newman (WAM T131095) **C** same, endogyne **D** same, paratype male Cathedral Gorge, ~ 40 km from Newman (sel_1207, WAM T155583) **E** same, holotype female, Orebody 24, ~ 7 km N of Newman (WAM T131095) **F***Karaopsnyamal*, paratype female, Mt. Webber (WAM T107698). Scale bars: 0.5 mm (**B, C**); 1 mm (**D**); 2 mm (**F**); 5 mm (**E**).

The genitalia of both the male and the female of *Karaopskarrawarla* (Figs 55D, E, 58A–F) are re-figured for ease of comparison with *K.joehaeneri* sp. nov. and *K.morganoconnelli* sp. nov. as they closely resemble these species (Figs 55D, E, 58A, D–F, 59B, C). The types were collected in a pitfall trap set in January and collected in May, so there is no way to pinpoint the time of year adults are present other than in the first five months of the year. January–May begins at the hottest and wettest time in the area, but by April and May, temperatures begin to drop, yet rainfall remains at the highest (Suppl. material 2: table S1).

Upon visiting the type locality, it does not appear to be good habitat for selenopid species, as there are no rocks and no trees at the locality or in the vicinity. The collection data have been confirmed to be correct. It has been suggested (J. Waldock, pers. comm.) that the specimens could have washed down to the area from further inland.

#### ﻿ Karaops morganoconnelli sp. nov.

Taxon classificationAnimaliaAraneaeSelenopidae

9C6A876C-85FD-5E3B-98BE-AAC91C566535

https://zoobank.org/0B10B227-EE64-4C63-A6EF-22A182872FDE

**Figure 60.**
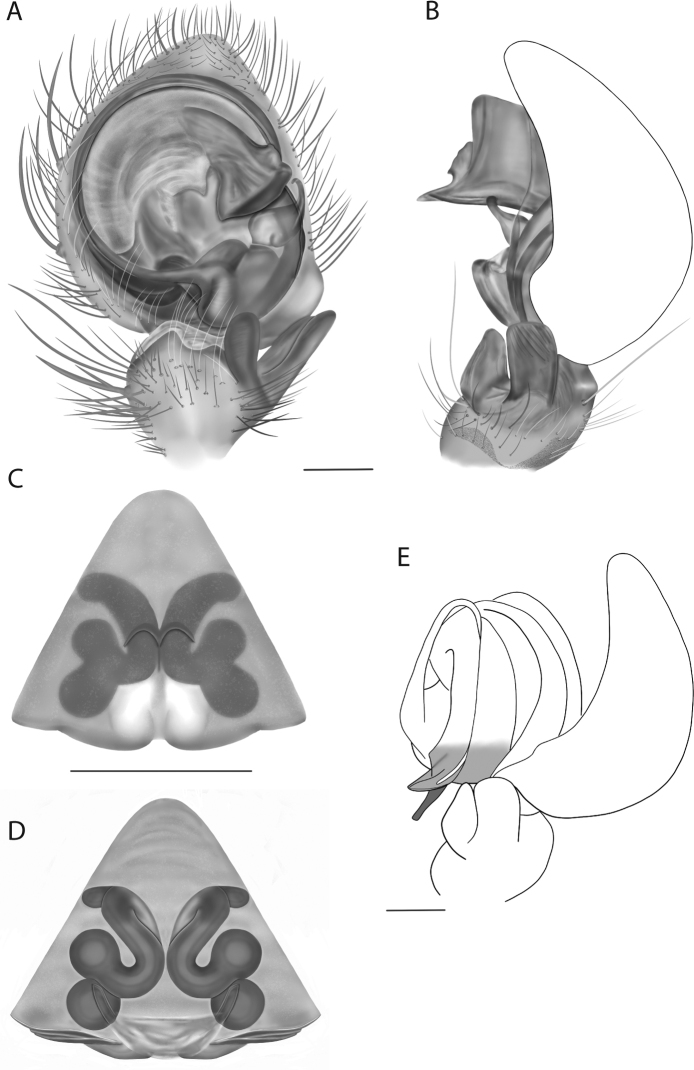
*Karaopsmorganoconnelli* sp. nov. and *Karaopsnyamal* from the Pilbara-Gascoyne species group **A***Karaopsmorganoconnelli* sp. nov., paratype male, palp, ventral, Cathedral Gorge, ~ 40 km from Newman (sel_1207, WAM T155583) **B** same retrolateral **C***Karaopsnyamal*, paratype female, epigyne, Mt. Webber (WAM T107698) **D** same, endogyne **E***Karaopsmorganoconnelli* sp. nov., paratype male, palp, expanded, (sel_1207, WAM T155583); light gray = tip of conductor, dark gray = median apophysis. Scale bars: 0.5 mm.

Figs 59A–E , 60A, B, E , Maps 1 , 9A, B

##### Material examined.

***Holotype***: Western Australia • ♀; Orebody 24, ~ 7 km N of Newman; 23°17'27.66"S, 119°47'38.76"E (WGS84); 29 Apr. 2013–7 May 2013; S. Callan: Biologic Env. leg.; foraging, rock cracks and crevices, gorge, 55-OB24-T1-R; BES:0389; (WAM T131095). ***Paratype***: ♂ (reared in captivity) Cathedral Gorge, ~ 40 km from Newman; 23°16'30.60"S, 119°37'29.55"E; ~ 594 m; 15 May 2016; S. Crews, J. DeJong leg.; outcrop just off the road (on right side, facing North) sel_1207; SCC016_035; (WAM T155583). **Other material examined**: 1 imm.; same data as previous; sel_1206, 1208–1210; (WAM T155582, T155584–155586). **Other records.** 1 imm.; Orebody 24, ~ 7 km N of Newman; 23°17'25.64"S, 119°43'32.66"E; 5–11 Apr. 2013; B. Durrant leg.; foraging; under rocks; (WAM T131088) • 1 imm.; Orebody 25, ~ 5 km N of Newman; 23°19'36.82"S, 119°46'06.43"E; 5–13 Aug. 2013; S. Callan leg.; foraging; gully; (WAM T131234) • 1 imm.; Orebody 25, ~ 5 km N of Newman; 23°19'36.82"S, 119°46'06.43"E; 29 Apr.–7 May 2013; S. Callan leg.; foraging; under rocks; (WAM T131097) • 1 imm.; Orebody 25, ~ 5 km N of Newman; 23°19'24.30"S, 119°47'24.99"E; 5–13 Aug. 2013; S. Callan leg.; foraging; gully; (WAM T131225) • 1 imm.; same as previous; slope/ridge; (WAM T131233) • 1 imm.; Orebody 24, ~ 7 km N of Newman; 23°16'26.31"S, 119°51'15.16"E; 29 Apr.–7 May 2013; S. Callan leg.; foraging; under rocks; (WAM T131098) • same as previous; (WAM T131099) • 1 imm.; Orebody 24, ~ 7 km N of Newman; 23°17'04.62"S, 119°46'23.89"E; 29 Apr.–7 May 2013; S. Callan leg.; foraging; under rocks; (WAM T131104) • 1 imm.; Orebody 24, ~ 7 km N of Newman; 23°17'48.13"S, 119°51'41.88"E; 29 Apr.–7 May 2013; S. Callan leg.; foraging; under rocks; (WAM T131100) • 1 imm.; Orebody 24, ~ 7 km N of Newman; 23°17'24.14"S, 119°46'14.31"E; 5–13 Aug. 2013; S. Callan leg.; foraging; gorge; (WAM T131239) • 1 imm.; Orebody 24, ~ 7 km N of Newman; 23°17'00.58"S, 119°46'10.22"E; 29 Apr.–7 May 2013; S. Callan leg.; foraging; under rocks; (WAM T131102) • 1 imm.; Orebody 24, ~ 7 km N of Newman; 23°17'04.62"S, 119°46'23.89"E; 29 Apr.–7 May 2013; S. Callan leg.; foraging; under rocks; (WAM T131103) • 1 imm.; Orebody 24, ~ 7 km N of Newman; 23°17'04.62"S, 119°46'23.89"E; 5–13 Aug. 2013; S. Callan leg.; foraging; gorge; (WAM T131238) • 1 imm.; Ophthalmia Range, ~ 20 km N. of Newman; 23°10'31.1"S, 119°44'01.8"E; 19 Jun. 2014; J.M. Waldock, C.A. Car leg.; hand forage; hillslope/foot-slope; rock; (WAM T132728) • 1 imm.; Orebody 24, ~ 7 km N of Newman; 23°17'35.04"S, 119°45'25.61"E; 5–11 Apr. 2013; B. Durrant leg.; foraging; under rocks; (WAM T131087) • 1 imm.; Orebody 24, ~ 7 km N of Newman; 23°17'04.04"S, 119°44'25.09"E; 29 Apr.–7 May 2013; S. Callan leg.; foraging; under rocks; (WAM T131093) • 1 imm.; Orebody 24, ~ 7 km N of Newman; 23°17'06.95"S, 119°44'36.64"E; 29 Apr.–7 May 2013; S. Callan leg.; foraging; under rocks; (WAM T131091) • 1 imm.; Orebody 24, ~ 7 km N of Newman; 23°17'04.04"S, 119°44'25.09"E; 29 Apr.–7 May 2013; S. Callan leg.; foraging; under rocks; (WAM T131094) • 1 imm.; Orebody 24, ~ 7 km N of Newman; 23°16'26.97"S, 119°44'23.69"E; 29 Apr.–7 May 2013; S. Callan leg.; foraging; under rocks; (WAM T131092) • 1 imm.; Orebody 24, ~ 7 km N of Newman; 23°17'35.04"S, 119°45'25.61"E; 5 –13 Aug. 2013; S. Callan leg.; foraging; gully; (WAM T131223) • 1 imm.; Orebody 24, ~ 7 km N of Newman; 23°17'06.04"S, 119°44'30.54"E; 5–11 Apr. 2013; B. Durrant leg.; foraging; under rocks; (WAM T131089) • 1 imm.; Orebody 24, ~ 7 km N of Newman; 23°17'11.69"S, 119°44'39.94"E; 29 Apr.–7 May 2013; S. Callan leg.; foraging; under rocks; (WAM T131090) • 1 imm.; Orebody 24, ~ 7 km N of Newman; 23°17'16.32"S, 119°44'48.41"E; 5–13 Aug. 2013; S. Callan leg.; foraging; gully; (WAM T131224) • 1 imm.; Orebody 24, ~ 7 km N of Newman; 23°17'06.95"S, 119°44'36.64"E; 5–13 Aug. 2013; S. Callan leg.; foraging; drainage line; (WAM T131227) • 1 imm.; Orebody 24, ~ 7 km N of Newman; 23°16'57.11"S, 119°44'58.86"E; 5–13 Aug. 2013; S. Callan leg.; foraging; gorge; (WAM T131228) • 1 imm.; Orebody 24, ~ 7 km N of Newman; 23°16'57.11"S, 119°44'58.86"E; 5–13 Aug. 2013; S. Callan leg.; foraging; gorge; (WAM T131230) • 1 imm.; Orebody 24, ~ 7 km N of Newman; 23°16'26.97"S, 119°44'23.69"E; 5–13 Aug. 2013; S. Callan leg.; foraging; slope/ridge; (WAM T131231).

##### Diagnosis.

The female of *Karaopsmorganoconnelli* sp. nov. is similar to other members of the Pilbara-Gascoyne species group but differs by the conformation of the genitalia (Fig. 59B, C). The lateral lobes of the new species are not fused, but they are fused in *K.joehaeneri* sp. nov., *K.julianneae*, *K.badgeradda*, *K.nyamal*, and *K.martamarta*. *Karaopsmorganoconnelli* sp. nov. can be separated from *K.karrawarla* by the copulatory openings situated in a medial depression, and the copulatory ducts of the new species are much wider throughout their length and closer medially.

The male is similar to all the known males of the group by the large conductor that extends dorsally and ventrally (Fig. 60A, B, E). It can be differentiated from *Karaopsjoehaeneri* sp. nov. and *K.karrawarla* by the narrower cymbium that is not elongated retrobasally as much as in the aforementioned. The conductor is shaped differently in both ventral and retrolateral views and is easily seen when the palp is expanded, as is the different position of the median apophysis (Fig. 60E).

##### Description.

**Female** (holotype). Total length 5.89. Carapace: length 2.49, width 3.24. Chelicerae: promargin with three teeth, retromargin with two teeth (1-0-1). Eyes: AER recurved, PER strongly recurved; diameters AME 0.15, ALE 0.11, PME 0.21, PLE 0.32; interdistances AME–PME 0.05, PME-PLE 0.16, ALE–PLE 0.29, PME–PME 0.88, ALE–ALE 1.37, AME–AME 0.43, PLE–PLE 1.70. Sternum: length 1.34, width 1.71. Abdomen: length 3.40, width 2.77. Color: Carapace: orangish with dark marks laterally and in between these and fovea, with dark, slender setae. Chelicerae: orange-brown, paturon with a longitudinal curved mark frontally, mostly dark setae, darkened anteromedially. Maxillae: orange-brown, pale distally. Labium: dark orange-brown. Sternum: orange-brown. Abdomen: dorsally reddish brown with four dark dots anteromedially on either side of darker median band, more flecks laterally, two dark patches medially, two laterally adjacent to the most posterior, two triangular marks anteromedially, connected to one another by a thin, dark mark, tip of abdomen with dusky mark (Fig. 59E); ventrally yellowish. Spinnerets: anterior yellowish, posterior yellowish with dark marks dorsally. Legs: yellowish orange with dark mark prolaterally on Cx and Tr, marks on Fm that do not completely encircle legs, unpigmented medially, darker annulation proximally on Pt, proximally and distally on Ti and Mt, Ti dusky distally/dark marks faded in ethanol, Fm with white setal tufts, setae are long, slightly widened distally; spination leg I Fm d 1-1-1, pr 1-1-1, Ti v 2-2-2-2-2, Mt v 2-2-2-2; leg II missing; leg III Fm d 1-1-1, Ti v 2-2; leg IV Fm d 1-1-1, Ti v 2-2; measurements leg I 8.44 (2.48, 0.96, 2.03, 1.88, 1.09); leg II missing; leg III 11.43 (3.37, 1.20, 3.04, 2.07, 1.75); leg IV 11.62 (3.01, 1.01, 2.85, 3.00, 1.75). Palp: spination Fm d 0-1-2; 2.86 (0.80, 0.54, 0.63, 0.89); claw with ~ 5 teeth. Epigyne: EP somewhat triangular, longer than wide, plicate; MF with narrow m-shaped depressions, in a larger depression rounded anteriorly; LL clearly separated, COs slightly asymmetrical. Endogyne: CDs nearly touching anteriorly, unsclerotized, extended anteriorly slightly beyond more sclerotized part of EP, curved sharply posteriorly, with small ABs where the CDs become sclerotized, textured at this juncture, CDs curve dorsomedially, then ventrally away from one another, wrapped around S, which are round anteriorly, then curved around to allantoid posterior part; FDs located distally on S, directed anterolaterally; small pdf medially.

**Male** (paratype). Total length 3.87. Carapace: length 2.06, width 2.53. Chelicerae: promargin with three teeth, retromargin with two teeth (1-0-1). Eyes: AER recurved, PER strongly recurved; diameters AME 0.16, ALE 0.07, PME 0.16, PLE 0.27; interdistances AME–PME 0.06, PME–ALE 0.11, ALE–PLE 0.11, PME–PME 0.73, ALE–ALE 1.07, AME–AME 0.41, PLE–PLE 1.30. Sternum: length 0.98, width 1.38. Abdomen: length 1.81, width 1.80. Color (in life Fig. 59A/preserved Fig. 59D): Carapace: yellowish brown to golden, three pairs of black marks laterally, with three more in between these and center of carapace/yellowish orange, setae dark, slightly stiff, evenly distributed, with reddish orange, thinner setae laid flat against carapace, especially prominent in ocular region. Chelicerae: yellowish orange, paturon with a longitudinal curved mark frontally, setae paler and less dense laterally, darker anteriorly and where the chelicerae meet. Maxillae: whitish. Labium: dusky, pale distally. Sternum: yellowish white. Abdomen: dorsally with dark brown and golden areas/with black and yellowish orange areas; ventrally, whitish to yellow-orange. Legs: yellowish, Cx and Tr with dark spot prolaterally, dots at base of Fm, two spots with darker edge, unpigmented medially, dots/dusky area at Fm-Pt joint, Pt with dark mark ventrally at Fm-Pt joint, Ti with basal and proximal annulation, basal extends further ventrally, pale annulations basally and distally on Mt, Ta tip a bit dusky; spination leg I Fm d 1-1-1, pl 1-1-1, rl 0-0-1, Ti v 2-2-2-2-2, pl 1-0-0, rl 1-1-1, Mt v 2-2-2-2; leg II Fm d 1-1-1, pl 0-1-1, rl 0-0-1, Ti v 2-2-2-2-2, d 1-2-0, pr 1-1, Mt v 2-2-2-2; leg III Fm d 1-1-1, pr 0-0-1, rl 0-1-1, Tirl 1-1, pr 0-1, v 2-2, d 1-1, Mt 2-1; leg IV Fm d 1-1-1, pr 0-0-1, rl 0-1-1, Ti v 2-2, rl 1-1, pr 0-1, d 1-1, Mt v 2-1; leg formula 3241; measurements leg I 7.29 (2.15, 0.73, 1.92, 1.53, 0.96); leg II 9.06 (2.89, 0.82, 2.29, 1.94, 1.12); leg III 9.14 (2.99, 0.77, 2.24, 2.05, 1.09); leg IV 8.37 (2.61, 0.63, 2.04, 2.11, 0.98). Palp: spination Fm d 0-1-1; 2.14 (0.54, 0.32, 0.38, 0.90); RTA with both branches smallish, approximately same size, vRTA spoon shaped in ventral view, rounded triangular in lateral view, dRTA rectangular in lateral view, plicate distally, Ti with dark mark dorsobasally extending prolaterally (Fig. 60B), dark mark proximal to RTA; rbcp large; Cy roundish to triangular; C large, distally elongated dorsally and ventrally, twisted ventral tip, pointed, tapering in lateral view (Fig. 60B); E long, thin arising from TL at ~ 6 o’clock, ending at ~ 2 o’clock, curved around edge of bulb in CS; MA with a broad base, tapered, increasingly sclerotized distally.

##### Etymology.

This species is named for Morgan O’Connell in recognition the work he has done in the Pilbara. Name in genitive case.

##### Distribution.

Known from only the region of the Ophthalmia Range, Pilbara, Western Australia (Map 9A, B).

##### Natural history.

The species is only found in the Hamersley subregion of the Pilbara bioregion. The Pilbara is arid to tropical. The Hamersley subregion is characterized by mulga woodland over bunch grasses on fine soils and snappy gum over *Triodiabrizoides* Burbidge on skeletal soils (Kendrick 2001a).

The female was present in late April–May when the weather was getting wetter and cooler; however, mostly juveniles were collected, likely due to non-targeted collecting. The male was reared in captivity and did not molt for four months. It then molted twice within two weeks to a penultimate male, and then a month later, in December, to an adult. This is during the hottest and mostly dry part of the year. Necessarily, males and females should be present simultaneously. It is probable that there are females throughout the year, and males more common 1–2 times/year as in other selenopids species. All other records are from juveniles, and various sizes have been found August–April. The immatures and male and female were placed together based on molecular data (Suppl. materials 1, 2: tables S1, S17). This species has been collected by hand in gorges, gullies and drainage lines, on outcrops and slopes and ridges, in rock cracks and crevices, and under rocks.

##### Discussion.

This species is only known from a small area in the Pilbara. It overlaps with one species only known from immatures and is in close proximity to another (Map 9B). The Hamersley subregion contains many organisms with restricted distributions. This area is heavily affected by the iron-ore industry.

#### ﻿ Karaops nyamal

Taxon classificationAnimaliaAraneaeSelenopidae

Crews, 2013

4B65DF0D-F213-5F03-99F4-C4D03E382015

**Figure 61.**
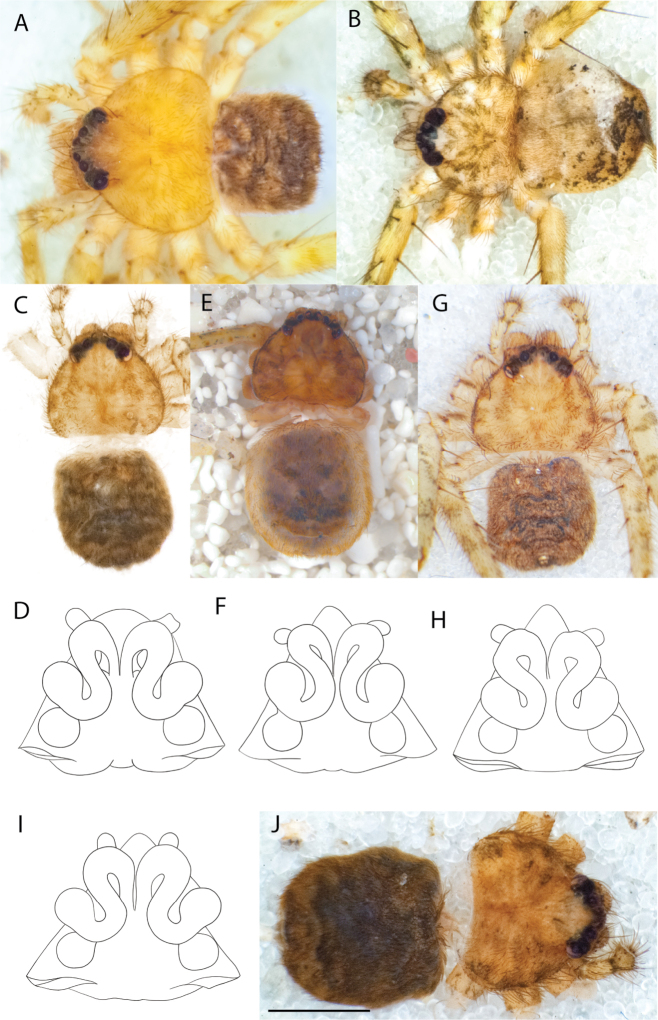
*Karaopsnyangumarta* and *Karaopsnyamal* from the Pilbara-Gascoyne species group **A***Karaopsnyangumarta*, holotype female, Southern Flank to Jinidi Rail (WAM T117876) **B***Karaopsnyangumarta*, paratype male, Southern Flank to Jinidi Rail (WAM T117875) **C***Karaopsnyamal*, Mt. Webber (WAM T107699) **D** same, line drawing showing variation of endogyne **E***Karaopsnyamal* (WAM T125602) **F** same, line drawing showing variation of endogyne **G***Karaopsnyamal*, holotype female, Mt. Webber (WAM T107697) **H** same, line drawing showing variation of endogyne **I***Karaopsnyamal* (WAM T107696) line drawing showing variation of endogyne **J***Karaopsnyamal* (WAM T107696). Scale bar: 2 mm.

Figs 59F , 60C, D , 61C–J , Maps 1 , 9A, B

Karaops nyamal Crews, 2013: 464, figs 31, 32 (♀, examined).

##### Diagnosis.

*Karaopsnyamal* is similar to other species found in the Pilbara and Gascoyne region but can be differentiated by the genitalia. The copulatory openings are located beneath an m-shaped hood centrally on the epigynal plate (Fig. 60C). The epigyne is most similar to that of *Karaopsburbidgei* (Crews and Harvey 2011: fig. 38), *K.karrawarla* (Fig. 55D), and some specimens of *K.martamarta* (Fig. 49C). *Karaopsnyamal* differs from these by the endogyne. In *K.burbidgei*, the spermathecae and accessory bulbs are oval to round and not located on the copulatory ducts, and the copulatory ducts are short. In *K.karrawarla*, the posterior part of each spermatheca is allantoid and the anterior part is round, and the accessory bulbs are found beyond where ducts turn 180° from anterior to posterior. In *K.martamarta*, the copulatory ducts are long, and the spermathecae look like dumbbells. The copulatory ducts curve gently inward then outward, connecting to the spermathecae in the center, narrow part (Fig. 49C–F). In *K.nyamal*, the curvature of the copulatory ducts is more severe, the accessory bulbs are located at the top of the curve, there is no narrow part between the anterior and posterior part of the spermathecae, and the copulatory duct connects to the anterior part (Figs 60C, D, F, H, I).

##### Description.

The description of the female can be found in Crews (2013).

**Male.** Unknown.

##### Distribution.

Known from the type locality in the Northern Pilbara, Western Australia.

##### Natural history.

This species is only known from the type locality in the Pilbara ecoregion, Chichester subregion. This subregion consists of basalt ranges with a shrubby steppe on the plains and snappy gum steppes on the ranges. The climate is semi-desert-tropical, with the wettest months being December–March, the driest August–October, highest temperature November–March, and lowest temperatures May–September. All of the specimens collected are adult females, and they were collected by pitfall traps set from March 31–May 7, a time of transition to drying/cooling, from wettest/hottest in March.

##### Discussion.

The genitalia of the holotype female (WAM T107697) is illustrated for ease of comparison with similar species (Figs 59C, 60C, D). The genitalia of the paratype female (WAM T107698) and others (Fig. 61C–M) are illustrated to show variation from the holotype (Fig. 61D, F, H, I). There is variation in the shape of the accessory bulb and the spaces between the turns of the copulatory ducts. The Chichester subregion is a center of endemism for many, many taxa. Despite several hours looking for the species, none were found, and what was collected nearby was another species. Permission to go to the type locality was not granted because of the large Mt. Webber Iron Ore Mine, which opened in 2013. The habitat and landforms have been severely altered by mining in this area (Suppl. material 2: table S1).

#### ﻿ Karaops nyangumarta

Taxon classificationAnimaliaAraneaeSelenopidae

Crews, 2013

10CEAA5C-A86D-5687-BA09-0395EA1D0769

**Figure 62.**
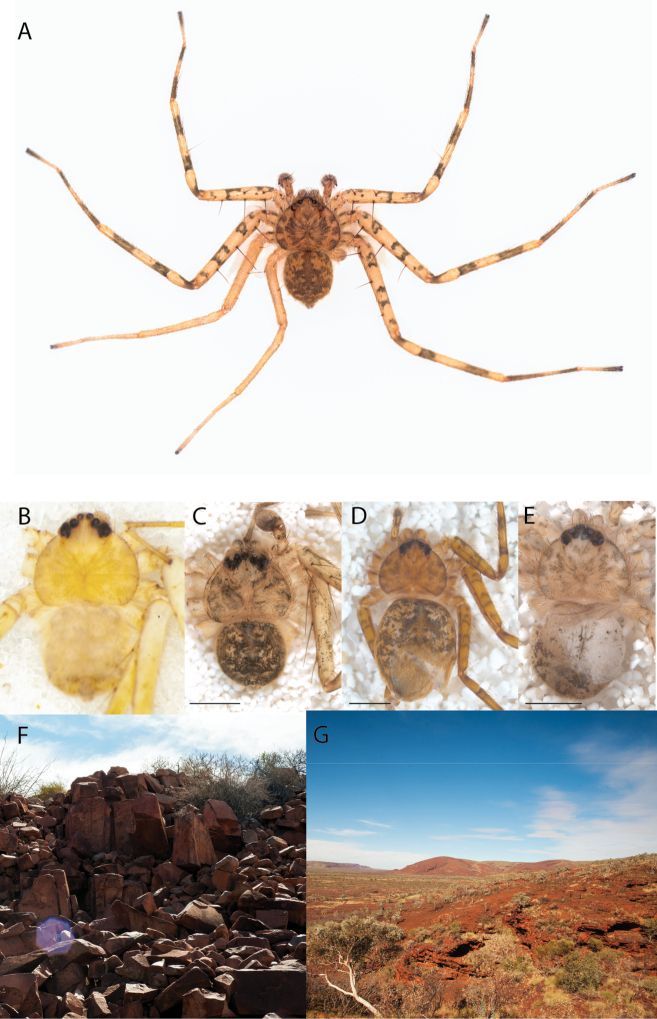
*Karaopsnyangumarta* and *Karaopsjaburrara* from the Pilbara-Gascoyne species group **A** adult male *Karaopsnyangumarta*, Mt. Nameless (sel_1179, WAM T155555) **B***Karaopsjaburrara*, male holotype, vic. Wickham (WAM T79397) **C***Karaopsnyangumarta*, adult male, Mt. Meharry (sel_1197, WAM T155573) **D***Karaopsnyangumarta*, holotype female, 98.5 km W of Newman (WAM T117876) **E***Karaopsnyangumarta*, paratype male, 98.5 km W of Newman (WAM T117875) **F** habitat of *Karaopsjaburrara*, vic. Wickham **G** habitat around Mt. Meharry where *Karaopsnyangumarta* was collected. Scale bars : 2 mm.

**Figure 63.**
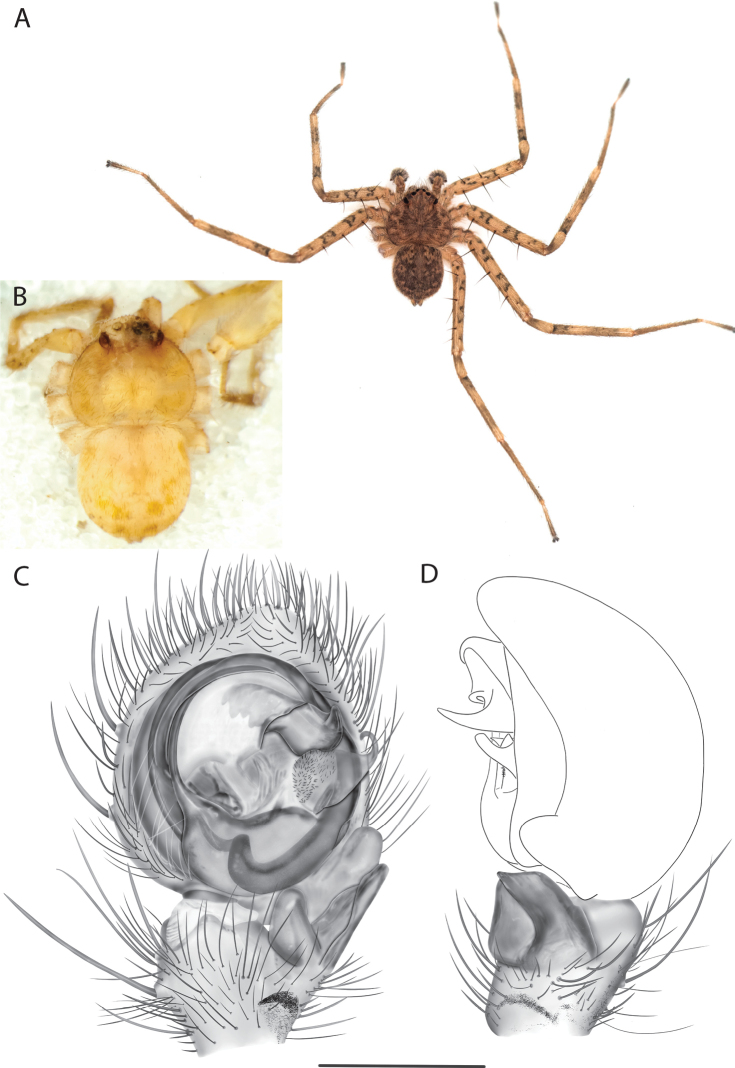
*Karaopsnyangumarta* and *Karaopsnyiyaparli* from the Pilbara-Gascoyne species group **A** adult male *Karaopsnyangumarta*, Mt. Nameless (sel_1197, T155573) **B***Karaopsnyiyaparli*, ex *Karaopsyindjibarndi*, syn. nov., holotype male, 6 km N of Millstream (WAM T79405) **C***Karaopsnyangumarta*, palp, ventral (sel_1179, WAM T155555) **D** same, retrolateral. Scale bar: 0.5 mm.

Figs 61A, B , 62A, C–E, G , 63A, C, D , Maps 1A , 9A, B

Karaops nyangumarta Crews, 2013: 461, figs 25–28 (♀, examined).

##### Material examined.

Western Australia • 7 imm., ♂ (reared in captivity); Tom Price, Mt. Nameless, just after 4WD Mt. Nameless sign, pullout ~ 15 m from parking area; 22°43'38.45"S, 117°44'58.13"E; ~ 765 m; 13 May 2016; S. Crews, J. DeJong leg.; at night under rocks in creek bed; sel_1175–1182; SCC16_030; (WAM T155551–155558) • 3 imm.; Karijini National Park, Juna Downs Road at Wildflower Range, right side of road heading south; 22°47'38.05"S, 118°25'20.49"E; ~ 801 m; 14 May 2016; S. Crews, J. DeJong leg.; on and under flat rocks on roadside, at night; sel_1191–1193; SCC16_032; (WAM T155567–155569) • 1♂, 3 imm.; near Mt. Meharry, just before road to Mt. Meharry curves east; 22°56'20.95"S, 118°34'2.16"E; ~ 803 m; 14 May 2016; S. Crews, J. DeJong leg.; under rocks in gorge; sel_1194–1197; SCC16_033; (WAM T155570–155573). **New records.** 1 imm.; Tom Price, 2 km S of Tom Price, A20080815.CH23-01; 22°42'46"S, 117°46'33"E; 15 Aug. 2008; Z. Hamilton leg.; under rock; (WAM T92503) • 8 imm.; 16.8 km SW of Tom Price, site 1000-tSX1; 22°46'46.30"S, 117°39'34.93"E; 1 Apr.–1 Jun. 2012; E. S. Volschenk leg.; litter sifting; (WAM T124820) • 2 imm.; 14 km WSW of Tom Price, site 1000-ts09; 22°45'41.22"S, 117°40'30.76"E; 30 Apr. 2012; E.S. Volschenk leg.; foraging; (WAM T124816) • 1 imm.; 30 km SW of Tom Price, site 1000-ts01; 22°53'46.16"S, 117°36'05.33"E; 20 Jun. 2012; E.S. Volschenk leg.; foraging; (WAM T124817) • 1 imm.; 16 km WSW of Tom Price, site 1000-ts12; 22°45'56.12"S, 117°39'22.91"E; 18 Jun. 2012; E.S. Volschenk leg.; foraging; (WAM T124819) • 1 imm.; 17 km WSW of Mt. Brockman, site 999-E3; 22°32'03.03"S, 117°09'37.90"E; 23 Jun. 2012; P. Langlands leg.; foraging; (WAM T124810) • 1 imm.; 129.2 km NW of Newman; 22°49'54"S, 118°36'09"E; 30 Mar. 2012; N. Watson, P. Brooshooft leg.; under rock; rocky gorge; (WAM T122804) • same as previous; (WAM T122805) • 1 imm.; 127.1 km NW of Newman; 22°50'13"S, 118°37'43"E; 30 Mar. 2012; C. Cole, N. Watson leg.; under rock; rocky gorge; (WAM T122806) • same as previous; (WAM T122807) • 1 imm.; -23.209548, 118.753; 16 Oct. 2018; A. Slabber leg.; gorge, gull, hand collected; BMR00069 • 1 imm.; Mudlark, 96 km WNW of Newman; 23°02'25"S, 118°51'06"E; 5 Jul. 2011; N. Watson leg.; by hand; collected from under rocks; (WAM T116567) • 1 imm.; Mudlark, 98 km WNW of Newman; 23°04'23"S, 118°49'12"E; 6 Jul. 2011; C. Cole, M. Greenham leg.; by hand; collected from under rocks; (WAM T116569) • same as previous; (WAM T116568) • 1 imm.; 98.5 km W of Newman; 23°04'14"S, 118°49'16"E; 5 Aug. 2011; N. Watson, J. Tatler leg.; turning rock; under rocks; (WAM T117877) • 1 imm.; same as previous; (WAM T117878) • 1 imm.; same as previous; (WAM T117879) • 1 imm.; same as previous; (WAM T117880) • 1 imm.; Mudlark, 98 km WNW of Newman; 23°04'23"S, 118°49'30"E; 6 Jul. 2011; N. Watson, J. Cairnes leg.; by hand; collected from under rocks; (WAM T116575) • 1 imm.; 119.4 km NW of Newman; 22°56'2"S, 118°39'50"E; 31 Mar. 2012; C. Cole; J. Tatler, N. Watson, P. Brooshooft leg.; under rock; rocky gorge; (WAM T122810).

##### Diagnosis.

The genitalia are similar to those other species in the group by having medially located copulatory openings, long copulatory ducts, and spermathecae shaped like dumbbells. *Karaopsnyangumarta* can be differentiated by the two oblong depressions toward the posterior where the copulatory openings are located on the epigyne. The endogyne differs by having fully sclerotized copulatory ducts, the copulatory ducts fold back on themselves rather than out to the sides, the ducts are narrow and short, the accessory bulbs are quite large and oval (erroneously labeled as spermathecae in Crews (2013)) and do not extend anteriorly of the spermathecae (Crews 2013: figs 25, 26).

The diagnosis of the male has been emended because of its similarity to the previously undescribed male of *Karaopsbanyjima*. More detailed differences can be found in the description of *K.banyjima*. Both *K.banyjima* and *K.nyangumarta* have a very large tegular lobe, but they can be easily separated by the median apophysis. In *K.nyangumarta*, there are many small spinules at the base, whereas there are none in *K.banyjima* (Figs 63C, 69A–C).

##### Description.

The description of the male (Figs 61B, 62C, E) has been emended with data from live spiders (Figs 62A, 63A). No live females were collected or reared. Color. Carapace: dark brown medially, three pairs of dark spots at lateral edges, tan background, very setose. Abdomen: dorsally highly setose, dark longitudinal band medially, just more than half of abdomen length, pairs of small, dark spots on either side, paler brown on sides, interspersed with darker (darker than median band) brown, mottled, posteriorly some pale setal tufts forming horizontal, w-shaped pattern, pale setal tufts at posterior; spinnerets dusky; setae appear soft and thin, very dense. Legs: yellowish brown; dark spots prolaterally and retrolaterally on Cx, dark mark prolaterally on Tr, Fm with markings that do not completely encircle leg, centers of markings unpigmented, Pt with dark annulation basally, two annulations on Mt and Ti basally and distally, Ta dark at tip. The full description of the male and female can be found in Crews (2013).

##### Distribution.

Known from the Hamersley subregion in the Pilbara, Western Australia (Fig. 62G, Map 9A, B).

##### Natural history.

The animals in captivity matured around the same time that the types were collected (August). The males reared matured in September and October. In August, it is cooler and drying. In September and October, it is dry and starting to get warmer (Suppl. material 2: tables S1, S18). More information about the Hamersley subregion can be found in the descriptions of other species found there.

##### Discussion.

In the original description there was a typo, and the male described is 5.77, not 6.77. The two males reared were sel_1179 and sel_1197, and they are 4.40 and 4.71, respectively. This species occurs in the Hamersley subregion of the Pilbara with *Karaopsmartamarta* and *K.banyjima*. Based on collecting records, it is clear that it overlaps with *K.banyjima*, and with more collecting it will probably overlap with *K.martamarta*, too. This species often gets misidentified. In Crews (2013), a record for a specimen from East of Meenatheena Outcamp, hundreds of kilometers away from where any *K.nyangumarta* had been found, was given for this species. The author doubted the identification and was sent images of this specimen. It is now known as an undescribed species that will be described in a future publication.

#### ﻿ Karaops jaburrara

Taxon classificationAnimaliaAraneaeSelenopidae

Crews, 2013

7F886668-672B-5C09-B7A9-38714E3D3A97

Fig. 62B, F , Maps 1 , 9A, B

Karaops jaburrara Crews, 2013: 458, figs 21, 22 (♂, examined).

##### New records.

(These are specimens considered *Karaopsjaburrara* based on molecular and geographic data – see Discussion). Western Australia • 5 imm.; Burrup Peninsula off Burrup Road; 20°39.848'S, 116°44.203'E; 11 May 2016; ~ 39 m; S. Crews, J. DeJong leg.; under rocks; sel_1141–1145; SCC16_021; (WAM T155517–T155521) • 7 imm.; ~ 13.5 km W Wickham, out Cleaverville Road; 20°41.086'S, 117°00.487'E; 11 May 2016; ~ 19 m; S. Crews, J. DeJong leg.; under rocks, on rocks, on ground at dusk; sel_1146–1152; SCC16_022; (WAM T155522–T155528).

##### Diagnosis.

This species can be differentiated from the other species of the group by the dRTA, which is toothed along the upper margin in ventral view (Crews 2013: fig. 21).

##### Description.

The description of the male can be found in Crews (2013).

**Female.** Unknown.

##### Distribution.

This species is only known from the type locality in the Pilbara, west of Wickham, Western Australia.

##### Natural history.

This species occurs in the Chichester subregion of the Pilbara bioregion (Fig. 62F), which is absurdly diverse in *Karaops* species (Suppl. material 2: table S1). For details on the subregion, see other species descriptions.

##### Discussion.

*Karaopsjaburrara* (Fig. 62B) is only known from a single adult male specimen collected in an ethylene glycol pitfall trap that was out for 15 months. Thus, there is no way to know when the animals are active or adults. Other species collected nearby are known from males or males and females, so this is not a case of an unmatched sex. Rearing juveniles from the collection localities failed. Molecular data indicate that juvenile specimens collected in the area form a clade, and even though there are other species that have been found nearby, none have been found at these particular localities (Suppl. material 1). Because of this, it is believed that these specimens are indeed *K.jaburrara*, and records are given above.

#### ﻿ Karaops nyiyaparli

Taxon classificationAnimaliaAraneaeSelenopidae

Crews, 2013

839620ED-F384-5E15-8464-9430B742400D

Figs 63B , 68B, D, G , 69D–F , 70B , 71A–F , 72A–C, E , 73A–D , Maps 1 , 9A, B

Karaops nyiyaparli Crews, 2013: 450, figs 5, 6 (♀, examined).
Karaops yindjibarndi Crews, 2013: 458, figs 19–20. syn. nov.

##### New records.

Western Australia • 1 imm.; 113 km NNW of Newman; -22.335190, 119.653100; 23 Mar.–29 Apr. 2011; E.S. Volschenk leg.; foraging on ridgetop; (WAM T112018) • 1 imm.; 115 km N of Newman; -22.313470, 119.788600; 25 Mar.–29 Apr. 2011; E.S. Volschenk leg.; litter sifting; (WAM T112019) • 1 imm.; ~ 3 km W of Wodgina Mine Site, site H2; -21.178970, 118.646300; 3 Mar. 2011; A. Rakimov leg.; hand collected; *Ficus* on ridge; (WAM T113414) • 1 imm.; Karratha to Millstream-Chichester National Park; -21.208000, 117.045100; 19–27 Jun. 2011; S. White, F. Bokhari leg.; foraged on rocks; (WAM T114678) • 1 imm.; ~ 60 km SW of Marble Bar; -21.611060, 119.031900; 29 Feb.–29 Mar. 2012; A. Slabber leg.; target searching on granite outcrop; (WAM T122639) • 1 imm.; BHP Main Rail, 208 km SSE of Port Hedland; -22.204850, 119.029900; 23 Apr. 2012; S. Catomore leg.; foraging on South-facing aspect of BIF gully/gorge; (WAM T123594) • 1 imm.; BHP Main Rail, 190 km SSE of Port Hedland; -22.052140, 118.987100; 23 Apr. 2012; S. Catomore leg.; foraging on South-facing aspect (overhang) of rocky gulley; (WAM T123595) • 1 imm.; BHP Main Rail, 190 km SSE of Port Hedland; -22.026480, 119.002900; 23–04 Apr. 2012; S. Callan leg.; litter sift on ridge slope, large rocks and cracks; (WAM T123596) • 1 imm.; BHP Main Rail, 190 km SSE of Port Hedland; -22.01464, 119.0058; 23 Apr 2012; S. Callan leg.; ridge slope, large rocks and cracks; (WAM T123597) • 1 imm.; ~ 106 km S of Port Hedland; -21.345170, 118.748600; 13–15 Apr. 2013; N. Dight leg.; foraging on footslope; (WAM T128002) • same as prev.; -21.345, 118.749; (WAM T128003) • same as prev.; ~ 95 km S of Port Hedland; -21.243, 118.668; (WAM T128008) • same as prev.; ~ 82 km S of Port Hedland; -21.117, 118.657; (WAM T128009) • same as prev.; (WAM T128010) • same as prev.; ~ 115 km S of Port Hedland; -21.430, 118.783; (WAM T128011) • 1 imm.; Mt. Webber, ~ 200 km SE of Port Hedland; 7–24 Mar. 2014; A. Slabber leg.; targeted searching, riverine; (WAM T128795) • 1 imm.; ~ 78 km NE of Wittenoom; -21.829, 118.951; 15 Apr. 2014; N. Dight leg.; foraging; (WAM T135298) • 1 imm.; 80 km S of Whim Creek; -21.580, 117.882; 3–8 Jun. 2014; N. Dight leg.; foraging; (WAM T135301) • 2 imm.; 50 km S of Whim Creek; -21.262, 117.936; 3–8 Jun. 2014; N. Dight leg.; foraging; (WAM T135303) • 4 imm.; 95 km S of Whim Creek; -21.688, 117.872; 3–8 Jun. 2014; N. Dight leg.; foraging; (WAM T135304) • 1 imm.; 75 km S of Whim Creek; -21.512, 117.940; 3–8 Jun. 2014; N. Dight leg.; foraging; (WAM T135305) • 1 imm.; 41 km S of Whim Creek; -21.180, 117.951; 14–20 Jul. 2014; N. Dight leg.; foraging; (WAM T135306) • same as prev.; (WAM T135307) • 1 penultimate ♀; Millstream-Chichester National Park, along Karratha Tom Price Road; 20°58.595'S, 117°06.158'E; 12 May 2016; S. Crews, J. DeJong leg.; under rock; sel_1159; SCC16_024; (WAM T155535) • 3 imm.; Millstream-Chichester National Park, near Python Pool, N side of Roebourne-Wittenoom Road; 21°19.4968'S, 117°13.1746'E; ~ 349 m; 12 May 2016; S. Crews, J. DeJong leg.; under flat rocks on hillside; sel_1163–1165; SCC16_026; (WAM T155539– T155541) • 3 imm.; Millstream-Chichester National Park, Python Pool, hillside along trail; 21°20.4783'S 117°11.3150'E; 12 May 2016; S. Crews, J. DeJong leg.; under rock; sel_1160–1162; SCC16_025; (WAM T155536–T155538) • 2♀ (one reared in captivity); Millstream-Chichester National Park, Water District Road, water tanks; 21°32'24.67"S, 117°03'16.61"E; ~ 331 m; 13 May 2016; S. Crews, J. DeJong leg.; under rock; sel_1166–1167; SCC16_027; (WAM T155542–T155543) • 1♂(reared in captivity), 4 imm.; Coolawanyah Station, 4.8 km NNE of homestead, hill, S side of Roebourne-Wittenoom Road; 21°45'57.29"S, 117°49'34.25"E; 13 May 2016; S. Crews, J. DeJong leg.; under rocks on hillside; sel_1168–1172; SCC16_028; (WAM T155544–T155548) • 1 imm.; Bonney Downs Station, Roy Hill Railway line, ~ 14 km SW of Bonney Downs homestead; 22°17'54.36"S, 119°52'20.78"E; 16 May 2016; S. Crews, J. DeJong leg.; under rock; sel_1212; SCC16_037; (WAM T155588) • 1♀, 4 imm. (reared in captivity); Bonney Downs Station, Roy Hill Railway line, ~ 14 km SW of Bonney Downs homestead; 22°17'48.28"S, 119°52'31.54"E; ~ 497 m; 16 May 2016; S. Crews, J. DeJong leg.; sel_1213–1217; SCC16_038; (WAM T155589–T155593) • 4 imm.; Bonney Downs Station, Roy Hill Railway line, ~ 13 km SW of Bonney Downs homestead; 22°17'14.61"S, 119°53'06.05"E; ~ 522 m; 16 May 2016; S. Crews, J. DeJong leg.; sel_1218–1221; SCC16_039; (WAM T155594–T155597) • 1♀, 3 imm. (reared in captivity); beside BHP Rail Line, ~ 59 km SSW of Mt. Webber; 22°08'13.36"S, 119°01'28.02"E; ~ 475 m; 17 May 2016; S. Crews, J. DeJong leg.; lots of egg sacs; sel_1228–1231; SCC16_042; (WAM T155604–T155607); • 1 imm.; Kanagan Station, ~ 6 km NW of Mt. Tinstone [mine]; 21°08'50.19"S, 118°39'39.58"E; 17 May 2016; S. Crews, J. DeJong leg.; under rock; sel_1232; SCC16_044; (WAM T155608).

##### Diagnosis.

The female is most similar to *Karaopskariyarra* in that the copulatory ducts are located in a depression of the median field of the epigyne, and the lateral lobes are easy to distinguish (Crews 2013: figs 5, 7). The endogynes are also similar in that the spermathecae are dumbbell shaped, and the accessory bulbs (erroneously labeled as spermathecae in Crews (2013)) are long and thin. They can be differentiated by the shape of the depression on the endogyne. In *K.nyiyaparli*, it is oval and in *K.kariyarra* it is heart shaped. The lateral lobes of *K.kariyarra* are pointed at the basomedial margin, and they are not in *K.nyiyaparli*. The accessory bulbs are extremely tiny in *K.kariyarra* (Crews 2013: figs 6, 8).

The male can be distinguished from other species in the Pilbara by the dRTA and vRTA of equal length in lateral view. The embolus is hooked and does not follow the perimeter of the bulb (Crews 2013: figs 19, 20).

##### Addenda to original description.

Color (in life Figs 70B–E, 71D, F, 72A, B, E, 73A/preserved Figs 63B, 68B, D, G, H): in nature, on red rocks, appears much more reddish (Fig. 73A), but pale and dark areas mentioned below are still applicable. Carapace: golden brown to brown, three pairs of dark marks on lateral edges, dark spots between edges and middle; highly setose, reddish setae around eyes, posterior to eyes, row of whitish setae following curve of eye area, mixture of dark brown, whitish, pale brown, orangish setae. Abdomen: dorsally extremely setose, row of pale setae along anterior margin, posterior to that, orangish brown and whitish, two dark marks surrounded by pale setae, either side of center, not touching, followed by two dark dots, surrounded by pale setae, separated from anterior by paler brown setae, horizontal dark bands from center to lateral edges, surrounded by pale setae, just posterior to pale setae, horizontal dark mark, does not extend to lateral edges, surrounded by pale setae, pale brown orangish setae with dark flecks to end; darkest parts on carapace and abdomen from pigmentation, other colors from setae, the setae are soft, slender but very dense. Legs: yellowish to golden brown, dark spot prolaterally on Cx and Tr, Fm with markings that do not completely encircle leg, markings not pigmented in center, Pt with dark annulation basally, some specimens with dark mark prolaterally on Pt, two annulations on Ti, centers darker than those markings on Fm but not completely pigmented, two annulations on Mt, basal, distal, Ta dark at tip.

**Male.** The description of the male can be found in Crews (2013: sub *Karaopsyindjibarndi*).

##### Variation.

Additional specimens were measured, and the sizes range from 5.78–8.31 (holotype 6.49, sel_1166 8.31, sel_1213 5.781, sel_1230 5.879).

##### Distribution.

Primarily found throughout the Chichester region of the Pilbara, Western Australia (Figs 69D, F, 71B, 72B, D, 73B, C, Map 9A, B).

##### Natural history.

*Karaopsnyiyaparli* is found in the Chichester subregion of the Pilbara bioregion. This subregion harbors myriad *Karaops* species, at least eight. The climate of the Pilbara is arid-tropical and generally hottest from October–April and wettest from January–March. Adult females have been collected in March and/or April (collected in a pitfall trap left out for a month, so precise month unknown) and May, and egg sacs (Fig. 71C, E) are present in May. March–May is a time of transition from the hottest, wettest time to the coolest, driest time in the region. A male was reared in captivity, reaching adulthood in March, which is during the hottest and wettest period. An adult female (sel_1166) made an egg sac shortly after collection in May, with ~ 30 eggs; however, not all of them were viable. The females (Fig. 69B, D, E) continued to eat and died at different times (it is unknown how long they had been adults when collected), one living an additional seven months from collection, all feeding until death. In captivity, maturity overlapped that found in nature (Suppl. material 2: table S19).

##### Discussion.

The male was described as *Karaopsyindjibarndi* (Crews 2013). Molecular data indicate that *K.yindjibarndi* and *K.nyiyaparli* are the same species. Thus, *K.yindjibarndi* is syn. nov. In this subregion, the type localities of all of the species collected previously were re-visited. Some of the juveniles that were collected may be some of these previously-described species or new species. At almost every site in the area, *K.nyiyaparli* was collected and no other species. The only probable undescribed species are juveniles, and those were only to the north and east of collections of *K.nyiyaparli*. This species has been collected via litter sifting and hand collecting, under rocks, on rocks, targeted on granite outcrops, ridge slopes, and cracks.

Based on locality data and molecular data, sel_1171 is considered to be an adult although the palps are not fully formed. The spider tore its palps off during molting. Typically, when this happens, all of the parts are there, but a bit deformed from being trapped in the exuvia; however, the spiders can often still be identified as the more sclerotized RTA is not damaged. With this specimen, the palps were not fully formed, and there were no structures visible.

The author was bitten on the back of her hand by one of the specimens (sel_1172) on 2 March 2016 at 6:45pm. The spider was sitting on her hand to be fed sugar water (a dietary supplement if they were not eating enough). It had been sitting perfectly still for several minutes. The author then felt the fangs and pinching of the strong chelicerae and could feel the venom entering, moderately painful for such a small animal. At first, it felt a bit like after coming into contact with a nettle, then it stopped for ~ 30 s, then it began again, dissipating within a few hours. The next day there was a small, slightly raised area. There was no pain by 4 March, but the small area had become redder. By 6 March, it was swollen into a small, red bump, a few mm in diameter. It neither hurt nor itched and remained the same after one week. After a few more days, it was completely gone.

This is one of the more widespread species in the Chichester subregion, overlapping with most of the species there: *Karaopsnyamal*, *K.kariyarra* Crews, 2013, *K.yurlburr*, *K.feedtime* Crews, 2013, and *K.forteyi* Crews, 2013. Two of these species, *K.yurlburr* and *K.forteyi*, were collected in pitfall traps set for > 1 year; thus, when adults are present cannot be pinpointed. These are also the only ones of the five for which both sexes have been collected. The other three species were collected in pitfall traps that were set from March–April or March–May. It is known that females of *K.nyiyaparli* are found during these months, indicating that there is no temporal barrier to adults of different species co-existing. After extensive collecting at or near the type localities of each of these species, only *K.nyiyaparli* was collected. Because these six species were collected in pitfall traps, they are in fairly poor condition (Fig. 73D–I). It is unknown how they look in life, and the poor preservation has destroyed many features. The collecting efforts made here allowed the documentation of how *K.nyiyaparli* looks in nature (Figs 71F, 71A, 72A, C), in life in captivity, and freshly preserved. Assuming that none of these six species look exactly like *K.nyiyaparli*, it is now possible to determine if one has found *K.nyiyaparli* in the field. Knowing this allows collectors to spend more time and effort in a particular locality if *K.nyiyaparli* is not the species for which they are searching.

#### ﻿ Karaops durrantorum sp. nov.

Taxon classificationAnimaliaAraneaeSelenopidae

7ED38587-1425-5B41-ACE5-83D8BCA83806

https://zoobank.org/97BA1BD5-54EA-4A2F-BA7A-AF4F7B99FC5E

**Figure 64.**
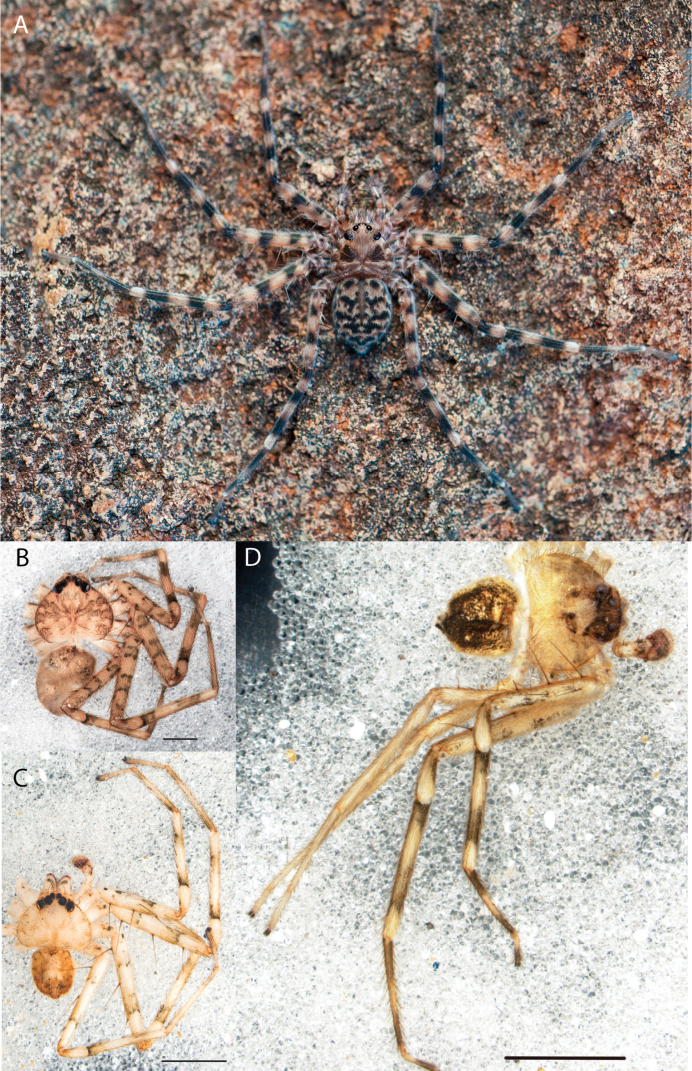
*Karaops* spp. from the Pilbara-Gascoyne species group **A***Karaops* sp. Harding Dam **B***Karaopsbanyjima*, adult male, palps not fully developed, Newman-Port Hedland Road (sel_1198, WAM T155574) **C***Karaopsdurrantorum* sp. nov., holotype male, Mandu Mandu Gorge, Cape Range National Park (sel_1136, WAM T155512) **D***Karaopsbanyjima*, adult male, Dale’s Gorge, Karijini National Park (sel_1187, WAM T155563). Scale bars: 2 mm.

**Figure 65.**
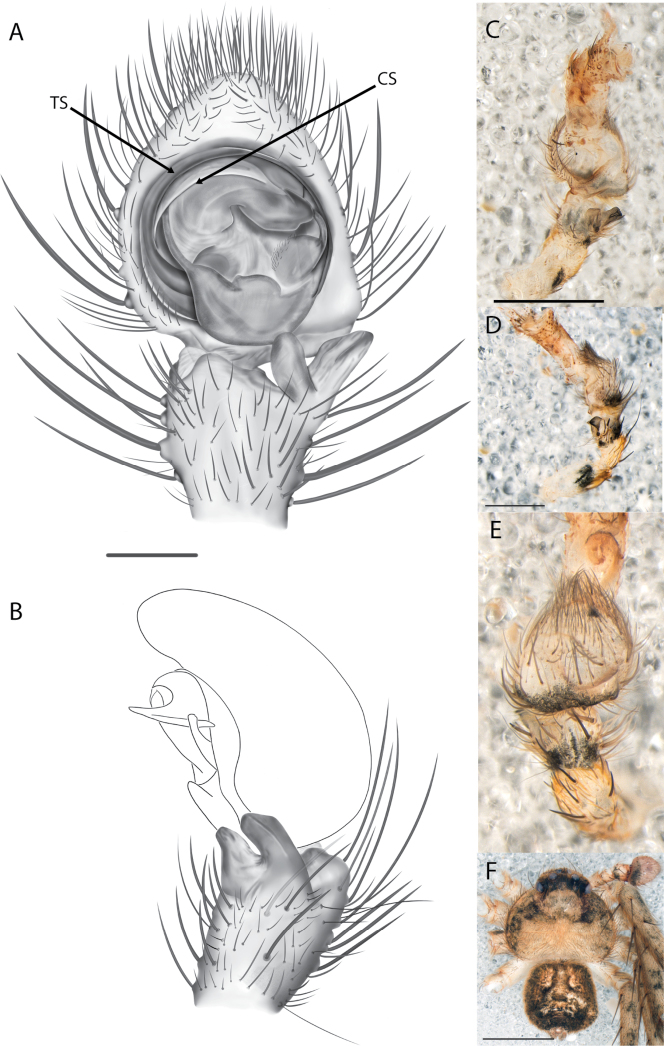
*Karaopsdurrantorum* sp. nov. and *Karaopsbanyjima* from the Pilbara-Gascoyne species group **A***Karaopsdurrantorum* sp. nov., holotype male, palp ventral, Mandu Mandu Gorge, Cape Range National Park (sel_1136; WAM T155512); TS = Tegular sheath, CS = Conductor sheath **B** same, retrolateral **C***Karaopsbanyjima*, adult male, palp, ventral, palps not fully developed, Newman-Port Hedland Road (sel_1198, WAM T155574) **D** same, retrolateral **E** same, dorsal **F***Karaopsbanyjima*, adult male, ~ 1.5 km from Hamersley Gorge on side of road (sel_1174, WAM T155550). Scale bars: 0.2 mm (**A, B**); 1 mm (**C–E**); 2 mm (**F**).

**Figure 66.**
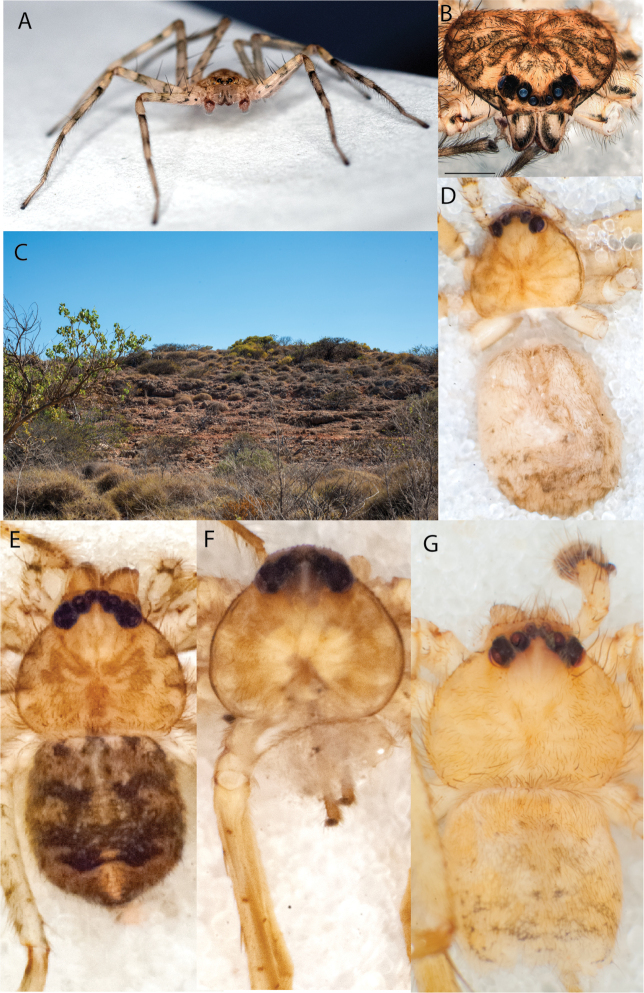
*Karaops* spp. from the Pilbara-Gascoyne species group **A***Karaopsdurrantorum* sp. nov., holotype male, Mandu Mandu Gorge, Cape Range National Park (sel_1136, WAM T155512) **B***Karaopsbanyjima*, adult male, face, palps not fully developed, Newman-Port Hedland Road (sel_1198, WAM T155574) **C** Mandu Mandu Gorge, habitat of *Karaopsdurrantorum* sp. nov. **D***Karaopsyurlburr*, holotype female, Python Pool, Millstream-Chichester National Park (WAM T79403) **E***Karaopsbanyjima*, holotype female, NW of Newman (WAM T101159) **F***Karaopsyurlburr*, holotype male, Python Pool, Millstream-Chichester National Park (WAM T79403) **G***Karaopsngarluma*, holotype male, SW of Roebourne (WAM T79396). Scale bar: 1 mm.

Figs 64C , 65A, B , 66A, C ; Maps 1 , 9A

##### Material examined.

***Holotype***: Western Australia • ♂ (reared in captivity); Cape Range National Park, Mandu Mandu Gorge Trail, hill above car park; 22°09.045'S, 113°52.999'E; ~ 16 m; 10 May 2016; S. Crews, J. DeJong leg.; under rocks in area with spinifex; sel_1136; SCC16_019; (WAM T155512). **Other material examined**: 3 imm. (2 different instars present); same data as holotype; sel_1135, 1137–1138; (WAM T155513, 155514) • 2 imm.; Cape Range National Park, Shothole Canyon Road; 22°03.118'S, 114°01.276'E; ~ 90 m; 10 May 2016; S. Crews, J. DeJong leg.; under rocks; sel_1139–1140; SCC16_020 (WAM T155515, 155516).

##### Diagnosis.

*Karaopsdurrantorum* sp. nov. is most similar to *K.burbidgei* and *K.nyangumarta* by the large tegular lobe, the relatively short embolus that does not follow the edge of the bulb, and the twisted distal part of the conductor (Fig. 65A). It can be differentiated from *K.burbidgei* by the median apophysis. In *K.burbidgei*, the median apophysis has a long base and a short branch, and the median apophysis of *K.durrantorum* sp. nov. has a large, wide base and a narrow branch that is nearly the length of the base. Additionally, the spermophor of *K.burbidgei* is undulate, with much of it near the top of the tegular lobe. In *K.durrantorum* sp. nov., the spermophor is U-shaped, reaching the bottom of the tegular lobe medially. *Karaopsburbidgei* is not known to occur on the mainland, only on Barrow and Varanus Islands.

*Karaopsdurrantorum* sp. nov. differs from *K.nyangumarta* in that the former is nearly half the size of the latter. The median apophysis branch is hooked in *K.nyangumarta* and straighter in *K.durrantorum* sp. nov., and the spinules on the median apophysis of *K.nyangumarta* are denser and shorter, covering a larger area than those on the median apophysis the new species. The spermophor of *K.nyangumarta* is very broad, forming a J-shape from the retrolateral side of the tegular lobe to the middle, then is narrowed abruptly (Fig. 63C). In *K.durrantorum* sp. nov., the width of the spermophor is uniform throughout the tegular lobe (Fig. 65A).

##### Description.

**Male** (holotype). Total length 3.24. Carapace: length 1.72, width 2.26. Chelicerae: promargin with three teeth, two nearest fang very close together, smallest closest to fang, other two roughly same size, retromargin with two teeth (1-0-1). Eyes: AER recurved, PER strongly recurved; diameters AME 0.12, ALE 0.08, PME 0.19, PLE 0.27; interdistances AME–PME 0.06, PME–ALE 0.07, ALE–PLE 0.23, PME–PME 0.65, ALE–ALE 0.89, AME–AME 0.34, PLE–PLE 1.12. Sternum: length 0.89, width 1.2. Abdomen: length 1.87, width 1.23. Color (in life Fig. 66A, C/preserved Fig. 64C): Carapace: pale yellowish brown with darker spots, three pair laterally, four spots medially, and two anteriorly between lateral and medial/yellowish white, markings indistinct; setose, with thin, white setae medially and laterally around eye area, red setae around eyes, sparse, darker, shorter, thicker setae present from approximately middle to posterior/after preservation, red setae around eyes and thicker, darker setae remain. Chelicerae: tan, paturon with curved, dark mark frontally (Fig. 66A)/pale whitish yellow, dark marks distinct; cheliceral setae sparser anteriorly and laterally, denser and much stouter on inner cheliceral margin. Maxillae: yellowish white. Labium: dusky, pale distally. Sternum: pale yellowish white. Abdomen: dorsally golden brown, with multi-colored setae, reddish w-shaped mark on anterior half, another posteriorly, posterior third darker overall, longitudinal darker median band, originates anteriorly, extended just past halfway, several small, dark dots along band and posteriorly and laterally/centrally whitish, golden brown anterolaterally, darkened posteriorly, dark spots more distinct as they are from pigment and hair setae have come off in ethanol; ventrally yellowish white. Spinnerets: posterior with red setae dorsally, indistinct in preserved specimen, anterior with dusky markings laterally. Legs: yellowish white, Tr II–IV with black spot prolaterally, Fm leg I–III with a dark spot prolaterally, two dark marks ventrally, other Fm with pale marks basally, and pale annulations distally, Pt with dark area ventrally at Fm-Pt joint, Ti with dark annulation at Pt-Ti joint and pale annulation distally, darker ventrally, Mt with dusky area at Ti-Mt joint and Mt-Ta joint, Ta dusky at tips; spination leg I Fm d 1-1-1, pr 1-1-1, rl 0-0-1, Ti v 2-2-2-2-2, Mt v 2-2-2-2; leg II Fm d 1-1 1, pr 0-0-1, Ti v 2-2-2-2-2, Mt v 2-2-2-2; leg III d 1-1-1, pr 0-0-1, rl 0-1-1, Ti v 2-2, Mt v 2; leg IV Fm d 1-1, pr 0-0-1, rl 0-1-1, Ti 2-2, Mt v 2; leg formula 3241; measurements leg I 8.53 (2.48, 0.97, 2.22. 1.88, 0.98); leg II 10.6 (3.28, 1.02, 2.71, 2.38, 1.21); leg III 11.57 (3.65, 0.99, 2.72, 2.77, 1.44); leg IV 9.85 (3.05, 0.88, 2.37, 2.44, 1.11). Palp: spination Fm d 0-1-2; 1.98 (0.59, 0.30, 0.40, 0.69); dusky area at base of Ti dorsally (Fig. 35C, H); vRTA rounded triangular in retrolateral view, oblong in ventral view, dRTA squared off distally in retrolateral view, slightly curved ventrally, roughly the same size as vRTA; Cy tip with somewhat dense brush of setae (Fig. 66A), Cy triangular, extended slightly further retrobasally in ventral view; rbcp small; C large, twisted, flattened both at tip and retrolaterally, extended dorsally and ventrally (Fig. 35H), heavily sclerotized tip, with CS that covers E and TS that covers CS; E somewhat short, arising from large TL, not following edge of bulb but in prolateral quarter of bulb, extended apically, hooked retrolaterally, beginning ~ 7 o'clock, ending at ~ 12:30 o'clock; MA with broad base, single branch, slightly curved, nearly as long as base, base with long, somewhat sparse Sp along top edge.

**Female**. The female of this species is undescribed, but a specimen has been located in the collection at WAM and will be described in a forthcoming publication.

##### Etymology.

This species is named after Brad and John Durrant, two people who helped me immensely before, during, and after my field work. Name in genitive case.

##### Distribution.

Known from only the Cape Range National Park, Western Australia (Fig. 66C, Map 9A).

##### Natural history.

This species is found in the Cape Range subregion of the Carnarvon bioregion. The climate is semi-desert to subtropical, with summer and winter rainfall. The subregion contains high ecosystem and species diversity, although the subterranean terrestrial invertebrate fauna is poorly surveyed (Kendrick and Mau 2002).

At least two different instars were collected. Molting occurs from every month to every other month, the shortest time being three weeks. According to the data, males are present in late October, when it is dry and starting to become warmer. sel_1135 lived for 1.5 years beyond collection, and although it molted eight times, it did not reach adulthood (Suppl. material 2: tables S1, S20). This species was collected under rocks.

##### Discussion.

The bioregion comprises diverse habitats on Quaternary alluvial, aeolian and marine sediments atop Cretaceous strata. The Cape Range is elevated limestone, with rugged topography and karstic features.

This species appears to be endemic to the area. It is most morphologically similar to *Karaopsburbidgei*, and there were likely connections between the North West Cape Peninsula and Barrow Island to the mainland during times of lower sea level. Molecular data, however, do not indicate that they are each other’s closest relatives (Suppl. material 1).

#### ﻿ Karaops banyjima

Taxon classificationAnimaliaAraneaeSelenopidae

Crews, 2013

C36072AB-5A8A-5854-B3D3-CCC4BE627764

**Figure 67.**
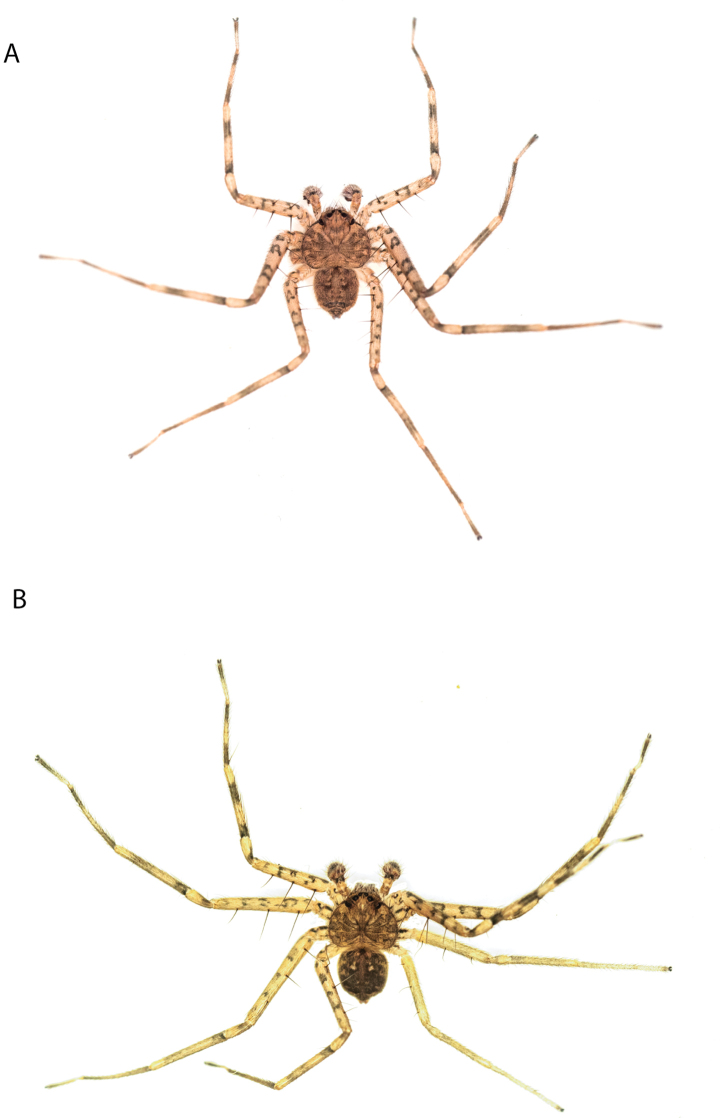
*Karaopsbanyjima***A** adult male, ~ 1.5 km from Hamersley Gorge on side of road (sel_1174, WAM T155550) **B** adult male, Dale’s Gorge, Karijini National Park (sel_1187, WAM T155563).

**Figure 68.**
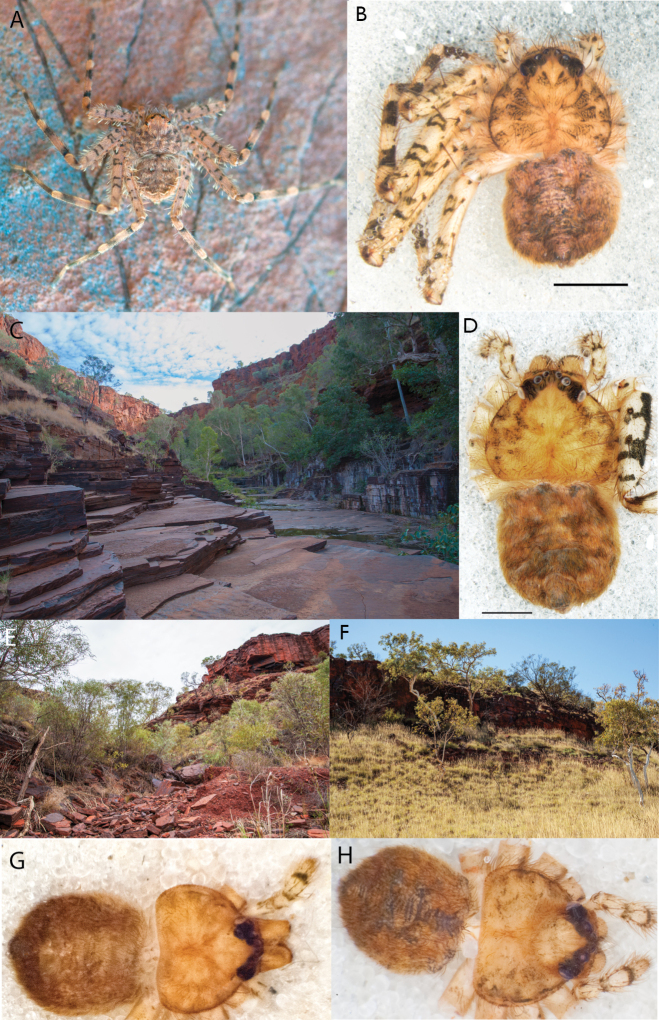
*Karaopsbanyjima* and *Karaopsnyiyaparli* from the Pilbara-Gascoyne species group **A***Karaopsbanyjima*, Dale’s Gorge, Karijini National Park **B***Karaopsnyiyaparli*, adult female, Roy Hill Mine Rail, Bonney Downs (sel_1213, WAM T155589) **C** habitat of *Karaopsbanyjima*, Dale’s Gorge, Karijini National Park **D***Karaopsnyiyaparli*, adult female, Millstream-Chichester National Park (sel_1166, WAM T155542) **E** Hamersley Gorge, habitat of *Karaopsbanyjima***F***Karaopsbanyjima* habitat along Port Hedland-Newman Road **G***Karaopsnyiyaparli*, holotype female, 113 km NNW Newman (WAM T111455) **H***Karaopsnyiyaparli*, paratype female, NW of Mt. Florance Homestead (WAM T125601). Scale bars: 2 mm.

Figs 64B , 65C–F , 66B, E , 67A , 68A, C, E, F , Maps 1 , 9A, B

Karaops banyjima Crews, 2013: 466, figs 33, 34 (♀, examined).

##### Material examined.

Western Australia • 1♂, 1 imm.; ~ 1.5 km from Hamersley Gorge, on side of road; 22°14'5.86"S, 117°58'8.46"E; ~ 571 m; 13 May 2016; S. Crews, J. DeJong leg.; under rocks in dry creek bed; sel_1173–1174; SCC16_029; (WAM T155549, 155550) • 1♂ (reared in captivity), 7 imm.; Karijini National Park, Dale’s Gorge, along trail to Circular Pool by Fortescue Falls sign at bottom of trail to gorge; 22°28'41.18"S, 118°33'43.55"E; ~ 594 m; 14 May 2016; S. Crews, J. DeJong leg.; under rocks, abundant; sel_1183–1190; SCC16_031; (WAM T155559–155566) • 8 imm.; Great Northern Highway (Newman-Port Hedland Road); 23°9'6.73"S, 119°19'46.11"E; ~ 702 m; 15 May 2016; S. Crews, J. DeJong leg.; under rocks on side of road; sel_1198–1205; SCC16_034; (WAM T155574–155581).

##### New records.

Western Australia • 1♂; Yandi (Marillana Creek), ~ 98 km NW of Newman; 22°43'22.2"S, 118°58'30.2"E; 8 Sep. 2014; S. Callan, C. Brooks leg.; foraging; under rocks; (WAM T134063) • 1 imm.; Yandi (Marillana Creek), ~ 98 km NW. of Newman; 22°44'46.1"S, 119°00'30.1"E; 8 Sep. 2014; S. Callan, C. Brooks leg.; foraging; under rocks; (WAM T134072) • 1 imm.; Yandi (Marillana Creek), ~ 98 km NW of Newman; 22°43'33.23"S, 118°59'02.47"E; 14 May 2013; S. Callan leg.; foraging; drainage/ridge-outcrop; (WAM T130404) • 1 imm.; Yandi (Marillana Ck), ~ 98 km NW of Newman; 22°42'39.70"S, 119°00'50.36"E; 14 May 2013; S. Callan, Biologic Env. leg.; foraging; ridge-outcrop; (WAM T130400) • 1 imm.; Yandi (Marillana Ck), ~ 98 km NW of Newman; 22°42'33.08"S, 119°00'15.33"E; 14 May 2013; S. Callan, Biologic Env. leg.; foraging; ridge-outcrop/veg. grove; (WAM T130402) • 1 imm.; Yandi (Marillana Creek), ~ 98 km NW of Newman; 22°42'33.08"S, 119°00'15.33"E; 14 May 2013; S. Callan, Biologic Env. leg. foraging; ridge-outcrop/ veg. grove; (WAM T130403) • 1 imm.; Yandi (Marillana Ck), ~ 98 km NW of Newman; 22°44'23.42"S, 119°00'13.28"E; 14 May 2013; S. Callan, Biologic Env. leg.; foraging; drainage/ridge-outcrop; (WAM T130405) • 1 imm.; Yandi (Marillana Ck), ~ 98 km NW of Newman; 22°46'27.87"S, 119°07'43.67"E; 14 May 2013; S. Callan, Biologic Env. leg.; foraging; ridge-outcrop/ gully; (WAM T130406) • 1 imm.; Yandi (Marillana Ck), ~ 98 km NW of Newman; 22°46'27.87"S, 119°07'43.67"E; 14 May 2013; S. Callan, Biologic Env., leg.; foraging; ridge-outcrop/gully; (WAM T130407) • 1 imm.; Yandi (Marillana Ck), ~ 98 km NW of Newman; 22°46'28.48"S, 119°07'24.24"E; 14 May 2013; S. Callan, Biologic Env., leg.; foraging; ridge-outcrop/gully; (WAM T130409) • 1 imm.; Yandi (Marillana Ck), ~ 98 km NW of Newman; 22°46'33.74"S, 119°07'16.60"E; 14 May 2013; S. Callan, Biologic Env. leg.; foraging; ridge-outcrop/gully; (WAM T130410) • 1 imm.; Yandi (Marillana Ck), ~ 98 km NW of Newman; 22°43'49.65"S, 119°02'49.81"E; 14 May 2013; S. Callan, Biologic Env. leg.; foraging; gully/minor outcrop; (WAM T130411) • 1 imm.; Yandi (Marillana Ck), ~ 98 km NW of Newman; 22°43'49.65"S, 119°02'49.81"E; 14 May 2013; S. Callan, Biologic Env. leg.; foraging; gully/minor outcrop; (WAM T130412) • 1 imm.; Yandi (Marillana Ck), ~ 98 km NW of Newman; 22°43'55.81"S, 119°03'00.10"E; 14 May 2013; S. Callan, Biologic Env. leg.; foraging; drainage/ridge-outcrop; (WAM T130413) • 1 imm.; Yandi (Marillana Ck), ~ 98 km NW of Newman; 22°44'29.32"S, 118°59'34.47"E; 14 May 2013; S. Callan, Biologic Env. leg.; foraging; ridge-outcrop/drainage; (WAM T130415) • 1 imm.; Yandi (Marillana Ck), ~ 98 km NW of Newman; 22°42'50.61"S, 119°01'13.15"E; 14 May 2013; S. Callan, Biologic Env. leg.; foraging; ridge-outcrop; (WAM T130416) • 1♀; Area C, 87.3 km NW of Newman; 22°53'43"S, 119°02'42"E; 17 Feb. 2010; M. Greenham leg.; gully, in soil; (WAM T101159) • 1♀; Karijini National Park, Dales Gorge; 22°28'41"S, 118°33'44"E; 12 Mar. 2015; M.S. Harvey leg.; under rock; (WAM T135465) • 1♀; Karijini National Park, Dales Gorge; 22°28'41"S, 118°33'44"E; 15 Mar. 2015; M.S. Harvey et al. leg.; under rock; (WAM T135466).

##### Diagnosis.

The female (Fig. 66E) is somewhat similar to *Karaopsburbidgei* by the location of the copulatory openings and the general shape of the structures of the endogyne (Crews and Harvey 2011: figs 37, 38). In *K.banyjima*, however, the copulatory openings are located in a depression that is m-shaped along the top margin and w-shaped along the bottom margin (Crews 2013: fig. 33), the ducts connecting the spermathecae and the accessory bulbs are not twisted, and the spermathecae and accessory bulbs are highly sclerotized (Crews 2013: fig. 34).

**Figure 69.**
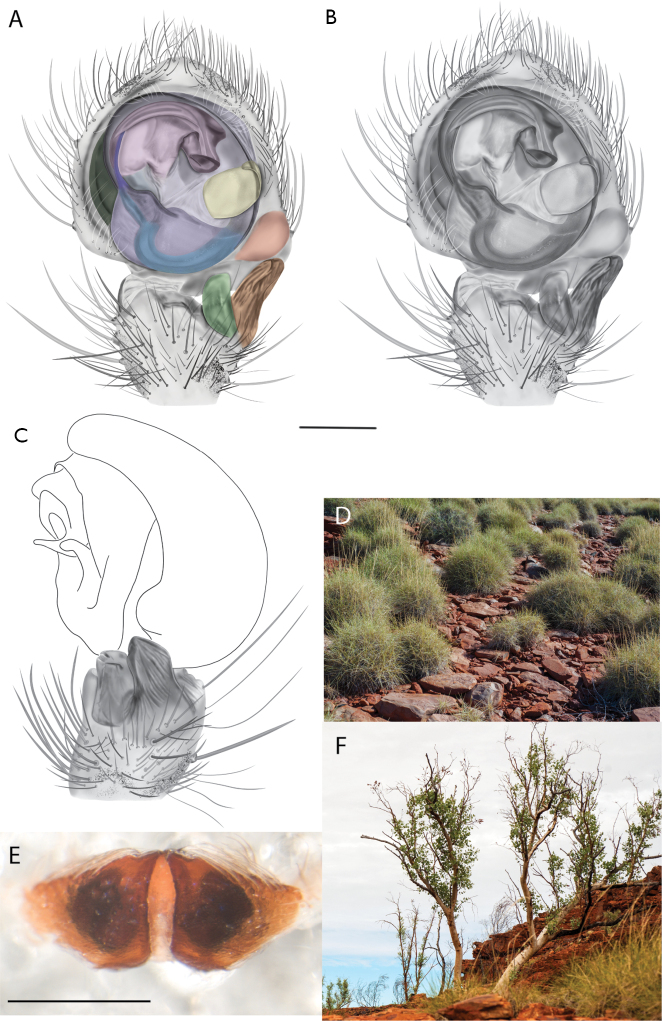
*Karaopsbanyjima* and *Karaopsnyiyaparli* from the Pilbara-Gascoyne species group **A***Karaopsbanyjima*, palp, ventral, Dale’s Gorge, Karijini National Park (sel_1187, WAM T155563); pink = conductor; yellow = MA; light gray-blue = tegulum; light purple = tegular lobe; blue-violet = embolus; blue = spermophor; dark green = subtegulum; salmon = retrobasal cymbial process; green = vRTA; orange = dRTA **B** same, ventral **C** same, retrolateral **D** habitat of *Karaopsnyiyaparli*, Millstream-Chichester, Tom Price-Karratha Road **E***Karaopsnyiyaparli*, epigyne, caudal, Millstream-Chichester National Park (sel_1166, WAM T155542) **F** habitat of *Karaopsnyiyaparli*, Coolawanyah Station. Scale bars: 0.2 mm (**A–C**); 0.5 mm (**E**).

The male is most similar to *Karaopsnyangumarta*, but the two species can be differentiated by the tegular lobe, the median apophysis, the conductor, and the dRTA. In *K.banyjima*, the narrowing of the tegular lobe to the embolus is nearly midway from the basal and distal portions of the bulb (Fig. 69A, B). In *K.nyangumarta*, this point is in the basal third of the bulb (Fig. 63C). The median apophysis of *K.banyjima* has no spinules (Fig. 69A, B), and the median apophysis of *K.nyangumarta* has a dense array of spinules (Fig. 63C). The conductor of *K.banyjima* does not reach the retrolateral edge of the bulb, and the tip is directed laterally (Fig. 69A, B), whereas in *K.nyangumarta*, the conductor reaches the retrolateral side of the bulb, and the tip of the conductor is directed ventrally (Fig. 63C). The dRTA of *K.banyjima* is rather short and stout (but not like a little teapot) (Fig. 69A, B), whereas that of *K.nyangumarta* is longer, narrower, and curved slightly ventrally (Fig. 63C, D). It is somewhat difficult to distinguish the two in life by the abdominal pattern, but *K.nyangumarta* males have more mottling dorsally on the abdomen.

##### Description.

**Figure 70.**
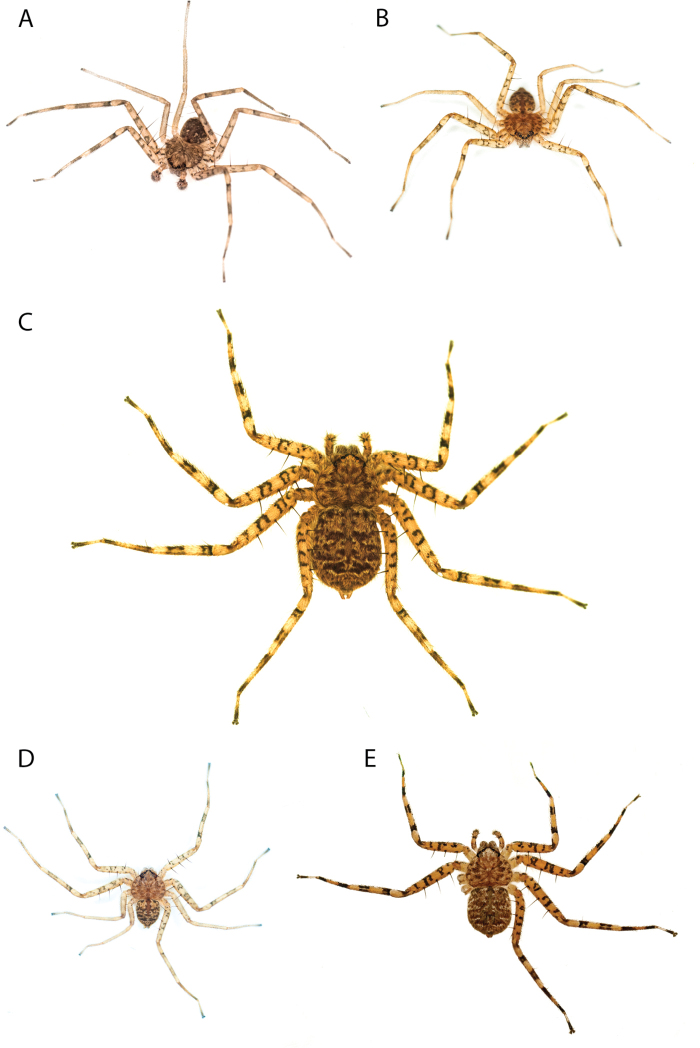
*Karaopsbanyjima* and *Karaopsnyiyaparli* from the Pilbara-Gascoyne species group **A***Karaopsbanyjima*, adult male, Dale’s Gorge, Karijini National Park (sel_1187, WAM T155563) **B***Karaopsnyiyaparli* adult male (tore palps off when molting), Coolawanyah Station (sel_1171, T155547) **C***Karaopsnyiyaparli*, adult female, Roy Hill Mine Rail, Bonney Downs (sel_1213, WAM T155589) **D***Karaopsnyiyaparli*, adult male (tore palps off when molting), Coolawanyah Station (sel_1171, T155547) **E***Karaopsnyiyaparli*, adult female, Millstream-Chichester National Park (sel_1166, WAM T155542).

**Male** (sel_1174, WAM T155550). Total length 4.77. Carapace: length 2.56, width 3.16. Chelicerae: promargin with three teeth, the two closest to the fang are very close together, smallest closest to fang, other two larger, same retromargin with two teeth (1-0-1). Eyes: AER nearly recurved; PER strongly recurved; diameters AME 0.19, ALE 0.12, PME 0.23, PLE 0.37; interdistances AME–PME 0.04, PME–ALE 0.13, ALE–PLE 0.20, ALE–ALE 1.33, AME–AME 0.44, PLE–PLE 1.6. Sternum: length 1.24, width 1.67. Abdomen: length 2.21, width 2.10. Color (in life Figs 67A, B, 68A, 70A/preserved Figs 64B, D, 65F, 66B): Carapace: dusky markings on tan background, in nature, pale yellowish brown with darker markings, orange around eye area/markings mostly faded, still visible laterally and just behind eyes, yellowish white. Chelicerae: yellowish white with a small spot below clypeus, paturon with curved, dark mark frontally (Fig. 66B), with more setae anteriorly than laterally, especially inner margin. Maxillae: whitish. Labium: dusky, pale distally. Sternum: yellowish white. Abdomen: dorsally setose, dark tan to brown, paler medially until posterior third, anteriorly with pale spot on either side of paler median area, dark horizontal band near posterior of abdomen, posterior of dark band with pale setae making somewhat w-shaped mark; in nature, at least in juveniles, anterior pale spots and w-shaped mark are easily discernable (Fig. 68A)/most paler areas very pale to white, darker areas very dark, with no particular pattern or shape visible, orangish setae around margin; ventrally yellowish white. Spinnerets: with dusky markings. Legs: pale yellowish to tan, dusky ventrally, Cx, Tr with dark prolateral spot, Fm with marks that do not encircle the legs, unpigmented centrally, Pt with dark annulation basally, Ti with two annulations, one basal and the other distal though not at joint with Mt, Mt with two annulations, one basal, one distal, Ta tip dark/markings quite faded; juveniles with Fm setal tufts of flat, white setae; spination leg I Fm d 1-1-1, pl 1-1-1, rl 0-0-1, Ti v 2-2-2-2-2, rl 1-1, pr 1-1, Mt v 2-2-2-2; leg II Fm d 1-1-1, pr 0-0-1, rl 0-0-1, Ti v 2-2-2-2-2-2, pr 0-1, rl 0-1, Mt v 2-2-2-2, pr 0-1-0; leg III Fm d 1-1-1, pr 0-0-1, rl 0-1-1, Ti v 2-2, Mt v 2-2; leg IV Fm d 1-1-1, pr 0-1-1, rl 0-1-1, Ti v 2-2, pr 1-1, rl 1-1, Mt v 2-1; leg formula 3421; measurements leg I 11.10 (3.08, 1.33, 3.10, 2.22, 1.37); leg II 12.43 (3.26, 1.47, 3.55, 2.69, 1.46); leg III 14.23 (3.81, 1.18, 3.80, 3.40, 2.04); leg IV 12.54 (3.76, 1.08, 3.19, 3.02, 1.49). Palp: spination Fm d 0-1-2; 2.60 (0.63, 0.46, 0.51, 1.00); Ti dark dorsally, with marking extended to both pro- and retrolateral sides, basal half of Cy with dark marks dorsally (Figs 64D, 65D, E, 69A–C) both vRTA and dRTA short, striae present on dRTA; rbcp large; Cy round triangular, extended retrobasally (Fig. 69C); C large but mostly restricted to medioapical part of bulb, TS covering part of C, CS covering ~ 1/2 of E, medially unsclerotized, sclerotized more distally, tip very sclerotized, ventrally projecting arch at point where unsclerotized area comes into contact with more sclerotized area, sclerotized projection directed retrolaterally, narrowed to flattened, pointed tip directed ventrally, groove along extended distal part; E short, arising from very large TL at ~ 9 o’clock, hooked, ending at ~ 1 o’clock; MA broad throughout, only slightly narrowing distally, not very sclerotized, distal portion gently curled.

**Female.** The description of the female can be found in Crews (2013).

##### Variation.

Additional males were measured and size ranges from 4.01–4.77. Specimen sel_1187 was smaller overall, with smaller features in general and also darker than sel_1174.

##### Distribution.

Known from only the Hamersley subregion of the Pilbara, Western Australia (Fig. 68C, E, F, Map 9A, B).

##### Natural history.

Males have matured in captivity and in nature in September and October, a dry time, transitioning from cool to hot. Females have been collected in February and March, a hot, wet time of the year (Suppl. material 2: tables S1, S21).

##### Discussion.

The palps of sel_1198 (WAM T155574) were stuck in the exuvia when molting, but the species could be determined by the RTA and molecular data (Fig. 65C–E). The distribution overlaps that of *Karaopsnyangumarta*, is close to that of *K.martamarta*, and perhaps overlaps with *K.morganoconnelli* sp. nov. However, none of the different species examined are known to have ever been taken from the same place at the same time.

#### ﻿ Karaops ngarluma

Taxon classificationAnimaliaAraneaeSelenopidae

Crews, 2013

D1F1A5EA-600F-5A2E-AF5E-1B6FB4E02102

Fig. 66G , Maps 1 , 9A, B

Karaops ngarluma Crews, 2013: 459, figs 23, 24 (♂, examined).

##### Additional probable record

(see discussion below). Western Australia • 6 imm.; Harding Dam, S of Roebourne; 20°58.621'S, 117°16.238'E; 12 May 2016; ~ 57 m; S. Crews, J. DeJong leg.; under rocks; rocky hill; sel_1153–1158; SCC16_023; (WAM T155529–155534).

##### Diagnosis.

*Karaopsngarluma* is most similar to *K.forteyi* by the long, narrow, pointed dRTA. They can be differentiated by the indentation along the apical margin of the tegular lobe in *K.ngarluma* (Crews 2013: figs 17, 18, 23, 24).

##### Description.

The description of the male can be found in Crews (2013).

**Female.** Unknown.

##### Distribution.

This species is known only from two nearby localities in the northeastern Pilbara, Western Australia (Map 9A, B).

##### Natural history.

This species was collected in pitfall traps left for 15 months, so it is unclear when adults are present.

##### Discussion.

*Karaopsngarluma* is only known from four males (Fig. 66G), all of which were collected in pitfall traps left out for more than a year or up to 15 months. They occur in the extremely *Karaops* species rich Chichester subregion of the Pilbara bioregion. Although the female is unknown, there are no unpaired females nearby. Molecular data indicate that juvenile specimens from Harding Dam may be *K.ngarluma* (Fig. 64A) (Suppl. material 1). This record is provided above. (Suppl. material 2: table S1).

#### ﻿ Karaops yurlburr

Taxon classificationAnimaliaAraneaeSelenopidae

Crews, 2013

159F3E22-F681-5F22-827B-31C96AEFD151

Figs 66D, F , 72D , Maps 1 , 9A, B

Karaops yurlburr Crews, 2013: 452, figs 9–12 (♀♂, examined).

##### Additional probable record

(see discussion below). Western Australia • 2 imm.; Millstream-Chichester National Park, Narrina Pool on Narrina Creek; 21°20'45"S, 117°16'08"E; 25 Mar. 2015; M.S. Harvey, J. Huey, R. Teale leg.; under rock; (WAM T135803–T135804).

##### Diagnosis.

The female is similar to *Karaopsnyiyaparli* and *K.kariyarra* by the copulatory openings located in a depression in the median field of the epigynal plate. In *K.yurlburr*, though, the depression is very large and more than half as wide as the epigynal plate (Crews 2013: figs 5, 7, 9).

The male of this species is similar to others in the Chichester region, but the keeled edge between the dRTA and vRTA is different from any of the others. There is also a very small indentation along the upper margin of the tegular lobe, and the conductor is unique (Crews 2013: figs 9–12).

##### Description.

The male and female are described in Crews (2013).

##### Distribution.

This species is known only from the type locality, vic. Python Pool, Pilbara, Western Australia (Fig. 72D).

**Figure 71.**
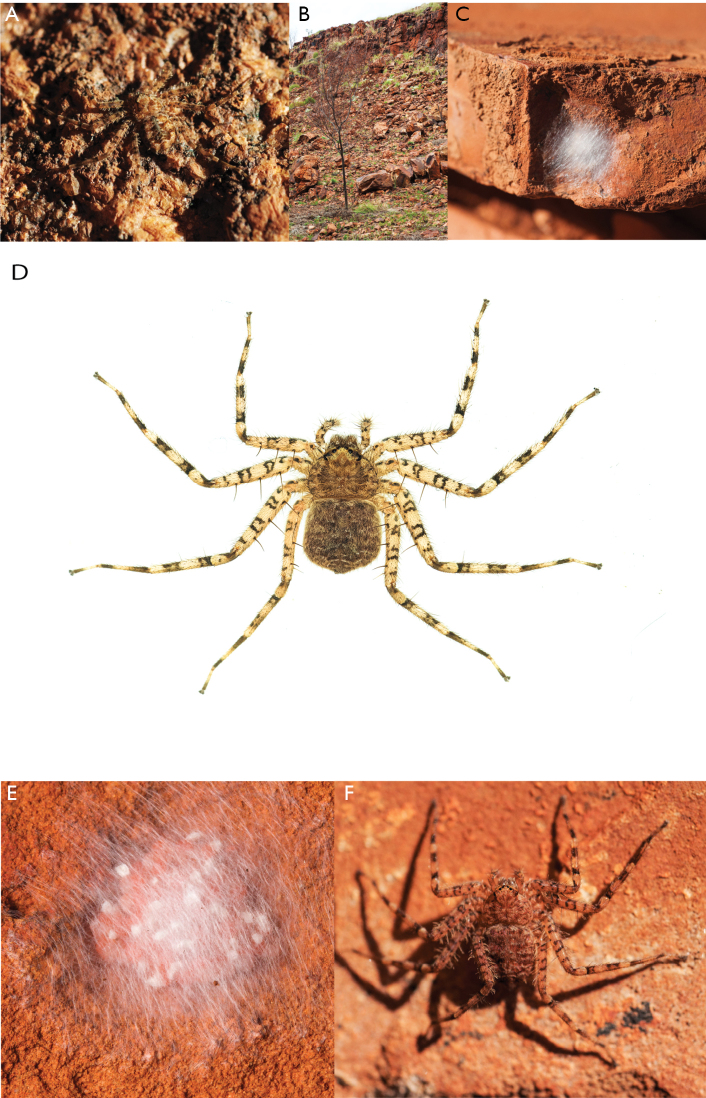
*Karaopsnyiyaparli***A** camouflaged on a rock, BHP Rail Line **B** habitat, Coolawanyah Station **C** egg sac, Roy Hill Mine Rail, Bonney Downs **D** adult female, Roy Hill Mine Rail, Bonney Downs (sel_1213, WAM T155589) **E** egg sac with spiderlings, Millstream-Chichester National Park (from sel_1166, WAM T155542) **F** adult female, Millstream-Chichester National Park.

**Figure 72.**
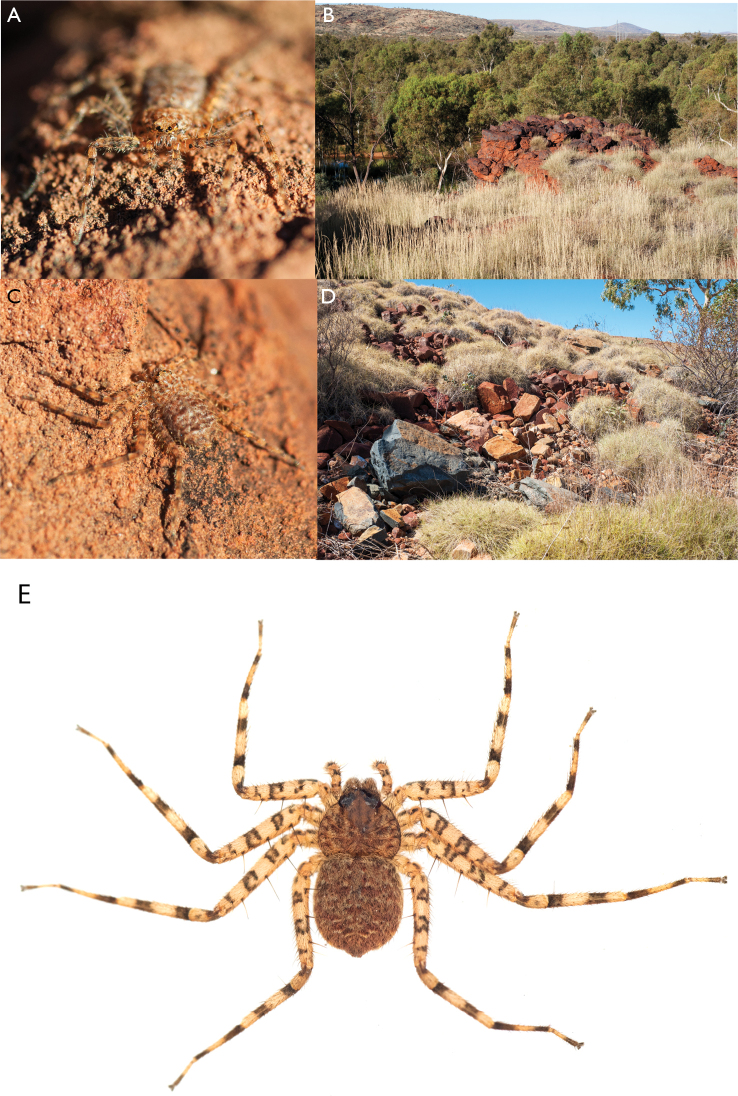
*Karaopsnyiyaparli* and *Karaopsyurlburr* from the Pilbara-Gascoyne species group **A***Karaopsnyiyaparli*, BHP Rail Line **B** habitat of *Karaopsnyiyaparli*, Bonney Downs Station **C***Karaopsnyiyaparli*, BHP Rail Line (Photo: J. DeJong) **D** habitat of *Karaopsyurlburr*, Python Pool, Millstream-Chichester National Park **E***Karaopsnyiyaparli*, BHP Rail Line (sel_1230, WAM T155606).

##### Natural history.

*Karaopsyurlburr* (Fig. 66D, F) is known from a single female and male specimen, two penultimate males, and five immatures. These were all collected in ethylene glycol pitfalls left for a 15-month period, thus it is impossible to know when adults are present. Python Pool and Narrina Creek are both found in the Chichester subregion of the Pilbara bioregion; this subregion is the most species rich region for *Karaops* (Suppl. material 2: table S1).

##### Discussion.

Despite searching the same locality as well as nearby localities, only *Karaopsnyiyaparli* was collected. The female and male of *K.yurlburr* are significantly different from *K.nyiyaparli*, so there is no chance of confusing them. Based on molecular data (Suppl. material 1), two immatures collected at nearby Narrina Pool on Narrina Creek may be *K.yurlburr*, and this record is provided above.

#### ﻿ Karaops kariyarra

Taxon classificationAnimaliaAraneaeSelenopidae

Crews, 2013

C6E89A76-CA27-5071-96EF-D86EE9149EF6

**Figure 73.**
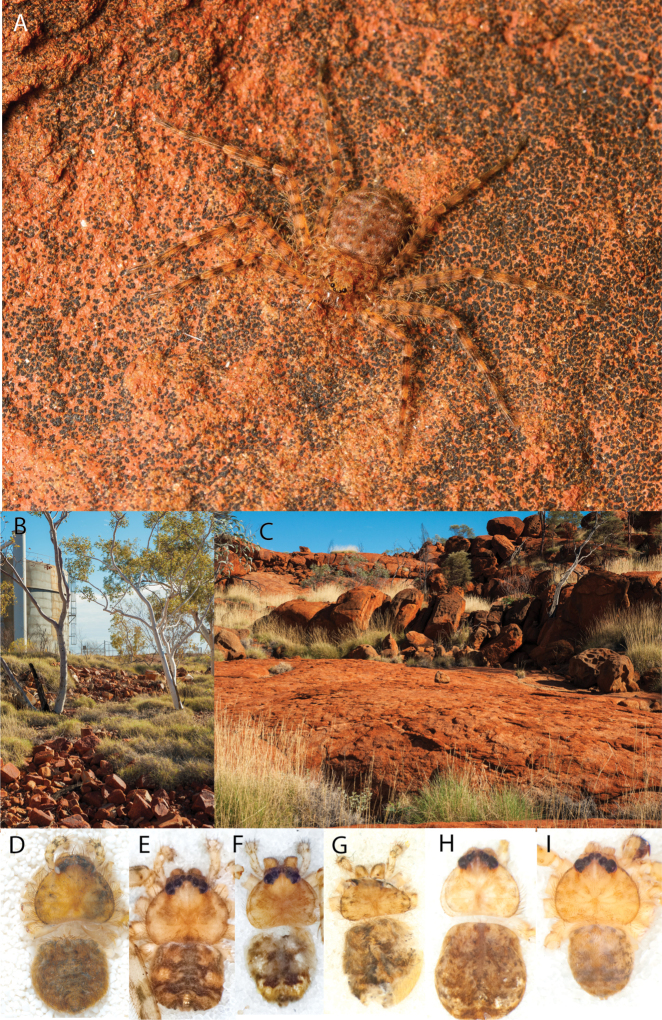
Members of the Pilbara-Gascoyne species group **A***Karaopsnyiyaparli*, BHP Rail Line (sel_1230, WAM T155606) (Photo: J. DeJong) **B** habitat of *Karaopsnyiyaparli*, Millstream-Chichester National Park **C** habitat of *Karaopsnyiyaparli* along BHP Rail Line **D***Karaopsnyiyaparli*, holotype female(WAM T111455) **E***Karaopskariyarra*, holotype female, Wodgina Mine (WAM T105208) **F***Karaopskariyarra*, adult female, 55 km S of Port Hedland (WAM T106657) **G***Karaopsfeedtime*, holotype female, NNW Newman (WAM T111456) **H***Karaopsforteyi*, holotype female, Cowra Line Camp (WAM T79407) **I** same, paratype male, Cowra Line Camp (WAM T79408).

Fig. 73E , Maps 1 , 9A, B

Karaops kariyarra Crews, 2013: 451, figs 7, 8 (♀, examined).

##### Diagnosis.

The female is most similar to *Karaopsnyiyaparli* in that the copulatory ducts are located in a depression of the median field of the epigyne, and the lateral lobes are easy to distinguish (Crews 2013: figs 5, 7). The endogynes are also similar in that the spermathecae are dumbbell shaped, and the accessory bulbs (erroneously labeled as spermathecae in Crews (2013)) are long and thin. They can be differentiated by the shape of the depression on the endogyne. In *K.nyiyaparli*, it is oval and in *K.kariyarra* it is heart shaped. The lateral lobes of *K.kariyarra* are pointed at the basomedial margin, and they are not in *K.nyiyaparli*. The accessory bulbs are extremely tiny in *K.kariyarra* (Crews 2013: figs 6, 8).

##### Description.

The female is described in in Crews (2013).

**Male.** Unknown.

##### Distribution.

This species is known only from two nearby localities in the Chichester subregion of the Pilbara, Western Australia (Map 9A, B).

##### Natural history.

Late March to early April are hot and wet times, transitioning to drier in April. The adult females of several species from the Chichester region have been found in the hotter, wetter times of the year (Suppl. material 2: table S1).

##### Discussion.

*Karaopskariyarra* is only known from two female specimens collected in two separate pitfall traps left out for ~ 10 days in late March–early April. This species is similar to and overlaps with *K.nyiyaparli*, but both specimens of *K.kariyarra* are exactly the same despite being collected at different areas, and these two are both different from *K.nyiyaparli* which is widespread; thus, it does not appear to be a variant of *K.nyiyaparli*. No molecular data from these specimens were available.

#### ﻿ Karaops feedtime

Taxon classificationAnimaliaAraneaeSelenopidae

Crews, 2013

18A2A5E4-C564-5B32-9615-AD0F2F3B1183

Fig. 73G , Maps 1 , 9A, B

Karaops feedtime Crews, 2013: 454, figs 13, 14 (♀, examined).

##### Diagnosis.

This species is unique from all other species in the Pilbara/Gascoyne group by having a long median lobe that isn’t as strongly sclerotized posteriorly as the rest of the lobe. Additionally, it has asymmetrical, long ducts with several curves. The accessory bulb is erroneously labeled as a spermatheca in fig. 14 of Crews (2013). The spermathecae are very long, with small bulbs on either end. The accessory bulbs are on the copulatory duct, with a small duct and a small bulb at each end of the spermathecae (Crews 2013: figs 13, 14).

##### Description.

The description of the female can be found in Crews (2013).

**Male.** Unknown.

##### Distribution.

Known only from the type locality on the border of the Chichester and Fortescue subregions of the Pilbara, Western Australia (Map 9A, B).

##### Natural history.

*Karaopsfeedtime* occurs in the Fortescue subregion of the Pilbara bioregion, close to the border of the Chichester subregion, around an area that has been altered extensively by mining operations. In the central part of the region where the specimen was collected, the habitat is bunch grass and short grass communities on and around mesas (Kendrick 2001b). The climate is semi-desert tropical, with rainfall occurring mostly during summer cyclone events. This species may be an SRE. March is quite a bit wetter than April and because this species was collected in a pitfall trap over both months, it is difficult to determine the climate at the time. The Fortescue subregion is conspicuously empty of *Karaops* (Map 9B). This could be because there are more plains and grasses and less rocky areas or less areas to survey because of a lack of mining operations in the subregion (Suppl. material 2: table S1).

##### Discussion.

*Karaopsfeedtime* is known from a single specimen that was collected in a pitfall trap that was left out from late March to late April. It is one of the many species known from a single specimen that falls within the range of the widespread *K.nyiyaparli*, which was the only species that was collected on a recent field trip to the type locality of *K.feedtime*. *Karaopsfeedtime* and *K.nyiyaparli* are not morphologically similar in genitalic characteristics. The type is damaged (Fig. 73G).

#### ﻿ Karaops forteyi

Taxon classificationAnimaliaAraneaeSelenopidae

Crews, 2013

C17594E2-D1A2-5A5F-93F5-22FB37DE7360

Fig. 73H, I , Maps 1 , 9A, B

Karaops forteyi Crews, 2013: 455, figs 15–18 (♂♀, examined).

##### Diagnosis.

The female of *Karaopsforteyi* is not similar to any species in the Pilbara by the epigyne. The endogyne is somewhat similar to that of *K.feedtime*. It has long, skinny, ducts with several turns. The spermathecae are very long, with either end being more oval than those in *K.feedtime*. The accessory bulbs are mislabeled as the spermathecae in Crews (2013). The accessory bulbs arise from the copulatory ducts and are long and narrow, similar to the spermathecae. Unlike the epigyne of *K.feedtime*, there is no median lobe but rather a circular area where the copulatory openings are located laterally. The posterior margin of the epigyne is also indented (Crews 2013: figs 13–16).

In the male of *Karaopsforteyi*, the dRTA is much longer than the vRTA, and there is no keel or ridge as in *K.yurlburr* or *K.ngarluma*. The conductor is similar to that of *K.nyiyaparli*, but in *K.forteyi*, the embolus follows the margin of the cymbium, whereas it is more toward the middle of the bulb in *K.nyiyaparli*.

##### Description.

The description of the male and female can be found in Crews (2013).

##### Distribution.

This species is known only from two localities separated by a couple of kilometers (Map 9A, B).

##### Natural history.

This species was collected in the Chichester subregion which contains more species than all other subregions (Suppl. material 2: table S1).

##### Discussion.

*Karaopsforteyi* is known from only two specimens, a male and a female, paired together because at the time of their description it was unknown that they occurred within or nearby the range of multiple other species, and they were obtained fairly close together in pitfalls at the same time. The pitfall traps were left for more than a year, so there is no information on when adults may be found, and the types are faded and missing setae (Fig. 73H, I). Collecting around the area for a few hours only produced *K.nyiyaparli*. This species is quite different from *K.nyiyaparli*, so there is no chance of it being a variant. Only two specimens were recovered in traps that were left for more than a year, so this species is thought to be quite rare.

## Supplementary Material

XML Treatment for Selenops

XML Treatment for Karaops

XML Treatment for Karaops ngarutjaranya

XML Treatment for Karaops pilkingtoni

XML Treatment for Karaops vadlaadambara

XML Treatment for Karaops manaayn

XML Treatment for Karaops deserticola

XML Treatment for Karaops kwartatuma

XML Treatment for Karaops larapinta

XML Treatment for Karaops mparntwe

XML Treatment for Karaops strayamate

XML Treatment for Karaops gangarie

XML Treatment for Karaops ellenae

XML Treatment for Karaops monteithi

XML Treatment for Karaops raveni

XML Treatment for Karaops jarrit

XML Treatment for Karaops marrayagong

XML Treatment for Karaops dawara

XML Treatment for Karaops nitmiluk

XML Treatment for Karaops jawayway

XML Treatment for Karaops yumbu

XML Treatment for Karaops francesae

XML Treatment for Karaops toolbrunup

XML Treatment for Karaops keithlongbottomi

XML Treatment for Karaops dejongi

XML Treatment for Karaops umiida

XML Treatment for Karaops dalmanyi

XML Treatment for Karaops jenniferae

XML Treatment for Karaops alanlongbottomi

XML Treatment for Karaops conilurus

XML Treatment for Karaops malumbu

XML Treatment for Karaops larryoo

XML Treatment for Karaops garyodwyeri

XML Treatment for Karaops yumbubaarnji

XML Treatment for Karaops kennerleyorum

XML Treatment for Karaops madhawundu

XML Treatment for Karaops mareeba

XML Treatment for Karaops markharveyi

XML Treatment for Karaops burbidgei

XML Treatment for Karaops martamarta

XML Treatment for Karaops julianneae

XML Treatment for Karaops badgeradda

XML Treatment for Karaops joehaeneri

XML Treatment for Karaops karrawarla

XML Treatment for Karaops morganoconnelli

XML Treatment for Karaops nyamal

XML Treatment for Karaops nyangumarta

XML Treatment for Karaops jaburrara

XML Treatment for Karaops nyiyaparli

XML Treatment for Karaops durrantorum

XML Treatment for Karaops banyjima

XML Treatment for Karaops ngarluma

XML Treatment for Karaops yurlburr

XML Treatment for Karaops kariyarra

XML Treatment for Karaops feedtime

XML Treatment for Karaops forteyi

## Footnotes

Cheliceral teeth in Karaops raveni are known to vary, and the sample sizes for K. marrayagong and K. jarrit are small; however, when the number of K. raveni teeth is variable, it is typically the retromarginal teeth and not the promarginal teeth that vary.

## Supplementary materials

Supplementary material 1 Tree from the IQTree full dataset (CO1, H3, 16S) analysis Data type .eps file Explanation note Tree from the IQTree full dataset (CO1, H3, 16S) analysis. Orange = Central Desert species group; purple = Kimberley species group; blue = strayamate species group; green = dawara species group; yellow = francesae species group; red = raveni species group; teal = Pilbara-Gascoyne species group. The lineages without a color are not assigned to a species group. https://binary.pensoft.net/file/816463This dataset is made available under the Open Database License (http://opendatacommons.org/licenses/odbl/1.0/). The Open Database License (ODbL) is a license agreement intended to allow users to freely share, modify, and use this Dataset while maintaining this same freedom for others, provided that the original source and author(s) are credited.Sarah C. Crews

Supplementary material 2 These data provide information related to the possible times of the year that adults of a species may be found in a particular region or subregion as determined by the IBRA Data type tables (word file) Explanation note Information related to the possible times of the year that adults of a species may be found in a particular region or subregion as determined by the IBRA. *Karaopsnitmiluk* sp. nov., *Karaopsjawayway* sp. nov., *Karaopsyumbu* – Tanami, *Karaopsyumbu* – O Victoria Plain, *Karaopskeithlongbottomi*, *Karaopsdejongi* sp. nov., *Karaopsdalmanyi* sp. nov., *Karaopsjenniferae*, *Karaopsmalumbu* sp. nov., *Karaopsgaryodwyeri* sp. nov., *Karaopsyumbubaarnji* sp. nov., *Karaopskennerleyorum* sp. nov., *Karaopsmarkharveyi* sp. nov., *Karaopsbadgeradda*, *Karaopsjoehaeneri* sp. nov., *Karaopsmorganoconnelli* sp. nov., *Karaopsnyangumarta* sp. nov., *Karaopsnyiyaparli*, *Karaopsdurrantorum* sp. nov., *Karaopsbanyjima*. https://binary.pensoft.net/file/816464This dataset is made available under the Open Database License (http://opendatacommons.org/licenses/odbl/1.0/). The Open Database License (ODbL) is a license agreement intended to allow users to freely share, modify, and use this Dataset while maintaining this same freedom for others, provided that the original source and author(s) are credited.Sarah C. Crews
