# Supplementary material for: ﻿But wait, there’s more! Descriptions of new species and undescribed sexes of flattie spiders (Araneae, Selenopidae, Karaops) from Australia
Source: Zookeys. 2023 Feb 27;1150:1–189. doi: 10.3897/zookeys.1150.93760 (PMC10208737; doi:10.3897/zookeys.1150.93760)
Supplement: Supplementary material 2 — These data provide information related to the possible times of the year that adults of a species may be found in a particular region or subregion as determined by the IBRA [file zookeys-1150-001_article-93760__-s002.docx]

**Supplementary Material**

**Table 1.** These data provide information related to the possible times of the year that adults of a species may be found in a particular region or subregion as determined by the IBRA.

| **Species** | **Sex** | **Bioregion** | **Subregion** | **Time of year** | **Climate** |
| --- | --- | --- | --- | --- | --- |
| *K. ngarutjaranya* | ♂♀ | Central Ranges | Mann-Musgrave Block | Oct | Hot, wetter |
| *K. pilkingtoni* | ♂♀ | MacDonnell Ranges | Hartz Range | ♂ May  ♀ Jun | Cooler, drier |
| *K. vadlaadambara* | ♂♀ | Flinders Lofty Block | Northern Flinders, Central Flinders | ♂♀ May  ♂ Oct | Cooler, slightly wet  Warm, wet |
| *K. manaayn* | ♂♀ | New South Wales North Coast | Macleay Hastings,  Coffs Coast and Escarpment | ♂♀ Apr  ♀ Nov | Temperate |
| *K. deserticola* | ♀ | Central Ranges | Watarru | Aug | Cooler, drier |
| *K. kwartatuma* sp. nov. | ♂ | MacDonnell Ranges | MacDonnell Ranges | Apr | Cooler, drier, increased humidity |
| *K. larapinta* sp. nov. | ♀ | Finke | Finke or Henbury | Oct | Hot, wet |
| *K. mparntwe* sp. nov. | ♀ | - | - | Aug | Cooler, drier |
| *K. strayamate* sp. nov. | ♂♀ | Brigalow Belt North; Central Mackay Coast | Bogie River Hills, Marlborough Plains; Whitsunday | ♀ Jun,  ♂♀ Jun, Jul; ♂ May | ♀ Cooler, drier  ♂ Both coolest and warmest, drier |
| *K. gangarie* | ♂♀ | Wet Tropics; Cape York Peninsula | Daintree-Bloomfield; Starke Coastal Lowlands | ♀♂ May; ♀ January | Cooler, drier; Hot, trans. from dry to wet |
| *K. ellenae* | ♂♀ | Jarrah; Swan Coastal Plain; Warren; Avon Wheatbelt | Northern Jarrah; Perth; Warren; Katanning | ♀♂ all months but Apr and May | All except the hottest time of year but may be due to collecting bias. |
| *K. monteithi* | ♀ | Cape York Peninsula | Coen-Yambo Inlier | May, Jun | Hot, trans. to cool, dry |
| *K. raveni* | ♂♀ | multiple | multiple | QLD: Jun–Jan; NSW: Nov–Jul | Humid subtropical; temperate |
| *K. jarrit* | ♂♀ | Jarrah Forest; Swan Coastal Plain; (Esperance Plains; Coolgardie | Northern Jarrah Forest; Perth; (Fitzgerald; Southern Cross) | ♂ Jun, Nov, Dec;  ♀Dec | Warm trans. to hot; drier |
| *K. marrayagong* | ♂♀ | Sydney Basin | Pittwater | Apr | Temperate |
| *K. dawara* | ♀ | Darwin Coastal | Darwin Coastal | Nov, Jan | Usually hot; former trans. to wet, latter wettest |
| *K. nitmiluk* sp. nov. | ♀ | Pine Creek | Pine Creek | Col. as imm. in Jun; adult ♀ Oct, Dec | Tropical monsoonal, hot, wet |
| *K. jawayway* sp. nov. | ♀ | Gulf Fall and Uplands | McArthur | Col. as imm. in Jun; adult ♀ Feb | Hot, wet |
| *K. yumbu* | ♂ | Tanami; Ord Victoria Plain | Tanami; Purnululu | Jan–Mar (pitfall); Oct, Dec | Hot, wet |
| *K. francesae* | ♂♀ | Jarrah Forest; Esperance Plains | Southern Jarrah Forest; Fitzgerald, Recherche | Feb–Jun, Oct, Nov | Trans. from hot and dry to cool and wet (could be due to collection bias) |
| *K. toolbrunup* | ♂♀ | Esperance | Fitzgerald | Feb, Apr | Trans. from hot and dry to hot and wet |
| *K. keithlongbottomi* | ♂ | Northern Kimberley | Mitchell | Jun | Cooler, drier |
| *K. dejongi* sp. nov. | ♀♂ | Dampierland | Fitzroy Trough | Jun, Sep | Cool, dry, trans. to hot |
| *K. umiida* | ♀ | Northern Kimberley | Mitchell | Jul | Cool, dry |
| *K. dalmanyi* sp. nov. | ♀♂ | Central Kimberley | Pentecost | Jun–Sep | Cool, dry |
| *K. jenniferae* | ♀ | Central Kimberley | Mount Eliza | Jul | Cool, dry |
| *K. alanlongbottomi* | ♂ | Northern Kimberley | Mitchell | Jul | Dry |
| *K. conilurus* sp. nov. | ♂ | Northern Kimberley | Mitchell | Jul | Cool, dry |
| *K. malumbu* sp. nov. | ♀♂ | Victoria Bonaparte | Victoria Bonaparte I | ♀ Oct, ♂ Sep | ♀ Hot, dry  ♂ trans. from cool, dry to hot, dry |
| *K. larryoo* | ♂ | Northern Kimberley | Berkeley, very close to Mitchell | Jun | Cool, dry |
| *K. garyodwyeri* sp. nov. | ♂ | Central Kimberley | Pentecost | Jun | Cool, dry |
| *K. yumbubaarnji* sp. nov. | ♀♂ | Ord Victoria Plains | Purnululu | ♀ Oct, ♂ Nov | ♀ Trans. from cool to warmer, dry; hot, trans. from dry to wet |
| *K. kennerleyorum* sp. nov. | ♂ | Gulf Fall and Uplands | McArthur | Oct | Hot, wet |
| *K. madhawundu* sp. nov. | ♀ | Einasleigh Uplands | Kidston | Sep | Cool, dry |
| *K. mareeba* sp. nov. | ♀ | Einasleigh Uplands | Hodgkinson Basin | Sep, Oct | Cool, dry |
| *K. markharveyi* sp. nov. | ♀♂ | Central Kimberley, Northern Kimberley, Victoria Bonaparte | Pentecost, Mitchell, Keep | ♀ Dec–Feb, ♂ Oct, Nov | Hot, wet |
| *Karaops burbidgei* | ♂♀ | Pilbara | Roebourne | Apr, Nov, Dec | Hot, wet and cool dry |
| *Karaops martamarta* | ♀♂ | Pilbara | Hamersley, Chichester (rare) | Mar–June, Aug–Nov | All except hot, wet |
| *Karaops julianneae* | ♀ | Gascoyne | Carnegie | Apr, Nov | trans. to cool, dry; dry trans. to warm, wet |
| *Karaops badgeradda* | ♀ | Murchison | West Murchison | Mar, May | Hot, wet; cool dry |
| *Karaops joehaeneri* sp. nov. | ♀♂ | Carnarvon | Wooramel | ♀ May, ♂ Nov | Cool, wet; hot, dry |
| *Karaops karrawarla* | ♀♂ | Carnarvon | Wooramel | Jan–May - pitfall | unclear |
| *K. morganoconnelli* sp. nov. | ♀♂ | Pilbara | Hamersley | ♀ Apr, May; ♂ Dec | Trans. to cool, wet; hot, dry |
| *Karaops nyamal* | ♀ | Pilbara | Chichester | Mar–May (pitfall) | Trans. to cool, dry from hot, wet |
| *Karaops nyangumarta* | ♀♂ | Pilbara | Hamersley | Aug–Oct | Cool, dry, trans. to warm, dry |
| *Karaops jaburrara* | ♂ | Pilbara | Chichester | unknown | unknown |
| *Karaops nyiyaparli* | ♀♂ | Pilbara | Chichester | ♀ March–May; ♂ March | trans. from hot, wet to cool, dry |
| *Karaops yurlburr* | ♀♂ | Pilbara | Chichester | unknown (pitfall) | unknown |
| *Karaops durrantorum* sp. nov. | ♂ | Carnarvon | Cape Range | Oct | Trans. to warm, dry |
| *Karaops banyjima* | ♀♂ | Pilbara | Hamersley | ♀ Feb, Mar; ♂ Sep, Oct | hot, wet; trans. from cool to hot, dry |
| *Karaops ngarluma* | ♂ | Pilbara | Chichester | unknown (no adults collected in May) | unknown |
| *Karaops kariyarra* | ♀ | Pilbara | Chichester | late Mar, early April | hot, wet, trans. to dry |
| *Karaops feedtime* | ♀ | Pilbara | Fortescue (border of Chichester) | Mar, Apr | hot, wet, trans. to dry |
| *Karaops forteyi* | ♀♂ | Pilbara | Chichester | unknown | unknown |

The tables below provide data regarding the times and numbers of molting for spiders reared in captivity as well as data showing when adults were collected in nature. It is possible that the molting schedule and time that the spiders in captivity reach adulthood could be completely different in nature and would depend on things like how much they eat, but such information is completely unknown; however, it seems prudent to share these data. The spiders were kept alive to collect biomechanical data and to rear to adulthood so that species could be determined.

Very basic climate data from the areas where the spiders were collected are provided. This assumes that the spiders would become adults in nature as they were being reared in captivity. At first the spiders were kept in chicken egg incubators to regulate the temperature and humidity, but eventually this became tedious and they all remained at room temperature without any apparent consequences (i.e., they did not all die). Additionally, data were collected on how much and when they ate, but this also became tedious and simply took too much time (there were > 100 specimens and many lived > 1 year). Eventually they were just kept in regular plastic collecting vials and were fed a couple of times a week. Some of them refused to eat so they were fed sugar water, which they would drink, usually unwillingly; however, this proved to be a good solution, and most of the time they would return to eating solid food. Sometimes a piece of bark or paper was put into the vial for them to sit on. Overall, they are fairly easy to take care of.

In each table, the top row indicates rainfall, more if bright blue, less if gray blue, and least if white. The second row indicates temperature, higher if red, lowest if blue, and purple is in between. The chart begins with May as this is when the spiders were collected. If a cell is split, it indicates the same month of the following year. Numbers in the cells indicate days that the spiders molted and when they reached adulthood. They were all different instars when collected so there is no way to determine how many molts it takes to get to adulthood. In a species of *Selenops* from the southwestern United States, one spiderling was reared to a penultimate male from the egg sac and has molted 17 times (unpubl. data).

**Table 2.** *Karaops nitmiluk* sp. nov.

|  | Jun | Jul | Aug | Sep | Oct | Nov | Dec | Jan | Feb | Mar | Apr | May |
| --- | --- | --- | --- | --- | --- | --- | --- | --- | --- | --- | --- | --- |
|  |  |  |  |  |  |  |  |  |  |  |  |  |
| sel_1333 | imm., 10 | 14 |  | 15  p♀ | 22, ♀ |  |  |  |  | 2, died |  |  |
| sel_1334 | imm., 21 |  |  | 16,  p♀ | 29,  ♀ |  |  |  |  |  |  |  |
|  | 1 |  |  |  |  |  |  |  |  |  |  |  |
| sel_1335 | imm., 21 | 24 |  | 15 | 20 |  |  | 13 | 26, died |  |  |  |
| sel_1336 | imm. |  |  |  | 13, died molting |  |  |  |  |  |  |  |
| sel_1337 | imm. |  |  | 22 | 20 | 19 Nov, was going to molt soon, died |  |  |  |  |  |  |
| sel_1338 | imm. | 31, died |  |  |  |  |  |  |  |  |  |  |
| sel_1339 | imm. | 25,  p♀ |  |  |  |  | 29,  ♀ |  |  |  |  |  |
|  | 2, died |  |  |  |  |  |  |  |  |  |  |  |
| sel_1340 | imm. |  |  | 30,  died |  |  |  |  |  |  |  |  |
| sel_1341 | imm. |  |  | 22 | 22 |  | 16, died |  |  |  |  |  |
| 1342 | imm. |  |  | 15, p♀ |  |  | 1,  ♀ | 14, died |  |  |  |  |

**Table 3.** *Karaops jawayway* sp. nov.

|  | Jun | Jul | | | Aug | Sep | Oct | Nov | Dec | Jan | Feb | Mar | Apr | May |
| --- | --- | --- | --- | --- | --- | --- | --- | --- | --- | --- | --- | --- | --- | --- |
|  |  |  | | |  |  |  |  |  |  |  |  |  |  |
| sel_1349 | imm. | |  |  | |  | 14 | 24, p♀ |  |  | 3, ♀ |  |  |  |
|  |  | | died 15 |  |  |  |  |  |  |  |  |  |  |  |
| sel_1350 | imm. | |  |  | |  | 10 | 4, 24 | 29 |  | 2 | 8 | 18 | 4, 26 |
|  |  | | 4, 21 | 1, died, (just prior to molting) | |  |  |  |  |  |  |  |  |  |

**Table 4.** *Karaops yumbu* - Tanami

|  | May | Jun | Jul | Aug | Sep | Oct | Nov | Dec | Jan | Feb | | | Mar | | Apr |
| --- | --- | --- | --- | --- | --- | --- | --- | --- | --- | --- | --- | --- | --- | --- | --- |
|  |  |  |  |  |  |  |  |  |  |  | | |  | |  |
| sel_1285 | imm. |  |  |  | 29 |  |  |  | 6 | 2 | | | died mid-mon | |  |
| sel_1286 | imm. |  |  |  |  | 13 |  | 29 |  |  | | | 12 | |  |
|  | 22 | 18 | 26 |  |  |  | 8^,^ died |  |  |  | | |  | |  |
| sel_1287 | imm. |  |  |  | 22 |  |  |  | Late Jan died molting |  | |  | | |  |
| sel_1288 | imm. | 23, 29 | 17 | 23 | 20 |  |  |  |  | |  |  | |  | |
|  | 4 | 18 |  | 10 | 15, died |  |  |  |  | |  |  | |  | |
| sel_1289 | imm. |  |  |  |  | 22, molted, died next day |  |  |  |  | | |  | |  |
| sel_1290 | Imm. |  |  |  |  |  |  | 3, died |  |  | | |  | |  |
| sel_1291 | imm. |  |  |  |  | 1, p♂ |  | 23, ♂ | died, exact date unknown, in care of someone else. |  | | |  | |  |
| sel_1292 | imm. |  |  |  |  | 7 |  | 29, died |  |  | | |  | |  |
| sel_1293 | Imm. |  |  |  | 22, p♂ | 26, ♂ | unclear what day died |  |  |  | | |  | |  |

**Table 5.** *Karaops yumbu* – O Victoria Plain

|  | May | Jun | Jul | Aug | Sep | Oct | Nov | Dec | Jan | Feb | Mar | Apr |
| --- | --- | --- | --- | --- | --- | --- | --- | --- | --- | --- | --- | --- |
|  |  |  |  |  |  |  |  |  |  |  |  |  |
| sel_1283 | imm. |  |  |  | 15 |  | 5 | 16, died |  |  |  |  |
| sel_1284 | imm. |  |  |  | 10 | 7 |  |  | Late Jan, died molting |  | 1, died |  |
| sel_1294 | imm. |  |  | 11 |  | 7 |  |  | 6, died |  |  |  |
| sel_1295 | imm. |  |  |  | 18, 29 |  | 24, p♂ | day unclear,  ♂, died |  |  |  |  |

**Table 6.** *Karaops keithlongbottomi*

|  | May | Jun | Jul | Aug | | Sep | Oct | Nov | Dec | Jan | Feb | Mar | Apr |
| --- | --- | --- | --- | --- | --- | --- | --- | --- | --- | --- | --- | --- | --- |
|  |  |  |  |  | |  |  |  |  |  |  |  |  |
| sel_1244 | p♂ | 25, ♂ |  |  | died | |  |  |  |  |  |  |  |
| sel_1245 | imm. | 19 |  |  |  | |  |  | 18 died molting; p♀ |  |  |  |  |

**Table 7.** *Karaops dejongi* sp. nov.

|  | May | Jun | Jul | Aug | | Sep | Oct | Nov | Dec | Jan | Feb | Mar | Apr |
| --- | --- | --- | --- | --- | --- | --- | --- | --- | --- | --- | --- | --- | --- |
|  |  |  |  |  | |  |  |  |  |  |  |  |  |
| sel_1270 | imm. |  | 7 |  | 29 | |  |  | 1^,^ died |  |  |  |  |
| sel_1271 | imm |  |  |  | 9 | |  |  |  | 10 | 9, died |  |  |
| sel_1272 | p♂ |  |  |  | 22, ♂ | |  | 17, died |  |  |  |  |  |
| sel_1273 | p♀ | 19, ♀ |  |  |  | | 17 died |  |  |  |  |  |  |

**Table 8.** *Karaops dalmanyi* sp. nov.

|  | May | Jun | Jul | Aug | | Sep | Oct | Nov | Dec | Jan | Feb | Mar | Apr |
| --- | --- | --- | --- | --- | --- | --- | --- | --- | --- | --- | --- | --- | --- |
|  |  |  |  |  | |  |  |  |  |  |  |  |  |
| sel_1233 | p♂ |  |  |  | 18, ♂ | |  |  |  |  |  |  |  |
| sel_1234 | p♂ |  |  | 21, ♂ |  | |  | 3,  died |  |  |  |  |  |
| sel_1235 | imm. |  |  | 14 | 23, died | |  |  |  |  |  |  |  |
| sel_1236 | p♀ | 19, ♀ |  |  | 29, died | |  |  |  |  |  |  |  |
| sel_1237 | p♂ |  | 1, ♂ / 31, died |  |  | |  |  |  |  |  |  |  |
| sel_1238 | Imm. | 8 |  |  | 4, died | |  |  |  |  |  |  |  |

**Table 9.** *Karaops jenniferae*

|  | May | Jun | Jul | Aug | | Sep | Oct | Nov | Dec | Jan | Feb | Mar | Apr |
| --- | --- | --- | --- | --- | --- | --- | --- | --- | --- | --- | --- | --- | --- |
|  |  |  |  |  | |  |  |  |  |  |  |  |  |
| WAM T65078 |  |  | 26, ♀ |  |  | |  |  |  |  |  |  |  |
| sel_1274 | imm. |  |  |  | 9^,^ died | |  |  |  |  |  |  |  |
| sel_1275 | imm. |  |  | 14 |  | |  | 24, died |  |  |  |  |  |

**Table 10.** *Karaops malumbu* sp. nov.

|  | May | Jun | Jul | Aug | | Sep | Oct | Nov | Dec | Jan | Feb | Mar | Apr |
| --- | --- | --- | --- | --- | --- | --- | --- | --- | --- | --- | --- | --- | --- |
|  |  |  |  |  | |  |  |  |  |  |  |  |  |
| sel_1305 | imm. | 22,  p♀ |  |  |  | | 3^­^, ♀; 27, died |  |  |  |  |  |  |
| sel_1306 | imm |  |  |  | 22 | |  |  | 2, died |  |  |  |  |
| sel_1307 | imm. | 22 |  |  | 17, p♂ | | died (unsure which day) |  |  |  |  |  |  |
| sel_1308 | imm. | 29  (unsure when died) |  |  |  | | 17 died |  |  |  |  |  |  |
| sel_1309 | p♂ |  |  |  | 18, ♂ | |  | 24, died |  |  |  |  |  |
| sel_1310 | imm. |  |  |  | 4, 29 died | |  |  |  |  |  |  |  |

**Table 11.** *Karaops garyodwyeri* sp. nov.

|  | May | Jun | Jul | Aug | | Sep | Oct | Nov | Dec | Jan | Feb | Mar | Apr |
| --- | --- | --- | --- | --- | --- | --- | --- | --- | --- | --- | --- | --- | --- |
|  |  |  |  |  | |  |  |  |  |  |  |  |  |
| sel_1254 | imm. |  |  |  |  | |  | 17, died |  |  |  |  |  |
| sel_1255 | imm. | 25, p♀, died molting |  |  |  | |  |  |  |  |  |  |  |
| sel_1256 | p♂ | 29, ♂, died molting |  |  |  | |  |  |  |  |  |  |  |
| sel_1257 | imm. |  |  |  |  | | 21,  died |  |  |  |  |  |  |
| sel_1258 | imm. |  |  |  | 29 | |  |  |  |  |  |  |  |
|  | 31 |  |  |  |  |  |  |  |  |  |  |  |  |

**Table 12.** *Karaops yumbubaarnji* sp. nov.

|  | May | Jun | Jul | | Aug | Sep | Oct | Nov | Dec | Jan | Feb | Mar | Apr |
| --- | --- | --- | --- | --- | --- | --- | --- | --- | --- | --- | --- | --- | --- |
|  |  |  |  | |  |  |  |  |  |  |  |  |  |
| sel_1296 | imm. | 10 | 20 |  | |  |  | 19,  died |  |  |  |  |  |
| sel_1297 | imm. |  |  | 31, died | |  |  |  |  |  |  |  |  |
| sel_1298 | imm. |  | 24 |  | |  | 26 |  |  |  |  |  | 5, died |
| sel_1299 | imm. |  |  | 5 | |  |  |  | 1 |  |  | between 12 a 28, died |  |
| sel_1300 | imm. | 19 |  |  | | 9, p♂ |  | 10, ♂ | unclear when died |  |  |  |  |
| sel_1301 | imm. |  |  |  | | 15 |  | 18 |  |  |  | 9 | 5 |
|  | 9 |  |  |  |  |  |  |  |  |  |  |  |  |
| sel_1302 | imm. |  | 25, p♀ |  | |  | 17^,^ ♀ |  | unclear when died |  |  |  |  |
| sel_1303 |  | 22 |  | 22 | |  |  |  |  | 5, died, after molting |  |  |  |
| sel_1304 |  |  |  |  | |  | 3; 14, died |  |  |  |  |  |  |

**Table 13.** *Karaops kennerleyorum* sp. nov.

|  | Jun | Jul | | Aug | Sep | Oct | Nov | Dec | Jan | Feb | Mar | Apr | May |
| --- | --- | --- | --- | --- | --- | --- | --- | --- | --- | --- | --- | --- | --- |
|  |  |  | |  |  |  |  |  |  |  |  |  |  |
| sel_1343 | imm. | |  |  | 9,  p♂ | 13, ♂ | not sure when died |  |  |  |  |  |  |
| sel_1344 | p♀ | |  | 18, died |  |  |  |  |  |  |  |  |  |
| sel_1345 | imm. | |  | 11 | 22,  died |  |  |  |  |  |  |  |  |
| sel_1346 | imm. | |  |  | 15 |  |  | 18; 23, died |  |  |  |  |  |
| sel_1347 | imm. | |  | 11 | 9; 20, died |  |  |  |  |  |  |  |  |
| sel_1348 | imm. | |  |  | 17, died |  |  |  |  |  |  |  |  |

**Table 14.** *Karaops markharveyi* sp. nov.

|  | May | Jun | Jul | | | Aug | Sep | Oct | Nov | Dec | Jan | Feb | Mar | Apr |
| --- | --- | --- | --- | --- | --- | --- | --- | --- | --- | --- | --- | --- | --- | --- |
|  |  |  |  | | |  |  |  |  |  |  |  |  |  |
| sel_1248 | imm. |  | |  | 15, died | |  |  |  |  |  |  |  |  |
| sel_1249 | imm. | 10 | |  |  | | 15 |  | 24, p♀ |  | 14, ♀ |  | 1, died |  |
| sel_1250 | imm. |  | | 9 | 22 | | 29, p♂ | 28, ♂ | unclear when died |  |  |  |  |  |
| sel_1251 | imm. |  | |  |  | | 9 | 7 |  |  | 6 |  |  | 5 died |
| sel_1252 | imm. | 10 | |  |  | | 22, died |  |  |  |  |  |  |  |
| sel_1253 | imm. |  | |  |  | | 15,  p♀ |  |  |  |  | 6, ♀ | died, 12 |  |
| sel_1329 | imm. |  | |  |  | | 30, p♂ |  | 18, ♂ | 30, died |  |  |  |  |
| sel_1330 |  |  | |  |  | | 29, p♂ |  | 10, ♂ | 18, died |  |  |  |  |
| sel_1331 | imm. |  | |  |  | |  |  |  | 9 |  |  |  |  |
|  | 11 |  |  |  |  |  | 5, died |  |  |  |  |  |  |  |
| sel_1332 | imm. |  | |  |  | | 15 | 29, p♀ |  | 16, ♀ | died, exact date unknown, in care of someone else. |  |  |  |

**Table 15.** *Karaops badgeradda*

|  | May | Jun | Jul | | | Aug | Sep | Oct | Nov | Dec | Jan | Feb | Mar | Apr |
| --- | --- | --- | --- | --- | --- | --- | --- | --- | --- | --- | --- | --- | --- | --- |
|  |  |  |  | | |  |  |  |  |  |  |  |  |  |
| T97213 |  |  | |  |  | |  |  |  |  |  |  | ♀ 18 |  |
| T97214 |  |  | |  |  | |  |  |  |  |  |  | ♀ 18 |  |
| T97215 |  |  | |  |  | |  |  |  |  |  |  | ♀ 18 |  |
| T95019 |  |  | |  |  | |  | imm |  |  |  |  |  |  |
| T95020 |  |  | |  |  | |  | imm. |  |  |  |  |  |  |
| sel_1119 | imm. 8 |  | |  |  | | died |  |  |  |  |  |  |  |
| sel_1120 | imm. 8 |  | |  | 19 | | died |  |  |  |  |  |  |  |
| sel_1123 | ♀ 8 |  | |  |  | | died 4 |  |  |  |  |  |  |  |
| sel_1124 | imm. 8 | died | |  |  | |  |  |  |  |  |  |  |  |
| sel_1125 | imm. 8 | 19 | | 22 |  | |  | died |  |  |  |  |  |  |
| sel_1126 | imm. 8 | 19 | |  |  | | 30 |  |  | 1 |  |  | died |  |
| sel_1127 | ♀ 8 |  | |  | died | |  |  |  |  |  |  |  |  |
| sel_1128 | imm. 8 |  | | 29 | 14 | | died |  |  |  |  |  |  |  |
| sel_1129 | imm. 8 |  | | 11 |  | |  | died |  |  |  |  |  |  |

**Table 16.** *Karaops joehaeneri* sp. nov.

|  | May | Jun | Jul | | | Aug | | Sep | Oct | Nov | Dec | Jan | Feb | Mar | Apr |
| --- | --- | --- | --- | --- | --- | --- | --- | --- | --- | --- | --- | --- | --- | --- | --- |
|  |  |  |  | | |  | |  |  |  |  |  |  |  |  |
| sel_1130 | ♀ | died | |  |  | |  | |  |  |  |  |  |  |  |
| sel_1131 | imm. | 28 | |  |  | | 17, p♂ | |  | 10, ♂ |  |  |  |  |  |
| sel_1132 | imm. |  | | 25 | 24 | | died | |  |  |  |  |  |  |  |
| sel_1133 | imm. |  | |  |  | |  | | 20 |  | 18 |  | 12 |  | 18, p♀ |
|  | 9, died (2017) |  |  |  |  |  |  |  |  |  |  |  |  |  |  |
| sel_1134 | imm. |  | |  |  | |  | | 13, p♂ | 10, ♂ |  |  |  |  |  |

**Table 17.** *Karaops morganoconnelli* sp. nov.

|  | May | Jun | Jul | | | Aug | | Sep | Oct | Nov | Dec | Jan | Feb | Mar | Apr |
| --- | --- | --- | --- | --- | --- | --- | --- | --- | --- | --- | --- | --- | --- | --- | --- |
|  |  |  |  | | |  | |  |  |  |  |  |  |  |  |
| T131095 | ♀ |  | |  |  | |  | |  |  |  |  |  |  |  |
| sel_1207 |  |  | |  |  | |  | | 26 | 10, p♂ | 9, ♂ |  | 16, died |  |  |

**Table 18.** *Karaops nyangumarta* sp. nov.

|  | May | Jun | Jul | Aug | Sep | Oct | Nov | Dec | Jan | Feb | Mar | Apr |
| --- | --- | --- | --- | --- | --- | --- | --- | --- | --- | --- | --- | --- |
|  |  |  |  |  |  |  |  |  |  |  |  |  |
| sel_1175 | imm. |  |  |  | died |  |  |  |  |  |  |  |
| sel_1176 | imm. |  | 16 |  |  | 20 |  | died |  |  |  |  |
| sel_1177 | imm. |  | 19 | 31 |  | died |  |  |  |  |  |  |
| sel_1178 | imm. |  | 23 |  |  |  | 24 | 29 |  | 16 |  | 26, died molting |
| sel_1179 | imm. |  | 20,  p♂ |  | 17, ♂ |  |  |  |  |  |  |  |
| sel_1180 | imm. | 22 |  | 19 |  | 27 |  |  |  | 16 |  | 23 |
|  | 30 | 26, died molting |  |  |  |  |  |  |  |  |  |  |
| sel_1181 | imm. |  | 22 |  |  | 27 |  | 1 |  | 9 |  |  |
|  | 4 | 5 |  |  |  | 20 |  |  |  |  |  |  |
| sel_1182 | imm. |  |  |  | 9, died molting |  |  |  |  |  |  |  |
| sel_1191 | imm. |  |  | 2 |  |  |  | died |  |  |  |  |
| sel_1192 | imm. |  |  |  | 4 |  | died |  |  |  |  |  |
| sel_1193 | imm. |  |  |  |  | 20 |  |  |  | 12 |  |  |
|  | died |  |  |  |  |  |  |  |  |  |  |  |
| sel_1194 | imm. | 10 |  | 14 |  |  |  | died |  |  |  |  |
| sel_1195 | imm. |  | 18 | 28 |  | died |  |  |  |  |  |  |
| sel_1197 | imm. molted to p♂ |  |  |  |  | 1, ♂ |  |  |  |  |  |  |

**Table 19.** *Karaops nyiyaparli*

|  | May | Jun | Jul | Aug | Sep | Oct | Nov | Dec | Jan | Feb | Mar | Apr |
| --- | --- | --- | --- | --- | --- | --- | --- | --- | --- | --- | --- | --- |
|  |  |  |  |  |  |  |  |  |  |  |  |  |
| T111455 |  |  |  |  |  |  |  |  |  |  | ♀pitfall art 22 Mar | ♀ pitfall e 29 Apr |
| T103130 | ♀pitfall es 5 May |  |  |  |  |  |  |  |  |  | ♀pitfall art 28 Mar | ♀pitfall rough Apr |
| sel_1166 | ♀ 13 |  |  | died |  |  |  |  |  |  |  |  |
| sel_1167 | ♀ 13 |  |  |  |  |  |  | 11, died |  |  |  |  |
| sel_1171 | imm. 13 |  |  |  |  | 1 | 10 |  |  | 12, p♂ | ♂ mid | died |
| sel_1213 | ♀ 16 |  |  |  | died |  |  |  |  |  |  |  |
| sel_1230 | ♀ 17 |  |  |  |  |  | 24, died |  |  |  |  |  |
| sel_1159 | imm. 12 |  |  |  | 24 |  | 18 | 23 |  | died molting |  |  |
| sel_1160 | imm. 12 |  | died |  |  |  |  |  |  |  |  |  |
| sel_1161 | imm. 12 | molt | 20 | 28, died |  |  |  |  |  |  |  |  |
| sel_1162 | imm. 12 | molt |  |  |  |  |  |  | died |  |  |  |
| sel_1163 | imm. 12 | molt |  |  |  | 1 |  |  | died |  |  |  |
| sel_1164 | imm. 12 |  |  |  |  |  |  | 16, died |  |  |  |  |
| sel_1165 | imm. 12 |  | 5 |  |  |  | 10 |  |  |  | died |  |
| sel_1168 | imm. 13 |  | 5 |  |  | 7 |  |  |  | 6 |  |  |
|  |  | 28, died |  |  |  |  |  |  |  |  |  |  |
| sel_1169 | imm. 13 | 29 | 4 |  |  | 7 | 24 | 16, died (accident) |  |  |  |  |
| sel_1170 | imm. 13 |  | 27 |  |  | 26 |  |  | 13 |  | 29 | 20 |
|  |  | 22 |  | 7, died (accident) |  |  |  |  |  |  |  |  |
| sel_1172 | imm. 13 |  |  |  |  | 1 |  | 29 |  | 16 |  | 10 |
|  | 9, died molting |  |  |  |  |  |  |  |  |  |  |  |
| sel_1214 | imm. 16 | 10 | 1 |  |  |  | died |  |  |  |  |  |
| sel_1216 | imm. 16 |  |  | 11 |  |  |  | died |  |  |  |  |
| sel_1217 | imm. 16 |  |  | 1 |  | died (accident) 27 |  |  |  |  |  |  |
| sel_1218 | imm. 16 |  |  |  |  |  | died 3 |  |  |  |  |  |
| sel_1219 | imm. 16 |  | 5 |  |  | died |  |  |  |  |  |  |
| sel_1220 | imm. 16 |  | 7 |  |  |  | 17, died |  |  |  |  |  |
| sel_1228 | imm. 17 | 13 |  | 20 |  | 14 | 3, 18 | 16 |  | 9, p♂ |  |  |
|  | 14, died |  |  |  |  |  |  |  |  |  |  |  |
| sel_1229 | imm. 17 |  |  |  | died |  |  |  |  |  |  |  |
| sel_1231 | imm. 17 |  | 15 |  |  | 22 |  | 9, died molting |  |  |  |  |

**Table 20.** *Karaops durrantorum* sp. nov.

|  | May | Jun | Jul | Aug | Sep | Oct | Nov | Dec | Jan | Feb | | Mar | Apr |
| --- | --- | --- | --- | --- | --- | --- | --- | --- | --- | --- | --- | --- | --- |
|  |  |  |  |  |  |  |  |  |  | |  |  |  |
| sel_1135 | imm. |  | 19 |  | 5 |  |  |  | 10 | | 20 |  |  |
|  | 1 | 5, 26 |  | 8 |  | 25, died |  |  |  |  |  |  |  |
| sel_1136 | imm. |  |  |  | 22, p♂ | 28, ♂ |  |  |  | |  |  |  |
| sel_1137 | imm. |  | 19 |  |  | 20; died late Oct |  |  |  | |  |  |  |
| sel_1138 | imm. |  | 29 |  |  | 7 | 24 |  |  | | 22, died molting |  |  |
| sel_1139 | imm. | 5 |  |  | 5 | 26, died |  |  |  | |  |  |  |
| sel_1140 | imm. |  |  |  | 4 | died mid-mon |  |  |  | |  |  |  |

**Table 21.** *Karaops banyjima*

|  | May | Jun | Jul | Aug | Sep | Oct | Nov | Dec | Jan | Feb | Mar | Apr |
| --- | --- | --- | --- | --- | --- | --- | --- | --- | --- | --- | --- | --- |
|  |  |  |  |  |  |  |  |  |  |  |  |  |
| sel_1173 | imm. |  | 15 |  |  |  | died |  |  |  |  |  |
| sel_1174 | p♂ |  |  |  |  | 14, ♂ |  |  |  |  |  |  |
| sel_1183 | imm. |  |  |  | 23, died molting |  |  |  |  |  |  |  |
| sel_1184 | imm. |  |  | 12 |  |  |  | 11  died molting |  |  |  |  |
| sel_1185 | imm. | 19 | 19 |  | 17 | 26 | 18 died molting |  |  |  |  |  |
| sel_1186 | imm. | 25 |  |  |  |  | 10 |  | 16 |  | 10, died |  |
| sel_1187 | imm. |  | 15 |  | 5,  p♂ | 22, ♂ |  |  |  |  |  |  |
| sel_1188 | imm. |  | died |  |  |  |  |  |  |  |  |  |
| sel_1189 | imm. |  |  | 11 |  | 27 |  |  | 16 | 23 | 28 |  |
|  | 1, 22 | 17, 26 |  | died, accident |  |  |  |  |  |  |  |  |
| sel_1190 | imm. | 28 | 19 |  | 9 | died |  |  |  |  |  |  |
| sel_1198 | p♂ |  |  |  | 22, ♂ |  |  |  |  |  |  |  |
| sel_1199 | imm. |  |  |  |  |  | died |  |  |  |  |  |
| sel_1200 | imm. |  |  |  |  | died |  |  |  |  |  |  |
| sel_1201 | imm. |  | 19 |  |  | died |  |  |  |  |  |  |
| sel_1202 | imm. |  | 19 |  |  |  | 4 | 23 died |  |  |  |  |
| sel_1203 | imm. |  | 27, p♂ | 1, died |  |  |  |  |  |  |  |  |
| sel_1204 | imm. |  | 11 |  | 17, 29 died |  |  |  |  |  |  |  |
| sel_1205 | imm. |  | 4 |  |  | 14 | 3, 18, died |  |  |  |  |  |
